# Expressing banana transcription factor MaERFVII3 in *Arabidopsis* confers enhanced waterlogging tolerance and root growth

**DOI:** 10.7717/peerj.17285

**Published:** 2024-04-30

**Authors:** Ee Yang Teoh, Chee How Teo, Nadiya Akmal Baharum, Boon Chin Tan

**Affiliations:** 1 Centre for Research in Biotechnology for Agriculture, Universiti Malaya, Kuala Lumpur, Malaysia; 2 Institute for Advanced Studies, Universiti Malaya, Kuala Lumpur, Malaysia; 3 Department of Cell and Molecular Biology, Faculty of Biotechnology and Biomolecular Sciences, Universiti Putra Malaysia, UPM Serdang, Selangor, Malaysia

**Keywords:** Abiotic stress, Banana, Crop improvement, Ethylene responsive factors, Waterlogging stress, Genetic engineering

## Abstract

**Background:**

Waterlogging poses a significant threat to plant growth and yield worldwide. Identifying the genes responsible for mitigating waterlogging stress is crucial. Ethylene-responsive factors (ERFs) are transcriptional regulators that respond to various biotic and abiotic stresses in plants. However, their roles and involvement in responding to waterlogging stress remain largely unexplored. Hence, this study aimed to elucidate the role of ERFs in enhancing banana plant resilience to waterlogging.

**Methods:**

We hypothesized that introducing a group VII ERF transcription factor in *Arabidopsis* could enhance waterlogging stress tolerance. To test this hypothesis, we isolated *MaERFVII3* from banana roots, where it exhibited a significant induction in response to waterlogging stress. The isolated *MaERFVII3* was introduced into *Arabidopsis* plants for functional gene studies.

**Results:**

Compared with wild-type plants, the *MaERFVII3*-expressing *Arabidopsis* showed increased survival and biomass under waterlogging stress. Furthermore, the abundance of transcripts related to waterlogging and hypoxia response showed an elevation in transgenic plants but a decrease in wild-type and empty vector plants when exposed to waterlogging stress. Our results demonstrate the significant contribution of *MaERFVII3* to waterlogging tolerance in *Arabidopsis*, providing baseline data for further exploration and potentially contributing to crop improvement programs.

## Introduction

Soil waterlogging affects crop growth and productivity worldwide. It has become more frequent and unpredictable, severely affecting about 12% of global arable land (Tian et al., 2021). The adverse effects of waterlogging have been observed in several countries, especially Southern Asia countries (Wang et al., 2021a). For example, about USD18.9 million of agriculture loss and damage was recorded in Malaysia in 2006 due to flooding (Alam et al., 2020). Between 2010 and 2014, Pakistan experienced devastating losses of about 11 billion tons of commercially important crops, such as rice (*Oryza sativa*), sugarcane (*Saccharum officinarum*), maize (*Zea mays*), and cotton (*Gossypium* spp.), valued at over 16 billion dollars due to flooding (Rehman et al., 2015). Besides, depending on the severity of the damage, resuming farming after a flooding event often requires an efficient recovery plan to remediate productive lands and replanting if the plant damage is lethal (Fukao et al., 2019).

Most plants are sensitive to waterlogging, primarily due to the low oxygen conditions created by soil waterlogging, which limits their access to carbon dioxide and oxygen. As a result, photosynthesis, root respiration, and the mitochondrial electron transport chain are inhibited, leading to energy shortages (Xiao & Jespersen, 2019; Xie et al., 2021). This waterlogging-induced energy deficiency disrupts various processes related to ion selectivity, active ion transport, and root and shoot growth. In response to waterlogging stress, plants adapt various responses, including morpho-physiological and molecular changes, to relieve root respiratory depression and damage caused by disrupted cellular processes and metabolisms under waterlogging. A notable adaptive morphological change is the formation of adventitious roots (Teoh et al., 2022). During waterlogging stress, plants develop adventitious roots, which contain more aerenchymas than primary roots. This alteration facilitates gas exchange as well as water and nutrient absorption. Besides changing their morpho-physiology, plants also undergo modifications in hormonal balance, gene expression and enzyme activities in response to waterlogging stress. For instance, exposure of plants to waterlogging stress led to an increase in ethylene and abscisic acid concentrations (Jurczyk et al., 2021; Qi et al., 2019), while a reduction of cytokinins was observed (Hu et al., 2022). Furthermore, the genes associated with these hormones exhibited significant differential expression in waterlogged plants (Ding et al., 2022; Teoh et al., 2022). These findings show that plant hormones are involved in adaptive responses to waterlogging, indicating their integral role in enhancing plant tolerance to waterlogging stress. Besides plant hormones, waterlogging also induces an upregulation in the expression of many transcription factors (TFs) (Gao et al., 2022; Wang et al., 2021b). For example, sesame cultivars exposed to waterlogging stress demonstrated increased expression of ethylene-responsive factors (ERFs) and WRKYs (Wang et al., 2021a).

ERF is part of the APETALA2/Ethylene Responsive Factor (AP2/ERF) superfamily, one of the largest gene families in plants (Nakano et al., 2006; Zhou, Tang & Wu, 2012). ERF involves signal transductions in biotic and abiotic stress responses (Yang et al., 2022; Zhang et al., 2022). Based on its conserved domains, AP2/ERF can be divided into three major families: AP2, which contains two AP2/ERF domains; ERF, containing a single AP2/ERF domain; and RELATED TO ABSCISIC ACID INSENSITIVE3/VIVIPAROUS1 (RAV) composed of an AP2/ERF domain and a B3 domain (Lakhwani et al., 2016; Nakano et al., 2006). The ERF subfamily can be divided into subgroups based on their homologous motifs. For example, in *Arabidopsis*, the 163 ERFs are divided into 12 subgroups, while in rice, the 144 ERFs are categorized into 15 subgroups (Nakano et al., 2006). Among the members of the ERF family, group VII (ERFVII), such as *HYPOXIA RESPONSIVE ERF1 (HRE1)*, *HRE2*, *RELATED TO APETALA2.12 (RAP2.12)*, *RAP2.2*, and *RAP2.3*, are crucial regulators of hypoxic responses (Luan et al., 2020; Zhou et al., 2019). For example, the knockout of *erfvii* in *Arabidopsis thaliana* increased hypoxia sensitivity (Licausi et al., 2010), while overexpressing *ERFVII* in wheat increased waterlogging tolerance without negatively impacting grain yield (Wei et al., 2019). ERFVII regulates stress responses mainly through the direct binding to the GCC-box and/or the hypoxia-responsive promoter element (HRPE), which are triplicates of 33 bp motifs present in 39 of 49 core hypoxia-responsive genes (Gasch et al., 2016; Giuntoli & Perata, 2018). However, some members exhibit distinct binding sites. For example, AtRAP2.1216 specifically recognizes the ATCTA motif in the promoter of downstream genes (Licausi et al., 2011).

Since many crops are sensitive to waterlogging stress, they develop a strategy to alleviate the adverse effects of waterlogging stress to improve their survival and growth. Therefore, identifying genes associated with waterlogging stress is crucial in understanding and improving this adaptive strategy. Bananas, a *Musa* genus member, are the world’s fourth most important food crop (Tan, 2022). Originating in Southeast Asia (Chin et al., 2014), bananas are well known for their high nutritional content. Among their many cultivars, the triploid banana cultivar (*Musa acuminata* AAA) is one of the most popular commercial cultivars because of its sweet taste and fruit quality (Lau et al., 2023). However, like other AAA cultivars, Berangan is vulnerable to waterlogging stress, emphasizing the need for new stress-resistant banana varieties. Our previous transcriptomics data showed that *MaERFVII3* exhibited significant expression levels among identified TFs in waterlogged bananas (Teoh et al., 2022). Considering the established roles of *MaERFVII3* homologs in conferring tolerance to various environmental stresses, such as drought and salinity, in various plant species, we hypothesize that introducing *MaERFVII3* in *Arabidopsis* could enhance its tolerance to waterlogging stress. To test our hypothesis, we isolated the full-length cDNA of *MaERFVII3* from bananas, cloned it into a plant expression vector, and introduced it into *Arabidopsis* for functional gene study.

## Materials and Methods

### Alignment and phylogenetic analysis

The candidate ERFVII sequences were aligned using ClustalW software (Kyoto University Bioinformatics Center, Kyoto, Japan). The exon-intron structure of ERFVII was analyzed by comparing gene sequences from five plant species: *Arabidopsis thaliana*, tomato (*Solanum lycopersicum*), soybean (*Glycine max*), banana (*Musa acuminata*), and rice (*Oryza sativa*), using Gene Structure Display Server (GSDS) (https://gsds.gao-lab.org/). Protein sequences of *Arabidopsis* and rice were obtained from the *Arabidopsis* Information Resource (TAIR, https://www.arabidopsis.org/index.jsp) and the Oryzabase (https://shigen.nig.ac.jp/rice/oryzabase/), respectively. The ERFVII protein sequences of the ten other plant species were obtained from NCBI (Table S1). To examine the phylogenetic relationship, a phylogenetic tree was constructed with MEGA X (version 10.2.4). Multiple sequence alignment was performed using the default parameters of ClustalW in the MEGA X program. The phylogenetic tree was generated using the maximum likelihood method and Jones-Taylor-Thornton (JTT) amino acid substitution model with a bootstrap value of 1,000 using the default parameters.

### Cloning of *MaERFVII3* and generation of transgenic *Arabidopsis* plants

The full-length open reading frame of *MaERFVII3* (Macma4_02_g02290) was amplified from the cDNA of *Musa acuminata* cultivar Berangan roots using high-fidelity Q5 DNA polymerase. The PCR reactions were carried out as follows: 95 °C (3 min) for one cycle and 95 °C (15 s), 60 °C (30 s), and 72 °C (30 s) for 35 cycles, and a final extension of 72 °C (20 min) for one cycle using forward primer 5′-ATGTGTGGCGGC GCCATCAT-3′ and reverse primer 5′-CCATGGCGCCGGCAGAATGTCGTCGAA-3′ containing a *Nco*I site (the underlined nucleotides). The PCR product was cloned at the *Nco*I site of pCAMBIA1301 to form *MaERFVII3*:pCAMBIA1301 vector. *Agrobacterium tumefaciens* LBA4404 harboring pCAMBIA1301 or *MaERFVII3*:pCAMBIA1301 was used to infect *Arabidopsis thaliana via* floral dip transformation as described by Clough & Bent (1998). All transformants were cultured on half-strength Murashige and Skoog (MS) medium supplemented with 25 mg/l hygromycin, 0.5% (w/v) Gelrite^™^, and 3% (w/v) sucrose before harvesting for PCR analysis (Table S2). Three independent lines with the segregation ratio of 3:1 at the T_3_ generation were used for the subsequent experiment.

### Subcellular localization

The subcellular localization was conducted according to Simmons, VanderGheynst & Upadhyaya (2009). The *Agrobacterium* strain LBA4404 harboring p35S::*MaERFVII3*-pCAMBIA1304 and pCAMBIA1304 constructs were cultured in 50 ml YEB broth containing 50 mg/l kanamycin, 100 mg/l streptomycin, and 50 mg/l rifampicin. The *Agrobacterium* cell suspension was grown overnight until reaching OD_600_ of 1.0–1.5. The culture was then diluted to a final OD_600_ of 0.5 and collected through centrifugation. The cells were resuspended in full-strength MS medium containing 0.2 mM MES buffer (pH 7.0), 5% (w/v) sucrose, and 200 μM acetosyringone. The surface sterilized onion epidermal layer with 70% (v/v) ethanol for 2 min was immersed in *Agrobacterium* culture. Then, a vacuum was applied at −0.08 M Pa for 10 min before slowly releasing. The onion epidermal cells were placed on MS medium without antibiotics for 2 days. The onion epidermal cells were examined using an Olympus IX73 inverted microscope with the following excitation or emission settings: 488/505–550 nm for green fluorescent protein (GFP). Images were processed using CellSens imaging software (Olympus, Japan).

### Histochemical GUS assay

GUS assay was carried out as described by Jefferson, Kavanagh & Bevan (1987). The freshly cut leaves of transgenic *Arabidopsis* plantlets were used to verify the GUS expression. The X-Gluc (5-bromo4-chloro-3-indoyl-beta-d-glucuronide) was prepared by adding 1 ml of dimethylformamide to 5 mg of X-Gluc. X-Gluc staining buffer contained 0.1 M sodium phosphate buffer (pH 7.0), 0.1% (v/v) Triton X-100, 2 mM dimethyl sulfoxide (DMSO), 1 mM of potassium ferricyanide and 10 mM EDTA (pH 8.0). The leaf samples and 2-week-old *Arabidopsis* seedlings were immersed in 1 ml of X-Gluc staining buffer in a 1.5 ml sterile centrifuge tube. The tubes containing the buffer and sample were placed into a bell jar under a vacuum. A vacuum (−0.08 M Pa) was applied for 10 min and then slowly released. The samples were stained in 1 ml of GUS reaction mixture for at least 16 h at 37 °C. Parafilm was used to seal the tubes to prevent evaporation. The samples were then soaked in 70% (v/v) ethanol at room temperature for at least 5 min or until chlorophyll was completely destained. The GUS stain of the sample was visualized against a white background.

### Waterlogging treatment and morphological measurements

Two-week-old *A. thaliana* cv. Col-0 obtained from Plant Biotechnology Research Lab (PBRL), Universiti Malaya, were transferred to a soil pot when true leaves were formed, where each pot contained four seedlings. Plants were maintained in a growth chamber for a long day photoperiod (16 h light/8 h dark) at 20 °C. Plants were watered once every 4 days and fertilized with foliar fertilizer once a week. Waterlogging treatment was conducted according to our previous study (Teoh et al., 2022). Briefly, the pots containing plants of uniform size at 10-leaf rosette formation were gently placed in plastic containers (60 cm × 44 cm × 20 cm). Soil-grown plants were waterlogged for 1, 3, and 5 days. Plants were flooded with tap water in containers until the water level was 1 cm above the soil level. Well-watered plants were watered once every 4 days. The difference in rosette area was assessed based on the image difference before and after the waterlogging period using the ImageJ software. After the waterlogging stress treatment, plants were harvested and washed with tap water until no soil debris remained. The primary root length was recorded by measuring the longest length of the root. The *Arabidopsis* plantlets were snap-frozen immediately in liquid nitrogen and stored at −80 °C for the subsequent qPCR experiment.

### RNA extraction and gene expression

Total RNA from wild-type and *Arabidopsis* containing pCAMBIA1301 or *MaERFVII3*:pCAMBIA1301 was extracted from the whole plant, according to Teoh et al. (2022). The extracted RNA was treated with DNase I (Qiagen, Hilden, Germany) according to the manufacturer’s protocol. DNAse-treated RNA was measured with a nano-spectrophotometer (Implen, Westlake Village, CA, USA). RNA with a yield above 50 ng/μl and an A280/260 value between 1.8 and 2.0 were used for the subsequent experiments. The presence of genomic DNA and RNA integrity were verified through gel visualization. The total RNA (500 ng) of each biological replicate was reverse transcribed using the NxGEN M-MuLV Reverse Transcriptase cDNA synthesis kit (Lucigen, UK; Catalogue no: 30222-1). Briefly, the RNA was incubated at 65 °C for 5 min, then added to a reaction mix containing 1 × NxGen M-MuLV Reverse Transcriptase buffer, 2 mM dNTP, and 40 µg/ml oligo(dT)_12–18_ to the final volume of 10 µl. The reaction mixture was heated to 25 °C for 2 min, and 200 U of M-MuLV reverse transcriptase was added and gently mixed through pipetting. The reaction was incubated at 42 °C for 60 min, followed by the inactivation of the enzyme by incubating at 85 °C for 10 min. The cDNA was stored at −20 °C until future use. Quantitative RT-PCR was performed using a SYBR green mix in a 7,500 Fast Real-Time PCR system (Applied Biosystems, Waltham, MA, USA) using a 7,500 real-time PCR system v2.0.6 program. All the primers were designed using Primer Blast from NCBI (Table 1). The qPCR reaction contained a final volume of 15 μl containing 2 × SG Fast qPCR Master Mix (Sangon Biotech, Shanghai, China), 0.3 μM forward and reverse primers, and 10 ng cDNA. The qPCR reactions were carried out as the following: 95 °C (3 min) for one cycle and 95 °C (3 s), 60 °C (30 s), followed by plate read, for 40 cycles. *AtTUB* and *AtUBQ* were used as internal controls. The gene expression was calculated using the Pffafl method across three biological replicates and three technical replicates per sample.

**Table 1:** Primer sequences used for qPCR analysis.

Primer name	NCBI accession number	Forward primer sequence (5′-3′)	Reverse primer sequence (5′-3′)	Product length (bp)
AtTUB	NM_125665.4	GGTTGGTTTTGCTCCTCTCACC	TAGCGTCCGTGCCTTGGG TC	129
AtUBQ	NM_001084884.5	GGCCTTGTATAATCCCTGATGAATAAG	AAAGAGATAACAGGAACGGAAACATAG T	61
AtRAP2.12	AT1G53910	TGCTGGATGTAATGGGTATCAG	CAGAAGAGATGTCGGGAGTTATC	113
AtRAP2.2	AT3G14230	CATGGAAGAGAAGCCTCAGATG	GCCCTGATCGGAACTGAAATA	103
AtHRE2	AT2G47520	GAAGCGTAA ACCCGT CTCAGT	TTTGCTCGGGCACGAATCT	123
AtADH1	AT1G77120	TATTCGATGCAAAGCTGCTGTG	CGAACTTCGTGTTTCTGCGGT	93
AtPIN1	AT1G73590	ACAAAACGACGCAGG CTA AG	AGCTGGCATTTCAATGTTCC	164
AtLBD16	AT2G42430	TCAAACCGGAGGAGG AGTAT	AGC CTG AAG CTCACCTAAATC	112
AtLBD18	AT2G45420	GAAGTGTGTGCCGGGATGTA	CGTTACTGGCTCCGAACACT	98

### Statistical analyses

Statistical analyses were conducted using SPSS 16.0 (Chicago, IL, USA). All data were analyzed statistically by one-way analysis of variance (ANOVA), followed by Duncan’s *post-hoc* test and independent *t*-test at a significance level of *p* < 0.05.

## Results

### Phylogenetic tree and domain analysis of MaERFVII3

We previously found that an APETALA2/ethylene-responsive factor named *MaERFVII3* was differentially expressed in waterlogged bananas than in the well-watered plants (Teoh et al., 2022). To characterize the *MaERFVII3*, we isolated the full-length cDNA of *MaERFVII3* from the banana roots. The sequence analysis showed that the full-length cDNA of *MaERFVII3* is 696 bp in length, encoding a protein of 232 amino acids, with a predicted molecular weight of about 26 kDa and an isoelectric point of 5.59 (Fig. S1). The deduced MaERFVII3 amino acid sequence contains a conserved N-terminal motif (MCGGAI/L) and an AP2 domain at 57-114 positions, indicating that MaERFVII3 is a member of the ERFVII group (Fig. S2).

The alignment analysis revealed that residues Gly-4, Arg-6, Arg-8, Gly-11, Ile-17, Asp-19, Pro-20, Arg-25, Trp-27, Gly-29, Thr-30, Ala-37, Ala-38, Ala-40, Tyr-41, Asp-42, and Gly-50, are completely conserved among the 75 proteins (Table S1), while the amino acid residues Arg-3, Pro-9, Trp-10, Trp-13, Ala-14, Ala-15, Glu-16, Arg-18, Lys-22, Gly-23, Val-26, Leu-28, Ala-34, Glu-35, Ala-45, and Arg-49 are conserved in at least 90% of the sequence (Fig. S2).

**Figure 1:**
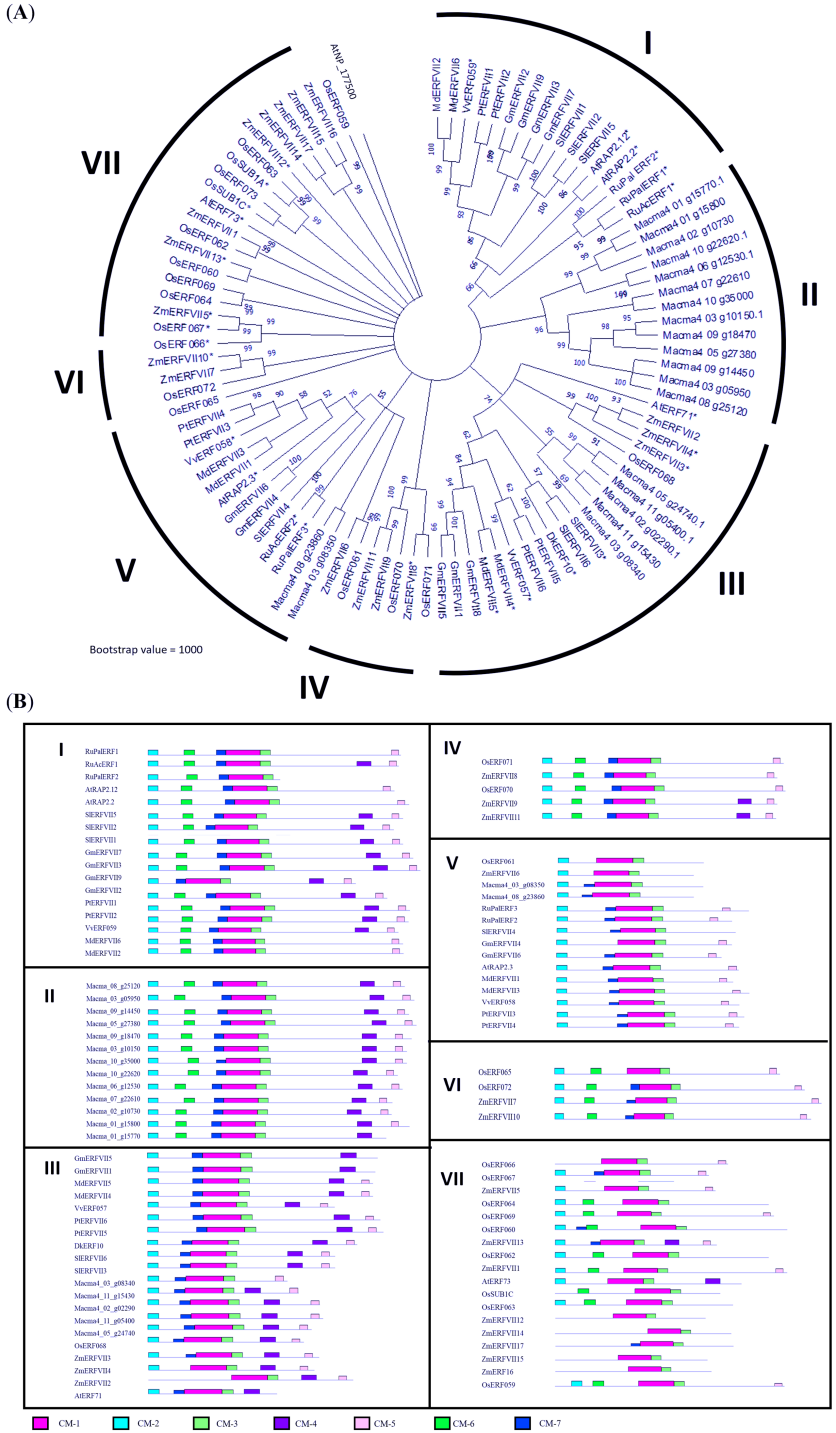
Phylogenetic analysis of MaERFVII protein. (A) Amino acid sequences of group VII ERF members from 11 plant species (*Musa acuminata, Arabidopsis thaliana, Zea mays, Oryza sativa, Solanum* *lycopersicum, Rumex acetosa, Rumex palsturis, Glycine max, Malus domestica, Diospyrus kaki, Populus trichocarpa*, and *Vitis vinifera*) were used with branch length proportional to estimated sequence distance. The scale bar represents 0.2 expected amino acid residue substitution per site. (B) Conserved motif (CM) sites based on ERFVII protein clusters of 11 plant species arranged according to the protein clusters from the phylogenetic tree. Motifs were identified using MEME software and marked according to the legend on protein sequences. The length and order of each motif correspond to the actual length and position of protein sequences.

The BLAST search revealed that MaERFVII3 shared a high sequence identity with its homologues from *Zingiber officinale* (82.92%) and *Phoenix dactylifera* (87.11%). We then constructed a phylogenetic tree to investigate the evolutionary relationship of *MaERFVII3* with its homologs from other plant species (Fig. 1). These include *M. acuminata*, *A. thaliana*, *Zea mays*, *Oryza sativa*, *Solanum lycopersicum*, *Rumex acetosa*, *Rumex palsturis*, *Glycine max*, *Malus domestica*, *Diospyrus kaki*, *Populus trichocarpa*, and *Vitis vinifera*. The exon-intron structure analysis of ERFVII was also performed to support the phylogenetic analysis (Fig. S3).

**Figure 2:**
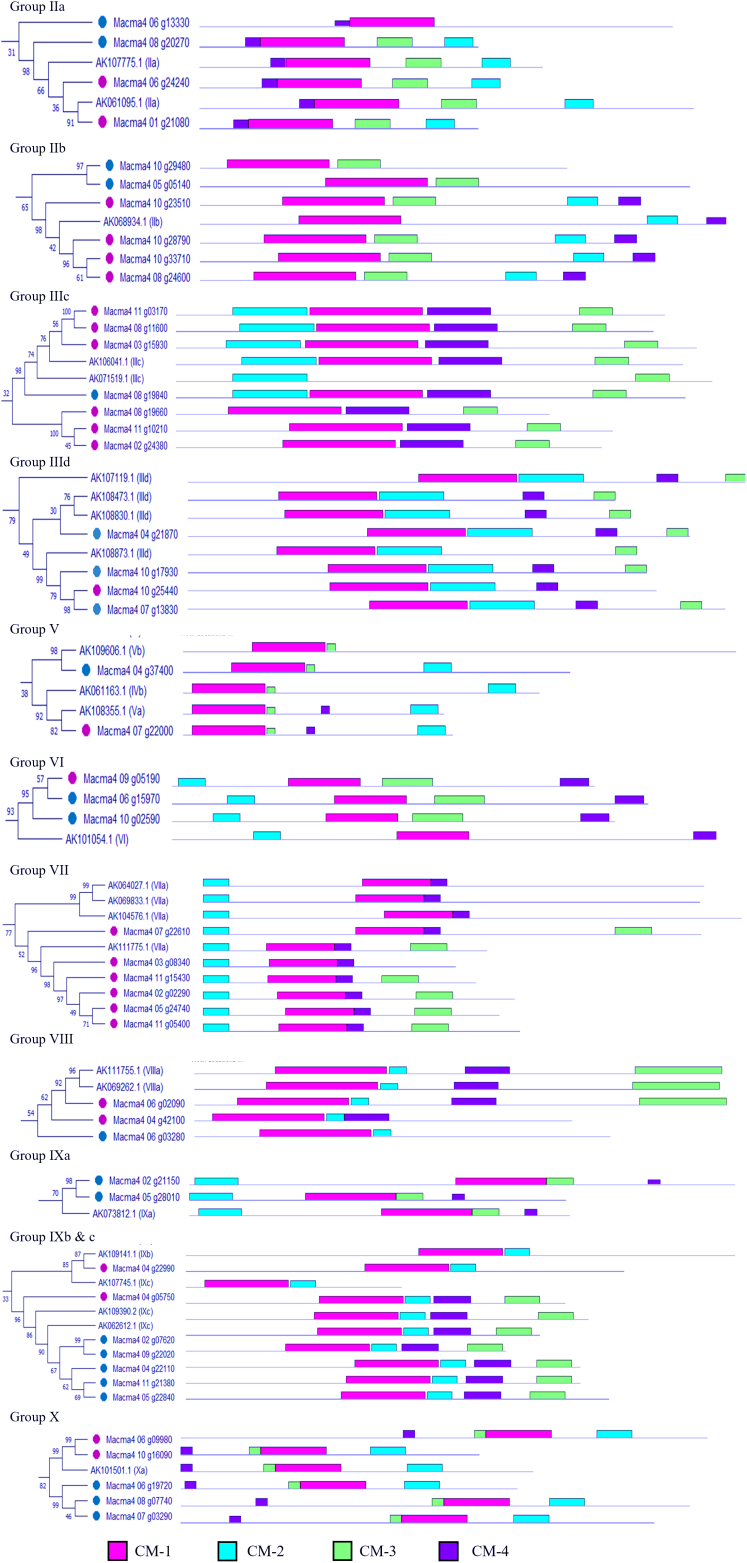
Phylogenetic relationships among differentially expressed *Musa acuminata* ERF compared to rice ERF sequence. Bootstrap values from 1,000 replicates were used to assess the robustness of the tree. The pink circle indicates upregulated genes, while the blue circle indicates downregulated genes during waterlogging stress (Teoh et al., 2022). Each colored box represents conserved domains within the groups. The amino acid sequences of the conserved motifs from each group are summarized in Table S4.

A phylogenetic tree was constructed based on the amino acid sequences of 67 differentially expressed ERF obtained from our previous study (Teoh et al., 2022) (Fig. 2). The phylogram was differentiated into 11 groups, namely subgroup IIa (four genes), IIb (six genes), IIIc (seven genes), IIId (four genes), V (two genes), VI (three genes), VII (six genes), VIII (three genes), IXa (two genes), IXb & c (seven genes), and X (five genes). Domain analysis revealed that proteins within the same group showed structural similarities (Table S3). All ERF TFs possess the AP2 domain, represented by the pink bar conserved motif (CM) −1, followed by the conserved domain specific to the ERF family (Fig. 2). Additionally, some motifs were only present in specific subclades. For example, proteins in Group II commonly possess CMII-2 and CMII-3 adjacent to the AP2/ERF protein conserved at the C-terminal. These motifs are similar to the ERF-associated amphiphilic repression (EAR) motif, (L/F)DLN(L/F)xP, at the C-terminal of the protein sequence. The EAR domain was also present in Group VIII genes as the CMVIII-2 and CMVIII-3 domains, where two out of three genes in this group were differentially downregulated in our previous study (Teoh et al., 2022). All proteins in the Group III contain CMIII-3 and CMIII-4 motifs, reported as LNFP and D(I/V)QAA, neighboring the AP2/ERF domain. Seven out of 11 Group III ERF genes were upregulated in bananas under waterlogging treatment (Fig. 2; Teoh et al., 2022). All genes within the Group VII ERF family, with the conserved MCGGAI(I/L) motif in the N-terminal and the KKA(K/R)(V/L)NFP sequence, were upregulated. Among these ERF TFs, the *MaERFVII3* gene expression (Macma4_02_g02290) showed the highest (Teoh et al., 2022), indicating that this TF might be critical in the waterlogging response.

### Generation of *MaERFVII3*-expressing *Arabidopsis*

**Figure 3:**
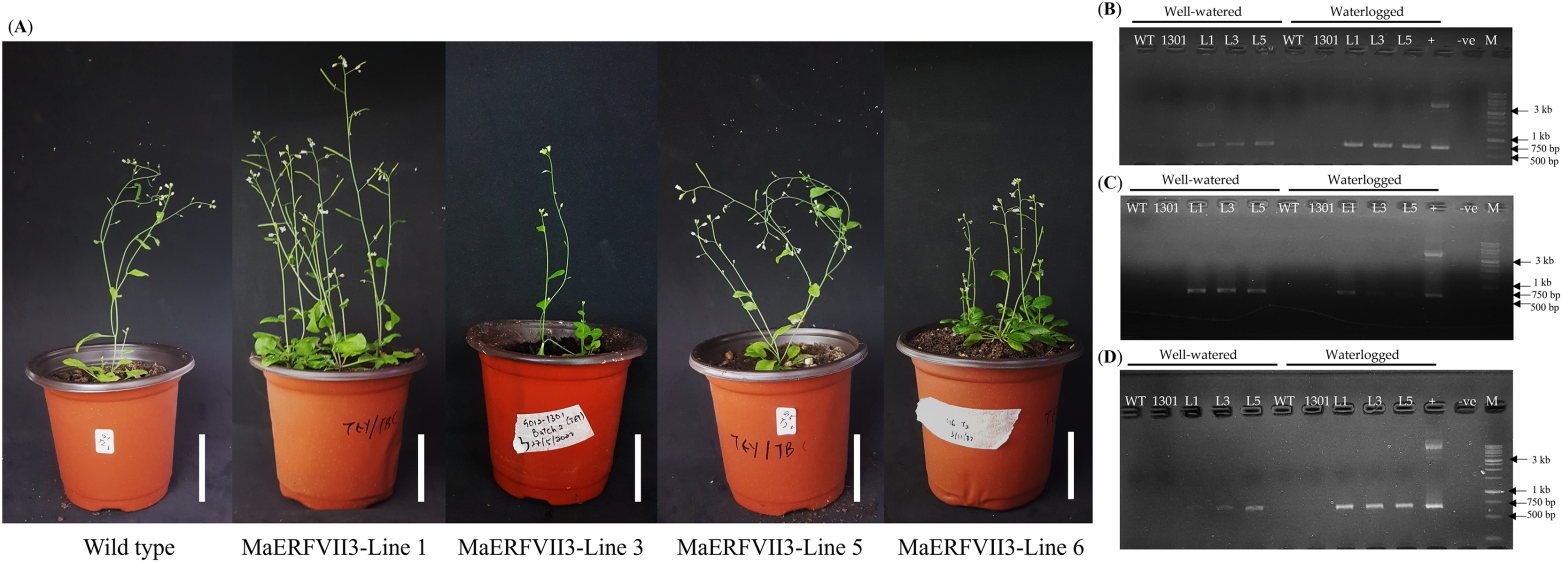
Six-week-old wild-type and transgenic *Arabidopsis* plantlets containing MaERFVII3. (A) Six-week-old *Arabidopsis* wild-type plantlets (left) and transgenic plantlets containing MaERFVII3 (right). The scale bar represents 3 cm. Detection of the presence of MaERFVII3 in each T_3_ genotype sample of (B) Day 1, (C) Day 3, and (D) Day 5 samples. ‘+’ indicates positive control, while ‘−ve’ indicates negative control. M represents the ladder for 1 kb ladder. (WT; Wild-type, 1301; pCAMBIA1301 control, L1; MaERFVII3-Line 1, L3; MaERFVII3-Line 3, L5; MaERFVII3-Line 5).

To investigate the biological function of *MaERFVII3*, we generated *Arabidopsis* lines expressing *MaERFVII3* (Lines 1, 3, and 5) by introducing the constructed *MaERFVII3*:pCAMBIA1301 plasmid (Fig. 3A). Phenotypically, we observed minimal differences between the shoots of wild-type and transgenic plants under well-watered conditions. After verifying the *MaERFVII3* sequence (Fig. S4), the constructed plasmid was transformed into *Arabidopsis* plantlets *via* the *Agrobacterium*-mediated transformation method. The DNA of the generated transformants were extracted and amplified using the *MaERFVII3*-specific and hygromycin primers (Fig. S5). All transgenic lines produced a single band at the expected size of 696 bp, indicating the successful integration of the transgene into the *Arabidopsis* plants until the T_3_ generation (Figs. 3B–3D).

### Subcellular localization and GUS assay of MaERFVII3 protein

**Figure 4:**
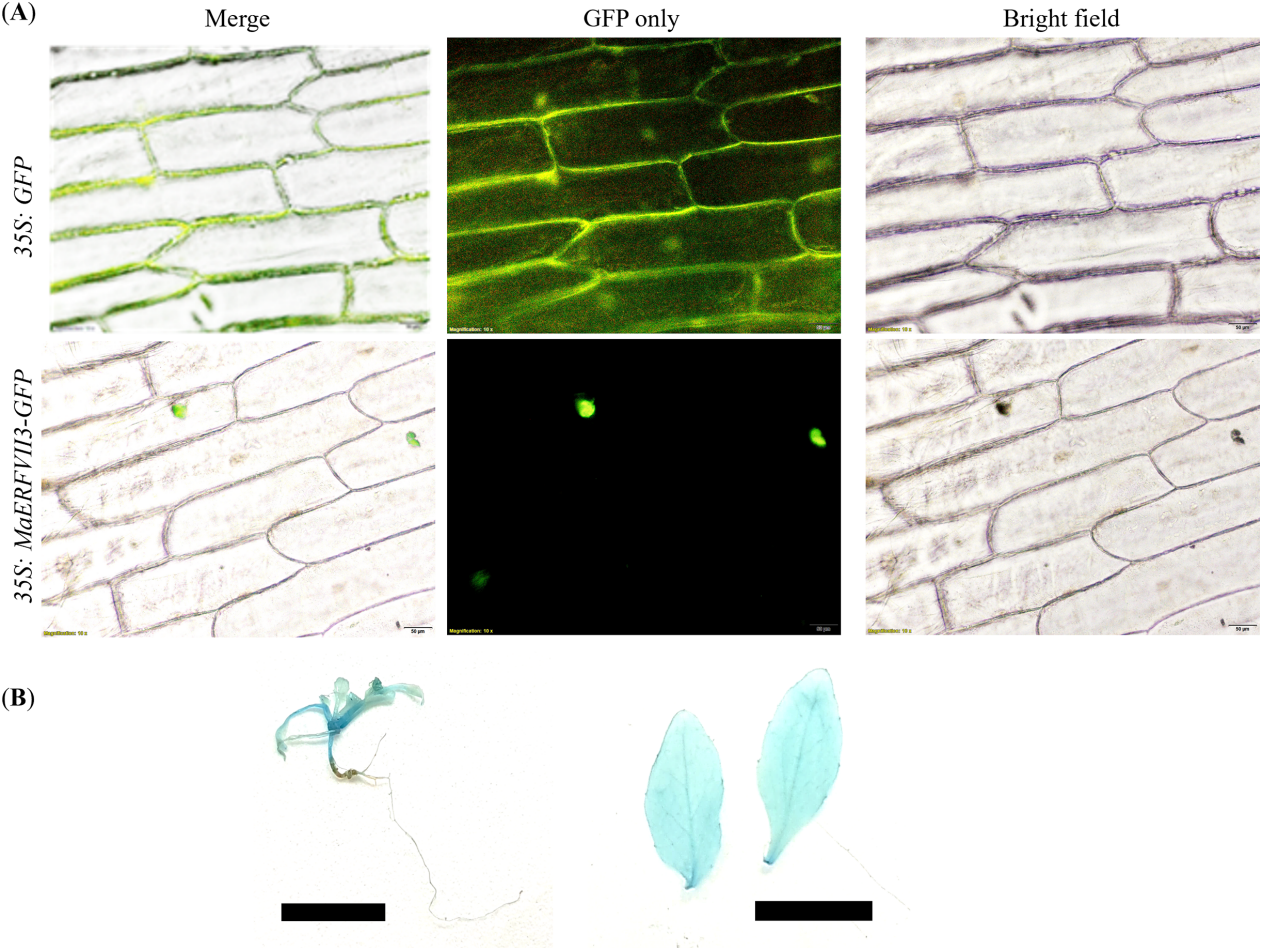
Expression of the MaERFVII-GFP and control GFP vectors in living onion epidermal cells. (A) The localization of MaERFVII3 protein was observed using an inverted fluorescent microscope, with photographs taken in a dark field to visualize green fluorescent (middle) and in bright light to visualize cell morphology (right). The merged image was produced by the software (left). The scale bars represent 50 μm, present at the bottom right of the image. GUS histochemical staining of (B) 2-week-old transgenic *Arabidopsis* seedling (left) and leaves of 6-week-old *Arabidopsis* plantlets (right). The scale bar indicates a length of 1 cm.

GFP was fused to the C-terminal of the *MaERFVII3* coding region and transiently expressed in onion epidermal cells. All transgenic lines showed a GFP signal in the nucleus of onion epidermal cells (Fig. 4A). In contrast, the transformants with the empty vector showed an even distribution of GFP throughout the cells. Similarly, GUS activity of the *MaERFVII*-expressing lines was detected in leaves, shoot apical meristem, petiole, veins, and stems of the 2-week-old transgenic seedlings and in the leaves of 6-week-old transgenic plantlets (Fig. 4B).

### Morphological changes of *MaERFVII3*-expressing lines

**Figure 5:**
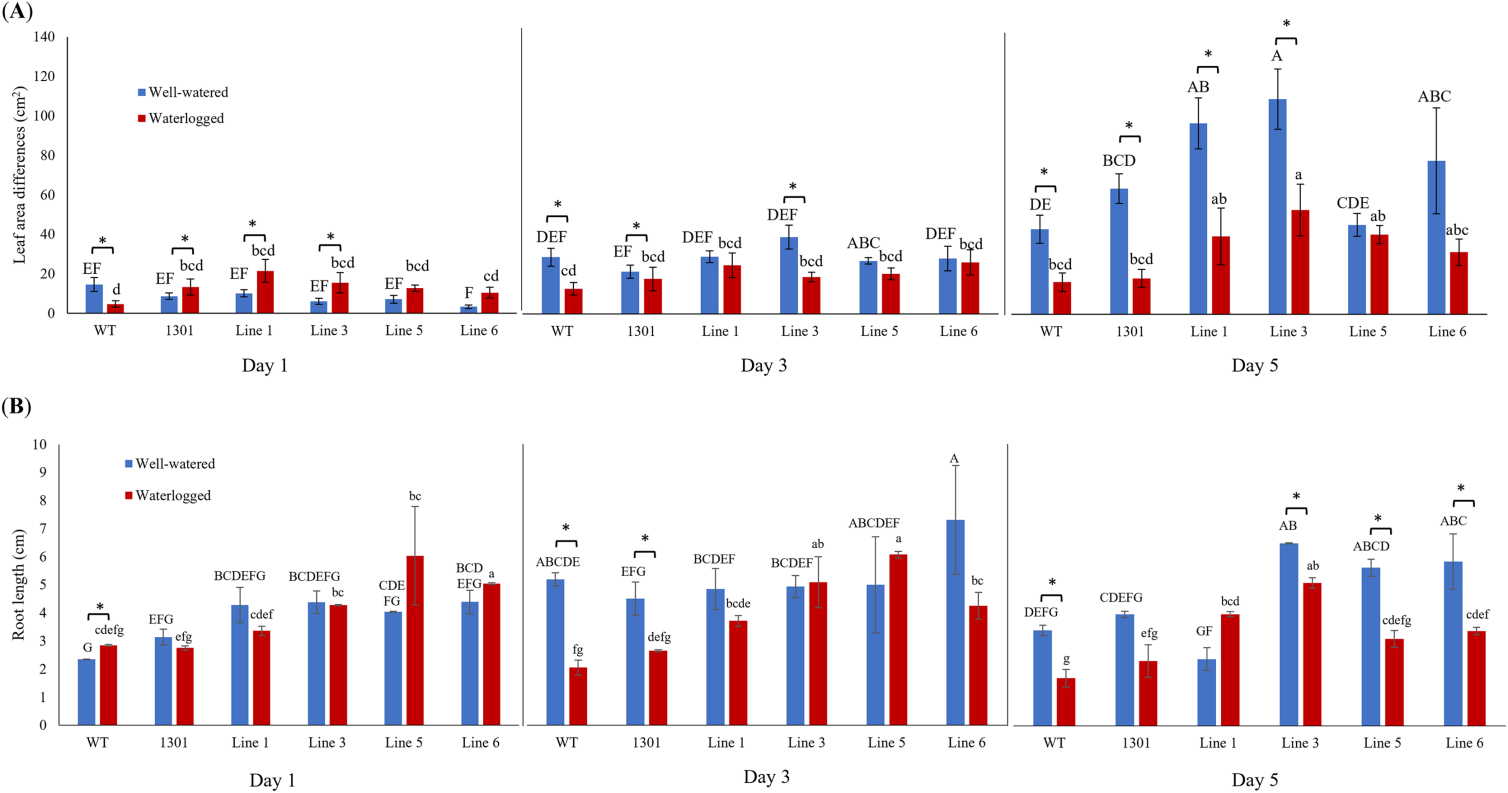
(A) Differences of leaf area in wild-type, pCAMBIA1301, MaERFVII3-pCAMBIA1301-expressing plants (*n* = 8) under well-watered and waterlogged conditions. (B) The root length of different *Arabidopsis* genotypes (*n* = 3) under well-watered and waterlogged conditions. Error bars indicate standard error between biological replicates. Asterisks (*) indicate a significant difference between well-watered and waterlogged samples at *p* < 0.05. Data are presented as means ± standard error from three independent biological replicates. Different capital letters indicate a significant difference between time points within well-watered samples, while different lowercase letters indicate a significant difference within waterlogged samples (*p* < 0.05). Line 1, Line 3, Line 5, and Line 6 are MaERFVII3-Line 1, MaERFVII3-Line 3, MaERFVII3-Line 5, and MaERFVII3-Line 6, respectively.

Wild-type *Arabidopsis*, *Arabidopsis* carrying the empty vector pCAMBIA1301, and four independent transgenic lines (Lines 1, 3, 5, and 6) were examined for their morphological changes when exposed to waterlogging stress (Fig. S6). While all plants showed similar morphology and growth rates during acclimatization (Fig. 3A), the transgenic plants displayed larger leaf area differences than the wild-type and pCAMBIA1301 after 3 and 5 days of waterlogging treatment (Fig. 5A). Nevertheless, all well-watered plants showed higher leaf area differences than their counterparts. The transgenic *Arabidopsis* produced a longer root length than wild-type and pCAMBIA1301 lines under well-watered and waterlogging stress conditions (Fig. 5B).

### Gene expression changes of *MaERFVII3*-expressing lines

**Figure 6:**
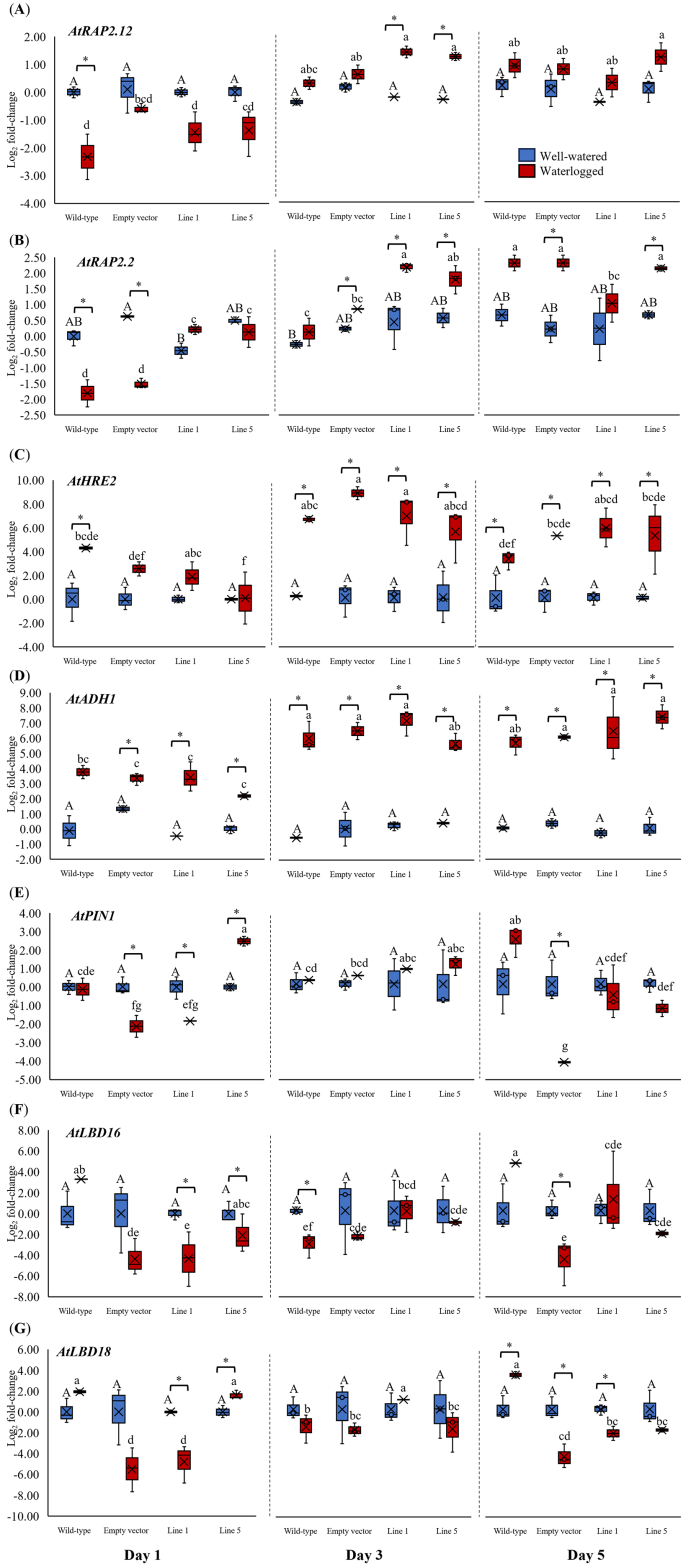
Gene expression of *Arabidopsis* plants for 1, 3, and 5 days of waterlogging. (A) *AtRAP2.12*, (B) *AtRAP2.2*, (C) *AtHRE2*, (D) *AtADH1*, (E) *AtPIN1*, (F) *AtLBD16*, and (G) *AtLBD18*. Error bars indicate standard error between three biological replicates. Asterisks (*) indicate a significant difference between well-watered and waterlogged samples at *p* < 0.05. Different capital letters indicate a significant difference between time points within well-watered samples, while different lowercase letters indicate a significant difference within waterlogged samples (*p* < 0.05). (WT; Wild-type, 1301; pCAMBIA1301, Line 1; *MaERFVII3*-Line 1, and Line 5; *MaERFVII3*-Line 5).

We analyzed the expression of three *Arabidopsis* ERFVII genes (*AtRAP2.12, AtRAP2.2*, and *AtHRE2*), the hypoxia-responsive gene (*AtADH1*), and genes related to adventitious root growth (*AtPIN1, AtLBD16*, and *AtLBD18*) in all samples throughout 1, 3, and 5 days (Fig. 6). *AtPIN1* and *AtLBD16*, were selected based on their known roles as hypoxia-responsive genes (Mustroph et al., 2010), whereas *AtLBD18* was selected for its known involvement in root growth regulation (Lee et al., 2019). MaERFVII3-Line 1 and MaERFVII3-Line 5 were selected for further analysis due to their comparable homozygosity. The *AtRAP2.12*, *AtRAP2.2*, *AtHRE2*, and *AtADH1* transcript levels of all waterlogged samples were generally higher than the well-watered ones. Of these, *AtRAP2.12* and *AtRAP2.2* recorded a significant upregulation in *MaERFVII3*-expressing Lines 1 and 5 after 3 days of waterlogging treatment (Figs. 6A and 6B). However, their expression was gradually reduced after 5 days of waterlogging treatment. Unlike *AtRAP2.12* and *AtRAP2.2*, the expression levels of *AtHRE2* were upregulated after 1 day of waterlogging, except Line 5 (Fig. 6C). The hypoxia-responsive gene, *AtADH1*, showed the highest expression after waterlogging exposure on days 3 and 5 (Fig. 6D).

The expression of auxin transporter PIN1 in wild-type *Arabidopsis* was comparable between well-watered and waterlogged conditions on days 1 and 3 (Fig. 6E). However, after 5 days of waterlogging stress, the waterlogged samples exhibited an upregulation of PIN1. Surprisingly, empty vector pCAMBIA1301 exhibited a comparable expression profile with *MaERFVII3-*Line 1. In contrast, the waterlogged *MaERFVII3-*Line 5 showed a significant upregulation on day 1, followed by a gradual decrease. On the other hand, *AtLBD16* and *AtLBD18* expressions in wild-type *Arabidopsis* were upregulated under waterlogging stress, except on day 3 (Figs. 6F and 6G). However, these genes were generally downregulated in the pCAMBIA1301 and transgenic lines.

## Discussion

### Characterization of MaERFVII3

There are 18 members of the ERFVII family in the banana genome (Lakhwani et al., 2016). However, their roles in response to abiotic stress remain unknown. In this study, we characterized the isolated *MaERFVII3* from banana roots, given its high expression observed in our previous study (Teoh et al., 2022). The MaERFVII3 protein belongs to the group VII ERF since it contains the AP2/ERF domain and the MCGGAI(I/L) conserved N-terminal region. MaERFVII3 was clustered with AtHRE2/ERF71 in Group III, a group predominantly associated with abiotic stress tolerance. For instance, a rice protein with similar motifs with members in Group III, OsERF68, was involved in cold and hypoxia stresses (Bevilacqua et al., 2015; Mohanty, 2022). Five members of the AtERFVII group, specifically AtERF71-75, have been shown to play crucial roles in responding to waterlogging stress and low oxygen conditions (Gibbs et al., 2015).

The subsequent subcellular localization experiment showed that the MaERFVII3 protein localizes in the cell nucleus, suggesting it likely performs the TF function. Similar findings have been observed in other plant species, such as *Salvia miltiorrhiza* and tomato plants (Shi et al., 2021; Zheng et al., 2021). The ERF TF is crucial in signal transduction of plant growth and development as well as the response towards biotic and abiotic stresses. For instance, 67 ERF TFs in bananas were differentially expressed in response to waterlogging stress (Teoh et al., 2022). They belong to groups IIa, IIb, IIIc, IIId, V, VI, VII, VIII, IX, and X. Among the group VII ERF, six out of 18 genes participated in the waterlogging response.

### Morphological and gene expression changes of transgenic *Arabidopsis* lines

The beneficial impacts of ERFVII members on plant tolerance to waterlogging stress have been previously reported (De Ollas et al., 2021; Luo et al., 2022; Yu et al., 2019). Hence, elucidating the function of upregulated *MaERFVII3* under waterlogging is crucial. For this reason, we isolated, cloned, and expressed *MaERFVII3* in *Arabidopsis* plants. After exposure to waterlogging stress, we found that the *MaERFVII3-*expressing lines showed enlarged leaves and longer roots than wild-type and pCAMBIA1301. This observation was similar to the study by Eysholdt-Derzso & Sauter (2019), where expressing *AtHRE2* (HRE group) increased the density of adventitious roots and root elongation. However, this gene could repress hypoxia-induced root bending (Eysholdt-Derzso & Sauter, 2017). In barley, *HvERF2.11* overexpression enhanced the survival rate, fresh weight, and root length of *Arabidopsis* (Luan et al., 2020). ERFVII TFs have been shown to reorganize plant root architecture by developing adventitious roots, repressing the lateral root, and promoting primary root bending (Eysholdt-Derzso & Sauter, 2019; Shukla et al., 2019). This characteristic might be due to the ability of ERFVII TF to inhibit genes related to auxin signaling, resulting in the repression of lateral root primordia (Butsayawarapat et al., 2019). Taken together, our findings suggest that *MaERFVII3* is involved in the growth of primary roots under hypoxia stress.

When analyzing the transcript of *MaERFVII3* and other waterlogging stress-related genes, we found a reduction of *AtRAP2.12* and *AtRAP2.2* transcripts in wild-type and pCAMBIA1301 lines after 1 day of waterlogging. This observation aligns with findings from other studies, where *AtRAP2.12* and *AtRAP2.2* expression levels declined rapidly after the first hour of anoxia stress, reaching near zero at 9 h of anoxia stress (Papdi et al., 2015). We speculate that the decreased levels of *AtRAP2.12* and *AtRAP2.2* in both wild-type and pCAMBIA1301 might be attributed to the negative regulation of *hypoxia response attenuator 1* (HRA1), a downstream target of RAP2.12 (Giuntoli et al., 2017). As shown by Wei et al. (2019), overexpression of *TaERFVII.1* in wheat led to an increase in the expression of the HRA1 homolog, resulting in decreased *TaERFVII.1*-regulated hypoxia-responsive genes, such as *RAP2.12* and *RAP2.2*. In comparison to wild-type and pCAMBIA1301 lines, the transgenic Lines 1 and 5 showed increased *AtRAP2.12* and *AtRAP2.2* expressions, suggesting that *MaERFVII3* might regulate the transcriptional activity of *AtRAP2.12* and *AtRAP2.2* under waterlogging stress.

Under oxygen-deprived conditions, genes related to fermentation enzymes, such as *ADH1* and *PDC1*, are often expressed (Liu et al., 2022; Luo et al., 2022; Xu, Shen & Zhang, 2022). Our results showed that *AtADH1* in wild-type and transgenic *Arabidopsis* was significantly upregulated under waterlogging stress. This observation is uncommon, as previous studies indicated that the expression of ERFVII TFs in *Arabidopsis*, *Actinidia chinesis*, and petunia led to a higher expression level of *ADH1* than their wild-type (Giuntoli et al., 2017; Liu et al., 2022; Ventura et al., 2020; Yin et al., 2019). We speculate that *MaERFVII3* might have a minimal role in carbohydrate conservation, like *AtHRE2*, or require a putative partner to activate waterlogging tolerance (Licausi et al., 2010). Since waterlogging tolerance was enhanced in *MaERFVII3*-expressing plants, it is believed that other mechanisms might be involved, such as reactive oxygen species (ROS) scavenging.

## Conclusions

Waterlogging stress affects plant growth and development. Continuing our previous study, this study focuses on characterizing the isolated MaERFVII3 and determining its role in responding to waterlogging stress in *Arabidopsis* plants. The results showed that MaERFVII3 contains the AP2/ERF domain and the MCGGAI(I/L) conserved N-terminal region and is localized in the cell nucleus. The generated transgenic plants generally exhibited larger leaf areas and longer root lengths than the wild-type and empty vector under waterlogging stress. Several ERFVII and hypoxia genes exhibited upregulation under waterlogging stress conditions, while genes associated with adventitious root growth generally downregulated in the pCAMBIA1301 and transgenic lines. Although additional studies are necessary to validate this observation, the increased tolerance of transgenic lines to waterlogging in this study supports the important role of MaERFVII3 in waterlogging stress regulation. These findings contribute to the development of new banana varieties that could withstand waterlogging stress.

## Supplemental Information

10.7717/peerj.17285/supp-1 Supplemental Information 1 The list of ERFVII genes containing the common name, species, and reported function used in the phylogenetic tree.

10.7717/peerj.17285/supp-2 Supplemental Information 2 Primers used to check for positive transformants.

10.7717/peerj.17285/supp-3 Supplemental Information 3 The conserved motifs in ERF of 95 proteins using MEME-suite.

10.7717/peerj.17285/supp-4 Supplemental Information 4 The conserved motifs based on the differentially expressed ERF genes.

10.7717/peerj.17285/supp-5 Supplemental Information 5 Protein information based on amino acid composition.

10.7717/peerj.17285/supp-6 Supplemental Information 6 Sequence alignment at the AP2/ERF domain across multiple ERFVII amino acid compositions using MegaX software.

10.7717/peerj.17285/supp-7 Supplemental Information 7 The exon-intron structure analysis of ERFVII. The exon-intron structure of ERFVII was analyzed by comparing gene sequences from five plant species: *Arabidopsis thaliana*, tomato (*Solanum lycopersicum*), soybean (*Glycine max*), banana (*Musa acuminata*), and rice (*Oryza sativa*), using Gene Structure Display Server (GSDS). Each ERFVII gene contains either zero or one intron (black line) within its coding sequence (yellow bar). The genes are flanked by 5’ upstream and 3’ downstream regulatory regions, represented by the blue blocks. The scale bar at the bottom indicates the length of each gene.

10.7717/peerj.17285/supp-8 Supplemental Information 8 Amplification and sequence alignment of the MaERFVII3-pGEMT-Easy and MaERFVII3-pCAMBIA1301. (A) Amplicons of MaERFVII3-pGEMT Easy amplified using M13 primers. M: 1 kb ladder, −ve: Negative control, Lanes 1, 2, 3: Amplicons of extracted plasmid from three independent colonies. (B) Plasmid excised with restriction enzyme NcoI to release the insert with the expected size of 693 bp. M: 1 kb ladder. Lanes 1, 2, and 3: The linearized plasmid with insert from three independent colonies. (C) The sequencing alignment between three independent colonies containing MaERFVII3-pGEMT-EASY. (D) The alignment between MaERFVII3-pGEMT-Easy and MaERFVII3-pCAMBIA1301. Asterisks (*) indicate a similar sequence between the three colonies. The green bar highlights the 100% similar identity between the sequences.

10.7717/peerj.17285/supp-9 Supplemental Information 9 The extracted DNA and verification of the MaERFVII3 transgene in T2 transgenic Arabidopsis plantlets. (A) The extracted DNA of the T2 transgenic Arabidopsis plantlets. Verification of the MaERFVII3 transgene in T2 transgenic Arabidopsis plantlets using (B) MaERFVII3 gene-specific primers and (C) hygromycin primers. Lane 1: Line 1 plant 1, Lane 2: Line 1 plant 2, Lane 3: Line 3 plant 1, Lane 4: Line 5 plant 1, Lane 5: Line 5 plant 2. M indicates a 1 kb DNA marker ladder with the indicated band size, and –ve and +ve represent the negative and positive controls, respectively.

10.7717/peerj.17285/supp-10 Supplemental Information 10 Different T3 Arabidopsis plantlets grown under well-watered and waterlogged conditions for 1, 3, and 5 days. T3 Arabidopsis plantlets (A) Wild-type, (B) pCAMBIA1301, (C) MaERFVII3-Line 1, (D) MaERFVII3-Line 3, (E) MaERFVII3-Line 5, and (F) MaERFVII3-Line 6, grown under well-watered and waterlogged conditions for 1, 3, and 5 days.

10.7717/peerj.17285/supp-11 Supplemental Information 11 Primer efficiency and melting curve. The primer efficiency graph (left) with its corresponding melting curve (right) of each primer pair was used in gene expression analysis. (A) AtTUB, (B) AtUBQ2, (C) AtRAP2.12, (D) AtRAP2.2, (E) AtADH1, (F) MaERFVII3, (G) AtPIN1, (H) AtLBD16, and (I) AtLBD18.

10.7717/peerj.17285/supp-12 Supplemental Information 12 Amplification plot for gene expression analysis. The amplification plot of a SYBR green gene expression graph. Grey samples represent NTC, while the remaining color represents the qPCR reaction mix containing a cDNA template.

10.7717/peerj.17285/supp-13 Supplemental Information 13 Detection of the presence of MaERFVII3 in each T3 genotype sample. Full gel images for Figure 3. Detection of the presence of MaERFVII3 in each T3 genotype sample of (B) Day 1, (C) Day 3, and (D) Day 5 samples. ‘+’ indicates positive control, while ‘−ve’ indicates negative control. M represents the ladder for 1 kb ladder. (WT: Wild-type, 1301: pCAMBIA 1301 control, L1: MaERFVII3-Line 1, L3: MaERFVII3-Line 3, L5: MaERFVII3-Line 5)

10.7717/peerj.17285/supp-14 Supplemental Information 14 Differences of leaf area and root length in Arabidopsis under well-watered and waterlogged conditions. Differences of leaf area and root length in wild-type, pCAMBIA1301, MaERFVII3-pCAMBIA1301-expressing Arabidopsis plants under well-watered and waterlogged conditions (*n* = 8).

10.7717/peerj.17285/supp-15 Supplemental Information 15 Gene expression of Arabidopsis plants for 1, 3, and 5 days of waterlogging. Gene expression of Arabidopsis plants for 1, 3, and 5 days of waterlogging. (A) *AtRAP2.12*, (B) *AtRAP2.2*, (C) *AtHRE2*, (D) *AtADH1*, (E) *AtPIN1*, (F) *AtLBD16*, and (G) *AtLBD18*. Error bars indicate standard error between three biological replicates. Asterisks (*) indicate a significant difference between well-watered and waterlogged samples at *p* < 0.05. Different capital letters indicate a significant difference between time points within well-watered samples, while different lowercase letters indicate a significant difference within waterlogged samples (*p* < 0.05). (WT: Wild-type, 1301: pCAMBIA1301, Line 1: *MaERFVII3*-Line 1, and Line 5: *MaERFVII3*-Line 5)

10.7717/peerj.17285/supp-16 Supplemental Information 16 *I. silico* specificity analysis of each primer.

10.7717/peerj.17285/supp-17 Supplemental Information 17 MaERFVII3 sequence (accession number: PP083633).

10.7717/peerj.17285/supp-18 Supplemental Information 18 MIQE Checklist.

10.7717/peerj.17285/supp-19 Supplemental Information 19 Multiple sequence alignment of group VII ERFs. Multiple sequence alignment of group VII ERFs illustrates the conservation of the DNA-binding domain (AP2/ERF domain) (Blue color box).

## Additional Information and Declarations

### DNA Deposition

The following information was supplied regarding the deposition of DNA sequences: The MaERFVII3 cDNA sequence is available at GenBank: PP083633.

### Data Availability

The following information was supplied regarding data availability: The raw data are available in the Supplemental Files.
