# Supplementary material for: Expressing banana transcription factor MaERFVII3 in Arabidopsis confers enhanced waterlogging tolerance and root growth
Source: PeerJ. 2024 Apr 30;12:e17285. doi: 10.7717/peerj.17285 (PMC11067909; doi:10.7717/peerj.17285)
Supplement: Supplemental Information 1 [file peerj-12-17285-s001.docx]

**Supplementary Table 1**. The list of ERFVII genes containing the common name, species, and reported function used in the phylogenetic tree.

| **No** | **Gene Accession** | **Common name** | **Species** | **Function** | **Reference** |
| --- | --- | --- | --- | --- | --- |
| 1 | AT1G72360 | ERF73 | Arabidopsis thaliana | Hypoxia stress | Hartman et al. (2019)  Wany et al. (2019) |
| 2 | AT1G53910 | RAP2.12 | Arabidopsis thaliana | Hypoxia stress | White et al. (2018)  Hartman et al. (2019) |
| 3 | AT2G47520 | ERF71 | Arabidopsis thaliana | Hypoxia stress | Hartman et al. (2019) |
| 4 | AT3G14230 | RAP2.2 | Arabidopsis thaliana | Hypoxia stress | White et al. (2018)  Hartman et al. (2019) |
| 5 | AT3G16770 | EBP/RAP2.3 | Arabidopsis thaliana | Hypoxia stress | Hartman et al. (2019) |
| 6 | Os09g11460 | OsERF073/ SUB1C | Oryza sativa | Hypoxia stress | Niroula et al. (2012) |
| 7 | Os07g47790 | OsERF066 | Oryza sativa | Hypoxia stress | Lin et al. (2019) |
| 8 | Os01g21120 | OsERF067 | Oryza sativa | Hypoxia stress | Lin et al. (2019) |
| 9 | DQ011598 | SUB1A | Oryza sativa | Submergence tolerance | Niroula et al. (2012)  Lin et al. (2019)  Singh & Sinha (2016) |
| 10 | Os01g21120 | OsERF068 | Oryza sativa | Hypoxia stress | Fukushima et al. (2016) |
| 11 | Os10g25170 | OsERF059 | Oryza sativa | Unknown | None |
| 12 | Os03g08460 | OsERF060 | Oryza sativa | Response to auxin and stress |  |
| 13 | Os05g29810 | OsERF061 | Oryza sativa | Unknown | None |
| 14 | Os03g08470 | OsERF062 | Oryza sativa | Response to gibberellin | Zhao et al. (2019) |
| 15 | Os09g11480 | OsERF063/ SUB1B | Oryza sativa | Submergence stress, gibberellin response | Fukao et al. (2011)  Fukao & Bailey-Serres (2008)  Xu et al. (2006) |
| 16 | Os03g08500 | OsERF064 | Oryza sativa | Unknown | None |
| 17 | Os07g42510 | OsERF065 | Oryza sativa | Anther development | Jin et al. (2020) |
| 18 | Os03g08490 | OsERF069 | Oryza sativa | Unknown | None |
| 19 | Os02g54160 | OsERF070/EREB1 | Oryza sativa | Defense response, drought responsive gene, response to herbicide | Serra et al. (2013)  Shi et al. (2017)  Wang et al. (2017) |
| 20 | Os06g09390 | OsERF071 | Oryza sativa | Drought responsive gene, JA responsive gene, cell wall modification, root development, defense responsive gene | Zhan et al. (2017) |
| 21 | Os09g26420 | OsERF072 | Oryza sativa | Fungus infection responsive | Lin et al. (2007)  Campos-Soriano et al. (2012) |
| 22 | AEQ58795.1 | RuAceERF1 | Rumex acetosa | Unknown | van Veen et al. (2014) |
| 23 | AEQ58796.1 | ERF2 | Rumex acetosa | Unknown | van Veen et al. (2014) |
| 24 | AEQ58797.1 | ERF1 | Rumex palustris | Unknown | van Veen et al. (2014) |
| 25 | AEQ58798.1 | ERF2 | Rumex palustris | Unknown | van Veen et al. (2014) |
| 26 | AEQ58799.1 | ERF3 | Rumex palustris | Unknown | van Veen et al. (2014) |
| 27 | XP_004252402.1 | RAP2.12 | Solanum lycopersicon | Unknown | None |
| 28 | NP_001234513.1 | JERF1 | Solanum lycopersicon | Not affected by Hypoxia | Safavi-Rizi et al. (2020) |
| 29 | AAO34704.1 | ERF2 | Solanum lycopersicon | Hypoxia responsive | Safavi-Rizi et al. (2020) |
| 30 | NP_001266125.1 | ERF | Solanum lycopersicon | Unknown | None |
| 31 | AIW04142.1 | RAP2.2 | Solanum lycopersicon | Unknown | None |
| 32 | NP_001234308.2 | ERF2 | Solanum lycopersicon | Unknown | None |
| 33 | XP_024452250.1 | RAP2.12/ERFB2-1 | Populus trichocarpa | Unknown | None |
| 34 | XP_006369093.1 | RAP2.12 | Populus trichocarpa | Unknown | None |
| 35 | XP_006380240.1 | RAP2.3 | Populus trichocarpa | Unknown | None |
| 36 | XP_002315490.2 | RAP2.3 isoform X1 | Populus trichocarpa | Unknown | None |
| 37 | XP_002301490.2 | ERF071 | Populus trichocarpa | Unknown | None |
| 38 | XP_002320996.1 | ERF071 | Populus trichocarpa | Unknown | None |
| 39 | NP_001241423.1 | ERFVII1 | Glycine max | Responds to ethylene, increases with submergence stress | Tamang et al. (2014) |
| 40 | NP_001238300.2 | ERFVII2 | Glycine max | Insensitive to ethylene | Tamang et al. (2014) |
| 41 | NP_001341213.1 | ERFVII3 | Glycine max | Responds to ethylene, increases with submergence stress | Tamang et al. (2014) |
| 42 | NP_001243393.1 | ERFVII4 | Glycine max | Responds to ethylene, increases with submergence stress | Tamang et al. (2014) |
| 43 | NP_001241446.1 | ERFVII5 | Glycine max | Responds to ethylene, increases with submergence stress | Tamang et al. (2014) |
| 44 | XP_003546368.1 | ERFVII6 | Glycine max | Responds to ethylene, increases with submergence stress | Tamang et al. (2014) |
| 45 | NP_001335962.1 | ERFVII7 | Glycine max | Responds to ethylene, increases with submergence stress | Tamang et al. (2014) |
| 46 | NP_001235127.1 | ERFVII8 | Glycine max | Responds to ethylene, increases with submergence stress | Tamang et al. (2014) |
| 47 | XP_006604931.1 | ERFVII9 | Glycine max | Insensitive to ethylene | Tamang et al. (2014) |
| 48 | NP_001336749.1 | ZmERFVII1 | Zea mays | Unknown | None |
| 49 | ONM25589.1 | ZmERFVII2 | Zea mays | Unknown | None |
| 50 | XP_023157587.1 | ZmERFVII3 | Zea mays | Hypoxia responsive | Du et al. (2014) |
| 51 | NP_001105270.2 | ZmERFVII4 | Zea mays | Hypoxia responsive | Du et al. (2014) |
| 52 | NP_001152666.1 | ZmERFVII5 | Zea mays | Hypoxia responsive | Du et al. (2014) |
| 53 | XP_008647763.1 | ZmERFVII6 | Zea mays | Unknown | None |
| 54 | NP_001149434.1 | ZmERFVII7 | Zea mays | Unknown | None |
| 55 | XP_008658488.1 | ZmERFVII8 | Zea mays | Hypoxia responsive | Du et al. (2014) |
| 56 | NP_001150246.1 | ZmERFVII9 | Zea mays | Unknown | None |
| 57 | NP_001150673.2 | ZmERFVII10 | Zea mays | Hypoxia responsive | Du et al. (2014) |
| 58 | NP_001148691.1 | ZmERFVII11 | Zea mays | Unknown | None |
| 59 | ONM56278.1 | ZmERFVII12 | Zea mays | Hypoxia responsive | Du et al. (2014) |
| 60 | NP_001136511.1 | ZmERFVII13 | Zea mays | Hypoxia responsive | Du et al. (2014) |
| 61 | NP_001144222.2 | ZmERFVII14 | Zea mays | Unknown | None |
| 62 | NP_001147181.2 | ZmERFVII15 | Zea mays | Unknown | None |
| 63 | NP_001241699.1 | ZmERFVII16 | Zea mays | Unknown | None |
| 64 | NP_001149730.2 | ZmERFVII17 | Zea mays | Unknown | None |
| 65 | XP_008390617.1 | MDP0000128979 | Malus domestica | Not really responsive to hypoxia | Cukrov et al. (2016) |
| 66 | XP_008340186.1 | MDP0000308922 | Malus domestica | Not really responsive to hypoxia | Cukrov et al. (2016) |
| 67 | XP_028951794.1 | MDP0000413387 | Malus domestica | Unknown | Cukrov et al. (2016) |
| 68 | NP_001306945.1 | MDP0000288465 | Malus domestica | Hypoxia responsive | Cukrov et al. (2016) |
| 69 | NP_001315685.1 | MDP0000848905 | Malus domestica | Hypoxia responsive | Cukrov et al. (2016) |
| 70 | NP_001280975.1 | MDP0000403580 | Malus domestica | Absent in hypoxia | Cukrov et al. (2016) |
| 72 | AGC79344.1 | DkERF10 | Diospyros kaki | Hypoxia responsive | Meitha et al. (2018) |
| 73 | VIT_07s0005g00820 | VvERF057 | Vitis vinifera | Unknown | None |
| 74 | VIT_05s077g01860 | VvERF058 | Vitis vinifera | Unknown | None |
| 75 | VIT_09s0002g00470 | VvERF059 | Vitis vinifera | Unknown | None |
