# Supplementary material for: Expressing banana transcription factor MaERFVII3 in Arabidopsis confers enhanced waterlogging tolerance and root growth
Source: PeerJ. 2024 Apr 30;12:e17285. doi: 10.7717/peerj.17285 (PMC11067909; doi:10.7717/peerj.17285)
Supplement: Supplemental Information 2 [file peerj-12-17285-s002.docx]

**Supplementary Table 2**. Primers used to check for positive transformants.

| **Primer name** | **Gene ID** | **Forward primer sequence (5’-3’)** | **Reverse primer sequence (5’-3’)** | **Product length (bp)** |
| --- | --- | --- | --- | --- |
| MaERFVII3 | Macma4_02_g02290 | ATGTGTGGCGGC GCCATCAT | CCATGGCGCCGGCAGAATGTCGTC GAA | 694 |
| Hygromycin | AF234297 | ATGCGGAGCATATACGCCCGG | TTATCGGCACTTTGCATCGGC | 618 |
