# Supplementary material for: Expressing banana transcription factor MaERFVII3 in Arabidopsis confers enhanced waterlogging tolerance and root growth
Source: PeerJ. 2024 Apr 30;12:e17285. doi: 10.7717/peerj.17285 (PMC11067909; doi:10.7717/peerj.17285)
Supplement: Supplemental Information 3 [file peerj-12-17285-s003.docx]

**Supplementary Table 3**. The conserved motifs in ERF of 95 proteins using MEME-suite.

|  | **Name** | **Sites** | **Width** | **E-value** |
| --- | --- | --- | --- | --- |
| 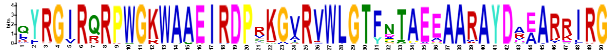 | CM-1 | 94 | 50 | 9.2e-4305 |
| 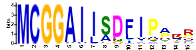 | CM-2 | 85 | 15 | 4.5e-827 |
| 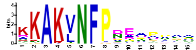 | CM-3 | 95 | 21 | 1.4e-528 |
| 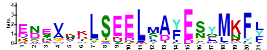 | CM-4 | 48 | 15 | 6.3e-389 |
| 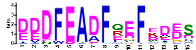 | CM-5 | 49 | 11 | 6.3e-296 |
| 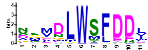 | CM-6 | 72 | 11 | 1.3e-289 |
| 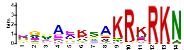 | CM-7 | 76 | 24 | 6.8e-257 |
