# Supplementary material for: Expressing banana transcription factor MaERFVII3 in Arabidopsis confers enhanced waterlogging tolerance and root growth
Source: PeerJ. 2024 Apr 30;12:e17285. doi: 10.7717/peerj.17285 (PMC11067909; doi:10.7717/peerj.17285)
Supplement: Supplemental Information 4 [file peerj-12-17285-s004.docx]

**Supplementary Table 4**. The conserved motifs based on the differentially expressed ERF genes.

| Group IIa motifs | | E-value |
| --- | --- | --- |
| CM-1 | 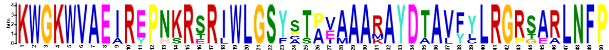 | 1.6e-143 |
| CM-2 | 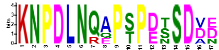 | 6.2e-027 |
| CM-3 | 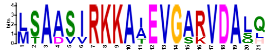 | 2.2e-025 |
| CM-4 | 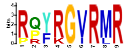 | 1.2e-009 |
| Group IIb motifs | | E-value |
| CM-1 | 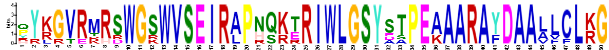 | 2.4e-181 |
| CM-2 | 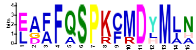 | 5.7e-022 |
| CM-3 | 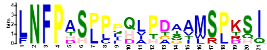 | 7.2e-019 |
| CM-4 | 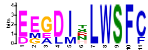 | 1.0e-013 |
| Group IIIc motifs | | E-value |
| CM-1 | 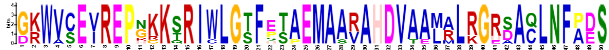 | 4.6e-207 |
| CM-2 | 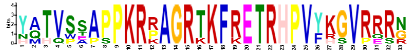 | 8.7e-080 |
| CM-3 | 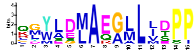 | 1.4e-024 |
| CM-4 | 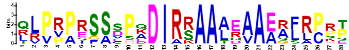 | 4.3e-032 |
| Group IIId motifs | | E-value |
| CM-1 | 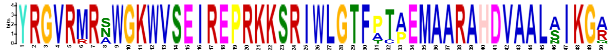 | 3.5e-274 |
| CM-2 | 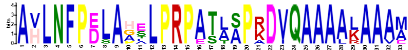 | 9.3e-089 |
| CM-3 | 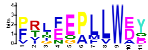 | 1.3e-011 |
| CM-4 | 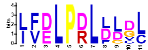 | 1.8e-013 |
| Group V motifs | | E-value |
| CM-1 | 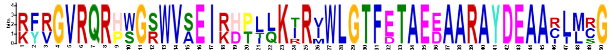 | 2.1e-105 |
| CM-2 | 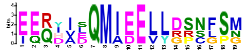 | 4.6e-007 |
| CM-3 | 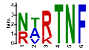 | 7.3e-007 |
| CM-4 | 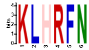 | 4.5e-001 |
| Group VI motifs | | E-value |
| CM-1 | 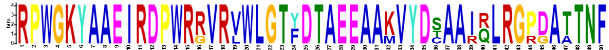 | 2.9e-073 |
| CM-2 | 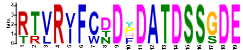 | 1.6e-013 |
| CM-3 | 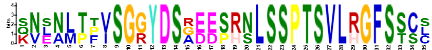 | 4.3e-011 |
| CM-4 | 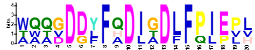 | 6.1e-010 |
| Group VII | | E-value |
| CM-1 | 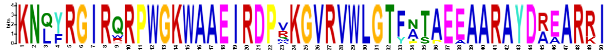 | 1.1e-335 |
| CM-2 | 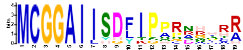 | 5.3e-074 |
| CM-3 | 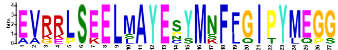 | 1.1e-065 |
| CM-4 | 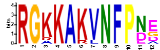 | 3.1e-053 |
| Group VIII motifs | | E-value |
| CM-1 | 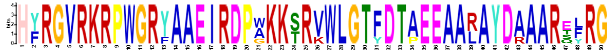 | 9.1e-101 |
| CM-2 | 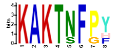 | 1.2e-011 |
| CM-3 | 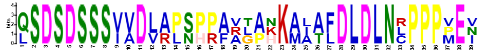 | 2.5e-011 |
| CM-4 | 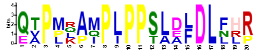 | 3.5e-006 |
| Group IXa motifs | | E-value |
| CM-1 | 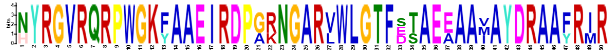 | 8.5e-033 |
| CM-2 | 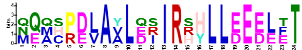 | 5.7e-005 |
| CM-3 | 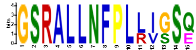 | 4.3e-003 |
| CM-4 | 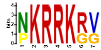 | 3.8e+000 |
| Group IXb & c motifs | | E-value |
| CM-1 | 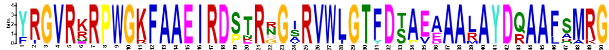 | 4.2e-296 |
| CM-2 | 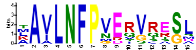 | 1.1e-042 |
| CM-3 | 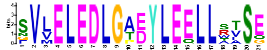 | 3.4e-042 |
| CM-4 | 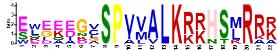 | 2.3e-027 |
| Group X motifs | | E-value |
| CM-1 | 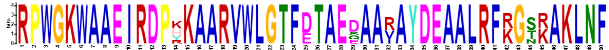 | 1.4e-163 |
| CM-2 | 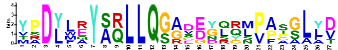 | 1.5e-018 |
| CM-3 | 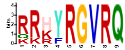 | 5.4e-014 |
| CM-4 | 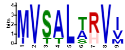 | 4.0e-011 |
