# Supplementary figures and images for: Expressing banana transcription factor MaERFVII3 in Arabidopsis confers enhanced waterlogging tolerance and root growth
Source: PeerJ. 2024 Apr 30;12:e17285. doi: 10.7717/peerj.17285 (PMC11067909; doi:10.7717/peerj.17285)

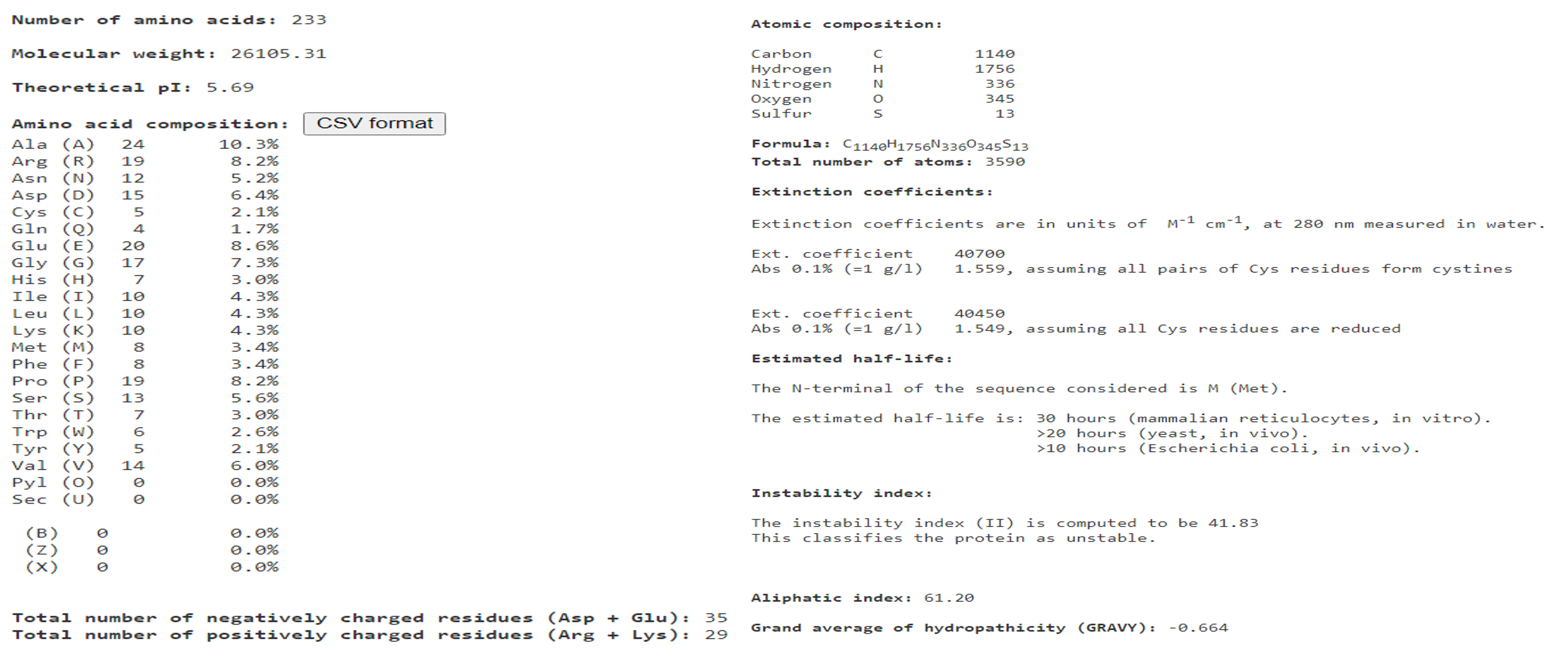

Supplement: Supplemental Information 5 [file peerj-12-17285-s005.png]

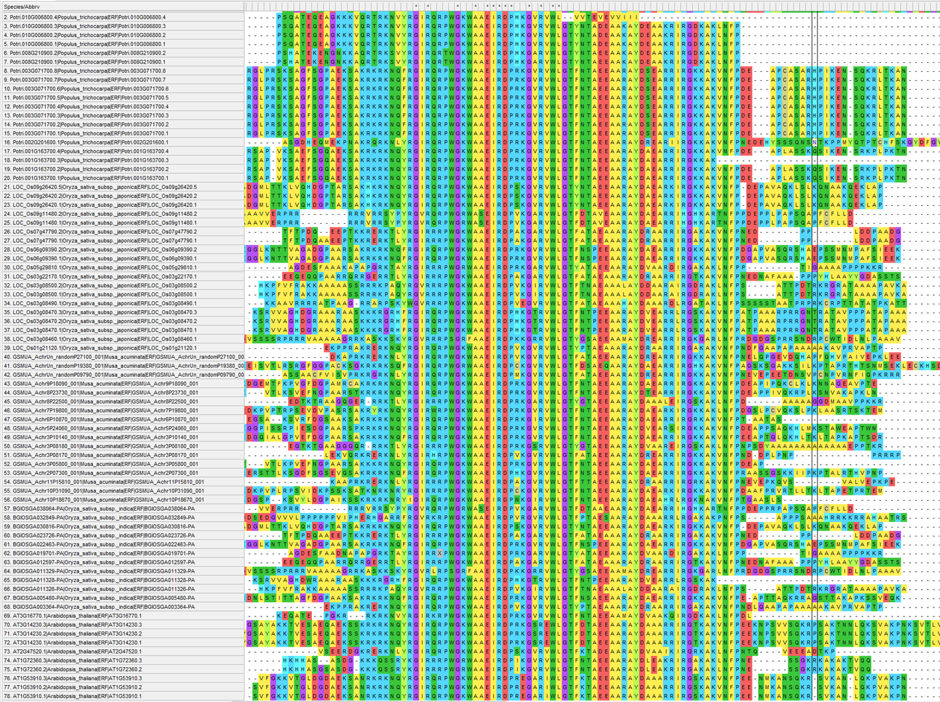

Supplement: Supplemental Information 6 [file peerj-12-17285-s006.png]

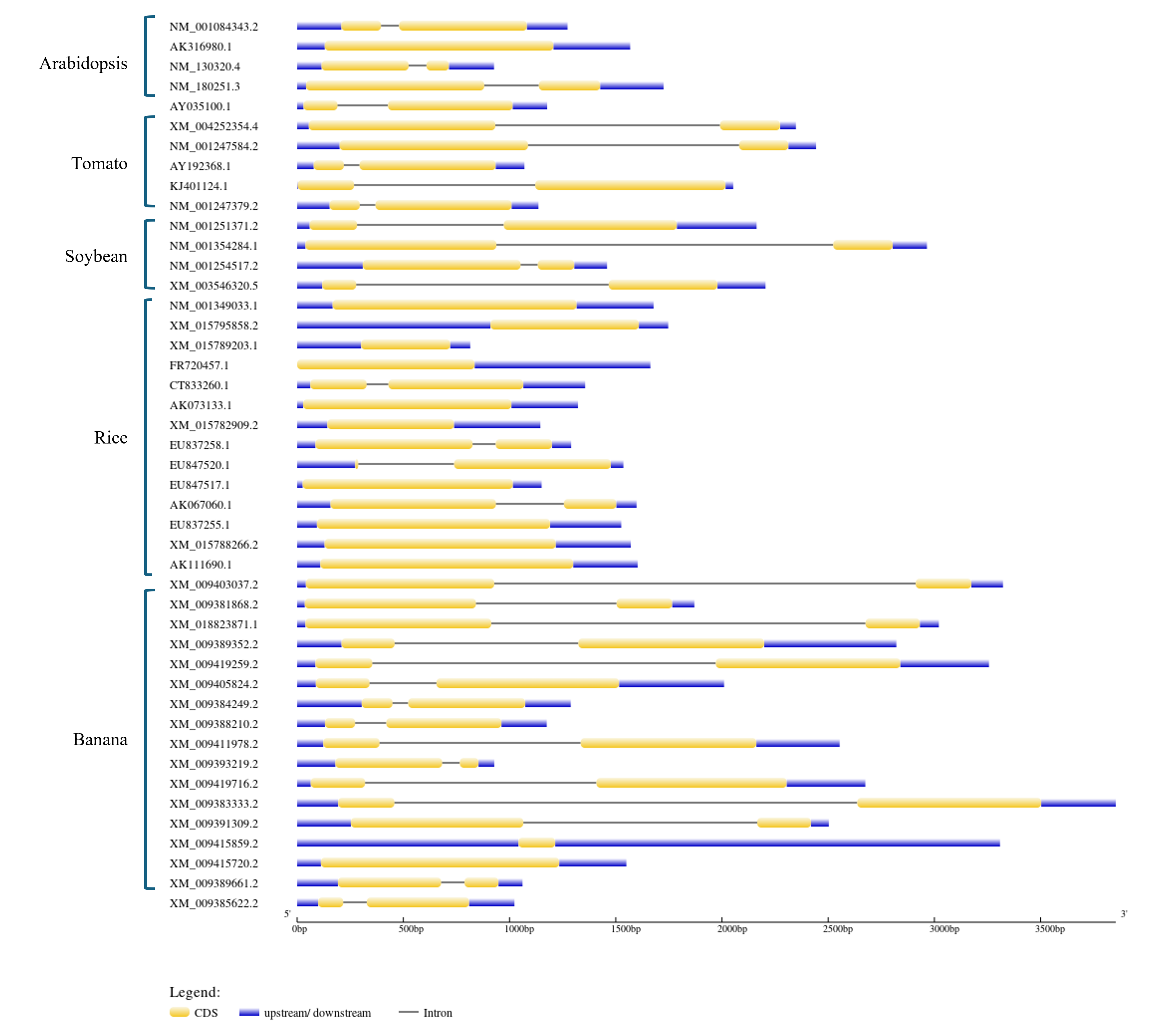

Supplement: Supplemental Information 7 — The exon-intron structure of ERFVII was analyzed by comparing gene sequences from five plant species: Arabidopsis thaliana, tomato (Solanum lycopersicum), soybean (Glycine max), banana (Musa acuminata), and rice (Oryza sativa), using Gene Structure Display Server (GSDS). Each ERFVII gene contains either zero or one intron (black line) within its coding sequence (yellow bar). The genes are flanked by 5’ upstream and 3’ downstream regulatory regions, represented by the blue blocks. The scale bar at the bottom indicates the length of each gene. [file peerj-12-17285-s007.png]

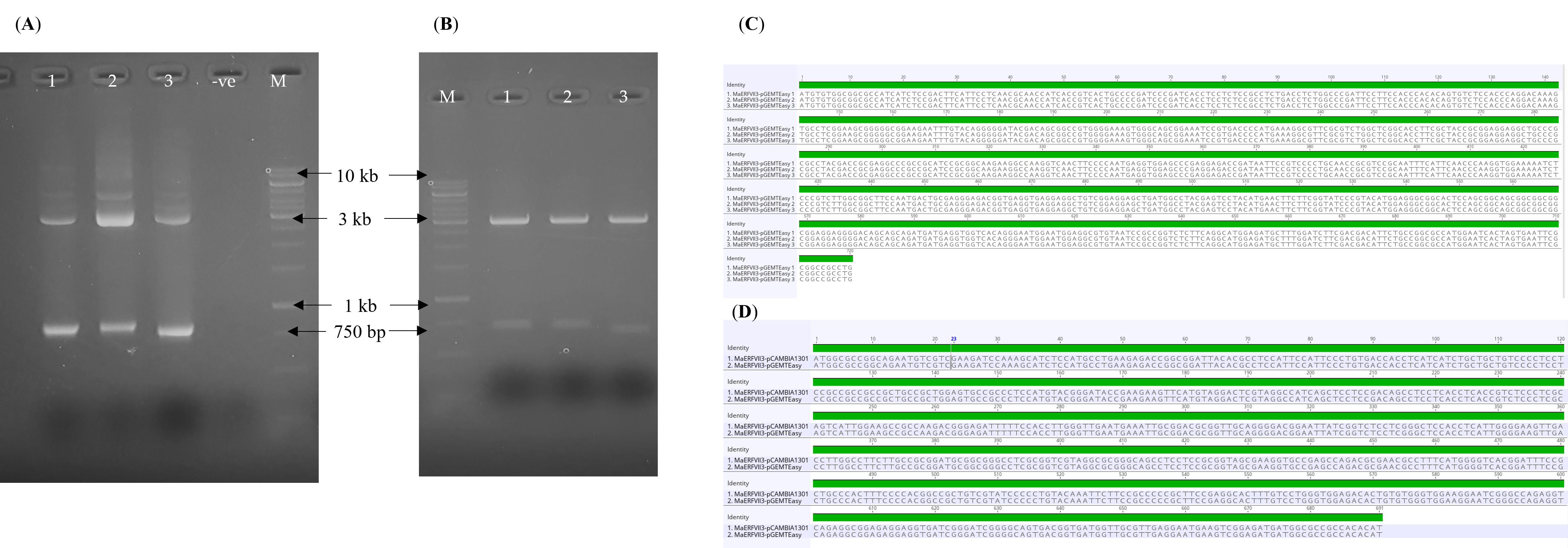

Supplement: Supplemental Information 8 — (A) Amplicons of MaERFVII3-pGEMT Easy amplified using M13 primers. M: 1 kb ladder, −ve: Negative control, Lanes 1, 2, 3: Amplicons of extracted plasmid from three independent colonies. (B) Plasmid excised with restriction enzyme NcoI to release the insert with the expected size of 693 bp. M: 1 kb ladder. Lanes 1, 2, and 3: The linearized plasmid with insert from three independent colonies. (C) The sequencing alignment between three independent colonies containing MaERFVII3-pGEMT-EASY. (D) The alignment between MaERFVII3-pGEMT-Easy and MaERFVII3-pCAMBIA1301. Asterisks (*) indicate a similar sequence between the three colonies. The green bar highlights the 100% similar identity between the sequences. [file peerj-12-17285-s008.png]

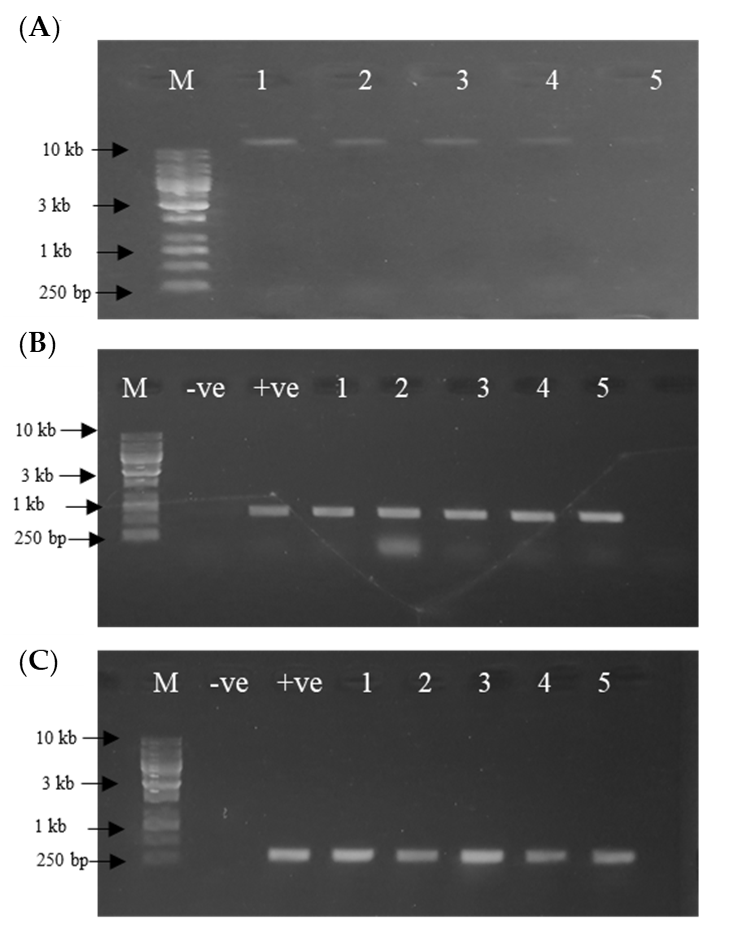

Supplement: Supplemental Information 9 — (A) The extracted DNA of the T2 transgenic Arabidopsis plantlets. Verification of the MaERFVII3 transgene in T2 transgenic Arabidopsis plantlets using (B) MaERFVII3 gene-specific primers and (C) hygromycin primers. Lane 1: Line 1 plant 1, Lane 2: Line 1 plant 2, Lane 3: Line 3 plant 1, Lane 4: Line 5 plant 1, Lane 5: Line 5 plant 2. M indicates a 1 kb DNA marker ladder with the indicated band size, and –ve and +ve represent the negative and positive controls, respectively. [file peerj-12-17285-s009.png]

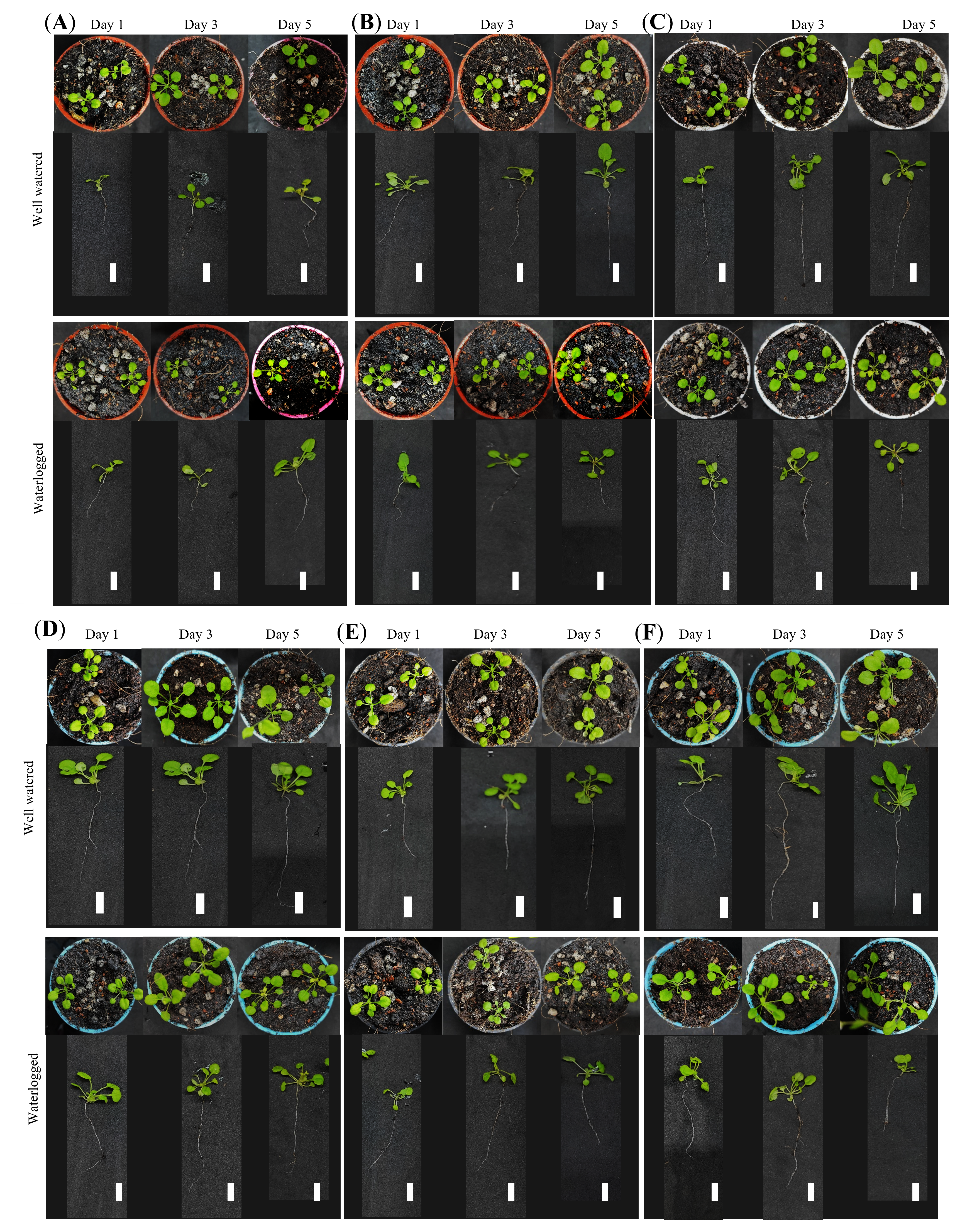

Supplement: Supplemental Information 10 — T3 Arabidopsis plantlets (A) Wild-type, (B) pCAMBIA1301, (C) MaERFVII3-Line 1, (D) MaERFVII3-Line 3, (E) MaERFVII3-Line 5, and (F) MaERFVII3-Line 6, grown under well-watered and waterlogged conditions for 1, 3, and 5 days. [file peerj-12-17285-s010.png]

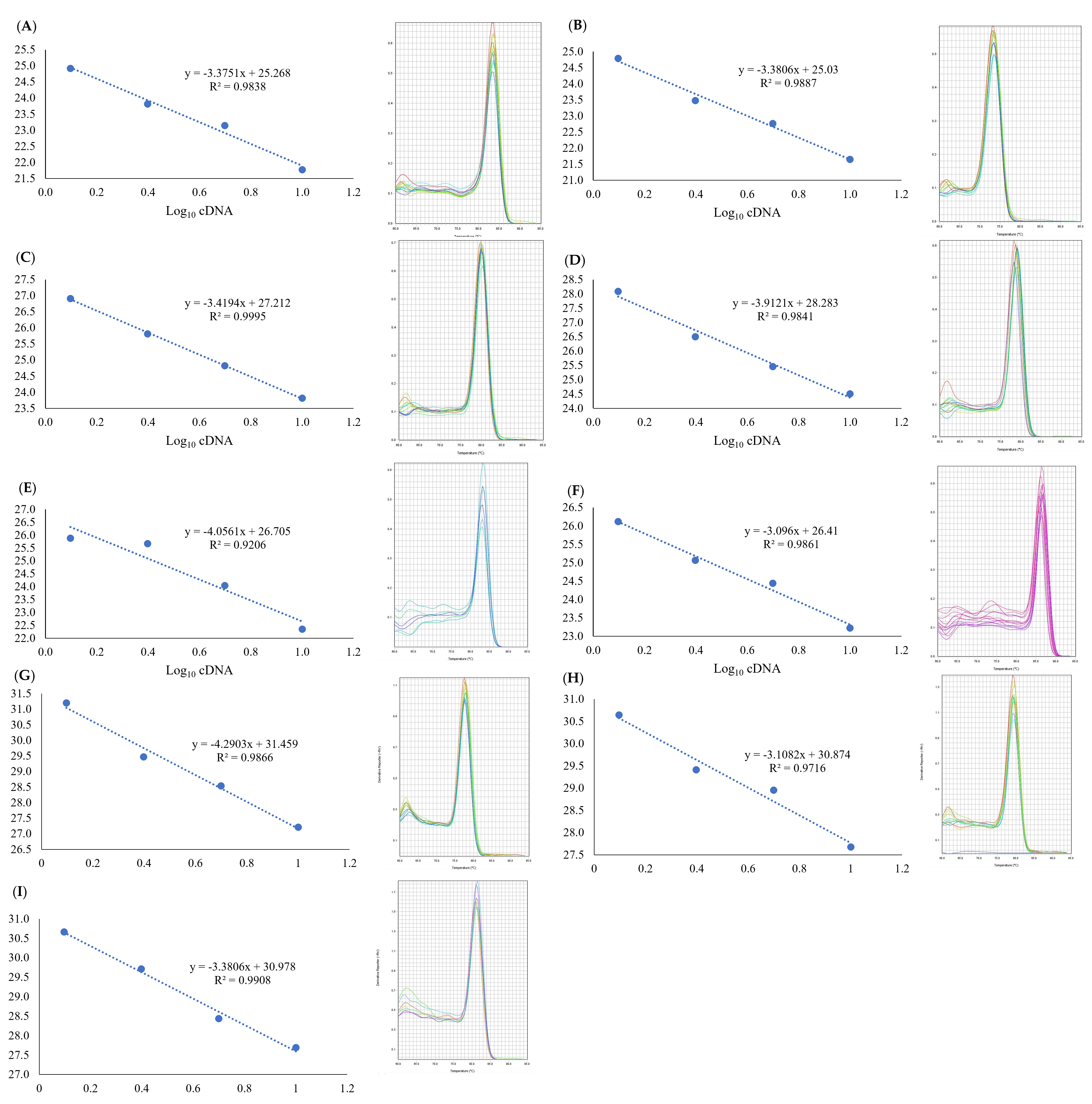

Supplement: Supplemental Information 11 — The primer efficiency graph (left) with its corresponding melting curve (right) of each primer pair was used in gene expression analysis. (A) AtTUB, (B) AtUBQ2, (C) AtRAP2.12, (D) AtRAP2.2, (E) AtADH1, (F) MaERFVII3, (G) AtPIN1, (H) AtLBD16, and (I) AtLBD18. [file peerj-12-17285-s011.png]

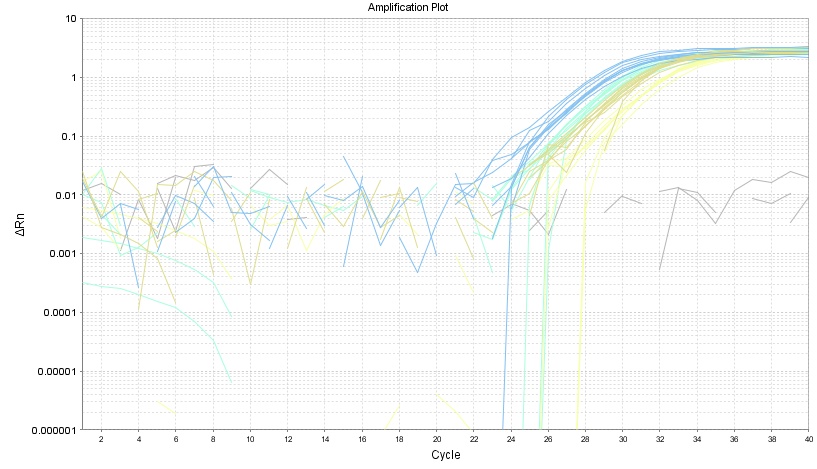

Supplement: Supplemental Information 12 — The amplification plot of a SYBR green gene expression graph. Grey samples represent NTC, while the remaining color represents the qPCR reaction mix containing a cDNA template. [file peerj-12-17285-s012.jpg]

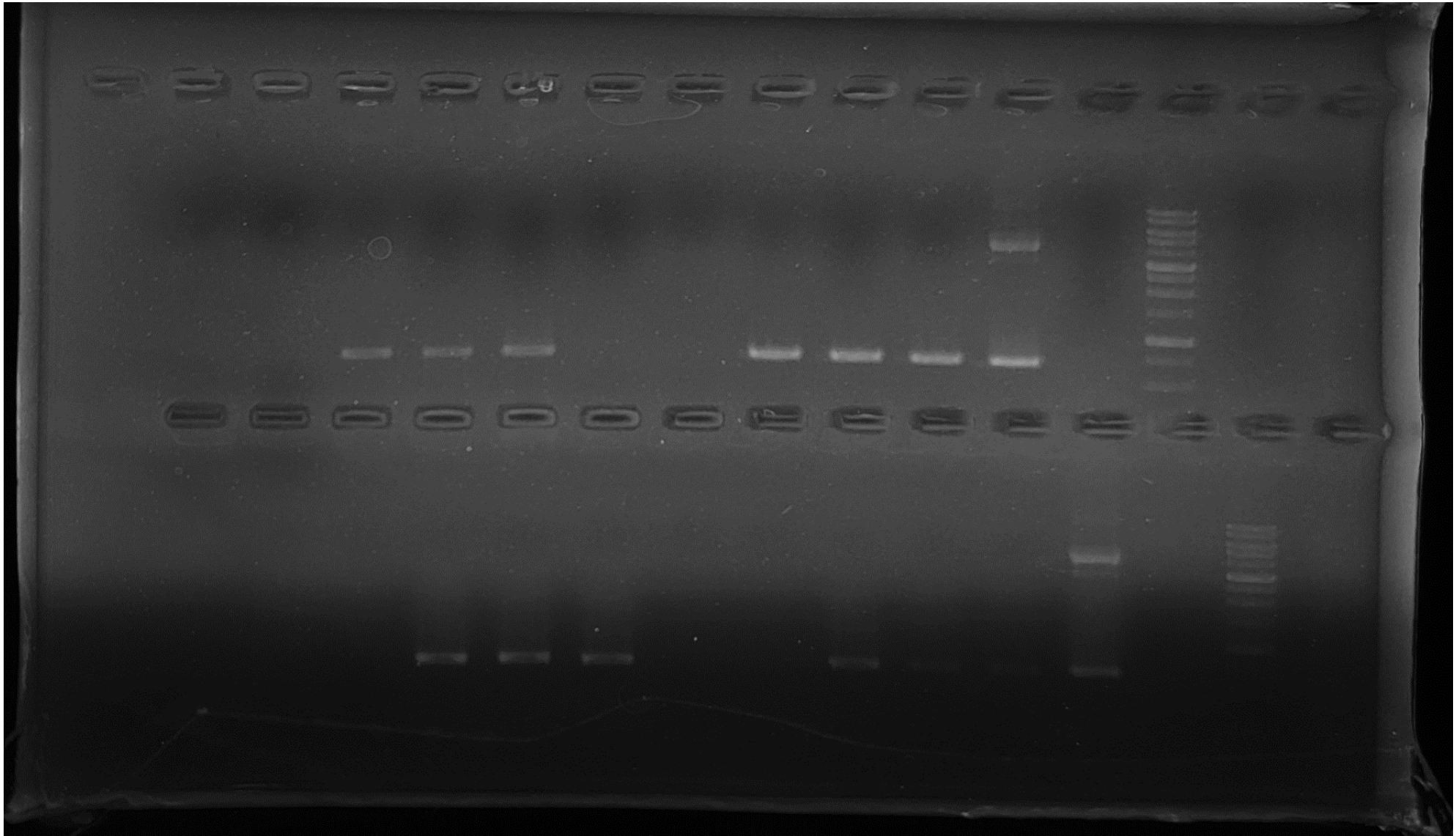

Figure 3B

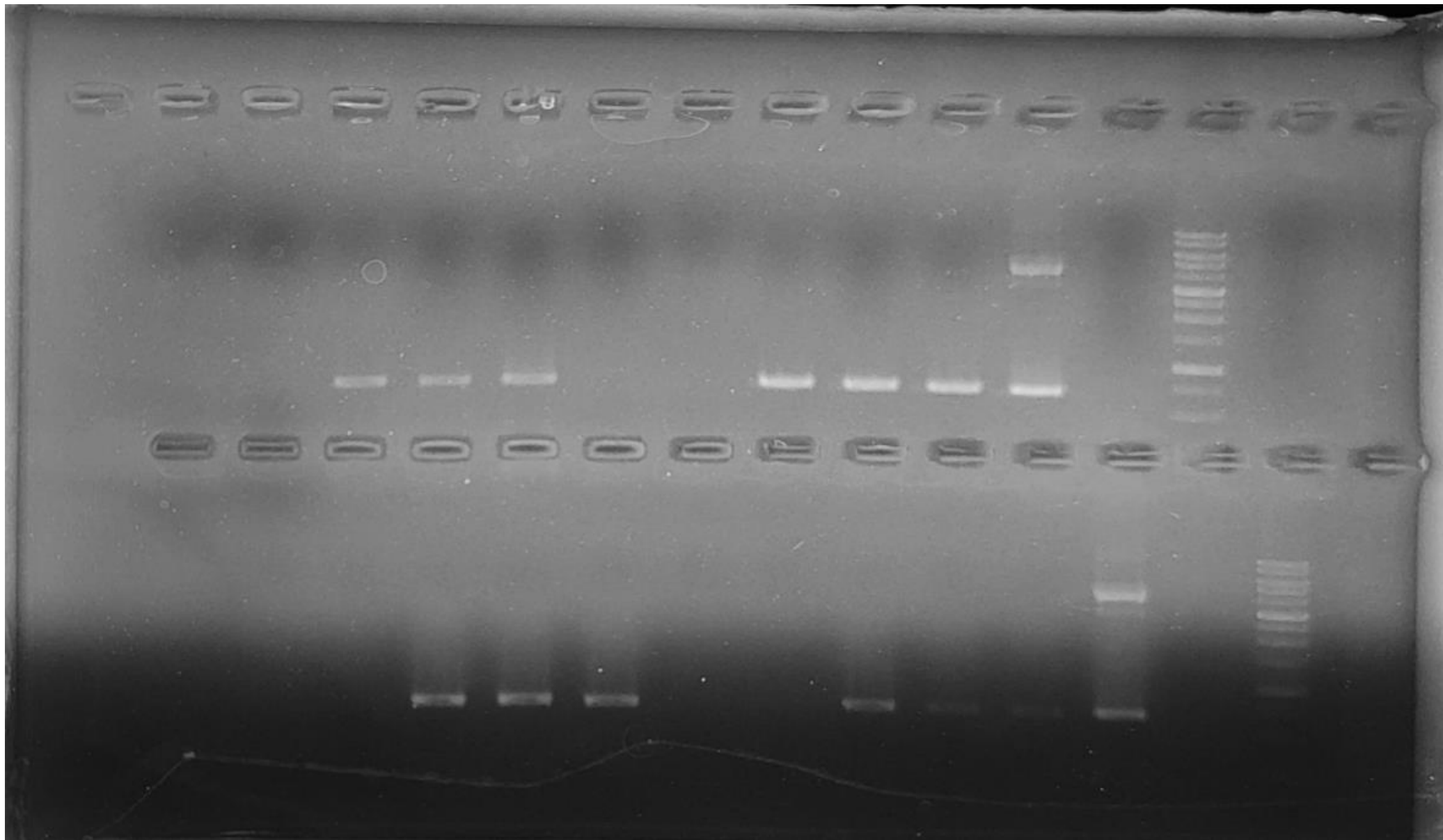

Figure 3C

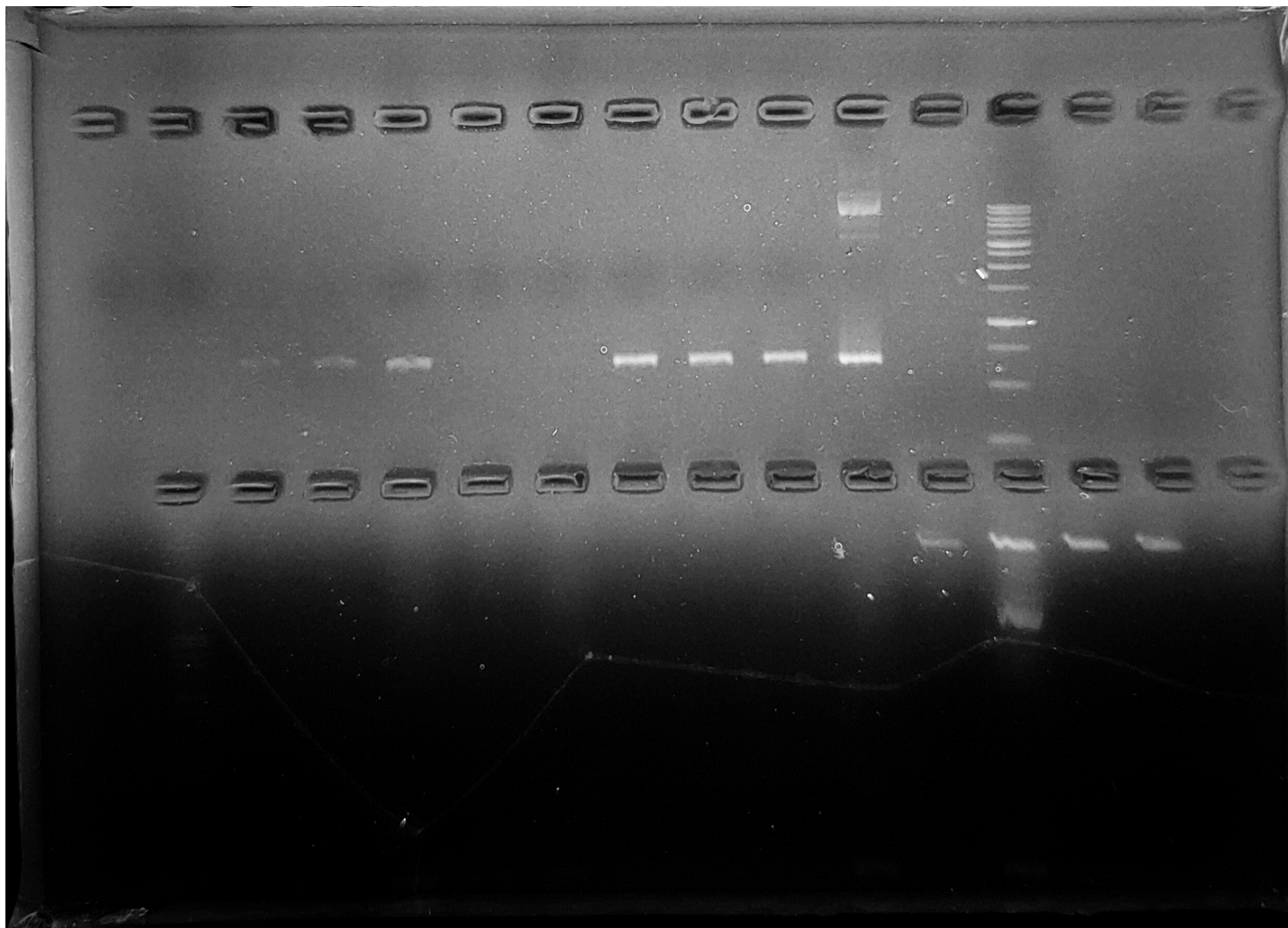

Figure 3D

Supplement: Supplemental Information 13 — Full gel images for Figure 3. Detection of the presence of MaERFVII3 in each T3 genotype sample of (B) Day 1, (C) Day 3, and (D) Day 5 samples. ‘+’ indicates positive control, while ‘−ve’ indicates negative control. M represents the ladder for 1 kb ladder. (WT: Wild-type, 1301: pCAMBIA 1301 control, L1: MaERFVII3-Line 1, L3: MaERFVII3-Line 3, L5: MaERFVII3-Line 5) [file peerj-12-17285-s013.pdf]
