# Supplementary material for: Expressing banana transcription factor MaERFVII3 in Arabidopsis confers enhanced waterlogging tolerance and root growth
Source: PeerJ. 2024 Apr 30;12:e17285. doi: 10.7717/peerj.17285 (PMC11067909; doi:10.7717/peerj.17285)
Supplement: Supplemental Information 16 [file peerj-12-17285-s016.pdf]

AtADH1

Primer pair 1

	Sequence (5'->3')	Length	Tm	GC%	Self complementarity	Self 3' complementarity
**Forward primer**	TATTCGATGCAAAGCTGCTGTG	22	59.90	45.45	7.00	7.00
**Reverse primer**	CGAACTTCGTGTTTCTGCGGT	21	61.72	52.38	7.00	0.00

Products on target templates

>NM_106362.3 Arabidopsis thaliana alcohol dehydrogenase 1 (ADH1), mRNA

```
product length = 93
Forward primer  1    TATTCGATGCAAAGCTGCTGTG  22
Template        275  ......................  296

Reverse primer  1    CGAACTTCGTGTTTCTGCGGT  21
Template        367  .....................  347
```

AtHRE2

Primer pair 1

	Sequence (5'->3')	Length	Tm	GC%	Self complementarity	Self 3' complementarity
**Forward primer**	GAAGCGTAAACCCGTCTCAGT	21	60.34	52.38	4.00	1.00
**Reverse primer**	TTTGCTCGGGCACGAATCT	19	60.00	52.63	6.00	1.00

Products on target templates

>NM_130320.4 Arabidopsis thaliana Integrase-type DNA-binding superfamily protein (ERF71), mRNA

```
product length = 123
Forward primer  1    GAAGCGTAAACCCGTCTCAGT  21
Template        299  .....................  319

Reverse primer  1    TTTGCTCGGG-CACGAATCT  19
Template        422  ..........T.........  403
```


```
product length = 123
Forward primer  1    GAAGCGTAAACCCGTCTCAGT  21
Template        299  .....................  319

Reverse primer  1    TTTGCTCGGG-CACGAATCT  19
Template        422  ..........T.........  403
```

AtLBD16

Primer pair 1

	Sequence (5'->3')	Length	Tm	GC%	Self complementarity	Self 3' complementarity
**Forward primer**	TCAAACCGGAGGAGGAGTAT	20	57.46	50.00	4.00	2.00
**Reverse primer**	AGCCTGAAGCTCACCTAAATC	21	57.73	47.62	5.00	0.00

Products on target templates

>NM_129804.4 Arabidopsis thaliana lateral organ boundaries-domain 16 (LBD16), mRNA

```
product length = 112
Forward primer  1    TCAAACCGGAGGAGGAGTAT  20
Template        915  ....................  934

Reverse primer  1     AGCCTGAAGCTCACCTAAATC  21
Template        1026  .....................  1006
```

AtLBD18

Primer pair 1

	Sequence (5'->3')	Length	Tm	GC%	Self complementarity	Self 3' complementarity
**Forward primer**	GAAGTGTGTGCCGGGATGTA	20	60.04	55.00	4.00	2.00
**Reverse primer**	CGTTACTGGCTCCGAACACT	20	60.04	55.00	3.00	1.00

Products on target templates

>NM_180105.2 Arabidopsis thaliana LOB domain-containing protein 18 (LBD18), mRNA

```
product length = 98
Forward primer  1    GAAGTGTGTGCCGGGATGTA  20
Template        391  ....................  410

Reverse primer  1    CGTTACTGGCTCCGAACACT  20
Template        488  ....................  469
```

AtPIN1

Primer pair 1

	Sequence (5'->3')	Length	Tm	GC%	Self complementarity	Self 3' complementarity
**Forward primer**	ACAAAACGACGCAGGCTAAG	20	59.13	50.00	3.00	3.00
**Reverse primer**	AGCTGGCATTTCAATGTTCC	20	56.96	45.00	6.00	1.00

Products on target templates

>NM_106017.4 Arabidopsis thaliana Auxin efflux carrier family protein (PIN1), mRNA

```
product length = 164
Forward primer  1     ACAAAACGACGCAGGCTAAG  20
Template        1439  ....................  1458

Reverse primer  1     AGCTGGCATTTCAATGTTCC  20
Template        1602  ....................  1583
```

AtRAP2.2

Primer pair 1

	Sequence (5'->3')	Length	Tm	GC%	Self complementarity	Self 3' complementarity
**Forward primer**	CATGGAAGAGAAGCCTCAGATG	22	58.53	50.00	4.00	3.00
**Reverse primer**	GCCCTGATCGGAACTGAAATA	21	57.81	47.62	4.00	2.00

Products on target templates

>NM_180251.3 Arabidopsis thaliana uncharacterized protein (RAP2.2), mRNA

```
product length = 103
Forward primer  1     CATGGAAGAGAAGCCTCAGATG  22
Template        1012  ......................  1033

Reverse primer  1     GCCCTGATCGGAACTGAAATA  21
Template        1114  .....................  1094
```

>NM_180252.1 Arabidopsis thaliana uncharacterized protein (RAP2.2), mRNA

```
product length = 103
Forward primer  1    CATGGAAGAGAAGCCTCAGATG  22
Template        847  ......................  868

Reverse primer  1    GCCCTGATCGGAACTGAAATA  21
Template        949  .....................  929
```

>NM_112281.2 Arabidopsis thaliana uncharacterized protein (RAP2.2), mRNA

```
product length = 103
Forward primer  1    CATGGAAGAGAAGCCTCAGATG  22
Template        862  ......................  883

Reverse primer  1    GCCCTGATCGGAACTGAAATA  21
Template        964  .....................  944
```

AtRAP2.12

Primer pair 1

	Sequence (5'->3')	Length	Tm	GC%	Self complementarity	Self 3' complementarity
**Forward primer**	TGCTGGATGTAATGGGTATCAG	22	57.64	45.45	3.00	1.00
**Reverse primer**	CAGAAGAGATGTCGGGAGTTATC	23	58.13	47.83	4.00	2.00

Products on target templates

>NM_001036108.3 Arabidopsis thaliana uncharacterized protein (RAP2.12), mRNA

```
product length = 113
Forward primer  1    TGCTGGATGTAATGGGTATCAG  22
Template        886  ......................  907

Reverse primer  1    CAGAAGAGATGTCGGGAGTTATC  23
Template        998  .......................  976
```

>NM_104269.4 Arabidopsis thaliana related to AP2 12 (RAP2.12), mRNA

```
product length = 113
Forward primer  1     TGCTGGATGTAATGGGTATCAG  22
Template        1142  ......................  1163

Reverse primer  1     CAGAAGAGATGTCGGGAGTTATC  23
Template        1254  .......................  1232
```

>NM_001084249.1 Arabidopsis thaliana uncharacterized protein (RAP2.12), mRNA

```
product length = 113
Forward primer  1    TGCTGGATGTAATGGGTATCAG  22
Template        868  ......................  889

Reverse primer  1    CAGAAGAGATGTCGGGAGTTATC  23
Template        980  .......................  958
```

AtTUB

Primer pair 1

	Sequence (5'->3')	Length	Tm	GC%	Self complementarity	Self 3' complementarity
**Forward primer**	GGTTGGTTTTGCTCCTCTCACC	22	61.65	54.55	3.00	0.00
**Reverse primer**	TAGCGTCCGTGCCTTGGGTC	20	64.62	65.00	2.00	1.00

Products on target templates

>NM_125665.4 Arabidopsis thaliana tubulin beta chain 3 (TUB3), mRNA

```
product length = 129
Forward primer  1     GGTTGGTTTTGCTCCTCTCACC  22
Template        1044  ......................  1065

Reverse primer  1     TAGCGTCCGTGCCTTGGGTC  20
Template        1172  ....................  1153
```

>NM_125664.4 Arabidopsis thaliana tubulin beta chain 2 (TUB2), mRNA

```
product length = 129
Forward primer  1     GGTTGGTTTTGCTCCTCTCACC  22
Template        1065  ...G..................  1086

Reverse primer  1     TAGCGTCCGTGCCTTGGGTC  20
Template        1193  ....................  1174
```

>NM_128508.3 Arabidopsis thaliana tubulin beta-7 chain (TUB7), mRNA

```
product length = 129
Forward primer  1    GGTTGGTTTTGCTCCTCTCACC  22
Template        926  ...G..A...............  947

Reverse primer  1     TAGCGTCCGTGCCTTGGGTC  20
Template        1054  .....A.....T........  1035
```

>NM_106228.3 Arabidopsis thaliana tubulin beta-1 chain (TUB1), mRNA

```
product length = 129
Forward primer  1     GGTTGGTTTTGCTCCTCTCACC  22
Template        1022  .........C..C........T  1043

Reverse primer  1     TAGCGTCCGTGCCTTGGGTC  20
Template        1150  ..A........G.G...A..  1131
```

>NM_101856.3 Arabidopsis thaliana tubulin beta-5 chain (TUB5), mRNA

```
product length = 129
Forward primer  1     GGTTGGTTTTGCTCCTCTCACC  22
Template        1155  ...G........G..C.....T  1176

Reverse primer  1     TAGCGTCCGTGCCTTGGGTC  20
Template        1283  ..A........A.G...A..  1264
```

AtUBQ

Primer pair 1

	Sequence (5'->3')	Length	Tm	GC%	Self complementarity	Self 3' complementarity
Forward primer	GGCCTTGTATAATCCCTGATGAATAAG	27	59.66	40.74	5.00	5.00
Reverse primer	AAAGAGATAACAGGAACGGAAACATAGT	28	60.74	35.71	2.00	2.00

Products on target templates

>NM_001084884.5 Arabidopsis thaliana polyubiquitin 10 (UBQ10), mRNA

```
product length = 61
Forward primer  1     GGCCTTGTATAATCCCTGATGAATAAG  27
Template        1493  ...........................  1519

Reverse primer  1     AAAGAGATAACAGGAACGGAAACATAGT  28
Template        1553  ............................  1526
```

>NM_001340546.1 Arabidopsis thaliana polyubiquitin 10 (UBQ10), mRNA

```
product length = 61
Forward primer  1     GGCCTTGTATAATCCCTGATGAATAAG  27
Template        619   ...........................  645

Reverse primer  1     AAAGAGATAACAGGAACGGAAACATAGT  28
Template        679   ............................  652
```

>NM_001340547.1 Arabidopsis thaliana polyubiquitin 10 (UBQ10), mRNA

```
product length = 61
Forward primer  1     GGCCTTGTATAATCCCTGATGAATAAG  27
Template        1058  ...........................  1084

Reverse primer  1     AAAGAGATAACAGGAACGGAAACATAGT  28
Template        1118  ............................  1091
```

>NM_178968.5 Arabidopsis thaliana polyubiquitin 10 (UBQ10), mRNA

```
product length = 61
Forward primer  1     GGCCTTGTATAATCCCTGATGAATAAG  27
Template        1724  ...........................  1750

Reverse primer  1     AAAGAGATAACAGGAACGGAAACATAGT  28
Template        1784  ............................  1757
```

>NM_116771.5 Arabidopsis thaliana polyubiquitin 10 (UBQ10), mRNA

```
product length = 61
Forward primer  1     GGCCTTGTATAATCCCTGATGAATAAG  27
Template        1303  ...........................  1329

Reverse primer  1     AAAGAGATAACAGGAACGGAAACATAGT  28
Template        1363  ............................  1336
```

>NM_202787.4 Arabidopsis thaliana polyubiquitin 10 (UBQ10), mRNA

```
product length = 61
Forward primer  1     GGCCTTGTATAATCCCTGATGAATAAG  27
Template        1075  ...........................  1101

Reverse primer  1     AAAGAGATAACAGGAACGGAAACATAGT  28
Template        1135  ............................  1108
```

>NM_178970.5 Arabidopsis thaliana polyubiquitin 10 (UBQ10), mRNA

```
product length = 61
Forward primer  1     GGCCTTGTATAATCCCTGATGAATAAG  27
Template        1496  ...........................  1522

Reverse primer  1     AAAGAGATAACAGGAACGGAAACATAGT  28
Template        1556  ............................  1529
```
