# Supplementary material for: Expressing banana transcription factor MaERFVII3 in Arabidopsis confers enhanced waterlogging tolerance and root growth
Source: PeerJ. 2024 Apr 30;12:e17285. doi: 10.7717/peerj.17285 (PMC11067909; doi:10.7717/peerj.17285)
Supplement: Supplemental Information 17 [file peerj-12-17285-s017.pdf]

>MaERFVII3

ATGTGTGGCGGCGCCATCATCTCCGACTTCATTCCTCAACGCAACCATCACCGTCACTGCCCCGATCCCGATCACC
TCCTCTCCGCCTCTGACCTCTGGCCCGATTCCTTCCACCCACACAGTGTCTCCACCCAGGACAAAGTGCCTCGGA
AGCGGGGGCGGAAGAATTTGTACAGGGGGATACGACAGCGGCCGTGGGGAAAGTGGGCAGCGGAAATCCGT
GACCCCATGAAAGGCGTTCGCGTCTGGCTCGGCACCTTCGCTACCGCGGAGGAGGCTGCCCGCGCCTACGACC
GCGAGGCCCGCCGCATCCGCGGCAAGAAGGCCAAGGTCAACTTCCCCAATGAGGTGGAGCCCGAGGAGACCG
ATAATTCCGTCCCCTGCAACCGCGTCCGCAATTTCATTCAACCCAAGGTGGAAAAATCTCCCGTCTTGGCGGCTT
CCAATGACTGCGAGGGAGACGGTGAGGTGAGGAGGCTGTCGGAGGAGCTGATGGCCTACGAGTCCTACATGA
ACTTCTTCGGTATCCCGTACATGGAGGGCGGCACTCCAGCGGCAGCGGCGGCGGCGGAGGAGGGGACAGCAG
CAGATGATGAGGTGGTCACAGGGAATGGAATGGAGGCGTGTAATCCGCCGGTCTCTTCAGGCATGGAGATGCT
TTGGATCTTCGACGACATTCTGCCGGCGTGA
