# Supplementary material for: Expressing banana transcription factor MaERFVII3 in Arabidopsis confers enhanced waterlogging tolerance and root growth
Source: PeerJ. 2024 Apr 30;12:e17285. doi: 10.7717/peerj.17285 (PMC11067909; doi:10.7717/peerj.17285)
Supplement: Supplemental Information 19 — Multiple sequence alignment of group VII ERFs illustrates the conservation of the DNA-binding domain (AP2/ERF domain) (Blue color box). [file peerj-12-17285-s019.docx]

CLUSTAL 2.1 multiple sequence alignment

NM_001159194.1 --------------------------------------------------

XM_015789203.1 --------------------------------------------------

XM_015774497.2 --------------------------------------------------

NM_001153709.2 --------------------------------------------------

NM_001254770.2 --------------------------------------------------

NM_001150750.2 --------------------------------------------------

CM007650.1_142530856-142531634 --------------------------------------------------

CM007648.1_220493755-220494609 --------------------------------------------------

NM_001111800.2 --------------------------------------------------

XM_023301819.1 --------------------------------------------------

XM_015775299.2 --------------------------------------------------

XM_009384249.2 --------------------------------------------------

XM_009385622.2 --------------------------------------------------

XM_009388210.2 --------------------------------------------------

XM_009389661.2 --------------------------------------------------

XM_009393219.2 --------------------------------------------------

XM_009415859.2 --------------------------------------------------

XM_008649541.3 --------------------------------------------------

XM_015782909.2 --------------------------------------------------

NM_001349820.1 --------------------------------------------------

EU837258.1 --------------------------------------------------

EU847517.1 --------------------------------------------------

XM_015775100.2 --------------------------------------------------

CT833260.1 --------------------------------------------------

AK067060.1 --------------------------------------------------

XM_015795858.2 --------------------------------------------------

EU847519.1 --------------------------------------------------

FR720457.1 --------------------------------------------------

EU847520.1 --------------------------------------------------

NM_001143039.1 --------------------------------------------------

AK073133.1 --------------------------------------------------

XM_006380178.2 --------------------------------------------------

XM_002315454.3 --------------------------------------------------

XM_008392395.2 --------------------------------------------------

XM_029095961.1 --------------------------------------------------

XM_002272390.4 GTTCTTTTTTCTTTTTATTTTTTTATGGAAAGAAACCACAAAATAGAGTA

NM_001256464.1 --------------------------------------------------

XM_003546320.5 --------------------------------------------------

NM_001279196.2 --------------------------------------------------

AY035100.1 --------------------------------------------------

JF968116.1 --------------------------------------------------

JF968119.1 --------------------------------------------------

NM_130320.4 --------------------------------------------------

NM_001248198.2 --------------------------------------------------

AY192368.1 --------------------------------------------------

NM_001247379.2 --------------------------------------------------

JX145122.1 --------------------------------------------------

XM_002301454.4 --------------------------------------------------

XM_002320960.3 --------------------------------------------------

NM_001320016.1 --------------------------------------------------

NM_001328756.1 --------------------------------------------------

FQ392750.1 --------------------------------------------------

NM_001254494.3 --------------------------------------------------

NM_001254517.2 --------------------------------------------------

AK316980.1 --------------------------------------------------

NM_180251.3 --------------------------------------------------

JF968115.1 --------------------------------------------------

JF968117.1 --------------------------------------------------

JF968118.1 --------------------------------------------------

NM_001247584.2 --------------------------------------------------

KJ401124.1 --------------------------------------------------

XM_004252354.4 --------------------------------------------------

XM_002266972.5 --------------------------------------------------

XM_024596482.1 --------------------------------------------------

XM_006369031.3 --------------------------------------------------

XM_008341964.3 --------------------------------------------------

NM_001294046.1 --------------------------------------------------

NM_001251371.2 --------------------------------------------------

XM_006604868.4 --------------------------------------------------

NM_001354284.1 --------------------------------------------------

NM_001349033.1 --------------------------------------------------

NM_001155962.1 --------------------------------------------------

NM_001157201.2 --------------------------------------------------

AK111690.1 --------------------------------------------------

XM_008660266.4 --------------------------------------------------

XM_015788266.2 --------------------------------------------------

NM_001156774.2 --------------------------------------------------

NM_001155219.2 --------------------------------------------------

EU837255.1 --------------------------------------------------

XM_009403037.2 --------------------------------------------------

XM_018823871.1 --------------------------------------------------

XM_009419716.2 --------------------------------------------------

XM_009419259.2 --------------------------------------------------

XM_009415720.2 --------------------------------------------------

XM_009411978.2 --------------------------------------------------

XM_009383333.2 --------------------------------------------------

XM_009381868.2 --------------------------------------------------

XM_009405824.2 --------------------------------------------------

XM_009389352.2 --------------------------------------------------

XM_009391309.2 --------------------------------------------------

NM_001084343.2 --------------------------------------------------

NM_001159194.1 --------------------------------------------------

XM_015789203.1 --------------------------------------------------

XM_015774497.2 --------------------------------------------------

NM_001153709.2 --------------------------------------------------

NM_001254770.2 --------------------------------------------------

NM_001150750.2 --------------------------------------------------

CM007650.1_142530856-142531634 --------------------------------------------------

CM007648.1_220493755-220494609 --------------------------------------------------

NM_001111800.2 --------------------------------------------------

XM_023301819.1 --------------------------------------------------

XM_015775299.2 --------------------------------------------------

XM_009384249.2 --------------------------------------------------

XM_009385622.2 --------------------------------------------------

XM_009388210.2 --------------------------------------------------

XM_009389661.2 --------------------------------------------------

XM_009393219.2 --------------------------------------------------

XM_009415859.2 --------------------------------------------------

XM_008649541.3 --------------------------------------------------

XM_015782909.2 --------------------------------------------------

NM_001349820.1 --------------------------------------------------

EU837258.1 --------------------------------------------------

EU847517.1 --------------------------------------------------

XM_015775100.2 --------------------------------------------------

CT833260.1 --------------------------------------------------

AK067060.1 --------------------------------------------------

XM_015795858.2 --------------------------------------------------

EU847519.1 --------------------------------------------------

FR720457.1 --------------------------------------------------

EU847520.1 --------------------------------------------------

NM_001143039.1 --------------------------------------------------

AK073133.1 --------------------------------------------------

XM_006380178.2 --------------------------------------------------

XM_002315454.3 --------------------------------------------------

XM_008392395.2 --------------------------------------------------

XM_029095961.1 --------------------------------------------------

XM_002272390.4 TTTACACATTCAATTTGAAAGAACTGCAATAATATTTGAACCCAAACTTA

NM_001256464.1 --------------------------------------------------

XM_003546320.5 --------------------------------------------------

NM_001279196.2 --------------------------------------------------

AY035100.1 --------------------------------------------------

JF968116.1 --------------------------------------------------

JF968119.1 --------------------------------------------------

NM_130320.4 --------------------------------------------------

NM_001248198.2 --------------------------------------------------

AY192368.1 --------------------------------------------------

NM_001247379.2 --------------------------------------------------

JX145122.1 --------------------------------------------------

XM_002301454.4 --------------------------------------------------

XM_002320960.3 --------------------------------------------------

NM_001320016.1 --------------------------------------------------

NM_001328756.1 --------------------------------------------------

FQ392750.1 --------------------------------------------------

NM_001254494.3 --------------------------------------------------

NM_001254517.2 --------------------------------------------------

AK316980.1 --------------------------------------------------

NM_180251.3 --------------------------------------------------

JF968115.1 --------------------------------------------------

JF968117.1 --------------------------------------------------

JF968118.1 --------------------------------------------------

NM_001247584.2 --------------------------------------------------

KJ401124.1 --------------------------------------------------

XM_004252354.4 --------------------------------------------------

XM_002266972.5 --------------------------------------------------

XM_024596482.1 --------------------------------------------------

XM_006369031.3 --------------------------------------------------

XM_008341964.3 --------------------------------------------------

NM_001294046.1 --------------------------------------------------

NM_001251371.2 --------------------------------------------------

XM_006604868.4 --------------------------------------------------

NM_001354284.1 --------------------------------------------------

NM_001349033.1 --------------------------------------------------

NM_001155962.1 --------------------------------------------------

NM_001157201.2 --------------------------------------------------

AK111690.1 --------------------------------------------------

XM_008660266.4 --------------------------------------------------

XM_015788266.2 --------------------------------------------------

NM_001156774.2 --------------------------------------------------

NM_001155219.2 --------------------------------------------------

EU837255.1 --------------------------------------------------

XM_009403037.2 --------------------------------------------------

XM_018823871.1 --------------------------------------------------

XM_009419716.2 --------------------------------------------------

XM_009419259.2 --------------------------------------------------

XM_009415720.2 --------------------------------------------------

XM_009411978.2 --------------------------------------------------

XM_009383333.2 --------------------------------------------------

XM_009381868.2 --------------------------------------------------

XM_009405824.2 --------------------------------------------------

XM_009389352.2 --------------------------------------------------

XM_009391309.2 --------------------------------------------------

NM_001084343.2 --------------------------------------------------

NM_001159194.1 --------------------------------------------------

XM_015789203.1 --------------------------------------------------

XM_015774497.2 --------------------------------------------------

NM_001153709.2 --------------------------------------------------

NM_001254770.2 --------------------------------------------------

NM_001150750.2 --------------------------------------------------

CM007650.1_142530856-142531634 --------------------------------------------------

CM007648.1_220493755-220494609 --------------------------------------------------

NM_001111800.2 --------------------------------------------------

XM_023301819.1 --------------------------------------------------

XM_015775299.2 --------------------------------------------------

XM_009384249.2 --------------------------------------------------

XM_009385622.2 --------------------------------------------------

XM_009388210.2 --------------------------------------------------

XM_009389661.2 --------------------------------------------------

XM_009393219.2 --------------------------------------------------

XM_009415859.2 --------------------------------------------------

XM_008649541.3 --------------------------------------------------

XM_015782909.2 --------------------------------------------------

NM_001349820.1 --------------------------------------------------

EU837258.1 --------------------------------------------------

EU847517.1 --------------------------------------------------

XM_015775100.2 --------------------------------------------------

CT833260.1 --------------------------------------------------

AK067060.1 --------------------------------------------------

XM_015795858.2 --------------------------------------------------

EU847519.1 --------------------------------------------------

FR720457.1 --------------------------------------------------

EU847520.1 --------------------------------------------------

NM_001143039.1 --------------------------------------------------

AK073133.1 --------------------------------------------------

XM_006380178.2 --------------------------------------------------

XM_002315454.3 --------------------------------------------------

XM_008392395.2 --------------------------------------------------

XM_029095961.1 --------------------------------------------------

XM_002272390.4 GTAACATTTCTAGGAAAATCTGACCTTCAAGTGAAAGACTTTAAATTATA

NM_001256464.1 --------------------------------------------------

XM_003546320.5 --------------------------------------------------

NM_001279196.2 --------------------------------------------------

AY035100.1 --------------------------------------------------

JF968116.1 --------------------------------------------------

JF968119.1 --------------------------------------------------

NM_130320.4 --------------------------------------------------

NM_001248198.2 --------------------------------------------------

AY192368.1 --------------------------------------------------

NM_001247379.2 --------------------------------------------------

JX145122.1 --------------------------------------------------

XM_002301454.4 --------------------------------------------------

XM_002320960.3 --------------------------------------------------

NM_001320016.1 --------------------------------------------------

NM_001328756.1 --------------------------------------------------

FQ392750.1 --------------------------------------------------

NM_001254494.3 --------------------------------------------------

NM_001254517.2 --------------------------------------------------

AK316980.1 --------------------------------------------------

NM_180251.3 --------------------------------------------------

JF968115.1 --------------------------------------------------

JF968117.1 --------------------------------------------------

JF968118.1 --------------------------------------------------

NM_001247584.2 --------------------------------------------------

KJ401124.1 --------------------------------------------------

XM_004252354.4 --------------------------------------------------

XM_002266972.5 --------------------------------------------------

XM_024596482.1 --------------------------------------------------

XM_006369031.3 --------------------------------------------------

XM_008341964.3 --------------------------------------------------

NM_001294046.1 --------------------------------------------------

NM_001251371.2 --------------------------------------------------

XM_006604868.4 --------------------------------------------------

NM_001354284.1 --------------------------------------------------

NM_001349033.1 --------------------------------------------------

NM_001155962.1 --------------------------------------------------

NM_001157201.2 --------------------------------------------------

AK111690.1 --------------------------------------------------

XM_008660266.4 --------------------------------------------------

XM_015788266.2 --------------------------------------------------

NM_001156774.2 --------------------------------------------------

NM_001155219.2 --------------------------------------------------

EU837255.1 --------------------------------------------------

XM_009403037.2 --------------------------------------------------

XM_018823871.1 --------------------------------------------------

XM_009419716.2 --------------------------------------------------

XM_009419259.2 --------------------------------------------------

XM_009415720.2 --------------------------------------------------

XM_009411978.2 --------------------------------------------------

XM_009383333.2 --------------------------------------------------

XM_009381868.2 --------------------------------------------------

XM_009405824.2 --------------------------------------------------

XM_009389352.2 --------------------------------------------------

XM_009391309.2 --------------------------------------------------

NM_001084343.2 --------------------------------------------------

NM_001159194.1 --------------------------------------------------

XM_015789203.1 --------------------------------------------------

XM_015774497.2 --------------------------------------------------

NM_001153709.2 --------------------------------------------------

NM_001254770.2 --------------------------------------------------

NM_001150750.2 --------------------------------------------------

CM007650.1_142530856-142531634 --------------------------------------------------

CM007648.1_220493755-220494609 --------------------------------------------------

NM_001111800.2 --------------------------------------------------

XM_023301819.1 --------------------------------------------------

XM_015775299.2 --------------------------------------------------

XM_009384249.2 --------------------------------------------------

XM_009385622.2 --------------------------------------------------

XM_009388210.2 --------------------------------------------------

XM_009389661.2 --------------------------------------------------

XM_009393219.2 --------------------------------------------------

XM_009415859.2 --------------------------------------------------

XM_008649541.3 --------------------------------------------------

XM_015782909.2 --------------------------------------------------

NM_001349820.1 --------------------------------------------------

EU837258.1 --------------------------------------------------

EU847517.1 --------------------------------------------------

XM_015775100.2 --------------------------------------------------

CT833260.1 --------------------------------------------------

AK067060.1 --------------------------------------------------

XM_015795858.2 --------------------------------------------------

EU847519.1 --------------------------------------------------

FR720457.1 --------------------------------------------------

EU847520.1 --------------------------------------------------

NM_001143039.1 --------------------------------------------------

AK073133.1 --------------------------------------------------

XM_006380178.2 --------------------------------------------------

XM_002315454.3 --------------------------------------------------

XM_008392395.2 --------------------------------------------------

XM_029095961.1 --------------------------------------------------

XM_002272390.4 GATACTTTTTTTAGCTAAACAATCTATTATTCTATTTGTTTTTCTATAAA

NM_001256464.1 --------------------------------------------------

XM_003546320.5 --------------------------------------------------

NM_001279196.2 --------------------------------------------------

AY035100.1 --------------------------------------------------

JF968116.1 --------------------------------------------------

JF968119.1 --------------------------------------------------

NM_130320.4 --------------------------------------------------

NM_001248198.2 --------------------------------------------------

AY192368.1 --------------------------------------------------

NM_001247379.2 --------------------------------------------------

JX145122.1 --------------------------------------------------

XM_002301454.4 --------------------------------------------------

XM_002320960.3 --------------------------------------------------

NM_001320016.1 --------------------------------------------------

NM_001328756.1 --------------------------------------------------

FQ392750.1 --------------------------------------------------

NM_001254494.3 --------------------------------------------------

NM_001254517.2 --------------------------------------------------

AK316980.1 --------------------------------------------------

NM_180251.3 --------------------------------------------------

JF968115.1 --------------------------------------------------

JF968117.1 --------------------------------------------------

JF968118.1 --------------------------------------------------

NM_001247584.2 --------------------------------------------------

KJ401124.1 --------------------------------------------------

XM_004252354.4 --------------------------------------------------

XM_002266972.5 --------------------------------------------------

XM_024596482.1 --------------------------------------------------

XM_006369031.3 --------------------------------------------------

XM_008341964.3 --------------------------------------------------

NM_001294046.1 --------------------------------------------------

NM_001251371.2 --------------------------------------------------

XM_006604868.4 --------------------------------------------------

NM_001354284.1 --------------------------------------------------

NM_001349033.1 --------------------------------------------------

NM_001155962.1 --------------------------------------------------

NM_001157201.2 --------------------------------------------------

AK111690.1 --------------------------------------------------

XM_008660266.4 --------------------------------------------------

XM_015788266.2 --------------------------------------------------

NM_001156774.2 --------------------------------------------------

NM_001155219.2 --------------------------------------------------

EU837255.1 --------------------------------------------------

XM_009403037.2 --------------------------------------------------

XM_018823871.1 --------------------------------------------------

XM_009419716.2 --------------------------------------------------

XM_009419259.2 --------------------------------------------------

XM_009415720.2 --------------------------------------------------

XM_009411978.2 --------------------------------------------------

XM_009383333.2 --------------------------------------------------

XM_009381868.2 --------------------------------------------------

XM_009405824.2 --------------------------------------------------

XM_009389352.2 --------------------------------------------------

XM_009391309.2 --------------------------------------------------

NM_001084343.2 --------------------------------------------------

NM_001159194.1 --------------------------------------------------

XM_015789203.1 --------------------------------------------------

XM_015774497.2 --------------------------------------------------

NM_001153709.2 --------------------------------------------------

NM_001254770.2 --------------------------------------------------

NM_001150750.2 --------------------------------------------------

CM007650.1_142530856-142531634 --------------------------------------------------

CM007648.1_220493755-220494609 --------------------------------------------------

NM_001111800.2 --------------------------------------------------

XM_023301819.1 --------------------------------------------------

XM_015775299.2 --------------------------------------------------

XM_009384249.2 --------------------------------------------------

XM_009385622.2 --------------------------------------------------

XM_009388210.2 --------------------------------------------------

XM_009389661.2 --------------------------------------------------

XM_009393219.2 --------------------------------------------------

XM_009415859.2 --------------------------------------------------

XM_008649541.3 --------------------------------------------------

XM_015782909.2 --------------------------------------------------

NM_001349820.1 --------------------------------------------------

EU837258.1 --------------------------------------------------

EU847517.1 --------------------------------------------------

XM_015775100.2 --------------------------------------------------

CT833260.1 --------------------------------------------------

AK067060.1 --------------------------------------------------

XM_015795858.2 --------------------------------------------------

EU847519.1 --------------------------------------------------

FR720457.1 --------------------------------------------------

EU847520.1 --------------------------------------------------

NM_001143039.1 --------------------------------------------------

AK073133.1 --------------------------------------------------

XM_006380178.2 --------------------------------------------------

XM_002315454.3 --------------------------------------------------

XM_008392395.2 --------------------------------------------------

XM_029095961.1 --------------------------------------------------

XM_002272390.4 TATGCCAATAAATACAAACATTAAGATCTTGAGTTATTTCCACATATCTT

NM_001256464.1 --------------------------------------------------

XM_003546320.5 --------------------------------------------------

NM_001279196.2 --------------------------------------------------

AY035100.1 --------------------------------------------------

JF968116.1 --------------------------------------------------

JF968119.1 --------------------------------------------------

NM_130320.4 --------------------------------------------------

NM_001248198.2 --------------------------------------------------

AY192368.1 --------------------------------------------------

NM_001247379.2 --------------------------------------------------

JX145122.1 --------------------------------------------------

XM_002301454.4 --------------------------------------------------

XM_002320960.3 --------------------------------------------------

NM_001320016.1 --------------------------------------------------

NM_001328756.1 --------------------------------------------------

FQ392750.1 --------------------------------------------------

NM_001254494.3 --------------------------------------------------

NM_001254517.2 --------------------------------------------------

AK316980.1 --------------------------------------------------

NM_180251.3 --------------------------------------------------

JF968115.1 --------------------------------------------------

JF968117.1 --------------------------------------------------

JF968118.1 --------------------------------------------------

NM_001247584.2 --------------------------------------------------

KJ401124.1 --------------------------------------------------

XM_004252354.4 --------------------------------------------------

XM_002266972.5 --------------------------------------------------

XM_024596482.1 --------------------------------------------------

XM_006369031.3 --------------------------------------------------

XM_008341964.3 --------------------------------------------------

NM_001294046.1 --------------------------------------------------

NM_001251371.2 --------------------------------------------------

XM_006604868.4 --------------------------------------------------

NM_001354284.1 --------------------------------------------------

NM_001349033.1 --------------------------------------------------

NM_001155962.1 --------------------------------------------------

NM_001157201.2 --------------------------------------------------

AK111690.1 --------------------------------------------------

XM_008660266.4 --------------------------------------------------

XM_015788266.2 --------------------------------------------------

NM_001156774.2 --------------------------------------------------

NM_001155219.2 --------------------------------------------------

EU837255.1 --------------------------------------------------

XM_009403037.2 --------------------------------------------------

XM_018823871.1 --------------------------------------------------

XM_009419716.2 --------------------------------------------------

XM_009419259.2 --------------------------------------------------

XM_009415720.2 --------------------------------------------------

XM_009411978.2 --------------------------------------------------

XM_009383333.2 --------------------------------------------------

XM_009381868.2 --------------------------------------------------

XM_009405824.2 --------------------------------------------------

XM_009389352.2 --------------------------------------------------

XM_009391309.2 --------------------------------------------------

NM_001084343.2 --------------------------------------------------

NM_001159194.1 --------------------------------------------------

XM_015789203.1 --------------------------------------------------

XM_015774497.2 --------------------------------------------------

NM_001153709.2 --------------------------------------------------

NM_001254770.2 --------------------------------------------------

NM_001150750.2 --------------------------------------------------

CM007650.1_142530856-142531634 --------------------------------------------------

CM007648.1_220493755-220494609 --------------------------------------------------

NM_001111800.2 --------------------------------------------------

XM_023301819.1 --------------------------------------------------

XM_015775299.2 --------------------------------------------------

XM_009384249.2 --------------------------------------------------

XM_009385622.2 --------------------------------------------------

XM_009388210.2 --------------------------------------------------

XM_009389661.2 --------------------------------------------------

XM_009393219.2 --------------------------------------------------

XM_009415859.2 --------------------------------------------------

XM_008649541.3 --------------------------------------------------

XM_015782909.2 --------------------------------------------------

NM_001349820.1 --------------------------------------------------

EU837258.1 --------------------------------------------------

EU847517.1 --------------------------------------------------

XM_015775100.2 --------------------------------------------------

CT833260.1 --------------------------------------------------

AK067060.1 --------------------------------------------------

XM_015795858.2 --------------------------------------------------

EU847519.1 --------------------------------------------------

FR720457.1 --------------------------------------------------

EU847520.1 --------------------------------------------------

NM_001143039.1 --------------------------------------------------

AK073133.1 --------------------------------------------------

XM_006380178.2 --------------------------------------------------

XM_002315454.3 --------------------------------------------------

XM_008392395.2 --------------------------------------------------

XM_029095961.1 --------------------------------------------------

XM_002272390.4 CCATTAACAACATAATGGAACTAGGAATGCTACTTTTTTTATTTTAATTA

NM_001256464.1 --------------------------------------------------

XM_003546320.5 --------------------------------------------------

NM_001279196.2 --------------------------------------------------

AY035100.1 --------------------------------------------------

JF968116.1 --------------------------------------------------

JF968119.1 --------------------------------------------------

NM_130320.4 --------------------------------------------------

NM_001248198.2 --------------------------------------------------

AY192368.1 --------------------------------------------------

NM_001247379.2 --------------------------------------------------

JX145122.1 --------------------------------------------------

XM_002301454.4 --------------------------------------------------

XM_002320960.3 --------------------------------------------------

NM_001320016.1 --------------------------------------------------

NM_001328756.1 --------------------------------------------------

FQ392750.1 --------------------------------------------------

NM_001254494.3 --------------------------------------------------

NM_001254517.2 --------------------------------------------------

AK316980.1 --------------------------------------------------

NM_180251.3 --------------------------------------------------

JF968115.1 --------------------------------------------------

JF968117.1 --------------------------------------------------

JF968118.1 --------------------------------------------------

NM_001247584.2 --------------------------------------------------

KJ401124.1 --------------------------------------------------

XM_004252354.4 --------------------------------------------------

XM_002266972.5 --------------------------------------------------

XM_024596482.1 --------------------------------------------------

XM_006369031.3 --------------------------------------------------

XM_008341964.3 --------------------------------------------------

NM_001294046.1 --------------------------------------------------

NM_001251371.2 --------------------------------------------------

XM_006604868.4 --------------------------------------------------

NM_001354284.1 --------------------------------------------------

NM_001349033.1 --------------------------------------------------

NM_001155962.1 --------------------------------------------------

NM_001157201.2 --------------------------------------------------

AK111690.1 --------------------------------------------------

XM_008660266.4 --------------------------------------------------

XM_015788266.2 --------------------------------------------------

NM_001156774.2 --------------------------------------------------

NM_001155219.2 --------------------------------------------------

EU837255.1 --------------------------------------------------

XM_009403037.2 --------------------------------------------------

XM_018823871.1 --------------------------------------------------

XM_009419716.2 --------------------------------------------------

XM_009419259.2 --------------------------------------------------

XM_009415720.2 --------------------------------------------------

XM_009411978.2 --------------------------------------------------

XM_009383333.2 --------------------------------------------------

XM_009381868.2 --------------------------------------------------

XM_009405824.2 --------------------------------------------------

XM_009389352.2 --------------------------------------------------

XM_009391309.2 --------------------------------------------------

NM_001084343.2 --------------------------------------------------

NM_001159194.1 --------------------------------------------------

XM_015789203.1 --------------------------------------------------

XM_015774497.2 --------------------------------------------------

NM_001153709.2 --------------------------------------------------

NM_001254770.2 --------------------------------------------------

NM_001150750.2 --------------------------------------------------

CM007650.1_142530856-142531634 --------------------------------------------------

CM007648.1_220493755-220494609 --------------------------------------------------

NM_001111800.2 --------------------------------------------------

XM_023301819.1 --------------------------------------------------

XM_015775299.2 --------------------------------------------------

XM_009384249.2 --------------------------------------------------

XM_009385622.2 --------------------------------------------------

XM_009388210.2 --------------------------------------------------

XM_009389661.2 --------------------------------------------------

XM_009393219.2 --------------------------------------------------

XM_009415859.2 --------------------------------------------------

XM_008649541.3 --------------------------------------------------

XM_015782909.2 --------------------------------------------------

NM_001349820.1 --------------------------------------------------

EU837258.1 --------------------------------------------------

EU847517.1 --------------------------------------------------

XM_015775100.2 --------------------------------------------------

CT833260.1 --------------------------------------------------

AK067060.1 --------------------------------------------------

XM_015795858.2 --------------------------------------------------

EU847519.1 --------------------------------------------------

FR720457.1 --------------------------------------------------

EU847520.1 --------------------------------------------------

NM_001143039.1 --------------------------------------------------

AK073133.1 --------------------------------------------------

XM_006380178.2 --------------------------------------------------

XM_002315454.3 --------------------------------------------------

XM_008392395.2 --------------------------------------------------

XM_029095961.1 --------------------------------------------------

XM_002272390.4 TCTATCACTACTGTTGAGTCACCTTCAATCTTTAAATTTAAGAACCCAGT

NM_001256464.1 --------------------------------------------------

XM_003546320.5 --------------------------------------------------

NM_001279196.2 --------------------------------------------------

AY035100.1 --------------------------------------------------

JF968116.1 --------------------------------------------------

JF968119.1 --------------------------------------------------

NM_130320.4 --------------------------------------------------

NM_001248198.2 --------------------------------------------------

AY192368.1 --------------------------------------------------

NM_001247379.2 --------------------------------------------------

JX145122.1 --------------------------------------------------

XM_002301454.4 --------------------------------------------------

XM_002320960.3 --------------------------------------------------

NM_001320016.1 --------------------------------------------------

NM_001328756.1 --------------------------------------------------

FQ392750.1 --------------------------------------------------

NM_001254494.3 --------------------------------------------------

NM_001254517.2 --------------------------------------------------

AK316980.1 --------------------------------------------------

NM_180251.3 --------------------------------------------------

JF968115.1 --------------------------------------------------

JF968117.1 --------------------------------------------------

JF968118.1 --------------------------------------------------

NM_001247584.2 --------------------------------------------------

KJ401124.1 --------------------------------------------------

XM_004252354.4 --------------------------------------------------

XM_002266972.5 --------------------------------------------------

XM_024596482.1 --------------------------------------------------

XM_006369031.3 --------------------------------------------------

XM_008341964.3 --------------------------------------------------

NM_001294046.1 --------------------------------------------------

NM_001251371.2 --------------------------------------------------

XM_006604868.4 --------------------------------------------------

NM_001354284.1 --------------------------------------------------

NM_001349033.1 --------------------------------------------------

NM_001155962.1 --------------------------------------------------

NM_001157201.2 --------------------------------------------------

AK111690.1 --------------------------------------------------

XM_008660266.4 --------------------------------------------------

XM_015788266.2 --------------------------------------------------

NM_001156774.2 --------------------------------------------------

NM_001155219.2 --------------------------------------------------

EU837255.1 --------------------------------------------------

XM_009403037.2 --------------------------------------------------

XM_018823871.1 --------------------------------------------------

XM_009419716.2 --------------------------------------------------

XM_009419259.2 --------------------------------------------------

XM_009415720.2 --------------------------------------------------

XM_009411978.2 --------------------------------------------------

XM_009383333.2 --------------------------------------------------

XM_009381868.2 --------------------------------------------------

XM_009405824.2 --------------------------------------------------

XM_009389352.2 --------------------------------------------------

XM_009391309.2 --------------------------------------------------

NM_001084343.2 --------------------------------------------------

NM_001159194.1 --------------------------------------------------

XM_015789203.1 --------------------------------------------------

XM_015774497.2 --------------------------------------------------

NM_001153709.2 --------------------------------------------------

NM_001254770.2 --------------------------------------------------

NM_001150750.2 --------------------------------------------------

CM007650.1_142530856-142531634 --------------------------------------------------

CM007648.1_220493755-220494609 --------------------------------------------------

NM_001111800.2 --------------------------------------------------

XM_023301819.1 --------------------------------------------------

XM_015775299.2 --------------------------------------------------

XM_009384249.2 --------------------------------------------------

XM_009385622.2 --------------------------------------------------

XM_009388210.2 --------------------------------------------------

XM_009389661.2 --------------------------------------------------

XM_009393219.2 --------------------------------------------------

XM_009415859.2 --------------------------------------------------

XM_008649541.3 --------------------------------------------------

XM_015782909.2 --------------------------------------------------

NM_001349820.1 --------------------------------------------------

EU837258.1 --------------------------------------------------

EU847517.1 --------------------------------------------------

XM_015775100.2 --------------------------------------------------

CT833260.1 --------------------------------------------------

AK067060.1 --------------------------------------------------

XM_015795858.2 --------------------------------------------------

EU847519.1 --------------------------------------------------

FR720457.1 --------------------------------------------------

EU847520.1 --------------------------------------------------

NM_001143039.1 --------------------------------------------------

AK073133.1 --------------------------------------------------

XM_006380178.2 --------------------------------------------------

XM_002315454.3 --------------------------------------------------

XM_008392395.2 --------------------------------------------------

XM_029095961.1 --------------------------------------------------

XM_002272390.4 TACTAACACACCATCTCTAAAAGTTAACATATTGACGTATTGCCCATATG

NM_001256464.1 --------------------------------------------------

XM_003546320.5 --------------------------------------------------

NM_001279196.2 --------------------------------------------------

AY035100.1 --------------------------------------------------

JF968116.1 --------------------------------------------------

JF968119.1 --------------------------------------------------

NM_130320.4 --------------------------------------------------

NM_001248198.2 --------------------------------------------------

AY192368.1 --------------------------------------------------

NM_001247379.2 --------------------------------------------------

JX145122.1 --------------------------------------------------

XM_002301454.4 --------------------------------------------------

XM_002320960.3 --------------------------------------------------

NM_001320016.1 --------------------------------------------------

NM_001328756.1 --------------------------------------------------

FQ392750.1 --------------------------------------------------

NM_001254494.3 --------------------------------------------------

NM_001254517.2 --------------------------------------------------

AK316980.1 --------------------------------------------------

NM_180251.3 --------------------------------------------------

JF968115.1 --------------------------------------------------

JF968117.1 --------------------------------------------------

JF968118.1 --------------------------------------------------

NM_001247584.2 --------------------------------------------------

KJ401124.1 --------------------------------------------------

XM_004252354.4 --------------------------------------------------

XM_002266972.5 --------------------------------------------------

XM_024596482.1 --------------------------------------------------

XM_006369031.3 --------------------------------------------------

XM_008341964.3 --------------------------------------------------

NM_001294046.1 --------------------------------------------------

NM_001251371.2 --------------------------------------------------

XM_006604868.4 --------------------------------------------------

NM_001354284.1 --------------------------------------------------

NM_001349033.1 --------------------------------------------------

NM_001155962.1 --------------------------------------------------

NM_001157201.2 --------------------------------------------------

AK111690.1 --------------------------------------------------

XM_008660266.4 --------------------------------------------------

XM_015788266.2 --------------------------------------------------

NM_001156774.2 --------------------------------------------------

NM_001155219.2 --------------------------------------------------

EU837255.1 --------------------------------------------------

XM_009403037.2 --------------------------------------------------

XM_018823871.1 --------------------------------------------------

XM_009419716.2 --------------------------------------------------

XM_009419259.2 --------------------------------------------------

XM_009415720.2 --------------------------------------------------

XM_009411978.2 --------------------------------------------------

XM_009383333.2 --------------------------------------------------

XM_009381868.2 --------------------------------------------------

XM_009405824.2 --------------------------------------------------

XM_009389352.2 --------------------------------------------------

XM_009391309.2 --------------------------------------------------

NM_001084343.2 --------------------------------------------------

NM_001159194.1 --------------------------------------------------

XM_015789203.1 --------------------------------------------------

XM_015774497.2 --------------------------------------------------

NM_001153709.2 --------------------------------------------------

NM_001254770.2 --------------------------------------------------

NM_001150750.2 --------------------------------------------------

CM007650.1_142530856-142531634 --------------------------------------------------

CM007648.1_220493755-220494609 --------------------------------------------------

NM_001111800.2 --------------------------------------------------

XM_023301819.1 --------------------------------------------------

XM_015775299.2 --------------------------------------------------

XM_009384249.2 --------------------------------------------------

XM_009385622.2 --------------------------------------------------

XM_009388210.2 --------------------------------------------------

XM_009389661.2 --------------------------------------------------

XM_009393219.2 --------------------------------------------------

XM_009415859.2 --------------------------------------------------

XM_008649541.3 --------------------------------------------------

XM_015782909.2 --------------------------------------------------

NM_001349820.1 --------------------------------------------------

EU837258.1 --------------------------------------------------

EU847517.1 --------------------------------------------------

XM_015775100.2 --------------------------------------------------

CT833260.1 --------------------------------------------------

AK067060.1 --------------------------------------------------

XM_015795858.2 --------------------------------------------------

EU847519.1 --------------------------------------------------

FR720457.1 --------------------------------------------------

EU847520.1 --------------------------------------------------

NM_001143039.1 --------------------------------------------------

AK073133.1 --------------------------------------------------

XM_006380178.2 --------------------------------------------------

XM_002315454.3 --------------------------------------------------

XM_008392395.2 --------------------------------------------------

XM_029095961.1 --------------------------------------------------

XM_002272390.4 CTTATTTATAGTCATTTTTATTATACCATTAGAATCTCTAATAAGCCATC

NM_001256464.1 --------------------------------------------------

XM_003546320.5 --------------------------------------------------

NM_001279196.2 --------------------------------------------------

AY035100.1 --------------------------------------------------

JF968116.1 --------------------------------------------------

JF968119.1 --------------------------------------------------

NM_130320.4 --------------------------------------------------

NM_001248198.2 --------------------------------------------------

AY192368.1 --------------------------------------------------

NM_001247379.2 --------------------------------------------------

JX145122.1 --------------------------------------------------

XM_002301454.4 --------------------------------------------------

XM_002320960.3 --------------------------------------------------

NM_001320016.1 --------------------------------------------------

NM_001328756.1 --------------------------------------------------

FQ392750.1 --------------------------------------------------

NM_001254494.3 --------------------------------------------------

NM_001254517.2 --------------------------------------------------

AK316980.1 --------------------------------------------------

NM_180251.3 --------------------------------------------------

JF968115.1 --------------------------------------------------

JF968117.1 --------------------------------------------------

JF968118.1 --------------------------------------------------

NM_001247584.2 --------------------------------------------------

KJ401124.1 --------------------------------------------------

XM_004252354.4 --------------------------------------------------

XM_002266972.5 --------------------------------------------------

XM_024596482.1 --------------------------------------------------

XM_006369031.3 --------------------------------------------------

XM_008341964.3 --------------------------------------------------

NM_001294046.1 --------------------------------------------------

NM_001251371.2 --------------------------------------------------

XM_006604868.4 --------------------------------------------------

NM_001354284.1 --------------------------------------------------

NM_001349033.1 --------------------------------------------------

NM_001155962.1 --------------------------------------------------

NM_001157201.2 --------------------------------------------------

AK111690.1 --------------------------------------------------

XM_008660266.4 --------------------------------------------------

XM_015788266.2 --------------------------------------------------

NM_001156774.2 --------------------------------------------------

NM_001155219.2 --------------------------------------------------

EU837255.1 --------------------------------------------------

XM_009403037.2 --------------------------------------------------

XM_018823871.1 --------------------------------------------------

XM_009419716.2 --------------------------------------------------

XM_009419259.2 --------------------------------------------------

XM_009415720.2 --------------------------------------------------

XM_009411978.2 --------------------------------------------------

XM_009383333.2 --------------------------------------------------

XM_009381868.2 --------------------------------------------------

XM_009405824.2 --------------------------------------------------

XM_009389352.2 --------------------------------------------------

XM_009391309.2 --------------------------------------------------

NM_001084343.2 --------------------------------------------------

NM_001159194.1 --------------------------------------------------

XM_015789203.1 --------------------------------------------------

XM_015774497.2 --------------------------------------------------

NM_001153709.2 --------------------------------------------------

NM_001254770.2 --------------------------------------------------

NM_001150750.2 --------------------------------------------------

CM007650.1_142530856-142531634 --------------------------------------------------

CM007648.1_220493755-220494609 --------------------------------------------------

NM_001111800.2 --------------------------------------------------

XM_023301819.1 --------------------------------------------------

XM_015775299.2 --------------------------------------------------

XM_009384249.2 --------------------------------------------------

XM_009385622.2 --------------------------------------------------

XM_009388210.2 --------------------------------------------------

XM_009389661.2 --------------------------------------------------

XM_009393219.2 --------------------------------------------------

XM_009415859.2 --------------------------------------------------

XM_008649541.3 --------------------------------------------------

XM_015782909.2 --------------------------------------------------

NM_001349820.1 --------------------------------------------------

EU837258.1 --------------------------------------------------

EU847517.1 --------------------------------------------------

XM_015775100.2 --------------------------------------------------

CT833260.1 --------------------------------------------------

AK067060.1 --------------------------------------------------

XM_015795858.2 --------------------------------------------------

EU847519.1 --------------------------------------------------

FR720457.1 --------------------------------------------------

EU847520.1 --------------------------------------------------

NM_001143039.1 --------------------------------------------------

AK073133.1 --------------------------------------------------

XM_006380178.2 --------------------------------------------------

XM_002315454.3 --------------------------------------------------

XM_008392395.2 --------------------------------------------------

XM_029095961.1 --------------------------------------------------

XM_002272390.4 TTAATGCACCTCTATTCTCTGCCTTGAGCCATCCTTGTTTTCAATTTTAG

NM_001256464.1 --------------------------------------------------

XM_003546320.5 --------------------------------------------------

NM_001279196.2 --------------------------------------------------

AY035100.1 --------------------------------------------------

JF968116.1 --------------------------------------------------

JF968119.1 --------------------------------------------------

NM_130320.4 --------------------------------------------------

NM_001248198.2 --------------------------------------------------

AY192368.1 --------------------------------------------------

NM_001247379.2 --------------------------------------------------

JX145122.1 --------------------------------------------------

XM_002301454.4 --------------------------------------------------

XM_002320960.3 --------------------------------------------------

NM_001320016.1 --------------------------------------------------

NM_001328756.1 --------------------------------------------------

FQ392750.1 --------------------------------------------------

NM_001254494.3 --------------------------------------------------

NM_001254517.2 --------------------------------------------------

AK316980.1 --------------------------------------------------

NM_180251.3 --------------------------------------------------

JF968115.1 --------------------------------------------------

JF968117.1 --------------------------------------------------

JF968118.1 --------------------------------------------------

NM_001247584.2 --------------------------------------------------

KJ401124.1 --------------------------------------------------

XM_004252354.4 --------------------------------------------------

XM_002266972.5 --------------------------------------------------

XM_024596482.1 --------------------------------------------------

XM_006369031.3 --------------------------------------------------

XM_008341964.3 --------------------------------------------------

NM_001294046.1 --------------------------------------------------

NM_001251371.2 --------------------------------------------------

XM_006604868.4 --------------------------------------------------

NM_001354284.1 --------------------------------------------------

NM_001349033.1 --------------------------------------------------

NM_001155962.1 --------------------------------------------------

NM_001157201.2 --------------------------------------------------

AK111690.1 --------------------------------------------------

XM_008660266.4 --------------------------------------------------

XM_015788266.2 --------------------------------------------------

NM_001156774.2 --------------------------------------------------

NM_001155219.2 --------------------------------------------------

EU837255.1 --------------------------------------------------

XM_009403037.2 --------------------------------------------------

XM_018823871.1 --------------------------------------------------

XM_009419716.2 --------------------------------------------------

XM_009419259.2 --------------------------------------------------

XM_009415720.2 --------------------------------------------------

XM_009411978.2 --------------------------------------------------

XM_009383333.2 --------------------------------------------------

XM_009381868.2 --------------------------------------------------

XM_009405824.2 --------------------------------------------------

XM_009389352.2 --------------------------------------------------

XM_009391309.2 --------------------------------------------------

NM_001084343.2 --------------------------------------------------

NM_001159194.1 --------------------------------------------------

XM_015789203.1 --------------------------------------------------

XM_015774497.2 --------------------------------------------------

NM_001153709.2 --------------------------------------------------

NM_001254770.2 --------------------------------------------------

NM_001150750.2 --------------------------------------------------

CM007650.1_142530856-142531634 --------------------------------------------------

CM007648.1_220493755-220494609 --------------------------------------------------

NM_001111800.2 --------------------------------------------------

XM_023301819.1 --------------------------------------------------

XM_015775299.2 --------------------------------------------------

XM_009384249.2 --------------------------------------------------

XM_009385622.2 --------------------------------------------------

XM_009388210.2 --------------------------------------------------

XM_009389661.2 --------------------------------------------------

XM_009393219.2 --------------------------------------------------

XM_009415859.2 --------------------------------------------------

XM_008649541.3 --------------------------------------------------

XM_015782909.2 --------------------------------------------------

NM_001349820.1 --------------------------------------------------

EU837258.1 --------------------------------------------------

EU847517.1 --------------------------------------------------

XM_015775100.2 --------------------------------------------------

CT833260.1 --------------------------------------------------

AK067060.1 --------------------------------------------------

XM_015795858.2 --------------------------------------------------

EU847519.1 --------------------------------------------------

FR720457.1 --------------------------------------------------

EU847520.1 --------------------------------------------------

NM_001143039.1 --------------------------------------------------

AK073133.1 --------------------------------------------------

XM_006380178.2 --------------------------------------------------

XM_002315454.3 --------------------------------------------------

XM_008392395.2 --------------------------------------------------

XM_029095961.1 --------------------------------------------------

XM_002272390.4 GTCTTTCTGCCTTGCATGATCATTTTTCTCTTTTTATTTTAGGGTGTGTT

NM_001256464.1 --------------------------------------------------

XM_003546320.5 --------------------------------------------------

NM_001279196.2 --------------------------------------------------

AY035100.1 --------------------------------------------------

JF968116.1 --------------------------------------------------

JF968119.1 --------------------------------------------------

NM_130320.4 --------------------------------------------------

NM_001248198.2 --------------------------------------------------

AY192368.1 --------------------------------------------------

NM_001247379.2 --------------------------------------------------

JX145122.1 --------------------------------------------------

XM_002301454.4 --------------------------------------------------

XM_002320960.3 --------------------------------------------------

NM_001320016.1 --------------------------------------------------

NM_001328756.1 --------------------------------------------------

FQ392750.1 --------------------------------------------------

NM_001254494.3 --------------------------------------------------

NM_001254517.2 --------------------------------------------------

AK316980.1 --------------------------------------------------

NM_180251.3 --------------------------------------------------

JF968115.1 --------------------------------------------------

JF968117.1 --------------------------------------------------

JF968118.1 --------------------------------------------------

NM_001247584.2 --------------------------------------------------

KJ401124.1 --------------------------------------------------

XM_004252354.4 --------------------------------------------------

XM_002266972.5 --------------------------------------------------

XM_024596482.1 --------------------------------------------------

XM_006369031.3 --------------------------------------------------

XM_008341964.3 --------------------------------------------------

NM_001294046.1 --------------------------------------------------

NM_001251371.2 --------------------------------------------------

XM_006604868.4 --------------------------------------------------

NM_001354284.1 --------------------------------------------------

NM_001349033.1 --------------------------------------------------

NM_001155962.1 --------------------------------------------------

NM_001157201.2 --------------------------------------------------

AK111690.1 --------------------------------------------------

XM_008660266.4 --------------------------------------------------

XM_015788266.2 --------------------------------------------------

NM_001156774.2 --------------------------------------------------

NM_001155219.2 --------------------------------------------------

EU837255.1 --------------------------------------------------

XM_009403037.2 --------------------------------------------------

XM_018823871.1 --------------------------------------------------

XM_009419716.2 --------------------------------------------------

XM_009419259.2 --------------------------------------------------

XM_009415720.2 --------------------------------------------------

XM_009411978.2 --------------------------------------------------

XM_009383333.2 --------------------------------------------------

XM_009381868.2 --------------------------------------------------

XM_009405824.2 --------------------------------------------------

XM_009389352.2 --------------------------------------------------

XM_009391309.2 --------------------------------------------------

NM_001084343.2 --------------------------------------------------

NM_001159194.1 --------------------------------------------------

XM_015789203.1 --------------------------------------------------

XM_015774497.2 --------------------------------------------------

NM_001153709.2 --------------------------------------------------

NM_001254770.2 --------------------------------------------------

NM_001150750.2 --------------------------------------------------

CM007650.1_142530856-142531634 --------------------------------------------------

CM007648.1_220493755-220494609 --------------------------------------------------

NM_001111800.2 --------------------------------------------------

XM_023301819.1 --------------------------------------------------

XM_015775299.2 --------------------------------------------------

XM_009384249.2 --------------------------------------------------

XM_009385622.2 --------------------------------------------------

XM_009388210.2 --------------------------------------------------

XM_009389661.2 --------------------------------------------------

XM_009393219.2 --------------------------------------------------

XM_009415859.2 --------------------------------------------------

XM_008649541.3 --------------------------------------------------

XM_015782909.2 --------------------------------------------------

NM_001349820.1 --------------------------------------------------

EU837258.1 --------------------------------------------------

EU847517.1 --------------------------------------------------

XM_015775100.2 --------------------------------------------------

CT833260.1 --------------------------------------------------

AK067060.1 --------------------------------------------------

XM_015795858.2 --------------------------------------------------

EU847519.1 --------------------------------------------------

FR720457.1 --------------------------------------------------

EU847520.1 --------------------------------------------------

NM_001143039.1 --------------------------------------------------

AK073133.1 --------------------------------------------------

XM_006380178.2 --------------------------------------------------

XM_002315454.3 --------------------------------------------------

XM_008392395.2 --------------------------------------------------

XM_029095961.1 --------------------------------------------------

XM_002272390.4 TGGTGCCTGAAAATACAGTACCATATCCAGAAAAATAATCTAAACCCTCG

NM_001256464.1 --------------------------------------------------

XM_003546320.5 --------------------------------------------------

NM_001279196.2 --------------------------------------------------

AY035100.1 --------------------------------------------------

JF968116.1 --------------------------------------------------

JF968119.1 --------------------------------------------------

NM_130320.4 --------------------------------------------------

NM_001248198.2 --------------------------------------------------

AY192368.1 --------------------------------------------------

NM_001247379.2 --------------------------------------------------

JX145122.1 --------------------------------------------------

XM_002301454.4 --------------------------------------------------

XM_002320960.3 --------------------------------------------------

NM_001320016.1 --------------------------------------------------

NM_001328756.1 --------------------------------------------------

FQ392750.1 --------------------------------------------------

NM_001254494.3 --------------------------------------------------

NM_001254517.2 --------------------------------------------------

AK316980.1 --------------------------------------------------

NM_180251.3 --------------------------------------------------

JF968115.1 --------------------------------------------------

JF968117.1 --------------------------------------------------

JF968118.1 --------------------------------------------------

NM_001247584.2 --------------------------------------------------

KJ401124.1 --------------------------------------------------

XM_004252354.4 --------------------------------------------------

XM_002266972.5 --------------------------------------------------

XM_024596482.1 --------------------------------------------------

XM_006369031.3 --------------------------------------------------

XM_008341964.3 --------------------------------------------------

NM_001294046.1 --------------------------------------------------

NM_001251371.2 --------------------------------------------------

XM_006604868.4 --------------------------------------------------

NM_001354284.1 --------------------------------------------------

NM_001349033.1 --------------------------------------------------

NM_001155962.1 --------------------------------------------------

NM_001157201.2 --------------------------------------------------

AK111690.1 --------------------------------------------------

XM_008660266.4 --------------------------------------------------

XM_015788266.2 --------------------------------------------------

NM_001156774.2 --------------------------------------------------

NM_001155219.2 --------------------------------------------------

EU837255.1 --------------------------------------------------

XM_009403037.2 --------------------------------------------------

XM_018823871.1 --------------------------------------------------

XM_009419716.2 --------------------------------------------------

XM_009419259.2 --------------------------------------------------

XM_009415720.2 --------------------------------------------------

XM_009411978.2 --------------------------------------------------

XM_009383333.2 --------------------------------------------------

XM_009381868.2 --------------------------------------------------

XM_009405824.2 --------------------------------------------------

XM_009389352.2 --------------------------------------------------

XM_009391309.2 --------------------------------------------------

NM_001084343.2 --------------------------------------------------

NM_001159194.1 --------------------------------------------------

XM_015789203.1 --------------------------------------------------

XM_015774497.2 --------------------------------------------------

NM_001153709.2 --------------------------------------------------

NM_001254770.2 --------------------------------------------------

NM_001150750.2 --------------------------------------------------

CM007650.1_142530856-142531634 --------------------------------------------------

CM007648.1_220493755-220494609 --------------------------------------------------

NM_001111800.2 --------------------------------------------------

XM_023301819.1 --------------------------------------------------

XM_015775299.2 --------------------------------------------------

XM_009384249.2 --------------------------------------------------

XM_009385622.2 --------------------------------------------------

XM_009388210.2 --------------------------------------------------

XM_009389661.2 --------------------------------------------------

XM_009393219.2 --------------------------------------------------

XM_009415859.2 --------------------------------------------------

XM_008649541.3 --------------------------------------------------

XM_015782909.2 --------------------------------------------------

NM_001349820.1 --------------------------------------------------

EU837258.1 --------------------------------------------------

EU847517.1 --------------------------------------------------

XM_015775100.2 --------------------------------------------------

CT833260.1 --------------------------------------------------

AK067060.1 --------------------------------------------------

XM_015795858.2 --------------------------------------------------

EU847519.1 --------------------------------------------------

FR720457.1 --------------------------------------------------

EU847520.1 --------------------------------------------------

NM_001143039.1 --------------------------------------------------

AK073133.1 --------------------------------------------------

XM_006380178.2 --------------------------------------------------

XM_002315454.3 --------------------------------------------------

XM_008392395.2 --------------------------------------------------

XM_029095961.1 --------------------------------------------------

XM_002272390.4 TACAAATGGGTTTTGGTTTTATTCTTTTTTCTGATTCTAAACAAGGATTT

NM_001256464.1 --------------------------------------------------

XM_003546320.5 --------------------------------------------------

NM_001279196.2 --------------------------------------------------

AY035100.1 --------------------------------------------------

JF968116.1 --------------------------------------------------

JF968119.1 --------------------------------------------------

NM_130320.4 --------------------------------------------------

NM_001248198.2 --------------------------------------------------

AY192368.1 --------------------------------------------------

NM_001247379.2 --------------------------------------------------

JX145122.1 --------------------------------------------------

XM_002301454.4 --------------------------------------------------

XM_002320960.3 --------------------------------------------------

NM_001320016.1 --------------------------------------------------

NM_001328756.1 --------------------------------------------------

FQ392750.1 --------------------------------------------------

NM_001254494.3 --------------------------------------------------

NM_001254517.2 --------------------------------------------------

AK316980.1 --------------------------------------------------

NM_180251.3 --------------------------------------------------

JF968115.1 --------------------------------------------------

JF968117.1 --------------------------------------------------

JF968118.1 --------------------------------------------------

NM_001247584.2 --------------------------------------------------

KJ401124.1 --------------------------------------------------

XM_004252354.4 --------------------------------------------------

XM_002266972.5 --------------------------------------------------

XM_024596482.1 --------------------------------------------------

XM_006369031.3 --------------------------------------------------

XM_008341964.3 --------------------------------------------------

NM_001294046.1 --------------------------------------------------

NM_001251371.2 --------------------------------------------------

XM_006604868.4 --------------------------------------------------

NM_001354284.1 --------------------------------------------------

NM_001349033.1 --------------------------------------------------

NM_001155962.1 --------------------------------------------------

NM_001157201.2 --------------------------------------------------

AK111690.1 --------------------------------------------------

XM_008660266.4 --------------------------------------------------

XM_015788266.2 --------------------------------------------------

NM_001156774.2 --------------------------------------------------

NM_001155219.2 --------------------------------------------------

EU837255.1 --------------------------------------------------

XM_009403037.2 --------------------------------------------------

XM_018823871.1 --------------------------------------------------

XM_009419716.2 --------------------------------------------------

XM_009419259.2 --------------------------------------------------

XM_009415720.2 --------------------------------------------------

XM_009411978.2 --------------------------------------------------

XM_009383333.2 --------------------------------------------------

XM_009381868.2 --------------------------------------------------

XM_009405824.2 --------------------------------------------------

XM_009389352.2 --------------------------------------------------

XM_009391309.2 --------------------------------------------------

NM_001084343.2 --------------------------------------------------

NM_001159194.1 --------------------------------------------------

XM_015789203.1 --------------------------------------------------

XM_015774497.2 --------------------------------------------------

NM_001153709.2 --------------------------------------------------

NM_001254770.2 --------------------------------------------------

NM_001150750.2 --------------------------------------------------

CM007650.1_142530856-142531634 --------------------------------------------------

CM007648.1_220493755-220494609 --------------------------------------------------

NM_001111800.2 --------------------------------------------------

XM_023301819.1 --------------------------------------------------

XM_015775299.2 --------------------------------------------------

XM_009384249.2 --------------------------------------------------

XM_009385622.2 --------------------------------------------------

XM_009388210.2 --------------------------------------------------

XM_009389661.2 --------------------------------------------------

XM_009393219.2 --------------------------------------------------

XM_009415859.2 --------------------------------------------------

XM_008649541.3 --------------------------------------------------

XM_015782909.2 --------------------------------------------------

NM_001349820.1 --------------------------------------------------

EU837258.1 --------------------------------------------------

EU847517.1 --------------------------------------------------

XM_015775100.2 --------------------------------------------------

CT833260.1 --------------------------------------------------

AK067060.1 --------------------------------------------------

XM_015795858.2 --------------------------------------------------

EU847519.1 --------------------------------------------------

FR720457.1 --------------------------------------------------

EU847520.1 --------------------------------------------------

NM_001143039.1 --------------------------------------------------

AK073133.1 --------------------------------------------------

XM_006380178.2 --------------------------------------------------

XM_002315454.3 --------------------------------------------------

XM_008392395.2 --------------------------------------------------

XM_029095961.1 --------------------------------------------------

XM_002272390.4 ACTTCAGCATTGTAAACATGTTTTTTTTTTTTTAAGTAATAAAGCCTTGA

NM_001256464.1 --------------------------------------------------

XM_003546320.5 --------------------------------------------------

NM_001279196.2 --------------------------------------------------

AY035100.1 --------------------------------------------------

JF968116.1 --------------------------------------------------

JF968119.1 --------------------------------------------------

NM_130320.4 --------------------------------------------------

NM_001248198.2 --------------------------------------------------

AY192368.1 --------------------------------------------------

NM_001247379.2 --------------------------------------------------

JX145122.1 --------------------------------------------------

XM_002301454.4 --------------------------------------------------

XM_002320960.3 --------------------------------------------------

NM_001320016.1 --------------------------------------------------

NM_001328756.1 --------------------------------------------------

FQ392750.1 --------------------------------------------------

NM_001254494.3 --------------------------------------------------

NM_001254517.2 --------------------------------------------------

AK316980.1 --------------------------------------------------

NM_180251.3 --------------------------------------------------

JF968115.1 --------------------------------------------------

JF968117.1 --------------------------------------------------

JF968118.1 --------------------------------------------------

NM_001247584.2 --------------------------------------------------

KJ401124.1 --------------------------------------------------

XM_004252354.4 --------------------------------------------------

XM_002266972.5 --------------------------------------------------

XM_024596482.1 --------------------------------------------------

XM_006369031.3 --------------------------------------------------

XM_008341964.3 --------------------------------------------------

NM_001294046.1 --------------------------------------------------

NM_001251371.2 --------------------------------------------------

XM_006604868.4 --------------------------------------------------

NM_001354284.1 --------------------------------------------------

NM_001349033.1 --------------------------------------------------

NM_001155962.1 --------------------------------------------------

NM_001157201.2 --------------------------------------------------

AK111690.1 --------------------------------------------------

XM_008660266.4 --------------------------------------------------

XM_015788266.2 --------------------------------------------------

NM_001156774.2 --------------------------------------------------

NM_001155219.2 --------------------------------------------------

EU837255.1 --------------------------------------------------

XM_009403037.2 --------------------------------------------------

XM_018823871.1 --------------------------------------------------

XM_009419716.2 --------------------------------------------------

XM_009419259.2 --------------------------------------------------

XM_009415720.2 --------------------------------------------------

XM_009411978.2 --------------------------------------------------

XM_009383333.2 --------------------------------------------------

XM_009381868.2 --------------------------------------------------

XM_009405824.2 --------------------------------------------------

XM_009389352.2 --------------------------------------------------

XM_009391309.2 --------------------------------------------------

NM_001084343.2 --------------------------------------------------

NM_001159194.1 --------------------------------------------------

XM_015789203.1 --------------------------------------------------

XM_015774497.2 --------------------------------------------------

NM_001153709.2 --------------------------------------------------

NM_001254770.2 --------------------------------------------------

NM_001150750.2 --------------------------------------------------

CM007650.1_142530856-142531634 --------------------------------------------------

CM007648.1_220493755-220494609 --------------------------------------------------

NM_001111800.2 --------------------------------------------------

XM_023301819.1 --------------------------------------------------

XM_015775299.2 --------------------------------------------------

XM_009384249.2 --------------------------------------------------

XM_009385622.2 --------------------------------------------------

XM_009388210.2 --------------------------------------------------

XM_009389661.2 --------------------------------------------------

XM_009393219.2 --------------------------------------------------

XM_009415859.2 --------------------------------------------------

XM_008649541.3 --------------------------------------------------

XM_015782909.2 --------------------------------------------------

NM_001349820.1 --------------------------------------------------

EU837258.1 --------------------------------------------------

EU847517.1 --------------------------------------------------

XM_015775100.2 --------------------------------------------------

CT833260.1 --------------------------------------------------

AK067060.1 --------------------------------------------------

XM_015795858.2 --------------------------------------------------

EU847519.1 --------------------------------------------------

FR720457.1 --------------------------------------------------

EU847520.1 --------------------------------------------------

NM_001143039.1 --------------------------------------------------

AK073133.1 --------------------------------------------------

XM_006380178.2 --------------------------------------------------

XM_002315454.3 --------------------------------------------------

XM_008392395.2 --------------------------------------------------

XM_029095961.1 --------------------------------------------------

XM_002272390.4 ATTTGGCCCATATGCTAGGGCTATGGGCTCTAACCCAGTATGAGCGTTTA

NM_001256464.1 --------------------------------------------------

XM_003546320.5 --------------------------------------------------

NM_001279196.2 --------------------------------------------------

AY035100.1 --------------------------------------------------

JF968116.1 --------------------------------------------------

JF968119.1 --------------------------------------------------

NM_130320.4 --------------------------------------------------

NM_001248198.2 --------------------------------------------------

AY192368.1 --------------------------------------------------

NM_001247379.2 --------------------------------------------------

JX145122.1 --------------------------------------------------

XM_002301454.4 --------------------------------------------------

XM_002320960.3 --------------------------------------------------

NM_001320016.1 --------------------------------------------------

NM_001328756.1 --------------------------------------------------

FQ392750.1 --------------------------------------------------

NM_001254494.3 --------------------------------------------------

NM_001254517.2 --------------------------------------------------

AK316980.1 --------------------------------------------------

NM_180251.3 --------------------------------------------------

JF968115.1 --------------------------------------------------

JF968117.1 --------------------------------------------------

JF968118.1 --------------------------------------------------

NM_001247584.2 --------------------------------------------------

KJ401124.1 --------------------------------------------------

XM_004252354.4 --------------------------------------------------

XM_002266972.5 --------------------------------------------------

XM_024596482.1 --------------------------------------------------

XM_006369031.3 --------------------------------------------------

XM_008341964.3 --------------------------------------------------

NM_001294046.1 --------------------------------------------------

NM_001251371.2 --------------------------------------------------

XM_006604868.4 --------------------------------------------------

NM_001354284.1 --------------------------------------------------

NM_001349033.1 --------------------------------------------------

NM_001155962.1 --------------------------------------------------

NM_001157201.2 --------------------------------------------------

AK111690.1 --------------------------------------------------

XM_008660266.4 --------------------------------------------------

XM_015788266.2 --------------------------------------------------

NM_001156774.2 --------------------------------------------------

NM_001155219.2 --------------------------------------------------

EU837255.1 --------------------------------------------------

XM_009403037.2 --------------------------------------------------

XM_018823871.1 --------------------------------------------------

XM_009419716.2 --------------------------------------------------

XM_009419259.2 --------------------------------------------------

XM_009415720.2 --------------------------------------------------

XM_009411978.2 --------------------------------------------------

XM_009383333.2 --------------------------------------------------

XM_009381868.2 --------------------------------------------------

XM_009405824.2 --------------------------------------------------

XM_009389352.2 --------------------------------------------------

XM_009391309.2 --------------------------------------------------

NM_001084343.2 --------------------------------------------------

NM_001159194.1 --------------------------------------------------

XM_015789203.1 --------------------------------------------------

XM_015774497.2 --------------------------------------------------

NM_001153709.2 --------------------------------------------------

NM_001254770.2 --------------------------------------------------

NM_001150750.2 --------------------------------------------------

CM007650.1_142530856-142531634 --------------------------------------------------

CM007648.1_220493755-220494609 --------------------------------------------------

NM_001111800.2 --------------------------------------------------

XM_023301819.1 --------------------------------------------------

XM_015775299.2 --------------------------------------------------

XM_009384249.2 --------------------------------------------------

XM_009385622.2 --------------------------------------------------

XM_009388210.2 --------------------------------------------------

XM_009389661.2 --------------------------------------------------

XM_009393219.2 --------------------------------------------------

XM_009415859.2 --------------------------------------------------

XM_008649541.3 --------------------------------------------------

XM_015782909.2 --------------------------------------------------

NM_001349820.1 --------------------------------------------------

EU837258.1 --------------------------------------------------

EU847517.1 --------------------------------------------------

XM_015775100.2 --------------------------------------------------

CT833260.1 --------------------------------------------------

AK067060.1 --------------------------------------------------

XM_015795858.2 --------------------------------------------------

EU847519.1 --------------------------------------------------

FR720457.1 --------------------------------------------------

EU847520.1 --------------------------------------------------

NM_001143039.1 --------------------------------------------------

AK073133.1 --------------------------------------------------

XM_006380178.2 --------------------------------------------------

XM_002315454.3 --------------------------------------------------

XM_008392395.2 --------------------------------------------------

XM_029095961.1 --------------------------------------------------

XM_002272390.4 TTATTATTATTATTTTTTTAATTTTGGGTGAACTAAAGATGGAAATTAGA

NM_001256464.1 --------------------------------------------------

XM_003546320.5 --------------------------------------------------

NM_001279196.2 --------------------------------------------------

AY035100.1 --------------------------------------------------

JF968116.1 --------------------------------------------------

JF968119.1 --------------------------------------------------

NM_130320.4 --------------------------------------------------

NM_001248198.2 --------------------------------------------------

AY192368.1 --------------------------------------------------

NM_001247379.2 --------------------------------------------------

JX145122.1 --------------------------------------------------

XM_002301454.4 --------------------------------------------------

XM_002320960.3 --------------------------------------------------

NM_001320016.1 --------------------------------------------------

NM_001328756.1 --------------------------------------------------

FQ392750.1 --------------------------------------------------

NM_001254494.3 --------------------------------------------------

NM_001254517.2 --------------------------------------------------

AK316980.1 --------------------------------------------------

NM_180251.3 --------------------------------------------------

JF968115.1 --------------------------------------------------

JF968117.1 --------------------------------------------------

JF968118.1 --------------------------------------------------

NM_001247584.2 --------------------------------------------------

KJ401124.1 --------------------------------------------------

XM_004252354.4 --------------------------------------------------

XM_002266972.5 --------------------------------------------------

XM_024596482.1 --------------------------------------------------

XM_006369031.3 --------------------------------------------------

XM_008341964.3 --------------------------------------------------

NM_001294046.1 --------------------------------------------------

NM_001251371.2 --------------------------------------------------

XM_006604868.4 --------------------------------------------------

NM_001354284.1 --------------------------------------------------

NM_001349033.1 --------------------------------------------------

NM_001155962.1 --------------------------------------------------

NM_001157201.2 --------------------------------------------------

AK111690.1 --------------------------------------------------

XM_008660266.4 --------------------------------------------------

XM_015788266.2 --------------------------------------------------

NM_001156774.2 --------------------------------------------------

NM_001155219.2 --------------------------------------------------

EU837255.1 --------------------------------------------------

XM_009403037.2 --------------------------------------------------

XM_018823871.1 --------------------------------------------------

XM_009419716.2 --------------------------------------------------

XM_009419259.2 --------------------------------------------------

XM_009415720.2 --------------------------------------------------

XM_009411978.2 --------------------------------------------------

XM_009383333.2 --------------------------------------------------

XM_009381868.2 --------------------------------------------------

XM_009405824.2 --------------------------------------------------

XM_009389352.2 --------------------------------------------------

XM_009391309.2 --------------------------------------------------

NM_001084343.2 --------------------------------------------------

NM_001159194.1 --------------------------------------------------

XM_015789203.1 --------------------------------------------------

XM_015774497.2 --------------------------------------------------

NM_001153709.2 --------------------------------------------------

NM_001254770.2 --------------------------------------------------

NM_001150750.2 --------------------------------------------------

CM007650.1_142530856-142531634 --------------------------------------------------

CM007648.1_220493755-220494609 --------------------------------------------------

NM_001111800.2 --------------------------------------------------

XM_023301819.1 --------------------------------------------------

XM_015775299.2 --------------------------------------------------

XM_009384249.2 --------------------------------------------------

XM_009385622.2 --------------------------------------------------

XM_009388210.2 --------------------------------------------------

XM_009389661.2 --------------------------------------------------

XM_009393219.2 --------------------------------------------------

XM_009415859.2 --------------------------------------------------

XM_008649541.3 --------------------------------------------------

XM_015782909.2 --------------------------------------------------

NM_001349820.1 --------------------------------------------------

EU837258.1 --------------------------------------------------

EU847517.1 --------------------------------------------------

XM_015775100.2 --------------------------------------------------

CT833260.1 --------------------------------------------------

AK067060.1 --------------------------------------------------

XM_015795858.2 --------------------------------------------------

EU847519.1 --------------------------------------------------

FR720457.1 --------------------------------------------------

EU847520.1 --------------------------------------------------

NM_001143039.1 --------------------------------------------------

AK073133.1 --------------------------------------------------

XM_006380178.2 --------------------------------------------------

XM_002315454.3 --------------------------------------------------

XM_008392395.2 --------------------------------------------------

XM_029095961.1 --------------------------------------------------

XM_002272390.4 ATAGCACGATAGATATAGAAAATTTTAAATTCTGTCAAACAAAGACAACT

NM_001256464.1 --------------------------------------------------

XM_003546320.5 --------------------------------------------------

NM_001279196.2 --------------------------------------------------

AY035100.1 --------------------------------------------------

JF968116.1 --------------------------------------------------

JF968119.1 --------------------------------------------------

NM_130320.4 --------------------------------------------------

NM_001248198.2 --------------------------------------------------

AY192368.1 --------------------------------------------------

NM_001247379.2 --------------------------------------------------

JX145122.1 --------------------------------------------------

XM_002301454.4 --------------------------------------------------

XM_002320960.3 --------------------------------------------------

NM_001320016.1 --------------------------------------------------

NM_001328756.1 --------------------------------------------------

FQ392750.1 --------------------------------------------------

NM_001254494.3 --------------------------------------------------

NM_001254517.2 --------------------------------------------------

AK316980.1 --------------------------------------------------

NM_180251.3 --------------------------------------------------

JF968115.1 --------------------------------------------------

JF968117.1 --------------------------------------------------

JF968118.1 --------------------------------------------------

NM_001247584.2 --------------------------------------------------

KJ401124.1 --------------------------------------------------

XM_004252354.4 --------------------------------------------------

XM_002266972.5 --------------------------------------------------

XM_024596482.1 --------------------------------------------------

XM_006369031.3 --------------------------------------------------

XM_008341964.3 --------------------------------------------------

NM_001294046.1 --------------------------------------------------

NM_001251371.2 --------------------------------------------------

XM_006604868.4 --------------------------------------------------

NM_001354284.1 --------------------------------------------------

NM_001349033.1 --------------------------------------------------

NM_001155962.1 --------------------------------------------------

NM_001157201.2 --------------------------------------------------

AK111690.1 --------------------------------------------------

XM_008660266.4 --------------------------------------------------

XM_015788266.2 --------------------------------------------------

NM_001156774.2 --------------------------------------------------

NM_001155219.2 --------------------------------------------------

EU837255.1 --------------------------------------------------

XM_009403037.2 --------------------------------------------------

XM_018823871.1 --------------------------------------------------

XM_009419716.2 --------------------------------------------------

XM_009419259.2 --------------------------------------------------

XM_009415720.2 --------------------------------------------------

XM_009411978.2 --------------------------------------------------

XM_009383333.2 --------------------------------------------------

XM_009381868.2 --------------------------------------------------

XM_009405824.2 --------------------------------------------------

XM_009389352.2 --------------------------------------------------

XM_009391309.2 --------------------------------------------------

NM_001084343.2 --------------------------------------------------

NM_001159194.1 --------------------------------------------------

XM_015789203.1 --------------------------------------------------

XM_015774497.2 --------------------------------------------------

NM_001153709.2 --------------------------------------------------

NM_001254770.2 --------------------------------------------------

NM_001150750.2 --------------------------------------------------

CM007650.1_142530856-142531634 --------------------------------------------------

CM007648.1_220493755-220494609 --------------------------------------------------

NM_001111800.2 --------------------------------------------------

XM_023301819.1 --------------------------------------------------

XM_015775299.2 --------------------------------------------------

XM_009384249.2 --------------------------------------------------

XM_009385622.2 --------------------------------------------------

XM_009388210.2 --------------------------------------------------

XM_009389661.2 --------------------------------------------------

XM_009393219.2 --------------------------------------------------

XM_009415859.2 --------------------------------------------------

XM_008649541.3 --------------------------------------------------

XM_015782909.2 --------------------------------------------------

NM_001349820.1 --------------------------------------------------

EU837258.1 --------------------------------------------------

EU847517.1 --------------------------------------------------

XM_015775100.2 --------------------------------------------------

CT833260.1 --------------------------------------------------

AK067060.1 --------------------------------------------------

XM_015795858.2 --------------------------------------------------

EU847519.1 --------------------------------------------------

FR720457.1 --------------------------------------------------

EU847520.1 --------------------------------------------------

NM_001143039.1 --------------------------------------------------

AK073133.1 --------------------------------------------------

XM_006380178.2 --------------------------------------------------

XM_002315454.3 --------------------------------------------------

XM_008392395.2 --------------------------------------------------

XM_029095961.1 --------------------------------------------------

XM_002272390.4 TTCATGAAGTGAGAAATAGAATAAAAAAGCGTTTTTTGATAATTTATTTT

NM_001256464.1 --------------------------------------------------

XM_003546320.5 --------------------------------------------------

NM_001279196.2 --------------------------------------------------

AY035100.1 --------------------------------------------------

JF968116.1 --------------------------------------------------

JF968119.1 --------------------------------------------------

NM_130320.4 --------------------------------------------------

NM_001248198.2 --------------------------------------------------

AY192368.1 --------------------------------------------------

NM_001247379.2 --------------------------------------------------

JX145122.1 --------------------------------------------------

XM_002301454.4 --------------------------------------------------

XM_002320960.3 --------------------------------------------------

NM_001320016.1 --------------------------------------------------

NM_001328756.1 --------------------------------------------------

FQ392750.1 --------------------------------------------------

NM_001254494.3 --------------------------------------------------

NM_001254517.2 --------------------------------------------------

AK316980.1 --------------------------------------------------

NM_180251.3 --------------------------------------------------

JF968115.1 --------------------------------------------------

JF968117.1 --------------------------------------------------

JF968118.1 --------------------------------------------------

NM_001247584.2 --------------------------------------------------

KJ401124.1 --------------------------------------------------

XM_004252354.4 --------------------------------------------------

XM_002266972.5 ----------------------------AAGTCACAAAATTTAAACAAAA

XM_024596482.1 --------------------------------------------------

XM_006369031.3 --------------------------------------------------

XM_008341964.3 --------------------------------------------------

NM_001294046.1 --------------------------------------------------

NM_001251371.2 --------------------------------------------------

XM_006604868.4 --------------------------------------------------

NM_001354284.1 --------------------------------------------------

NM_001349033.1 --------------------------------------------------

NM_001155962.1 --------------------------------------------------

NM_001157201.2 --------------------------------------------------

AK111690.1 --------------------------------------------------

XM_008660266.4 --------------------------------------------------

XM_015788266.2 --------------------------------------------------

NM_001156774.2 --------------------------------------------------

NM_001155219.2 --------------------------------------------------

EU837255.1 --------------------------------------------------

XM_009403037.2 --------------------------------------------------

XM_018823871.1 --------------------------------------------------

XM_009419716.2 --------------------------------------------------

XM_009419259.2 --------------------------------------------------

XM_009415720.2 --------------------------------------------------

XM_009411978.2 --------------------------------------------------

XM_009383333.2 --------------------------------------------------

XM_009381868.2 --------------------------------------------------

XM_009405824.2 --------------------------------------------------

XM_009389352.2 --------------------------------------------------

XM_009391309.2 --------------------------------------------------

NM_001084343.2 --------------------------------------------------

NM_001159194.1 --------------------------------------------------

XM_015789203.1 --------------------------------------------------

XM_015774497.2 --------------------------------------------------

NM_001153709.2 --------------------------------------------------

NM_001254770.2 --------------------------------------------------

NM_001150750.2 --------------------------------------------------

CM007650.1_142530856-142531634 --------------------------------------------------

CM007648.1_220493755-220494609 --------------------------------------------------

NM_001111800.2 --------------------------------------------------

XM_023301819.1 --------------------------------------------------

XM_015775299.2 --------------------------------------------------

XM_009384249.2 --------------------------------------------------

XM_009385622.2 --------------------------------------------------

XM_009388210.2 --------------------------------------------------

XM_009389661.2 --------------------------------------------------

XM_009393219.2 --------------------------------------------------

XM_009415859.2 --------------------------------------------------

XM_008649541.3 --------------------------------------------------

XM_015782909.2 --------------------------------------------------

NM_001349820.1 --------------------------------------------------

EU837258.1 --------------------------------------------------

EU847517.1 --------------------------------------------------

XM_015775100.2 --------------------------------------------------

CT833260.1 --------------------------------------------------

AK067060.1 --------------------------------------------------

XM_015795858.2 --------------------------------------------------

EU847519.1 --------------------------------------------------

FR720457.1 --------------------------------------------------

EU847520.1 --------------------------------------------------

NM_001143039.1 --------------------------------------------------

AK073133.1 --------------------------------------------------

XM_006380178.2 --------------------------------------------------

XM_002315454.3 --------------------------------------------------

XM_008392395.2 --------------------------------------------------

XM_029095961.1 --------------------------------------------------

XM_002272390.4 TAATAACAATTTTCAAAAGCTCTAAATGTCTAAATGATCCCGAGAGTTGG

NM_001256464.1 --------------------------------------------------

XM_003546320.5 --------------------------------------------------

NM_001279196.2 --------------------------------------------------

AY035100.1 --------------------------------------------------

JF968116.1 --------------------------------------------------

JF968119.1 --------------------------------------------------

NM_130320.4 --------------------------------------------------

NM_001248198.2 --------------------------------------------------

AY192368.1 --------------------------------------------------

NM_001247379.2 --------------------------------------------------

JX145122.1 --------------------------------------------------

XM_002301454.4 --------------------------------------------------

XM_002320960.3 --------------------------------------------------

NM_001320016.1 --------------------------------------------------

NM_001328756.1 --------------------------------------------------

FQ392750.1 --------------------------------------------------

NM_001254494.3 --------------------------------------------------

NM_001254517.2 --------------------------------------------------

AK316980.1 --------------------------------------------------

NM_180251.3 --------------------------------------------------

JF968115.1 --------------------------------------------------

JF968117.1 --------------------------------------------------

JF968118.1 --------------------------------------------------

NM_001247584.2 --------------------------------------------------

KJ401124.1 --------------------------------------------------

XM_004252354.4 --------------------------------------------------

XM_002266972.5 CAAGGTGAGTATGGAAAATTTTTAATTGGGACAAGGGTGAAAATGGGTTT

XM_024596482.1 --------------------------------------------------

XM_006369031.3 --------------------------------------------------

XM_008341964.3 --------------------------------------------------

NM_001294046.1 --------------------------------------------------

NM_001251371.2 --------------------------------------------------

XM_006604868.4 --------------------------------------------------

NM_001354284.1 --------------------------------------------------

NM_001349033.1 --------------------------------------------------

NM_001155962.1 --------------------------------------------------

NM_001157201.2 --------------------------------------------------

AK111690.1 --------------------------------------------------

XM_008660266.4 --------------------------------------------------

XM_015788266.2 --------------------------------------------------

NM_001156774.2 --------------------------------------------------

NM_001155219.2 --------------------------------------------------

EU837255.1 --------------------------------------------------

XM_009403037.2 --------------------------------------------------

XM_018823871.1 --------------------------------------------------

XM_009419716.2 --------------------------------------------------

XM_009419259.2 --------------------------------------------------

XM_009415720.2 --------------------------------------------------

XM_009411978.2 --------------------------------------------------

XM_009383333.2 --------------------------------------------------

XM_009381868.2 --------------------------------------------------

XM_009405824.2 --------------------------------------------------

XM_009389352.2 --------------------------------------------------

XM_009391309.2 --------------------------------------------------

NM_001084343.2 --------------------------------------------------

NM_001159194.1 --------------------------------------------------

XM_015789203.1 --------------------------------------------------

XM_015774497.2 --------------------------------------------------

NM_001153709.2 --------------------------------------------------

NM_001254770.2 --------------------------------------------------

NM_001150750.2 --------------------------------------------------

CM007650.1_142530856-142531634 --------------------------------------------------

CM007648.1_220493755-220494609 --------------------------------------------------

NM_001111800.2 --------------------------------------------------

XM_023301819.1 --------------------------------------------------

XM_015775299.2 --------------------------------------------------

XM_009384249.2 --------------------------------------------------

XM_009385622.2 --------------------------------------------------

XM_009388210.2 --------------------------------------------------

XM_009389661.2 --------------------------------------------------

XM_009393219.2 --------------------------------------------------

XM_009415859.2 --------------------------------------------------

XM_008649541.3 --------------------------------------------------

XM_015782909.2 --------------------------------------------------

NM_001349820.1 --------------------------------------------------

EU837258.1 --------------------------------------------------

EU847517.1 --------------------------------------------------

XM_015775100.2 --------------------------------------------------

CT833260.1 --------------------------------------------------

AK067060.1 --------------------------------------------------

XM_015795858.2 --------------------------------------------------

EU847519.1 --------------------------------------------------

FR720457.1 --------------------------------------------------

EU847520.1 --------------------------------------------------

NM_001143039.1 --------------------------------------------------

AK073133.1 --------------------------------------------------

XM_006380178.2 --------------------------------------------------

XM_002315454.3 --------------------------------------------------

XM_008392395.2 --------------------------------------------------

XM_029095961.1 --------------------------------------------------

XM_002272390.4 GGATCGCTGGATTTATTGAATTTTCAAAAAAAGGGCATACCCTCGTTGAT

NM_001256464.1 --------------------------------------------------

XM_003546320.5 --------------------------------------------------

NM_001279196.2 --------------------------------------------------

AY035100.1 --------------------------------------------------

JF968116.1 --------------------------------------------------

JF968119.1 --------------------------------------------------

NM_130320.4 --------------------------------------------------

NM_001248198.2 --------------------------------------------------

AY192368.1 --------------------------------------------------

NM_001247379.2 --------------------------------------------------

JX145122.1 --------------------------------------------------

XM_002301454.4 --------------------------------------------------

XM_002320960.3 --------------------------------------------------

NM_001320016.1 --------------------------------------------------

NM_001328756.1 --------------------------------------------------

FQ392750.1 --------------------------------------------------

NM_001254494.3 --------------------------------------------------

NM_001254517.2 --------------------------------------------------

AK316980.1 --------------------------------------------------

NM_180251.3 --------------------------------------------------

JF968115.1 --------------------------------------------------

JF968117.1 --------------------------------------------------

JF968118.1 --------------------------------------------------

NM_001247584.2 --------------------------------------------------

KJ401124.1 --------------------------------------------------

XM_004252354.4 --------------------------------------------------

XM_002266972.5 TTACATAGGATGAAGCTATGATGAGGTGTCTACTCCTATGTTTGGTTGAA

XM_024596482.1 --------------------------------------------------

XM_006369031.3 --------------------------------------------------

XM_008341964.3 --------------------------------------------------

NM_001294046.1 --------------------------------------------------

NM_001251371.2 --------------------------------------------------

XM_006604868.4 --------------------------------------------------

NM_001354284.1 --------------------------------------------------

NM_001349033.1 --------------------------------------------------

NM_001155962.1 --------------------------------------------------

NM_001157201.2 --------------------------------------------------

AK111690.1 --------------------------------------------------

XM_008660266.4 --------------------------------------------------

XM_015788266.2 --------------------------------------------------

NM_001156774.2 --------------------------------------------------

NM_001155219.2 --------------------------------------------------

EU837255.1 --------------------------------------------------

XM_009403037.2 --------------------------------------------------

XM_018823871.1 --------------------------------------------------

XM_009419716.2 --------------------------------------------------

XM_009419259.2 --------------------------------------------------

XM_009415720.2 --------------------------------------------------

XM_009411978.2 --------------------------------------------------

XM_009383333.2 --------------------------------------------------

XM_009381868.2 --------------------------------------------------

XM_009405824.2 --------------------------------------------------

XM_009389352.2 --------------------------------------------------

XM_009391309.2 --------------------------------------------------

NM_001084343.2 --------------------------------------------------

NM_001159194.1 --------------------------------------------------

XM_015789203.1 --------------------------------------------------

XM_015774497.2 --------------------------------------------------

NM_001153709.2 --------------------------------------------------

NM_001254770.2 --------------------------------------------------

NM_001150750.2 --------------------------------------------------

CM007650.1_142530856-142531634 --------------------------------------------------

CM007648.1_220493755-220494609 --------------------------------------------------

NM_001111800.2 --------------------------------------------------

XM_023301819.1 --------------------------------------------------

XM_015775299.2 --------------------------------------------------

XM_009384249.2 --------------------------------------------------

XM_009385622.2 --------------------------------------------------

XM_009388210.2 --------------------------------------------------

XM_009389661.2 --------------------------------------------------

XM_009393219.2 --------------------------------------------------

XM_009415859.2 --------------------------------------------------

XM_008649541.3 --------------------------------------------------

XM_015782909.2 --------------------------------------------------

NM_001349820.1 --------------------------------------------------

EU837258.1 --------------------------------------------------

EU847517.1 --------------------------------------------------

XM_015775100.2 --------------------------------------------------

CT833260.1 --------------------------------------------------

AK067060.1 --------------------------------------------------

XM_015795858.2 --------------------------------------------------

EU847519.1 --------------------------------------------------

FR720457.1 --------------------------------------------------

EU847520.1 --------------------------------------------------

NM_001143039.1 --------------------------------------------------

AK073133.1 --------------------------------------------------

XM_006380178.2 --------------------------------------------------

XM_002315454.3 --------------------------------------------------

XM_008392395.2 --------------------------------------------------

XM_029095961.1 --------------------------------------------------

XM_002272390.4 TCCAAACGAGGAGAAATGAAGGAGGGAATGGCAGGATTGAGATATGAGGA

NM_001256464.1 --------------------------------------------------

XM_003546320.5 --------------------------------------------------

NM_001279196.2 --------------------------------------------------

AY035100.1 --------------------------------------------------

JF968116.1 --------------------------------------------------

JF968119.1 --------------------------------------------------

NM_130320.4 --------------------------------------------------

NM_001248198.2 --------------------------------------------------

AY192368.1 --------------------------------------------------

NM_001247379.2 --------------------------------------------------

JX145122.1 --------------------------------------------------

XM_002301454.4 --------------------------------------------------

XM_002320960.3 --------------------------------------------------

NM_001320016.1 --------------------------------------------------

NM_001328756.1 --------------------------------------------------

FQ392750.1 --------------------------------------------------

NM_001254494.3 --------------------------------------------------

NM_001254517.2 --------------------------------------------------

AK316980.1 --------------------------------------------------

NM_180251.3 --------------------------------------------------

JF968115.1 --------------------------------------------------

JF968117.1 --------------------------------------------------

JF968118.1 --------------------------------------------------

NM_001247584.2 --------------------------------------------------

KJ401124.1 --------------------------------------------------

XM_004252354.4 --------------------------------------------------

XM_002266972.5 ATAAAAATAAATATTCATAGGAAATTTCAAGACACTCTCCTAACCTATTT

XM_024596482.1 --------------------------------------------------

XM_006369031.3 --------------------------------------------------

XM_008341964.3 -------------------------------------------GTTTTCT

NM_001294046.1 --------------------------------------------------

NM_001251371.2 --------------------------------------------------

XM_006604868.4 --------------------------------------------------

NM_001354284.1 --------------------------------------------------

NM_001349033.1 --------------------------------------------------

NM_001155962.1 --------------------------------------------------

NM_001157201.2 --------------------------------------------------

AK111690.1 --------------------------------------------------

XM_008660266.4 --------------------------------------------------

XM_015788266.2 --------------------------------------------------

NM_001156774.2 --------------------------------------------------

NM_001155219.2 --------------------------------------------------

EU837255.1 --------------------------------------------------

XM_009403037.2 --------------------------------------------------

XM_018823871.1 --------------------------------------------------

XM_009419716.2 --------------------------------------------------

XM_009419259.2 --------------------------------------------------

XM_009415720.2 --------------------------------------------------

XM_009411978.2 --------------------------------------------------

XM_009383333.2 --------------------------------------------------

XM_009381868.2 --------------------------------------------------

XM_009405824.2 --------------------------------------------------

XM_009389352.2 --------------------------------------------------

XM_009391309.2 --------------------------------------------------

NM_001084343.2 --------------------------------------------------

NM_001159194.1 --------------------------------------------------

XM_015789203.1 --------------------------------------------------

XM_015774497.2 --------------------------------------------------

NM_001153709.2 --------------------------------------------------

NM_001254770.2 --------------------------------------------------

NM_001150750.2 --------------------------------------------------

CM007650.1_142530856-142531634 --------------------------------------------------

CM007648.1_220493755-220494609 --------------------------------------------------

NM_001111800.2 --------------------------------------------------

XM_023301819.1 --------------------------------------------------

XM_015775299.2 --------------------------------------------------

XM_009384249.2 --------------------------------------------------

XM_009385622.2 --------------------------------------------------

XM_009388210.2 --------------------------------------------------

XM_009389661.2 --------------------------------------------------

XM_009393219.2 --------------------------------------------------

XM_009415859.2 --------------------------------------------------

XM_008649541.3 --------------------------------------------------

XM_015782909.2 --------------------------------------------------

NM_001349820.1 --------------------------------------------------

EU837258.1 --------------------------------------------------

EU847517.1 --------------------------------------------------

XM_015775100.2 --------------------------------------------------

CT833260.1 --------------------------------------------------

AK067060.1 --------------------------------------------------

XM_015795858.2 --------------------------------------------------

EU847519.1 --------------------------------------------------

FR720457.1 --------------------------------------------------

EU847520.1 --------------------------------------------------

NM_001143039.1 --------------------------------------------------

AK073133.1 --------------------------------------------------

XM_006380178.2 --------------------------------------------------

XM_002315454.3 --------------------------------------------------

XM_008392395.2 --------------------------------------------------

XM_029095961.1 --------------------------------------------------

XM_002272390.4 AAACAAGAAGGTCATATAGTAGGAGTAGCATTCCAAGGTGCCAACAACTG

NM_001256464.1 --------------------------------------------------

XM_003546320.5 --------------------------------------------------

NM_001279196.2 --------------------------------------------------

AY035100.1 --------------------------------------------------

JF968116.1 --------------------------------------------------

JF968119.1 --------------------------------------------------

NM_130320.4 --------------------------------------------------

NM_001248198.2 --------------------------------------------------

AY192368.1 --------------------------------------------------

NM_001247379.2 --------------------------------------------------

JX145122.1 --------------------------------------------------

XM_002301454.4 --------------------------------------------------

XM_002320960.3 --------------------------------------------------

NM_001320016.1 --------------------------------------------------

NM_001328756.1 --------------------------------------------------

FQ392750.1 --------------------------------------------------

NM_001254494.3 --------------------------------------------------

NM_001254517.2 --------------------------------------------------

AK316980.1 --------------------------------------------------

NM_180251.3 --------------------------------------------------

JF968115.1 --------------------------------------------------

JF968117.1 --------------------------------------------------

JF968118.1 --------------------------------------------------

NM_001247584.2 --------------------------------------------------

KJ401124.1 --------------------------------------------------

XM_004252354.4 --------------------------------------------------

XM_002266972.5 ATCTTCATCTTAAACTTACGTATTAAATACGAATGTGTCTCTTAAACAAC

XM_024596482.1 --------------------------------------------------

XM_006369031.3 --------------------------------------------------

XM_008341964.3 TATTATTTTTCTTGGTCGGCAAAAGCAAGCAGTAAAATTCAACCACCACA

NM_001294046.1 --------------------------------------------------

NM_001251371.2 --------------------------------------------------

XM_006604868.4 --------------------------------------------------

NM_001354284.1 --------------------------------------------------

NM_001349033.1 --------------------------------------------------

NM_001155962.1 --------------------------------------------------

NM_001157201.2 --------------------------------------------------

AK111690.1 --------------------------------------------------

XM_008660266.4 --------------------------------------------------

XM_015788266.2 --------------------------------------------------

NM_001156774.2 --------------------------------------------------

NM_001155219.2 --------------------------------------------------

EU837255.1 --------------------------------------------------

XM_009403037.2 --------------------------------------------------

XM_018823871.1 --------------------------------------------------

XM_009419716.2 --------------------------------------------------

XM_009419259.2 --------------------------------------------------

XM_009415720.2 --------------------------------------------------

XM_009411978.2 --------------------------------------------------

XM_009383333.2 --------------------------------------------------

XM_009381868.2 --------------------------------------------------

XM_009405824.2 --------------------------------------------------

XM_009389352.2 --------------------------------------------------

XM_009391309.2 --------------------------------------------------

NM_001084343.2 --------------------------------------------------

NM_001159194.1 --------------------------------------------------

XM_015789203.1 --------------------------------------------------

XM_015774497.2 --------------------------------------------------

NM_001153709.2 --------------------------------------------------

NM_001254770.2 --------------------------------------------------

NM_001150750.2 --------------------------------------------------

CM007650.1_142530856-142531634 --------------------------------------------------

CM007648.1_220493755-220494609 --------------------------------------------------

NM_001111800.2 --------------------------------------------------

XM_023301819.1 --------------------------------------------------

XM_015775299.2 --------------------------------------------------

XM_009384249.2 --------------------------------------------------

XM_009385622.2 --------------------------------------------------

XM_009388210.2 --------------------------------------------------

XM_009389661.2 --------------------------------------------------

XM_009393219.2 --------------------------------------------------

XM_009415859.2 --------------------------------------------------

XM_008649541.3 --------------------------------------------------

XM_015782909.2 --------------------------------------------------

NM_001349820.1 --------------------------------------------------

EU837258.1 --------------------------------------------------

EU847517.1 --------------------------------------------------

XM_015775100.2 --------------------------------------------------

CT833260.1 --------------------------------------------------

AK067060.1 --------------------------------------------------

XM_015795858.2 --------------------------------------------------

EU847519.1 --------------------------------------------------

FR720457.1 --------------------------------------------------

EU847520.1 --------------------------------------------------

NM_001143039.1 --------------------------------------------------

AK073133.1 --------------------------------------------------

XM_006380178.2 --------------------------------------------------

XM_002315454.3 --------------------------------------------------

XM_008392395.2 --------------------------------------------------

XM_029095961.1 --------------------------------------------------

XM_002272390.4 CGAGTCCCTCCCTCCACTGGGCCGTTAAATTTTAGAACTGCTATGACGCG

NM_001256464.1 --------------------------------------------------

XM_003546320.5 --------------------------------------------------

NM_001279196.2 --------------------------------------------------

AY035100.1 --------------------------------------------------

JF968116.1 --------------------------------------------------

JF968119.1 --------------------------------------------------

NM_130320.4 --------------------------------------------------

NM_001248198.2 --------------------------------------------------

AY192368.1 --------------------------------------------------

NM_001247379.2 --------------------------------------------------

JX145122.1 --------------------------------------------------

XM_002301454.4 --------------------------------------------------

XM_002320960.3 --------------------------------------------------

NM_001320016.1 --------------------------------------------------

NM_001328756.1 --------------------------------------------------

FQ392750.1 --------------------------------------------------

NM_001254494.3 --------------------------------------------------

NM_001254517.2 --------------------------------------------------

AK316980.1 --------------------------------------------------

NM_180251.3 -----ATATAATAAGCACTAAACTGTAAAATTATATTCAGCCACCCAAAC

JF968115.1 --------------------------------------------------

JF968117.1 --------------------------------------------------

JF968118.1 --------------------------------------------------

NM_001247584.2 --------------------------------------------------

KJ401124.1 --------------------------------------------------

XM_004252354.4 --------------------------------------------------

XM_002266972.5 CAAACATGGTCTTAAGAATTGGTTTTTAACGTAAGAAAAGGGCAAAAGTT

XM_024596482.1 --------------------------------------------------

XM_006369031.3 --------------------------------------------------

XM_008341964.3 GAAGGGCATTCTGGGCAACAAAAGCAGCGGCATCTGCAAATCTCCACATT

NM_001294046.1 --------------------------------------------------

NM_001251371.2 --------------------------------------------------

XM_006604868.4 --------------------------------------------------

NM_001354284.1 --------------------------------------------------

NM_001349033.1 --------------------------------------------------

NM_001155962.1 --------------------------------------------------

NM_001157201.2 --------------------------------------------------

AK111690.1 --------------------------------------------------

XM_008660266.4 -----------------------------------------------AAG

XM_015788266.2 --------------------------------------------------

NM_001156774.2 --------------------------------------------------

NM_001155219.2 --------------------------------------------------

EU837255.1 --------------------------------------------------

XM_009403037.2 --------------------------------------------------

XM_018823871.1 --------------------------------------------------

XM_009419716.2 --------------------------------------------------

XM_009419259.2 --------------------------------------------------

XM_009415720.2 --------------------------------------------------

XM_009411978.2 --------------------------------------------------

XM_009383333.2 --------------------------------------------------

XM_009381868.2 --------------------------------------------------

XM_009405824.2 --------------------------------------------------

XM_009389352.2 --------------------------------------------------

XM_009391309.2 --------------------------------------------------

NM_001084343.2 --------------------------------------------------

NM_001159194.1 --------------------------------------------------

XM_015789203.1 --------------------------------------------------

XM_015774497.2 --------------------------------------------------

NM_001153709.2 --------------------------------------------------

NM_001254770.2 --------------------------------------------------

NM_001150750.2 --------------------------------------------------

CM007650.1_142530856-142531634 --------------------------------------------------

CM007648.1_220493755-220494609 --------------------------------------------------

NM_001111800.2 --------------------------------------------------

XM_023301819.1 --------------------------------------------------

XM_015775299.2 --------------------------------------------------

XM_009384249.2 --------------------------------------------------

XM_009385622.2 --------------------------------------------------

XM_009388210.2 --------------------------------------------------

XM_009389661.2 --------------------------------------------------

XM_009393219.2 --------------------------------------------------

XM_009415859.2 --------------------------------------------------

XM_008649541.3 --------------------------------------------------

XM_015782909.2 --------------------------------------------------

NM_001349820.1 --------------------------------------------------

EU837258.1 --------------------------------------------------

EU847517.1 --------------------------------------------------

XM_015775100.2 -------------------------CATCCTCCCACCCACGACCCTTGAG

CT833260.1 --------------------------------------------------

AK067060.1 --------------------------------------------------

XM_015795858.2 --------------------------------------------------

EU847519.1 --------------------------------------------------

FR720457.1 --------------------------------------------------

EU847520.1 --------------------------------------------------

NM_001143039.1 --------------------------------------------------

AK073133.1 --------------------------------------------------

XM_006380178.2 --------------------------------------------------

XM_002315454.3 --------------------------------------------------

XM_008392395.2 --------------------------------------------------

XM_029095961.1 --------------------------------------------------

XM_002272390.4 CGTTACAAGTTACAACAGCTGCCCACACTTACGCTTCATCTAGACAGTGC

NM_001256464.1 --------------------------------------------------

XM_003546320.5 --------------------------------------------------

NM_001279196.2 --------------------------------------------------

AY035100.1 --------------------------------------------------

JF968116.1 --------------------------------------------------

JF968119.1 --------------------------------------------------

NM_130320.4 --------------------------------------------------

NM_001248198.2 --------------------------------------------------

AY192368.1 --------------------------------------------------

NM_001247379.2 --------------------------------------------------

JX145122.1 --------------------------------------------------

XM_002301454.4 --------------------------------------------------

XM_002320960.3 --------------------------------------------------

NM_001320016.1 --------------------------------------------------

NM_001328756.1 --------------------------------------------------

FQ392750.1 --------------------------------------------------

NM_001254494.3 --------------------------------------------------

NM_001254517.2 --------------------------------------------------

AK316980.1 --------------------------------------------------

NM_180251.3 CATGACAAATCACCTTAAAGGCTTAAACACATAACAGCCATTACGAGTCA

JF968115.1 --------------------------------------------------

JF968117.1 --------------------------------------------------

JF968118.1 --------------------------------------------------

NM_001247584.2 --------------------------------------------------

KJ401124.1 --------------------------------------------------

XM_004252354.4 --------------------------------------------------

XM_002266972.5 GGAAAAATTTAAATTATTTCATCTCAATGCCAAAAAGATTTTCATCCTGG

XM_024596482.1 --------------------------------------------------

XM_006369031.3 --------------------------------------------------

XM_008341964.3 AACCAAGGACAAAATGGTAAACTCACCATCCGTCGTCGGCCATTTCCCCG

NM_001294046.1 --------------------------------------------------

NM_001251371.2 --------------------------------------------------

XM_006604868.4 --------------------------------------------------

NM_001354284.1 --------------------------------------------------

NM_001349033.1 --------------------------------------------------

NM_001155962.1 --------------------------------------------------

NM_001157201.2 --------------------------------------------------

AK111690.1 --------------------------------------------------

XM_008660266.4 TCACTCACTCACTCCTCACGCGTCTACTCTACTACTTAACACGCGCCCGT

XM_015788266.2 --------------------------------------------------

NM_001156774.2 --------------------------------------------------

NM_001155219.2 --------------------------------------------------

EU837255.1 --------------------------------------------------

XM_009403037.2 --------------------------------------------------

XM_018823871.1 --------------------------------------------------

XM_009419716.2 --------------------------------------------------

XM_009419259.2 --------------------------------------------------

XM_009415720.2 --------------------------------------------------

XM_009411978.2 --------------------------------------------------

XM_009383333.2 --------------------------------------------------

XM_009381868.2 --------------------------------------------------

XM_009405824.2 --------------------------------------------------

XM_009389352.2 -----------------------------------------CTAAACCAC

XM_009391309.2 --------------------------------------------------

NM_001084343.2 --------------------------------------------------

NM_001159194.1 --------------------------------------------------

XM_015789203.1 --------------------------------------------------

XM_015774497.2 --------------------------------------------------

NM_001153709.2 --------------------------------------------------

NM_001254770.2 --------------------------------------------------

NM_001150750.2 --------------------------------------------------

CM007650.1_142530856-142531634 --------------------------------------------------

CM007648.1_220493755-220494609 --------------------------------------------------

NM_001111800.2 --------------------------------------------------

XM_023301819.1 --------------------------------------------------

XM_015775299.2 --------------------------------------------------

XM_009384249.2 --------------------------------------------------

XM_009385622.2 --------------------------------------------------

XM_009388210.2 --------------------------------------------------

XM_009389661.2 --------------------------------------------------

XM_009393219.2 --------------------------------------------------

XM_009415859.2 --------------------------------------------------

XM_008649541.3 --------------------------------------------------

XM_015782909.2 --------------------------------------------------

NM_001349820.1 --------------------------------------------------

EU837258.1 --------------------------------------------------

EU847517.1 --------------------------------------------------

XM_015775100.2 CGCCTCGCAACGTCGTCGGCTCGGCTCGACTCGTCTCGTCGCCCACGAAT

CT833260.1 --------------------------------------------------

AK067060.1 --------------------------------------------------

XM_015795858.2 --------------------------------------------------

EU847519.1 --------------------------------------------------

FR720457.1 --------------------------------------------------

EU847520.1 --------------------------------------------------

NM_001143039.1 --------------------------------------------------

AK073133.1 --------------------------------------------------

XM_006380178.2 --------------------------------------------------

XM_002315454.3 --------------------------------------------------

XM_008392395.2 --------------------------------------------------

XM_029095961.1 --------------------------------------------------

XM_002272390.4 TGTCACCCAGCCTCCCCACCCCTACGCGCCACGTCCTCACCCCGCTTCCG

NM_001256464.1 --------------------------------------------------

XM_003546320.5 --------------------------------------------------

NM_001279196.2 --------------------------------------------------

AY035100.1 --------------------------------------------------

JF968116.1 --------------------------------------------------

JF968119.1 --------------------------------------------------

NM_130320.4 --------------------------------------------------

NM_001248198.2 --------------------------------------------------

AY192368.1 --------------------------------------------------

NM_001247379.2 --------------------------------------------------

JX145122.1 --------------------------------------------------

XM_002301454.4 --------------------------------------------------

XM_002320960.3 --------------------------------------------------

NM_001320016.1 --------------------------------------------------

NM_001328756.1 --------------------------------------------------

FQ392750.1 --------------------------------------------------

NM_001254494.3 -------------------------------------GGCAAAGTAAAAG

NM_001254517.2 --------------------------------------------------

AK316980.1 --------------------------------------------------

NM_180251.3 CAGGTAAGGGTATAATAGTAAAGAATCAATCTATATAATATACGACCCAC

JF968115.1 --------------------------------------------------

JF968117.1 --------------------------------------------------

JF968118.1 --------------------------------------------------

NM_001247584.2 --------------------------------------------------

KJ401124.1 --------------------------------------------------

XM_004252354.4 --------------------------------------------------

XM_002266972.5 TAAATTAGAGTTGGAGGTAGGGGTAATGTGGTAAAACCGTAGCACAGCTA

XM_024596482.1 --------------------------------------------CAACCA

XM_006369031.3 --------------------------------------------------

XM_008341964.3 TAAGAAAAAATATAAAACGATAAACCCTCATTTTGTTCAGCAGAAAAATA

NM_001294046.1 ---------------------------------------GGAGAGAACTA

NM_001251371.2 --------------------------------------------------

XM_006604868.4 --------------------------------------------------

NM_001354284.1 -------------------------------------------AAACTCA

NM_001349033.1 -----------------------------------CTTACACCAAACTCA

NM_001155962.1 ------AACCTCGCCTCCCCCCACTCCTTCCCTCCCTCCCTCGCTCACCC

NM_001157201.2 -------------------------CACTCCCTCGCTCACCAGAGGAACT

AK111690.1 --------------------------------------------------

XM_008660266.4 GCCATCCGCCCACTCCTCTCCCACGCACTCCGCAATTCCGCATCACGCAG

XM_015788266.2 --------------------------------------------------

NM_001156774.2 --------------------------------------------------

NM_001155219.2 --------------------------------------------------

EU837255.1 --------------------------------------------------

XM_009403037.2 --------------------------------------------------

XM_018823871.1 --------------------------------------------------

XM_009419716.2 --------------------------------------------------

XM_009419259.2 --------------------------------------------------

XM_009415720.2 --------------------------------------------------

XM_009411978.2 --------------------------------------------------

XM_009383333.2 ----------CTACGTATCGTCATGATCACGTGCCTCTTTCCACTTCGCC

XM_009381868.2 --------------------------------------------------

XM_009405824.2 --------------------------------------------------

XM_009389352.2 CATTAAAGTACTTCCCAACTTTCCCATTTGCAATTACTTTTATCTCGTCC

XM_009391309.2 --------------------------------------------------

NM_001084343.2 --------------------------------------------------

NM_001159194.1 --------------------------------------------------

XM_015789203.1 --------------------------------------------------

XM_015774497.2 --------------------------------------------------

NM_001153709.2 --------------------------------------------------

NM_001254770.2 --------------------------------------------------

NM_001150750.2 --------------------------------------------------

CM007650.1_142530856-142531634 --------------------------------------------------

CM007648.1_220493755-220494609 --------------------------------------------------

NM_001111800.2 --------------------------------------------------

XM_023301819.1 --------------------------------------------------

XM_015775299.2 --------------------------------------------------

XM_009384249.2 ---------------------------------------------ACTGG

XM_009385622.2 --------------------------------------------------

XM_009388210.2 --------------------------------------------------

XM_009389661.2 --------------------------------------------------

XM_009393219.2 --------------------------------------------------

XM_009415859.2 --------------------------------------------------

XM_008649541.3 --------------------------------------------------

XM_015782909.2 --------------------------------------------------

NM_001349820.1 --------------------------------------------------

EU837258.1 --------------------------------------------------

EU847517.1 --------------------------------------------------

XM_015775100.2 TTTGGAGGGTCGCGTACTGAGCTCGTGGCCCGCGTGACCATTTCTTTTAT

CT833260.1 --------------------------------------------------

AK067060.1 --------------------------------------------------

XM_015795858.2 --------------------------------------------------

EU847519.1 --------------------------------------------------

FR720457.1 --------------------------------------------------

EU847520.1 --------------------------------------------------

NM_001143039.1 --------------------------------------------------

AK073133.1 --------------------------------------------------

XM_006380178.2 --------------------------------------------------

XM_002315454.3 --------------------------------------------------

XM_008392395.2 --------------------------------------------------

XM_029095961.1 --------------------------------------------------

XM_002272390.4 TCACCAAACGCACCACTCCCGTACCGTCACCCTCATTTCACCTTCGTATT

NM_001256464.1 --------------------------------------------------

XM_003546320.5 --------------------------------------------------

NM_001279196.2 --------------------------------------------------

AY035100.1 --------------------------------------------------

JF968116.1 --------------------------------------------------

JF968119.1 --------------------------------------------------

NM_130320.4 --------------------------------------------------

NM_001248198.2 --------------------------------------------------

AY192368.1 --------------------------------------------------

NM_001247379.2 --------------------------------------------------

JX145122.1 --------------------------------------------------

XM_002301454.4 --------------------------------------------------

XM_002320960.3 --------------------------------------------TAGGAA

NM_001320016.1 --------------------------------------------------

NM_001328756.1 --------------------------------------------------

FQ392750.1 --------------------------------------------------

NM_001254494.3 TAAAAACAAAAACAAAAGCGAATTGTAGGAAGGAAGGTTCCCAATGTATC

NM_001254517.2 --------------------------------------------------

AK316980.1 --------------------------------------------------

NM_180251.3 CCTTTCTCATTCTTTCTGGAGAGTAACATCGAGACAAAGAAGAAAAACTA

JF968115.1 ------------------------------------------AATTATTG

JF968117.1 -----------------------------------------GAATTATTA

JF968118.1 --------------------------------------------------

NM_001247584.2 ----------------------GCTTGATTCCTTCTGAACTTTGTAAAAC

KJ401124.1 --------------------------------------------------

XM_004252354.4 --------------------------------------------------

XM_002266972.5 CTTTAATATAAAATCCCTAACCTTTTGTTTAGAACTCATTTTTCGGAAGA

XM_024596482.1 TCTCTACCTAAATC-CCAATAATCGCTACTTACAAATTATTCTCTCTTGG

XM_006369031.3 -CTCCAACTAACTCTCCACTCACCGAACCCCAAAAATCATTTTCAC---A

XM_008341964.3 ACGAAAGCTTTTCTCTCCCTCTCTCTGA---TCGCTTGCTTTACTTCCAA

NM_001294046.1 ACGAAAGCTTTTCTATATCTCTCTCTCTCTCTCTCTCGATTCTCTTTCAA

NM_001251371.2 --------------------------------------------------

XM_006604868.4 --------------------------------------------------

NM_001354284.1 ATTATACAAAACCCTGAAGAAAGACACAGCCATTGTTTGTTTACTTTCCA

NM_001349033.1 ATTATACAAAACCCTGAAGAAAGACACAGCCATTGTTT----ACTTTTCA

NM_001155962.1 AGTGGGGCAGCCAAAGCCGAGCCGCA-----ACCGCAAAGATCTTGCGCG

NM_001157201.2 AGCGGACCAGGCAAAGCCGAGCCGCACCGCAACCGCGAAGATCTTGCGTG

AK111690.1 ---------------------------------------GGGCTTCCTCT

XM_008660266.4 AGGCAGAGCGACCAACCCAGAACCCCACCCCACCGCCCGCAACCGCAAGC

XM_015788266.2 ----------------------ATCCTCCTCACTCTCTGTAAAAACAAGC

NM_001156774.2 --CTGTGCCAGTGGCGGCCAGACAATAAATAGGGCCCACACCCCGTCCGC

NM_001155219.2 --------------------------------------------------

EU837255.1 --------------------------------------------------

XM_009403037.2 ---TAAGATCAATCACATGGCAGGTAGATTGTGTGGTTGGTTGATGTGTG

XM_018823871.1 --------------------------------------------------

XM_009419716.2 --------------------------------------------------

XM_009419259.2 -----------------------------------------------ACT

XM_009415720.2 --------------------------------------------GAAGAA

XM_009411978.2 ---------------------------TCTCTCTCTCTCTCTCTA-TTAA

XM_009383333.2 CTTTCTGCCTACCATTAGGAGCGTTTGTTCACGTCCATCTCCCCAGTCGA

XM_009381868.2 ----------------------------------------------TACT

XM_009405824.2 --------------------------------------------------

XM_009389352.2 CTACTCGTCCACCAAAATCCGATCTTTCCAACCCAACCCCGTTGGGTATA

XM_009391309.2 --------------------------------------------------

NM_001084343.2 --------------------------------------------------

NM_001159194.1 --------------------------------------------------

XM_015789203.1 --------------------------------------------------

XM_015774497.2 --------------------------------------------------

NM_001153709.2 -----------------------------------------------ATC

NM_001254770.2 -------------------------------------ATCATCGTCCATC

NM_001150750.2 -AGTTCGAAAGGTTCAACTTCAAACACACTCCAAACTCCAAGCCTCAATA

CM007650.1_142530856-142531634 --------------------------------------------------

CM007648.1_220493755-220494609 --------------------------------------------------

NM_001111800.2 --------------------------------------------------

XM_023301819.1 --------------------------------------------------

XM_015775299.2 --------------------------------------------------

XM_009384249.2 TCAACAGCCCAGATCGCCACTTAAACTGGTCCACGCGCAATCTAACCATT

XM_009385622.2 --------------------------------------------------

XM_009388210.2 --------------------------------------------------

XM_009389661.2 --------------------------------------------------

XM_009393219.2 --------------------------------------------------

XM_009415859.2 --------------------------------------------------

XM_008649541.3 --------------------------------------------------

XM_015782909.2 --------------------------------------------------

NM_001349820.1 --------------------------------------------------

EU837258.1 --------------------------------------------------

EU847517.1 --------------------------------------------------

XM_015775100.2 TTTCTCCCCCTACTTTTCCCGCGCTGCTTTCCCCTCGCGCCATCCAAGCG

CT833260.1 --------------------------------------------------

AK067060.1 --------------------------------------------------

XM_015795858.2 -------------CCACCATGACTGCAAAATTTCCAAGTTTCTCGCTAGT

EU847519.1 --------------------------------------------------

FR720457.1 --------------------------------------------------

EU847520.1 --------------------------------------------------

NM_001143039.1 --------------------------------------------------

AK073133.1 --------------------------------------------------

XM_006380178.2 --------------------------------------------------

XM_002315454.3 --------------------------------------------------

XM_008392395.2 --------------------------------------------------

XM_029095961.1 --------------------------------------------------

XM_002272390.4 TGTATTAGCTCTCCTCATTGGCTCTCCACCGTCCGATCAGATGACTAGTG

NM_001256464.1 --------------------------------------------------

XM_003546320.5 --------------------------------------------------

NM_001279196.2 --------------------------------------------------

AY035100.1 --------------------------------------------------

JF968116.1 --------------------------------------------------

JF968119.1 --------------------------------------------------

NM_130320.4 --------------------------------------------------

NM_001248198.2 --------------------------------------------------

AY192368.1 --------------------------------------------------

NM_001247379.2 --------------------------------------------------

JX145122.1 --------------------------------------------------

XM_002301454.4 --------------------------------------------------

XM_002320960.3 GGCGATGCTGGTGGAAAGAAAGTTCCAAAGCCTCTCCACTTGTTCGTACT

NM_001320016.1 --------------------------------------------------

NM_001328756.1 --------------------------------------------------

FQ392750.1 --------------------------------------------------

NM_001254494.3 TGTCACGCACTCTTTCCCCATTTTTTCCTACTACATAAGCCACTCCACCC

NM_001254517.2 --------------------------------------------------

AK316980.1 AAAGATATAACCCCCAAAAGTATCAATTAGTTTCCATTTTCGCCGCTAAG

NM_180251.3 AAAAAGAGAACCCC--AAAGAATCGAATATTTATTATTT-CGCCCCGAAG

JF968115.1 TTTCCTCTCCAATCTTCCCAAATTATTCACTCCCATTTCCGAAG-GAATT

JF968117.1 TTTCCCTTCCAATCTACTACCATTG---------ATTTCCGAAGCAAATT

JF968118.1 ---------AAGCTGAACGAGATCCGTGTTTTCTGCTCTCA----GAATC

NM_001247584.2 TAAAATACATTTTCTCTTCAAATTGAGCTTTTTCTCCATTAAAATTCTCT

KJ401124.1 --------------------------------------------------

XM_004252354.4 ----------------------------TAGATTCTTCTCGAAATATCCG

XM_002266972.5 AAAACCCTGCAAATTCGCTCTTTTTTCGCTTCCAATTTCGAAATCGATTT

XM_024596482.1 AAGAAAAAGGAAAATCA--CCATCTAACCACTCTCTCA-AAAATCTTT--

XM_006369031.3 GAAGAAAAGGAAAATCA--CAATTTGACCACTTTCTCTCAAAAAACTC--

XM_008341964.3 AACACAAAAACCAGTCAGTGGATTCAACTTTTTCACCTGAAAAATCCCAA

NM_001294046.1 AGCACTAAAACCAGTC---GAATCAAACTTTTTTCTGTGAAAAATCCC--

NM_001251371.2 ---------------------------------------------ATTCA

XM_006604868.4 --------------------------------------------------

NM_001354284.1 TTGATTGAGCGAAAGCTTTGTTTTTTCTGCGATCATTTTCCATTGGGTGG

NM_001349033.1 TTGATTGAGCCAAAGCTTTGTATTTTCTGCGATAATTTTCCATTGGGTGG

NM_001155962.1 GCCTCCTTTCCCCAGCTCGTCACTGTGCCGCTCGTGCCATCCACCCTCGG

NM_001157201.2 GCCTCCTTTTCCCAGCTCGTCACTGTGCCGCTCGTGCCCTACACTCCCGG

AK111690.1 -CCCACTGCGCCCTCCGCGTGAGCG-GCAGCAAGTGTTCACTGCGTTCT-

XM_008660266.4 TCAGATTCCCTCCCCACCCCACCCCACC-CCACCGTCCCGCTCACTCCAG

XM_015788266.2 AAACAAAGAAAGAAAAGCATCACAAATC-TCACGCACTGTCTCTCGTTCG

NM_001156774.2 GTCCGCGCTCTCCAAAGTTTCGTCCACCATTTCGCTCCCGCAAGCACCGA

NM_001155219.2 -------------AAAGTTTCGTCCACCATTTCGCTCTCGCAAGTCGCAA

EU837255.1 GAAGAGAGAACTCGAACTCTCGCCTACCATTTCGCCCCCCT---CCGCAA

XM_009403037.2 CGAAAAGTCTTGGTTTGCTTCTCCCTCTTTAAG-AGAAGCGTTGGCTTCA

XM_018823871.1 -------------CTCTCGTCTCCAGTGTTGGCCAAAAGCAAGTTCTTGT

XM_009419716.2 -----------------------------------ATAACGGAGCCTTTT

XM_009419259.2 TTTCTCCTCTCTCTCTCTCTCTCTCTTGTTCTTGAAAGGAAGCTCTTTT-

XM_009415720.2 GGTTTGGCTGCTACTGGTCTCCCTGTTGTTCTAAAAAAGGAGCTCCTTTT

XM_009411978.2 GGAATAAGGTTTGTATTTTTGATCCATTTCTCCGCCAATTGGTGCTGTTG

XM_009383333.2 TCTCTATCCATTAGGCAAATGACCTCTTTGATTCGGTTTTCCTTCTATTT

XM_009381868.2 ATAAAAAACCCTTCTTCTTCAACTATGAGGAGAGAAGCAAAGAAGACGGG

XM_009405824.2 -----------------TGCAAC-ATGAGAAGGGAAGAGAAGAAGAAGAA

XM_009389352.2 AATGCCCCCCCGCCTCCCCACGTCTTTCTCCAAGAGGGAGACTTCACCTT

XM_009391309.2 ---------------GCCACCGCAACTGTCCGAGAAA-AGGCATTCTTTT

NM_001084343.2 --------------------------------------------------

NM_001159194.1 --------------------------------------------------

XM_015789203.1 --------------------------------------------------

XM_015774497.2 --------------------------------------------------

NM_001153709.2 GCCGCGACCTCGG-AGTACACGACCGCCATCTATTCTCCGCCACTTTTTG

NM_001254770.2 GCTGCGACCTGCG-ACCTCGGAACCA--ACCTTTCCGCCACT-CTCTCTC

NM_001150750.2 ATCTCGACCTCTGTAATGACCAAGAAGCTCATCTCCATCTCCACCATGGC

CM007650.1_142530856-142531634 ---------------------------------------------ATGCA

CM007648.1_220493755-220494609 --------------------------------------------------

NM_001111800.2 --------------------------------------CATTTCCATTCT

XM_023301819.1 --------------------------------------------------

XM_015775299.2 --------------------------------------------------

XM_009384249.2 CAATGCAATCCTTCGTTTCGCCAGTAGCGCCGCCATCAACGCGTCCATGT

XM_009385622.2 --------------------------------------------------

XM_009388210.2 --------------------------------------------------

XM_009389661.2 --------------------------------------------------

XM_009393219.2 --------------------------------------------------

XM_009415859.2 --------------------------------------------------

XM_008649541.3 --------------------------------------------------

XM_015782909.2 --------------------------------------------------

NM_001349820.1 --------------------------------CTCCTCTCTCCGCCGGCA

EU837258.1 ------------------AGGCATTCGCAACACACACTTGAAGAAAAAAA

EU847517.1 --------------------------------------------------

XM_015775100.2 CCCGCCTCTATATATGCGCGCCGCCACCATGTCCAGCTCCGGCAACTAGC

CT833260.1 ---------------------------------------------AAAAC

AK067060.1 ---------GACAAGCCATTAAACAGAGACGACCAACGACTTAACCACAC

XM_015795858.2 TTAAATGCACGCCGCTAGCTTAGCTACTCTCTTCCACCCACAACCCCCAA

EU847519.1 --------------------------------------------------

FR720457.1 --------------------------------------------------

EU847520.1 -------------------GCAAGGATAACTCAAACTACTTGAATCAGAT

NM_001143039.1 --------------------------------------------------

AK073133.1 --------------------------------------------------

XM_006380178.2 --------------------------------------------------

XM_002315454.3 --------------------------------------------------

XM_008392395.2 --------------------------------------------------

XM_029095961.1 --------------------------------------------------

XM_002272390.4 TGAAAGCTATCGCTGGTGTTTGTCGTGTTGCGTGTTGATTTCTTTTGTAA

NM_001256464.1 --------------------------------------------------

XM_003546320.5 --------------------------------------------------

NM_001279196.2 --------------------------------------------------

AY035100.1 --------------------------------------------------

JF968116.1 --------------------------------------------------

JF968119.1 ------------------------------------------------CA

NM_130320.4 -----------------------------------------AGGTTATAG

NM_001248198.2 --------------------------------------------------

AY192368.1 --------------------------------------------------

NM_001247379.2 -------------------CCAACCCCCCTCCCTCCACCACTACAAATAC

JX145122.1 --------------------------------------------------

XM_002301454.4 ----------------------------TATTACTCACTCATCCTTCAAC

XM_002320960.3 GCTGTCACCCACCTACCTCATTTCCTCACGCTACATA-TAATCCTTCCAT

NM_001320016.1 -------------------------------------------------C

NM_001328756.1 --------------------------------------------------

FQ392750.1 --------------------------------------------------

NM_001254494.3 CTTCTTTATTCCTCACGTCCTATCACTCTCAATTCTCAAACTTGTTACAG

NM_001254517.2 --------------ACGTCCTATCACTCTCAGACTT-----GTGTTACAA

AK316980.1 ATTCTGTTTTCGAACATTTACACCCTCAAGAA-----------TCG----

NM_180251.3 ATTCTATTTCTGATCATTTACACCCCTAAAAAGAGTAGAGCTTTCGTGAA

JF968115.1 GTGTTTCTGAAACTC-------AATCAATCAAGCTGTTCGAGAATTCTGA

JF968117.1 GTGTTTTTCCTGCTCTGCAAA-AATCAATCAAGTTGTTAGAAGGTTTTGA

JF968118.1 GAG-----GAAGATC-------AGCTAGTTGTGTGTTTTGATTCCCCTGA

NM_001247584.2 CTGCAAATTTATAGTTTTTCTTTTTTCACTTTTTGA-GAAGAAATCAAAA

KJ401124.1 --------------------------------------------------

XM_004252354.4 CAAAAGCAAATTTTGACAGTACTTTTCACTTCTCAC-CGGTACCGGAAAA

XM_002266972.5 CCATTTCTCAGCTTTCGAGCCCTCATAATCTCTCCCACAATCTGTGAGAA

XM_024596482.1 --TCAATTACAACCCCGTGAAGCAACCCACCTGTATAATCACCATCAATC

XM_006369031.3 --TCATTTACACCCCTGTGAAGCAAACCACCAGCATCATCAACCTCAAGC

XM_008341964.3 CTTTTTTAATTTTTCTGACACCCTTTTCAGCTGCAAGTCCTCCGTGAAAC

NM_001294046.1 ----------TTTTCCGATACCCTTTTCTGCTGCAAGTCCGCTGTGAAAC

NM_001251371.2 ACCAAAACGGACGCTTTTGTTATTCTTCGTGTCTTGTTGTGT--TTTTCA

XM_006604868.4 --------------------------------------AGAA--AACCGG

NM_001354284.1 AAGAAAGTCTCAACCTTTAGTCGAAAGAGCAAGGATCTGAGT-GAGT-GA

NM_001349033.1 AAGAAAGTCTCAACCTTTATTCGAAAGAGCAAGGATCTGAGTTGAGTTGA

NM_001155962.1 TTCACCCAACCAACAATCCATCCG---------CCACCAC-ACCCCGCT-

NM_001157201.2 TCCGCCCAACCAATAATCCATCCATCCATCCATCCACCACCACCCCGCT-

AK111690.1 TCTTCTCGATTTATCTTTCTTGGTTTC-----TTGATCTGTAGCTTATTA

XM_008660266.4 CCCAGCCCGCGTCCCCACAGCCCAGCGACAGCG--GGCACCGGCGGC-AT

XM_015788266.2 CGCAAAGCACGCTGCTTTTCTCCGCT--TTGCG--AGCACCATAGCCTAG

NM_001156774.2 TTTGTTTACTGCTCTCTCCGACGCGCGGCGGCGGAGCTCCCTACGACGAC

NM_001155219.2 GCCACCGATTTGTTT-----ACA---GGCGCCGGAGCTCCCGACGACGAC

EU837255.1 GCAATCCACCACTGC-----ATC---GGCGGCG-AGGGCTCACCGGCGGC

XM_009403037.2 GTTGATGGTTGTT------TCTCTGAGGGGAGGAAGGAGCCGTTTCAGCT

XM_018823871.1 GATTTGGGTTCTTGT----TGTGTGGGAGGAAGGAAGAGTTGTGTACGGA

XM_009419716.2 GGCTGCTACAACTGC----TGCTTGGGGTGTTCTCAA---CGAGAAAGAA

XM_009419259.2 ----------------------GGAAGAGAAGCTGAGCGGGGTTTGAGGA

XM_009415720.2 TTGGAGTGCGTCGTCGAAAAAGGGTAGAAGAGTCGAGGGGAGTGTGAGGA

XM_009411978.2 GA--GTAGTCTTGAGAGAATCAGGAAGAGT-TCGAGGAGGGATTTGAGGG

XM_009383333.2 G---GCGTCCTCGAGAGAAGCAGGAAGAGTGTTGTGGGGGGATCTGAGGG

XM_009381868.2 GAGTCGGAAGAATTCCAAG------CTGTAAAGGAGCGATTTTTGAGGTT

XM_009405824.2 GAAGAAGTAGGAATAAAGGAGGGATTTTTAGGTTTGGGAAGGAGGGGGTT

XM_009389352.2 GAGTCGAAAAGAACCCATA------AAGTAGGAGTCTTGA-AGCGGTTGT

XM_009391309.2 GCTTTGCTTCGTTCTTGTG------CGAGAGGAGAGAAAAGAGGAGAAAC

NM_001084343.2 --------------------------------------------------

NM_001159194.1 ----------CGAGACACAGCAACCTCCCACTCCAC-TGTCCACTCCACC

XM_015789203.1 --------CCCACGGCTCCGCTCCCCCCTCCTCAAC-CAAGC-CCACGCG

XM_015774497.2 --TAAAGGCTGGATGGCGCGCATCCCCACCCTCACCTCGCTCTCCTCGTC

NM_001153709.2 GCGACACCGACGCCACGTCGTCGCACTCGAATCGGCAGAGCCACCA--CC

NM_001254770.2 GCGACACCGAC--CGACCTGACGACGTCGCACTCGCACAGCCACCA--C-

NM_001150750.2 CGGGAAGCAAGGTTGCAAGGA-GCAGTCCAATGATCGGAGGATCCAGGCT

CM007650.1_142530856-142531634 TGGGCAGAGAG---GCGTCGTCCCTCCTCGGCGCGGAGAGGGGCCA----

CM007648.1_220493755-220494609 ------------ATGATGCGCTCTCAT--TCGCTGACCTCCACTCCAAGC

NM_001111800.2 TCCAGTTGCACCCAGACCCAGGCAGGTCATCGGCGCCCACAACTCCAAGT

XM_023301819.1 ---------AGCGTCGGGCCGTCGGCT-ATTTCATACCGCCGAACCAAAT

XM_015775299.2 ----------------------CGCACGGTATATATACACACCCCCAGGT

XM_009384249.2 CTCGTATATAAGTAGACCCCCAACCACGGCCTCTTCTCCTCTGTCTATTG

XM_009385622.2 --------------------------------------------------

XM_009388210.2 --------------------------------------------------

XM_009389661.2 --------------------------------------------------

XM_009393219.2 --------------------------------------------------

XM_009415859.2 --------------------------------------------------

XM_008649541.3 -------------------------GCCCTGTGGCGCAGCACTGGTCACG

XM_015782909.2 --------------------------------------------------

NM_001349820.1 ACCTTATTGGTA--TCAAGAAAAACTTGTCGAAAAAT---------ACAC

EU837258.1 ACACGACGAACACGTTAAAAAAAGGTCGAAGAGAAGTGGGAGACCCAAAC

EU847517.1 -------------------------------AAGCAAAAGAAAAGCAGCA

XM_015775100.2 TACTGCGAAGTGCGAACTGATCAGATCGTCGAGAGGAAACCCAAGATCCA

CT833260.1 CCAATCAAACTCCAACCAAACTCACCTACCTACCCCCAACCCATCCAGAG

AK067060.1 TTCTTCTTTGTGCTGTCCAACCTCCATTGTTGGGTGTGAAAGCTTTGGCT

XM_015795858.2 GCATCAGCATCACAACCCACCAATCGAATCGATCGAACAAGACACGACAC

EU847519.1 --------ATCACAACC-ACCAATCGAATCGATCGAACAAGACACGACAC

FR720457.1 ----------------------------ATGTGTGGAGGAGAAGTGATCC

EU847520.1 CAGACAAAAACATCATAAGAATATCACGATGTGTGGAGGAGCACTGATCC

NM_001143039.1 --------GTGCGGTCTGCGGCCAAGCAGAGCCCGGGGAAACGATCGAG-

AK073133.1 ------------------------------GCCCGGAGAAAGAAGAGCGC

XM_006380178.2 --------------------------------------------------

XM_002315454.3 --------------------------------------ACTAGGCCTCGT

XM_008392395.2 ------------------------------GAGAGACCTCTCGTTTTGTG

XM_029095961.1 ----------------------------------GGCTTCTTGCTTTGTG

XM_002272390.4 CCGTCGTCGTTCTATAAATGAAGGCTCTCAGTGAAGCATCTTTCCTCTCT

NM_001256464.1 --------------------------------------------------

XM_003546320.5 -GCGTGTTATATTTCTCTCACTTCCTCACATTAATGTGTGTTTGTCACGA

NM_001279196.2 --------------------------------------------------

AY035100.1 --------------------------------------------------

JF968116.1 ---------------AGAAATCGACCC-TTCCGGAAAAC-----------

JF968119.1 ATTATTATAAAATTACAAGATCGACCCCTTCCGGAAAACACCAGGAAAAA

NM_130320.4 AGCACACAGTAAATAAAGTAGATGATATTAATAAAAGTTTCTAGAA----

NM_001248198.2 --------------------------------------------------

AY192368.1 --------------------------------------------------

NM_001247379.2 CTCCCCATTTCTCCCCACACCAA---AACCAAAACTGAAACTAAAA----

JX145122.1 -----GATTCACGTAGACACCAATCCATCCATCCCTTCCCATTACT----

XM_002301454.4 TAAAAAATCCTTCGT---AAATTCTTTTC-AACTCTTCTTCATCAT----

XM_002320960.3 TTTAGAACCCTTCTCTTAAAATCCTATTCGAAGTCGTCTTCATTAC----

NM_001320016.1 TTTCTCGCTCATCTCTCGCTCATCCCTTA-AACCCTTCTCTTTCTC----

NM_001328756.1 -----------TCTCTCCTCTCTCTCTT----TCTCTCTCTCTTCA----

FQ392750.1 -------------GCACAATCAACTGTTG-----CTTCTTCATTTC----

NM_001254494.3 ATAAAAACCCTTTCTTGAAACCTTT-CTCCAAAATTTCTCACAACC----

NM_001254517.2 ACAAAAACCCTTCTTCAAAACCTTTTCTCTAAAATTTCTCACAACC----

AK316980.1 -CCGCCATGTGTG-GAGGAGCTATAATATCCGATTTCATTCCACCG----

NM_180251.3 GCCACCATGTGTG-GAGGAGCTATAATCTCCGATTTCATACCTCCG----

JF968115.1 GTCA---TGTGTG-GTGGTGCGATCATCTCCGATTTTATACCGGCC----

JF968117.1 GCCA---TGTGTG-GTGGTGCGATTATCTCCGATTTCATACCGACA----

JF968118.1 GCTA---TGTGTG-GTGGTGCTATTATCTCCGATTTCATCCCGGCG----

NM_001247584.2 GCTA---TGTGTG-GTGGTGCAATTATCTCCGATTTGGTACCTCCT----

KJ401124.1 ATCA---TGTGTG-GTGGTTCTATAATCTCCGATTACATAGACCCT----

XM_004252354.4 AGCA---TGTGTG-GTGGTGCCATAATCTCCGATTGGATACCGCCG----

XM_002266972.5 ACGA---TGTGTG-GTGGTGCCATCATCTCCGACTTCATTCCGGCG----

XM_024596482.1 AACA---TGTGTG-GCGGTGCTATCATCTCAGACTTCATCGCTCCGACAA

XM_006369031.3 AGCAGCATGTGTG-GCGGTGCTATCATCTCTGACTTCATACCTCCGACCA

XM_008341964.3 AGCATAATGTGTG-GAGGTGCTATTATTTCCGATTTCATAGCGCCGGCG-

NM_001294046.1 AGCAAAATGTGTG-GAGGTGCTATTATTTCCGATTTCATAGCGCCG----

NM_001251371.2 TCTGAGATGTGTGGAGGA-GCTATCATCTCTGATTTCATTCCGGCGGCGG

XM_006604868.4 ACTGCGGCAAGGGCATAATAGTAACACTGCTCACTGATCTTCTCCATTTA

NM_001354284.1 GCGATCATGTGTG-GTGGTGCGATTATCTCCGACTTCATTCCAGCGGGTC

NM_001349033.1 GTGATCATGTGTG-GTGGTGCGATTATCTCCGACTTCATACCGGCAGGTC

NM_001155962.1 GCGGCGATGTGCG-GCGGCGCCATCCTCTCGGGTTTCATCCCGCCG----

NM_001157201.2 GCGGCGATGTGCG-GCGGCGCCATCCTCTCGGGTTTCATCCCGCCG----

AK111690.1 GCGGCCATGTGCG-GCGGAGCAATCATCTCCGGGTTCATCCCGCCG----

XM_008660266.4 CCAGCCATGTGCG-GCGGCGCCATCCTGTCGGACATCATCCCGC------

XM_015788266.2 CCCACCATGTGCG-GCGGCGCCATCCTCTCCGACCTCATCCCGC------

NM_001156774.2 TGAACCATGTGCG-GCGGCGCGATCCTTGCCAACCTTCGCGAGC------

NM_001155219.2 TGAGCCATGTGCG-GCGGCGCGATCCTTGCCGAGCTCCGCGAGC------

EU837255.1 -GAGCCATGTGCG-GCGGCGCCATCATCCACCACCTGAAGGGGC------

XM_009403037.2 TCGAGAATGTGCG-GAGGAGCGATCATCTCCGACTTCGTTCCG-------

XM_018823871.1 TCGAGGATGTGCG-GAGGGGCGATCATCTCCGACTTCATTCCG-------

XM_009419716.2 TCGAGGATGTGCG-GAGGAGCGATCGTCTCCGACTTCATTCCG-------

XM_009419259.2 TCGAGGATGTGCG-GAGGAGCGATCATCTCCGACTTCATTCCG-------

XM_009415720.2 TCGAAGATGTGCG-GAGGAGCGATCATTTCCGACCTCATTCCG-------

XM_009411978.2 TCGAGGATGTGTG-GAGGGGCGATCATCTCCGACTACATTCCG-------

XM_009383333.2 TCGAAGATGTGTG-GAGGGGCGATCATCTCCAACATCATTCCG-------

XM_009381868.2 CCAGAGATGTGTG-GGGGCGCGATCATCTCCGACTTCATTCCGCTG----

XM_009405824.2 TTAGAGATGTGTG-GAGGCGCCATCATCTCCGACTTCATTCCG-------

XM_009389352.2 GGAGAGATGTGTG-GAGGAGCGATCATCTCCGACCTCATACCG-------

XM_009391309.2 CGAGAGATGTGTG-GAGGAGCGATCATCTCCGAGTTCATACCG-------

NM_001084343.2 ---------CTATGATGAGGATAAAATGGTAAAATTAACAAAGCCCACTT

NM_001159194.1 CCCACCACCTTTGCC-------TCTCCTAGCTTTTCCTTG-GCAT-----

XM_015789203.1 CTCGCTCGCTTTGCAAGCAAAACGCCCCAACTCTCCCGCACGCAC-----

XM_015774497.2 ACCGCGATCGATCGATCGCCGCCGGTTTAGATTTCCTTCCTACGC-----

NM_001153709.2 ACCCCCCGCAATGACGTCGAG--------GCTGGAGAGCGGCGG------

NM_001254770.2 -CCCACCATGGCGGCGCCGAG--------GCTGGAGCGCGGCGG------

NM_001150750.2 TCGATCCAAGGAGACGCCAAGAGCGTGGTGGTGGGGTTCGGCGGC-----

CM007650.1_142530856-142531634 AAGGTGGAAGCAGCAGCAGGGAACGGCTTTCTCGGCCACAGTTA------

CM007648.1_220493755-220494609 TCTCTGTTTTCTTCGAGGA--ATCCATATGCGGCGGCGCAATCAT-----

NM_001111800.2 TGTATAAATACTCCGTCGACCGTCTCATAACAGTCATACCGCCGA-----

XM_023301819.1 CCAACGTCCGCAGAAACCAAACTCCCGTCGAGGAGGAGGCGTCGA-----

XM_015775299.2 CCGCCTCATACCG----------CCCATCCAAATCCAACGTCCGC-----

XM_009384249.2 CTTTACTCTGCATTCATCGACTCTCCTTTCCTATCGTTCACGAAA-----

XM_009385622.2 --------------------------------------CGTAAAG-----

XM_009388210.2 --------------------CCATCGACCGGGGTCAATCCCAAAC-----

XM_009389661.2 --------------------------------ATCTTTCGTCATT-----

XM_009393219.2 --------------------------------------------------

XM_009415859.2 -------------------------------GTGGGATCGGGGGA-----

XM_008649541.3 TCACACACGCACGCTCTATATACTATATAGGGAGGGGTTTGGCCT-----

XM_015782909.2 -----------AGCCAGCGACTGTGCCAATGTACTACTCCTACCC-----

NM_001349820.1 AGGGCACAATGTGCGGAGGCGCCATCCTCGCGGAGCTGATCCCGC-----

EU837258.1 CGCGAACAATGTGTGGAGGCGCCATCCTCGCCGAGTTCATCCCGG-----

EU847517.1 ACCA-AAGATGTGCGGCGGAGCGATCCTTGCGGAGCTCATACCGA-----

XM_015775100.2 ACGACGACATGTGTGGCGGCGCGATTCTGGCTAACATCATACCGG-----

CT833260.1 CTAGAGCTATGTGCGGCGGCGCGATCCTCGCCGACCTCATACCGT-----

AK067060.1 CATCTGCCATGTGTGGAGGATCCATTCTCGGCGACCTTCACTTGC-----

XM_015795858.2 CATGCGCC-GCCGCGTCTCCTCCTCCTCCTCCTCCTCCTCGTCCT-----

EU847519.1 CATGCGCC-GCCGCGTCTCCTCCTCCTCCTCCTCCTCCTCGTCCT-----

FR720457.1 CCGCCGAC-ATGCCGGCGGCGCCGTTCACGCCACG-CCACGGCGA-----

EU847520.1 CGAACGACTATGGCGACAA-GCCGCCGCCGCCGC--CGTCGGAGT-----

NM_001143039.1 ---ACGATATGTGCGGGGGAGCGATCCTTGCCGAACTCATCCCAG-----

AK073133.1 GCCGCGCCATGTGCGGCGGTGCAATCCTCGCCGATTTCACCCCGG-----

XM_006380178.2 AAGCCATATTTATCAGTGCATTTATTTCCTCTTTCTGCTTGA--------

XM_002315454.3 CCTCCTAATTTATTACACCATTTCCTCCTTCTCTTTCTCTGAA-------

XM_008392395.2 CGTGT--CCTGAGAGGGCTCTTTTTTCTCTCTAAGTTTCTAC--------

XM_029095961.1 CGTGTGTCATGAGAAAGCTCTTTTTTCTCTCAAAGTTTCTAC--------

XM_002272390.4 CATCTGCATTTATGGAGTGCTTCTCTTTGGCGTAGTTGCTACTTG-----

NM_001256464.1 --------------------------------------------------

XM_003546320.5 GACCCATAGTGCTGCCAAATTATAGTAACTCTCTTTCTCTATCTG-----

NM_001279196.2 ------------------------CTTTCACTCAGAAAACAAAAG-----

AY035100.1 --------------------------------------------------

JF968116.1 --TCCATTTCCACAAA-CCTGCAGATAAGAAGTAGCTGAAGATCC-----

JF968119.1 ACTCCATTTTCGCAAAACCTGCAGA-AATCAGTAGTGGAGGATCA-----

NM_130320.4 -----GAACTTGGTCGTCAAGCAAAAACAGCAACAACTCTGACATTGTCT

NM_001248198.2 --------------------------------------------------

AY192368.1 -----GAAAACTTTTCTATACATTTTTCATTTCTGTATCAATCAATATTC

NM_001247379.2 -----CAAAACTTTTCTATACATTTTTCATTTCTGTATCAATCAATATTC

JX145122.1 -----ACTCACTTTGATACCAACCAACCGACCACGACCAGAAGAAGAAGA

XM_002301454.4 -----AAACCCTC------GTCTACTTTACTTCCCTATCAACAGAT-C-T

XM_002320960.3 -----TCCCTCTTCCAT-TGTCTACTTTCCTTTACTATCAACAGAT-T-T

NM_001320016.1 -----TCTCTCTC--------CCCCCCCATCTCAATTT--TCAGTT-CAT

NM_001328756.1 -----CATCTTTC--------TCCTTCAATTTTCAGTTCATCAAAT-C-T

FQ392750.1 -----TACTCTTC--------CACTGCTGTTTTTTTTTTTTTCGTT-CCA

NM_001254494.3 -----CACTTCTACTCT--ACTCATTCCACACCCAACTTACAAAAA-CCC

NM_001254517.2 -----CATTTCTACTCTCTACTCATTCCACACCCAACTCACAAAAAACCC

AK316980.1 -----CCGAGGTCT---CGCCGTGTTACTAGCGAGTTTATTTGG------

NM_180251.3 -----CCGAGGTCC---CTCCGCGTCACTAACGAGTTTATCTGG------

JF968115.1 -----AAGGCCTCCGGCCGCCTTACCGCTACTGAGCTTTGGTCCGGCATC

JF968117.1 -----AAGGGTTCCGGCCGCCTGACTGCCGGCGAACTCTGGTCCGGTATC

JF968118.1 -----AAGGGATCCCGGCGCCTCGTCGCCAGCGATCTGTGGTCGGGTCTC

NM_001247584.2 -----AGCCGGATTTCTCGCCGGTTAACCGCTGATTTTCTATGGGGTACA

KJ401124.1 -----AGCCGGACTTCTCGCCGGCTCACCGCCGAGTTTCTATGGGGTCGT

XM_004252354.4 -----TCTCGATCTTCGAGCCGACTCACCGCCGACCAGTTATGGGGTTGC

XM_002266972.5 -----AGCCG---CTGCCGCCGGGTCGACGAGGATTACTT---------C

XM_024596482.1 CCACCGCTCGATCTTCTCGGCGGTTGACCTCGGGCTTTGAGTGG------

XM_006369031.3 CCACCGCTCGATCTTCTCGGCGGTTTGCGGAGGGATTCGAGTGG------

XM_008341964.3 -----GCACGGTCC---CGGCGGCTGACCGCCGACTACCTCTGG------

NM_001294046.1 -----GTACGGTCC---CGCCGGCTCACGGCCGACTACCTCTGG------

NM_001251371.2 CGATCGCCGGGTCT---CGCCGCCTGACCGCCGATTACCTGTGG------

XM_006604868.4 CCGACATCAGAGAA---TACAGAATAGCCTTCACTTGCATTCAA------

NM_001354284.1 CCGCCGGCGGGGCG---CAGCGCGTGACCGCCGACATCCTGTGG------

NM_001349033.1 CCGCCAGCGGGGCG---CGGCGCGTGACCGCCGACATCCTGTGG------

NM_001155962.1 --TC---CGGGGTGCCGGCGGCGGCAGCGGCGGCCAAGAAGAAA------

NM_001157201.2 --TC---CGGGGTGGCGGCGGCGGCGGCGGCGGCCAAGAAGCAG------

AK111690.1 --TCGGCCGCTGCGGCGGCGGCGGCTGCGGTGGCCAAGAAGCAG------

XM_008660266.4 ---CGCC---GCCACCGCGGCGGGTCACGGCTGGCCACCTCTGG------

XM_015788266.2 ---CGC---------CGCGGCGGGTCACCGCCGGCGACCTCTGG------

NM_001156774.2 ---C------GGCGCCGCGCCGGCTCACAGAGCGGGACATCTGG------

NM_001155219.2 ---C------GGCGCCGCGCCGGCTCACGGAGCGGGACATCTGG------

EU837255.1 ---ACCCGGAGGGGTCGCGCCGGGCGACGGAGGGGCTCCTGTGG------

XM_009403037.2 --GCTGCGAGGTCCTGGCGGCTCGTCTCCGAAGACGTTCTGTGG------

XM_018823871.1 --GCGGCGAGGTCGCGGCTGGTGACCGACGCCGACCTCCTGTGG------

XM_009419716.2 --ACAGCCAGGTCGCGGCGGGTCACCGACGCCGACCTTCTGTGG------

XM_009419259.2 --GCGACAAGGTCGGCGCGGCGGGTCACCGCCGACTACTTGTGG------

XM_009415720.2 --GCAACGAGGTCGGCGCGGCGGGTCACCGCGGACTACCTGTGG------

XM_009411978.2 --GCCGGGGCGGGGCCGCTGCGCGTGACCACGGATTGCCTGTGG------

XM_009383333.2 --GCGGGCCCGAGGTCGCGACGGCGGACCGCCGGCTACCAGCGG------

XM_009381868.2 --GCGACGGCGGCGTCGCAGCGGGTGATGGCGGAGCACCTGTGG------

XM_009405824.2 -----------GCGTCGCGGCGAGTGACCGCGGAGCAACTGTGG------

XM_009389352.2 -----GCCACGGTGGCGCGGCGGGTGACGGCGGAGCACCTGTGG------

XM_009391309.2 -----GCGGCAGCGTCGCGGCGGGTGACGGCGGAGAATCCGTGG------

NM_001084343.2 GGCCCAATTGTCGTTCCGAAGAACACGCCTTGTCGCTCTGCGTATAAGAG

NM_001159194.1 -----CCTAGCTCTTCCTCC-------TCAGC------CGCAGCAGATCG

XM_015789203.1 -----ACGCGCCCTTCCACCG-ACTCGTCAGCTTAGAGCACCGCAAGCGC

XM_015774497.2 --GGACCGCACCCACGCGCTA-GGT--TTAGC--------TAGCTAGTTT

NM_001153709.2 -GTTCCAGCTCCCGAACACCG-AGCAGGAGAACGCACTCTTGCTCCGCGC

NM_001254770.2 -GTTCCAGCTCCCGAACACCG-AGCAGGAGAACTCCCTCTTCCTCCGCGC

NM_001150750.2 AGGCTGGTCACCCGTGAGCAGGAGCAGGAGGACGCCATCATCGTCGCGGC

CM007650.1_142530856-142531634 --CTCCGCGGCCCG--CGCCG-ATTACGACGTCGCGGTCATGGCCGCGGC

CM007648.1_220493755-220494609 CTTC----GACTACATCCCGGCATGGCGCCGGGTGT-CGACTGCCGACTG

NM_001111800.2 ACCA----AATCCAACGTCCACAGAAAAGAAAACACATTCCCGTCGACTC

XM_023301819.1 CTTCCCATAGTTTCCACTCCACTTCGATCCTATCGTATCCTCCCCAAACA

XM_015775299.2 CTAAAGAAAAACACGCACACACTTCTTCTGCTTCCCTCCCCTTGTTTCCG

XM_009384249.2 GC-AGCGGCCTTATTCCTTCTTTCGACACTCGTTCCCTTCTCATAAAAT-

XM_009385622.2 AC-CCCACGATTTGCCTTCTCCGCTTCTCTTCATTACTACGAGTGGGTA-

XM_009388210.2 TC-ACCACCGTCGCTTTCTGCAGCGTCACTGCTAAAATATCTATCGGCTC

XM_009389661.2 TCCACCACCATCTCTTCTCTCTTTTGTTTACGTCAGATCTCCCAGAACTC

XM_009393219.2 -------------------------------GGAGAGGCAGAGGTGGGGT

XM_009415859.2 AACGGTGAAGTGTTGCAACGGCCTTGCTTTGAGGTTCCCATTTCTCTGAT

XM_008649541.3 CTGGTGGGAGCGCCGGCCGGCACCAAAACCACGCGCTACTACACCAGCCT

XM_015782909.2 CCTCTCCTACTCCTGTTAACAAGTTGTGCTGCTATATAGAGGAGCAGCCG

NM_001349820.1 CGACGCGGCGCGTGGCGTCG------AAGCCGGTGACAGAAGGCCACCTC

EU837258.1 CGCCGTCGCGCGCCGCGGCGGCGACCAAGCGGGTGACCGCCAGCCACCTG

EU847517.1 GCGCGCCGG------CGGCG------AGGCGCGTCACGGCGGGCCACGTC

XM_015775100.2 CCACGCCGC------CGC---------GGCCCGCCACGGCGGCGCATGTG

CT833260.1 CGCCGCGCTC-----CGGCG-------GCCACACCAAAAAGAACAAGCGG

AK067060.1 CGGTGCGGCGGACAGTGAACGCCGG-TGACCTGTGGGGAGACGCCGGCAA

XM_015795858.2 CGTCGCCGGC----GAGGCA-----TCACAA-GGCGCGGCGCAGCAGGAG

EU847519.1 CGTCGCCGGC----GAGGCA-----TCACAA-GGCGCGGCGCAGCAGGAG

FR720457.1 CGGCGA-GAC----ATGGGT-----TGACAG-AAAGAGGAGGAACAAGAA

EU847520.1 CGTCGG-AGT----GGGACG-----CCACAACGAAGATGAAGAAGAAGAA

NM_001143039.1 CACGGGTGCAC-CGGCCGCTGACCGCCGCCACGCTCTGGGCGGCGGCCCT

AK073133.1 CGAGGGTGCCC-CGGCGGCTGACCGCCGCCGAGCTCCTGCCGGTGACCCC

XM_006380178.2 -GAATTGGACAGA----------CAAAAACATGTGTGGAGGTGCCATTAT

XM_002315454.3 -GAACTGGACAGA----------CTAAAACATGTGTGGAGGTGCCATCAT

XM_008392395.2 --AAGTGGCAA------------TATAAACATGTGTGGTGGTGCTATCAT

XM_029095961.1 --AAGAGGCAA------------CATAAACATGTGTGGTGGTGCTATCAT

XM_002272390.4 CTAGGTGTTTAATTGCTTTCTTTTTTCAAGATGTGTGGAGGTGCAATTAT

NM_001256464.1 ------------------------------ATGTGTGGAGGTGCTATCAT

XM_003546320.5 AAGAAGAAGAAGTT------CTGAAGAAGCATGTGTGGAGGTGCTATCAT

NM_001279196.2 AAAAAAAAAGAGTT---------AGAAAAGATGTGTGGAGGTGCCATAAT

AY035100.1 GAAAGAAACCCACAGCAACAAACAGAGAAAATGTGTGGCGGTGCTATTAT

JF968116.1 AGAACAATCCCCGT--TGAATTCATCAATCATGTGTGGAGGCGCGATTAT

JF968119.1 AGAACAATCGTATTA-CGATTTCATCA-TCATGTGTGGAGGAGCGATTAT

NM_130320.4 CACTATATAAAACCCAAATTCTCTTTCTCTTTTAGGACTCGGTTCAAAAA

NM_001248198.2 --------------------------------------------------

AY192368.1 TTTGTTT------C-TGCTGTTTTGAGTAAA-CACACT----AAGATGTG

NM_001247379.2 TTTGTTT------C-TGCTGTTTTGAGTAAATCACACT----AAGATGTG

JX145122.1 AGAATCT------CATAGAGCCTTCCAGAAG--AAGCA----AGTATGTG

XM_002301454.4 CTCATA------GTTTTATTGTTTAGCTAATTAAAGA-----AATATGTG

XM_002320960.3 CTCTTATTATTTATTTATTTTTTTATCTAATTGAAGA-----AAGATGTG

NM_001320016.1 CTCGGAGTTAATTCA-ACAAGTTT-TTCGATTTCGAG-----AA--TGTG

NM_001328756.1 CTGAGAGTTATTTAACACAAGTTTGTTCGACTTCGAG-----AA--TGTG

FQ392750.1 TTTCTATCTTGTGATCG-AAGTCATTCTGAAGACTGA-----AGAATGTG

NM_001254494.3 ATTCTTT----TGCTCACTCATTTTCTTAACGTAGATTAATCATTATGTG

NM_001254517.2 ATTCTTTCTTTTGCTCACTCATTTTCTTAA-------------TCATGTG

AK316980.1 CCGGATCTG---AAGAAGAATTTGAAAGGATCGAAGAAAAGCTCGAAGAA

NM_180251.3 CCGGATCTG---AAAAACAAAGTGAAAGCTTCAAAGAAGAGATCGAATAA

JF968115.1 TCCGGCGAGGTCACGAAGAAGAAGGGGCTATCA------------AAGCC

JF968117.1 CCAAGCGAGGTCACGAGGCAGAAGGGGTTCTCT------------AAGCC

JF968118.1 GCTGGAGAAGGAAAGAAGAAGAAGAGATCATCTGGTGGATACTCGAAGCC

NM_001247584.2 TCCGATCTGAACAAGAAGAAGAAGAACCCTAGTAATTACCACTCAAAGCC

KJ401124.1 TTCGATCTCGGTAAGAAGCAAAAAAATCCCAACAATTATCACTCTAAAGC

XM_004252354.4 GCCGATCTGCAAAACAAGAAGAGGAACAAGAAGAAGAGGAATCCTTCCAA

XM_002266972.5 CCCGCCCT------CAAAAAACGCACCTCCGGCAACCACTGTTCCAAGTC

XM_024596482.1 CTTGAGCT------GAAGAAACCCTT---CAACAACAAGCACTTGAAGCC

XM_006369031.3 TTTGGTAT------GAAGAAACCCTTAGACAACAAGAAGTACTCGAAGCC

XM_008341964.3 CCCGATCT------CAAAAAACCCAGTTCGGGAAAGCGGTTCTCGAAGCC

NM_001294046.1 CCCGATCT------CAAAAAACCCAGTTCTGGGAAGCGGCTCTCGAAGCC

NM_001251371.2 CCGGATTT------GAAGAAGCGGAA--------GTCTGACTTGGACGTT

XM_006604868.4 CCAAAACT------GACG---CTTTT--------GTTATTCTTCGAGTCT

NM_001354284.1 CCGAATTT------GAGGAAGCGGTTC--TCGAAGTCGCTGCTGGACGAT

NM_001349033.1 CCGAGTTT------GAGGAAGCGCTTC--TCGAAGCCGCTGCTGGACGAT

NM_001155962.1 CAGCAGCGGC------------GTGCGACGGCGGACCTGCTGTGGCCGGG

NM_001157201.2 CAGCAGCAGCAGCAGCGGTGCCGTGTGACTGCGGACCTGCTATGGCCGGG

AK111690.1 CAG-GGCAGGA-----------GGGTCACGGCCGACGTGCTGTGGCCGGG

XM_008660266.4 CCCGAGAG---CAAGAAGCCG-------AGGAGGGCTGCATCCG--GCAG

XM_015788266.2 CTGGAGAAGACCAAGAAGCAGCAGCAGCAGAAGAAGAAGAACAA--GGGC

NM_001156774.2 CAGCAGAAA---AAGAAGCTC-------AAGAGGGGCGGCGGCG--GCGG

NM_001155219.2 CAGCAGAAG---AAGAAGCCC-------AAGAGGGGCGGCGCCG--GCGG

EU837255.1 CCCGAGAAG---AAGAAGCCC-------AGGTGGGGCGGCGGCG--GGAG

XM_009403037.2 CCGGATCTCAAGAA---CGGGTCGAAGAAGAAGAAGAAGAAGAAGAAGAA

XM_018823871.1 CCGAATC---------------CGAAGAAGGCCAAGTCGACGAAGAAGAA

XM_009419716.2 CCGAATC---------------TGAAGAAGGGTGGGTCGAAGAAGAAGAG

XM_009419259.2 CCGGATCTGAGGACGGGCGGAGCCAATGGGGCGACGAAGAAGCAGAAGAA

XM_009415720.2 CCGGATCTGAAGAAGGGGGGAGCCAGTGGGGC---GAAGAAGAAGAGGAG

XM_009411978.2 CCAGATCCGAACAATGGCTGCGGAATGAAGGCGGGGGAGAAGAAGAAGAA

XM_009383333.2 CCGGACCCGAAGAATGAATTTGGAGTGAAGGAGTGGGAGAAGCCGAAGGA

XM_009381868.2 CCGGGCCTCGAGAA------GGGTAAGCAGACGACGAGGAGGCGTCGGAG

XM_009405824.2 GTGGGCCGCGAGAA------GGGCGAGCTGAAGAAGGGGAGACATTGGGA

XM_009389352.2 CCGGGCGGGAGGAG------GAGGAGGGGGAAGCAGCAGAGAA---GGGA

XM_009391309.2 CCGGGCGGGAGGAA------GAGGTGGGGGAAACAGCGGAGGG---TGGA

NM_001084343.2 TGTGCTCAGGCAAAAATGAAAGAACAAAAACACAGAGACACCTTTAAAAT

NM_001159194.1 AC-----TAGGAAACATGTGC-GGCGGCGCGATC-----ATCTCCGAGTT

XM_015789203.1 GCAGCAGCAGCAAAGATGTGT-GGCGGCGCGATC-----ATTTCCGACTT

XM_015774497.2 GC-----TTGCGCGCAAGCGTCGATCGAGCTAGC------TAGTGAAGAT

NM_001153709.2 GCTCATCTCCGTCGTGTCCGGTGACACCGCCGCCGCGTCGTTGGTCCCGG

NM_001254770.2 GCTCATCTCCGTCGTGTCCGGGGACACCGCCGC------GTTGCTCCCGG

NM_001150750.2 GCTGCGGCACGTGGTGTCCGGGTACAGCAC---------GCCGCCGCCGG

CM007650.1_142530856-142531634 GCTCACGCACGTCGTCTGCG-CCACTGAGCCACC-----ACCGCCGCGCG

CM007648.1_220493755-220494609 CCG-ACTTCTGGCCTGGTT-----TTGAAGCAGACG---GCGAGGACATT

NM_001111800.2 CCCTAGTTTCATCCTATTCATTTCTCCCCCCAAACA---CTCCAGCTACT

XM_023301819.1 CTCCACCAAGAACCTTTTC--CCCCTCCAATAATAG---TCCAAGTTACT

XM_015775299.2 CTCCACTTCCCTTCCAAGCA---AGAAAACCGAGGC---TTCAGCTTAGC

XM_009384249.2 -TCCATCTTCCAAGCAAGCT----AAGTAGGAAAAG---CATAGCAGCCA

XM_009385622.2 -TCCATCTTCCGAT----CT----CAGAAGCAGCAG---CATAGCAGCAA

XM_009388210.2 ATTCATTCCTCTTCTCATAC----AATTGCCATCTC---CTAAAACTCGA

XM_009389661.2 ATACGTCGTCTCGGACTATA----ATCTTCCAAAAG---CAGTAGCGGAA

XM_009393219.2 TGGGGGACGGTGCACCG----GAGGAACAAGGTTCG---CCGGCGAAGAG

XM_009415859.2 CCGAGGCTTTTGGTCCGTCCCACAGATCCAGTTGCG---CCGGTTCAGGG

XM_008649541.3 AGCTAGCCCTTTCGCCGTCGCTGCGGCTCCTGCCCA---CTGACTCGTAG

XM_015782909.2 AGCAAGTGTGTTGCTAG--AAGAAAAGCCTTGTGAG---TTGGTAATTGA

NM_001349820.1 TGGTCG----GCGAGCTCCAAG--AAAGCCGGCAGCG--GCAGGGACAAG

EU837258.1 TGGCCG----GCCGGCTCCAAG--AACGCCGCCCGCG--GCAAGAGCAAG

EU847517.1 TGGCCG----GGCGACGCCAAC--AAGGCCA--AGAA--GAAGGGCGCGC

XM_015775100.2 TGGCCC----GGCGGCG---AC--GGGGAGA--AGCG--GCGGAAGGTTG

CT833260.1 CGGCGG----ATCAGCGACGAC--GAGGACT-TCGAG--GCCGCCTTCGA

AK067060.1 GGGTAGAGATGGTGGCGACGGCTTGAAGAAGAGGAAG--GGGAGTTCTTG

XM_015795858.2 GAAG------CTCGCCGTCGAC--GAGGACT-GGGAG--GCCGCCTTCCG

EU847519.1 GAAG------CTCGCCGTCGAC--GAGGACT-GGGAG--GCCGCCTTCCG

FR720457.1 GAAGAGGAAGCGCGGCGCCGAC--GAAGAAT-GGGAG--GCCGCCTTCCA

EU847520.1 GAAGCGTGGTGGCGGCGGCGAC--GACGACT-GGGAG--GCCGCCTTCCG

NM_001143039.1 GAGT------------GGGACCACTACCGTCGGCAAG--TGGAAGGCCGA

AK073133.1 GACTCCCCCCGCCGCCGAGAGGAGAACCACCCGGAAG--CGCAAGTCCGA

XM_006380178.2 TTCCGATTTTGTTACTGTT------AAG--CGTG-GCCGGAGATTGACAG

XM_002315454.3 TTCCGATTTCGTATCCGTT------AAA--CGTG-GCCGGAAACCGACCA

XM_008392395.2 TTCCGACTTCATCGCCGTC------AAG--CGCG-CCCTGAAGCTGACGG

XM_029095961.1 TTCCGACTTCATCGCCGCC------AAG--CGTG-GCCGGAAGCTGACGG

XM_002272390.4 ATCCGGCTTCATCGCCGCA------AAG--CGTG-GCCGGAAACTCACCA

NM_001256464.1 CTCAGACTTCATTGGTGTG------AAG--CGTG-GCCGCAACCTCGCCG

XM_003546320.5 CTCAGACTTCATTGGTGTG------AAG--CGTG-GCCGCAACCTCGCCG

NM_001279196.2 CTCCGATTATGATCCCGCCGG----AAG--CTTCTACCGGAAACTTTCTG

AY035100.1 TTCCGATTATGCCCCTCTCGTCACCAAGGCCAAGGGCCGTAAACTCACGG

JF968116.1 CTCCGACCTCATATCCGCC------AAG--AGAT-GCCGCCAGGTCTCCA

JF968119.1 CTCCGACTTCATCGCCGCC------AAG--CGTA-GCCGTCAGCTCACAA

NM_130320.4 CGAAAACGCAAATACGTCTTGTTCTAGTTTGGAGTTGGAAGCGTAAAAAA

NM_001248198.2 ------------------------------------ACTCACCAAAAAGA

AY192368.1 TGGTGGTGCAATTCTTGCTGATATCATTCCTCCTC---GTGACCGCCGTT

NM_001247379.2 TGGTGGTGCAATTCTTGCTGATATCATTCCTCCTC---GTGACCGCCGTT

JX145122.1 TGGCGGTGCAATTATCTCCAACCTCCTCCCTCGCAACGGTAGCCGCAGGC

XM_002301454.4 TGGCGGTGCTATTTTAGCTGATATCATCCCTCGTAACCGCGGCCGCCGCG

XM_002320960.3 TGGCGGTGCTATTTTGGCTGGCCTCATCCCTC---ACCGTGGCCACCGCG

NM_001320016.1 CGGTGGTGCTATAATCGCTAATTTCATCCCTCGCGACCGTCGCCGTTGTG

NM_001328756.1 CGGTGGTGCTATAATCTCCGATTTCATTCCTCGCGACCGTCGCCGTGGTG

FQ392750.1 TGGAGGTGCTATCATCTCTGATTTCATACCTCGCAACCGGAACCGCCGCG

NM_001254494.3 TGGCGGTGCCATCATCGCTGACTTCATACCCCGCCGTGGAGGCCGCCGCC

NM_001254517.2 TGGCGGTGCCATCATAGCCGACTTCATTCCCCGCCGTGGAGGCCGCCGCC

AK316980.1 TC---GTTCGAATTTCTTCGATTTT------GACGCTG---AGTTCGAAG

NM_180251.3 GC---GATCCGATTTCTTCGATCTT------GACGATG---ATTTCGAAG

JF968115.1 GGCGAGATCCCATGTCTCCGATGTGTTCAATGATTACGTTAATTTCGAGG

JF968117.1 TGCGAGATCCCATATGTCCGACGTCTTCAACGACTATGTTAATTTCGAGG

JF968118.1 GGCGAGATCAGAAGTCATCGATGTTGTGAAGGATGAGTATGATTTCGAGG

NM_001247584.2 CTTGAGGTCT-AAGTTTATTGACCT-----TGAAGATG---AATTTGAAG

KJ401124.1 T---AAGCAT-TTGCGATCTGAAGT-----TGTTGACG---ACTTTGAAG

XM_004252354.4 TTATCACTCT-AAGCGCTTGAGATC-----TGAGAATGTAGACTTTGAAG

XM_002266972.5 GCTGTGGTCCGAAGCCGTCGAGGACG---ATGACGACGACGACTTCGAGG

XM_024596482.1 -----AGTT----GTTGCTGATCCC------GAAGATG---ATTTTGAGG

XM_006369031.3 -----AGTT----GTTATTAATCTT------GACGATG---ATTTCGAGG

XM_008341964.3 TCTGAGGTCCGAAATCGTTGACTTG------GACGACG---ACTTCGAGG

NM_001294046.1 TCTGAAGTCCGAAATCGTTGACTTG------GACGACG---ACTTCGAGG

NM_001251371.2 -------------GACTTCGAGGCTG---ATTTCAGGG---ATTTTAAAG

XM_006604868.4 -------------CTGAGAGAGATGT---GTGGAGGCG---CTATCATCT

NM_001354284.1 -------------GATTTCGAGGCAG---GGTTCAGAG---AATTCGAGG

NM_001349033.1 -------------GATTTCGAGGCTG---GGTTCAGAG---AATTCAAGG

NM_001155962.1 GCC---CGGCAAGAAGGGAGCGCCCCGGGA------GGAGGACTTCGAGG

NM_001157201.2 GCC---CGGCAGTAAGGGAGCTCCCCAGGACAAGGAGGAGGACTTCGAGG

AK111690.1 GATG--CTGCGGAAGGGGAAGGCGGCGGCG-GCGGAGGAGGACTTTGAGG

XM_008660266.4 GAG---GGGAGCCCCCGTGGAGCAGCATGAGCAGGAGGAGGATTTCGAGG

XM_015788266.2 GCG---AGGAGGCTGC-CACTGCGCCAAGAGGAGGAGGATGATTTCGAGG

NM_001156774.2 GAG---GCGCTC------GTTCGCGGCGGAGGACGATGAGGACTTCGAGG

NM_001155219.2 GAG---GCGCTC------GTTCGCGGCGGAAGACGATGAGGACTTCGAGG

EU837255.1 GCG---CCACTTCGGGGGGTTCGTGGAGGAGGACGACGAGGACTTCGAGG

XM_009403037.2 GGA---CGGCCGACGGCGGGCCGTGGAGGAGGCGGAGGATGACTTCGACG

XM_018823871.1 GGG---CAGCCGTCGCCGGCTAGCGCAGGAGACGGAGGATGACTTCGAGG

XM_009419716.2 GGG---CAGCCGTCGCTGCGCCGTGGAGGAGACGGAGGACGACTTCGAGG

XM_009419259.2 GAG---CGGCCACCGCCGGGCTGTGGAGGAGGTCGAGGATGACTTCGAGA

XM_009415720.2 GAG---CGGCAATCGCCGGGCTGTGGAGGTGACCGAGGATGATTTCGAGG

XM_009411978.2 GAG---GAG--GAGGAGCGGTCGTGGCCGTCGCCT----CCCGGCGGAGG

XM_009383333.2 GAA---GAG--ATGGACCATCCGTGGCCCTCGTCG----CGCTTTGGAGA

XM_009381868.2 AGG---GGTGGAGGTCCCGGACGATGACGACGACGACGACGACTTCGAAG

XM_009405824.2 TGA---CGACGACGACTTTGAGGCTGACTTCCGGGATTTCAACGACGAGG

XM_009389352.2 GGT---CGAAGAGGACTTCGAGGCTGACTTCCTGGAGTTCGATGATGAGT

XM_009391309.2 GGC---CGAGGACGACTTCGAGGCCGATTTCCAGGAGTTTGATGATGAGT

NM_001084343.2 TGAGAAATTTTACTTTTTAGCCCAAAACTTTTAAAACTTATACTTTGAAC

NM_001159194.1 CATCCCGCATCGCGGCGCCAAGCGGGGTCT-CTGCGCCGAG--GACATCT

XM_015789203.1 CATCCCGCAGCGGGAAGCCCA----------CCGCGC------GGCCACC

XM_015774497.2 AGATATGTGCGGCGGCGCCATCCCGCTGAT-CAGCAGCCGC--GGCC-CC

NM_001153709.2 AGGCGGCCGCCGCAGAGGCCCCTGCCGCC----GCGGCGT----------

NM_001254770.2 AAGCGGCGGCCGTAGAGGCTCCCGCGGCCC-CCGTGGCGTG--CGCCAGG

NM_001150750.2 AGGTCGTCGTCACGGTGGCGGGCGGGGA-------GGCGTG--CGGGACC

CM007650.1_142530856-142531634 GGGGCGAGGCGGCGGCG-CTTCCGCCGGGG-CCGCGGCAAG--------G

CM007648.1_220493755-220494609 CATGCCTCGCACTCCTC-TGACCCACAGAG-AGATAAGCCC--TGCTTCC

NM_001111800.2 AGTTACTCGAGGAATCCATGTGCGGCGGCG-CAATCATCTT--CGACTAC

XM_023301819.1 CG------GAGGAATCCATGTGCGGCGGTG-CAATCATCTT--CGACTAC

XM_015775299.2 TAGGACCGACCGATCCGATGTGCGGCGGTG-CAATCATCTA--CGACTAC

XM_009384249.2 GCCGA---ACAGCAAAGATGTGTGGCGGTG-CCATAATCTC--CGACTTC

XM_009385622.2 ----------AGCAAAGATGTGTGGCGGCG-CCATCATCTC--GGACTTC

XM_009388210.2 CGACA---GCAGCAAAGATGTGTGGCGGCG-CCATCATCTC--CGACTTC

XM_009389661.2 ----------GGGAAAGATGTGTGGCGGCG-CCATCATCTC--CGACTTC

XM_009393219.2 AAGAGG------CCGAGATGTGTGGCGGAG-CGATCATCTC--TGACTTC

XM_009415859.2 ---GGG------CGAAGATGTGTGGCGGAG-CGATCATCTC--CGACTTC

XM_008649541.3 GTTTCGTTGTTTCGACAATGTGCGGCGGAG-CCATCATCGC--CGACTTC

XM_015782909.2 TAGTAGC----AAGACAATGTGTGGGGGAG-CGATCATCGC--CGACTTC

NM_001349820.1 AG------GCACCAGC-ACGAATA-------CGCCGACG---ATGACTTC

EU837258.1 AGCAAGAGGCAGCAGA-GGAGCTT-------CGCCGACGTCGACGACTTC

EU847517.1 --------GCGCCGA---CGACTT-------CGAGGCCG-----CGTTCC

XM_015775100.2 --------GTGGAGG---CGGGTG-------TGATGACG------ACTTC

CT833260.1 GGAGTTCGACGCCGG---CGACTC-------CGACTCCG------ACTCC

AK067060.1 GGATTTCGATGTTGATTGCGATGA-------TGATGATGATGATGACTTT

XM_015795858.2 CGAGTTCCTCTCCCGCGACCACGA-------CGACGACG------ACGAC

EU847519.1 CGAGTTCCTCTCCCGCGACCACGA-------CGACGACG------ACGAC

FR720457.1 GGAGTTCATGGCTGCTGACGACGA-------CGACGACG------GCGGC

EU847520.1 GGAGTTCATCGCTGGCGACGACGA-------CGACGACG------ACGGC

NM_001143039.1 TGCAGCCGCCGCTACCGACGACGA-------CGACGACG---AAGAGTTC

AK073133.1 CGTCGACTTCGAGGCGGAGTTCGAGCTTTT-CGAGGACG---ACGACGAC

XM_006380178.2 TTGAGGATCTCTGGTCTGAACTTGACCCCTTCTCTGAATTTCTTAGATTT

XM_002315454.3 CTGAGGATCTCTGGTCTGAACTTGACTCCTTATCTGATTTTCTTGGACTT

XM_008392395.2 CGGAGGACCTCTGGTCAGATCTTGACACCATCTCTGACCTCCTTGGCATA

XM_029095961.1 AGAAGGACCTCTGGTCGGAGCTTGACACCATCTCTGACCTCCTCGGCATA

XM_002272390.4 AGGAGGACCTCTGGTCGGAGCTCGATACCATCTCGGACCTCCTCGGTTTG

NM_001256464.1 CGCAGGAACTATGGTCTGAGCTTGACCCTTTCTCTGACTTCCTTGGCTTC

XM_003546320.5 CGCAGGAACTGTGGTCCGAGCTTGACCCTTTCTCTGACCTCCTTGGCTTC

NM_001279196.2 CTCGTGACCTCTGGGCTGAGCTGGACCCTATCTCCGA-CTACTGGTCCTC

AY035100.1 CTGAGGAACTCTGGTCAGAGCTCGATGCTTCCGCCGCCGACGACTTCTGG

JF968116.1 CCCAAGACCTCTGGTCCGAACTCGACGCC---TCCGACTTCTTTTCCTTC

JF968119.1 CTCAAGATCTCTGGTCTGAACTCGACGCT---TCCGACTTCTTCGGCTTC

NM_130320.4 CAAAAAA--CAAAAATGTGTGGGGGAGCTATCATTTCTGATT------TC

NM_001248198.2 CGAAAGAAGCAAGCATGTGTGGCGGAGCAATCATCGCAGATT------TC

AY192368.1 TGTCATCCACCGACCTATGGCCGA-------CTGATTTCTGG------CC

NM_001247379.2 TGTCATCCACCGACCTATGGCCGA-------CTGATTTCTGG------CC

JX145122.1 TGTACGCCGCCGACCTCTGGCC---------CAACCCTCCGG------TC

XM_002301454.4 AAGCAGCTTCACAGTTCTGGCCAAACTCATGCTTCGATAAAC------TC

XM_002320960.3 TCGCGGCTTCTGAGTTCTGGCCCAACTCATCCTTCAATAAAC------CC

NM_001320016.1 TCACCGCCTCTGACATATGGCCGGACTCTCCCTTCGCCAAGT------TC

NM_001328756.1 TCACCGCTTCTGACATATGGCCGGACTCTCCCTTCGCTAAGT------CC

FQ392750.1 ACCCAGCCCCAGACCTCTGGCCGGACTCTTTCTTCGCTAAAC------CC

NM_001254494.3 TCACGGCCTCCGAGCTCTGGCCAAACTC---CTTCGCCAAAG------AC

NM_001254517.2 TCACGGCCTCCGAGCTCTGGCCTAACTC---CTTCGGCAAAG------AC

AK316980.1 CTGATTTCCAAGGTTTCAAAGATGATTCGTCTATCGATTGCG------AT

NM_180251.3 CTGATTTCCAAGGGTTTAAGGATGACTCGGCTTTTGACTGCG------AA

JF968115.1 CTGATTTTCTCGAGTTCAAGGATGAGTC------AGATGGTG------AA

JF968117.1 CTGACTTCCTCGAGTTTAAGGACGAGTC------CGATGGTG------AG

JF968118.1 CTGATTTCGTTGATTTCGTGGATGAGTG------TGATGATG------AG

NM_001247584.2 CTGACTTTCAGCACTTCAAGGATAATTC------TGATGATG------AT

KJ401124.1 CCGATTTTCAGGACTTCAAAGAGTTATC------CGATGATG------AG

XM_004252354.4 CTGATTTTCAGGATTTTAAGGATTTTTC------TGATGATG------AA

XM_002266972.5 CCGATTTTCTGGGTTTCAAGGATGACTC------CGACGACG------AG

XM_024596482.1 CTGATTTTCAAGAGTTTAAGGATGAGTC------TGATGTCG------AC

XM_006369031.3 CTGACTTTCAAGAGTTTAAGGATGAGTC------TGATGTCG------AT

XM_008341964.3 CTGATTTCCAGGAATTCAAGGACGAGTC------CGATGTGG------AC

NM_001294046.1 CTGATTTCCAGGAGTTCAAGGACGAGTC------CGATGTGG------GC

NM_001251371.2 ACGATTCTGA---------TATCGACGA------CGACGACG------AC

XM_006604868.4 CTGACTTCAT---------TCTCCTTTT------TGA-GACT------TC

NM_001354284.1 ATGACTCGGAAATCGAGGATGTCGATGA------TGAGGACG------AT

NM_001349033.1 ATGATTCGGAAATCGAGGATGTTGATGA------CGAGGACG------AT

NM_001155962.1 CCGACTTCCGCGAGTTCGAGCGCGGCCTCGGAGAGGATGACG------AC

NM_001157201.2 CCGACTTCCGCGAGTTCGAGCGCGGCCTCGGCGAGGATGACGTGGACAGC

AK111690.1 CCGACTTCCGCGAGTTCGAGCGTGGCATGAGCGACGACGAGGCGGAGGGG

XM_008660266.4 CCGACTTCGAGGAGTTCGAGGTGGAGTCCGGCGAGTCGGAGC------TC

XM_015788266.2 CCGACTTCGAGGAGTTCGAGGTGGATTCCGGCGAGTGGGAGG------TG

NM_001156774.2 CCGACTTTGAGGTCTTCGAGGCCGACTCCAGTGATTCAGATT------TG

NM_001155219.2 CCGACTTCGAGGACTTTGAAGCCGACTCCGGTGATTCGGATT------TG

EU837255.1 CCGACTTCGAGGAGTTCGAGGTGGACTCCGGGGACTCGGATT------TG

XM_009403037.2 CCTTGTTCCAGGAGTTCAACGAAGAGTACGGGAGGTCCAAGA------AC

XM_018823871.1 CTGACTTCCGGGAGTTCAACGACGAGTCAGGGGAGTCCGAGG------AA

XM_009419716.2 CCGACTTCCAGGAGTTCGACAACGAGTCAGGCGAGTCGGAGG------AA

XM_009419259.2 CCGACTTCTTGGAGTTCGAGTACGAGGCGCTGGAGTCGGAAG------TG

XM_009415720.2 CCGACTTCCTGGAATTCGACTACGAGTCCATGGATTCCGAAG------TG

XM_009411978.2 AGACGGACGATGACTTCGAGGCAGATTTCCAGGAGTTCGAGG------AG

XM_009383333.2 AGAAGGACGACGACTTCGAGGCGGATTTCATGCAGTTCGAGG------AG

XM_009381868.2 CTGACTTCCAGGAGTTCA---GCGATGAGACCCAGGTGGACG------AG

XM_009405824.2 CCGAGGAGGACGAGTTCGATGGCGGCGAGACGGAGGCGGGTG------AG

XM_009389352.2 CGGGGGAGGACGAGTTCG---AGGACGAGTTCGA---TGATG------AG

XM_009391309.2 CGTCGGAGGACGAGTTCG---ATGACGAGGAGGAGATCGATG------AG

NM_001084343.2 CCTTTAGTTCTGAGAAAATGTGCGGAGGAG-CTGTAATTTCCGATTACAT

NM_001159194.1 GGCCGCACG--CGG----CCGCCGAC----TTC-GACGACCTCCTCCACG

XM_015789203.1 GGCAGCAAG--CG------TGCCCTC----TGC-GCCTCCGACTTCTGGC

XM_015774497.2 GGCGGCAAG--AGG----AGCCTCTC----CGCCGCCGATGAGCTCTGGC

NM_001153709.2 --CAGCGTG--CGG----GTGCCC------CGGCGGCTGCGACCTCGC--

NM_001254770.2 TGCGGCGCGGACGG----GTGCGCGGCGGCCGGCTGCTGCGAGCTCGT--

NM_001150750.2 TGCGGCATCGACGG----GTGCCT------CGGCTGCGACTTCTTCGGGG

CM007650.1_142530856-142531634 TACGGCACG--TGG----TCGTCTCTC---TAGCT-CTGCACGCTAGCAT

CM007648.1_220493755-220494609 GTCTTGCT----------CTGCTCTCCCGGCGACAACA--CGCCATCCAG

NM_001111800.2 ATCCCGGC----------GCGG-CGCCGGGTGTCGACAGCCGACTTCTGG

XM_023301819.1 ATCCCGGC----------GCGG-CGCCGGGTGTCCGCCGCTGACTTCTGG

XM_015775299.2 ATCCCGGC----------GCGC-CGCCGGTTGTGCGCCTCCGACTTCTGG

XM_009384249.2 ATCCCTTC----------CCGCAACCACCAGCACCGCCGCCG-CCCTAAC

XM_009385622.2 ACCCCCCC----------GCGCAACCACATCCGCCGCCGCCG-CCCCGAC

XM_009388210.2 ATTCCTCA----------ACGCAACCAT---CACCGTCACTG-CCCTGAT

XM_009389661.2 ATCCCTCC----------CTGCAGCCAACACCGCCGCAGCCG-CCCCAAC

XM_009393219.2 GTGACCGCCG-TGG----AAGACCTTGATCTGACGCCTGGCGGCA--ATC

XM_009415859.2 GTG----------G----ATGGTCGTGATGTCACGCCGGACGACG--AAC

XM_008649541.3 GTCCCCGCC----G----GCGCCCGGCGCCCGGCAACAGACGACACCATG

XM_015782909.2 GTCCCGCCCG-CCG----GCGCCCGCCGCGCCGCTGCCTCCGACATCTCC

NM_001349820.1 GAGGCCGCCTTCG------AGGACTTCGA--CGACGACTTTGACGTGCAT

EU837258.1 GAGGCCGCCTTCG------AGCAGTTCGA--CGATGACTCCGACTTCGAC

EU847517.1 GCGACTTCGAC--------AACGACTCC----GATGACGAGGAGATGATG

XM_015775100.2 GAGGCGGCGTTTG------AGAGATTCG----GACG-TGAGGACTCTGAG

CT833260.1 GACTCCGAGTCCG------AGGAGGTAG-----ACGAGTACGACGTCGTC

AK067060.1 GAGGCTGATTTTG------AGGAGTTTGA--GGATGACTATGGCGATGAT

XM_015795858.2 GA--CCACGACGGT----CAGCATGTCGT--TGTTGCGCCGTTGATCCGT

EU847519.1 GA--CCACGACGGT----CAGCATGTCGT--TGTTGCGCCGTTGATCCGT

FR720457.1 GGACTCGTGTTAAG----TAGTAAATCTT--TGGTGTTGAGGTCACCA--

EU847520.1 GG---CGTTTCCAT------GTTCCCTTC--TGGTGCAGGGAC-------

NM_001143039.1 GAGGCCGAGTTCC------AGCTTTTCGA--CGACGATGACGAGTGCGAG

AK073133.1 GACGATGAGTTCG------AGCTTTCCGA--CGATGGCGACGAGAGTTTG

XM_006380178.2 GATTATCACTCTA----------ATGATAATAATGGTAGTAAAAAAGA--

XM_002315454.3 GATCATCGTTCTAT----GAACAATATTAATAATGGTAGCAAAAAAGAAA

XM_008392395.2 GACTA---CTCCA-------------------ACAGCATCAACAAA--CA

XM_029095961.1 GACCA---CTCCA-------------------ACAGCATCAACAAA--CA

XM_002272390.4 GATTC---TGCTA-------------------ACGGCGGTGATGATTTCA

NM_001256464.1 GATAC---CACCA----------------------ATTCCAAAAA-----

XM_003546320.5 GATAC---CACCA----------------------CCACCACCA------

NM_001279196.2 TTCTTCCTCATCCT----CA--------------ACCGTCGGAAA-----

AY035100.1 GGTTTCTATTCCA-------------------------------------

JF968116.1 TCCGG---AGTAG----------------------GCGATGAGAAG----

JF968119.1 TCTGG---AGTCG----------------------GCGATGAGAAC----

NM_130320.4 AT--CTGG-------------------------------------TCGAA

NM_001248198.2 ATACCTCG-------------------------------------GCAGC

AY192368.1 AATTTC--------------------------------------------

NM_001247379.2 AATTTC--------------------------------------------

JX145122.1 G-TCCC--------------------------------------------

XM_002301454.4 GGTCCC--------------------------------------TTTGAG

XM_002320960.3 AGTCCC--------------------------------------TTCGAC

NM_001320016.1 GATCCCGACACTT-----------------------------TCTTCGAC

NM_001328756.1 AATCCCGACACTT-----------------------------TCTTCGGC

FQ392750.1 GATGGC--------------------------------------TGTGAA

NM_001254494.3 GATGAC--------------------------------------TTTGAC

NM_001254517.2 GGTGAC--------------------------------------TTTGAC

AK316980.1 GATGATTTCGACG---TCGGTGATGTTTTCGCCGATGTGAAACCATTCGT

NM_180251.3 GACGAT------------GATGATGTCTTCGTCAATGTTAAGCCTTTCGT

JF968115.1 GAGATG------------GAA------------GATGTCAAGCCCTTTGC

JF968117.1 GAGTTA------------GAA------------GATGTCAAGCCATTTGC

JF968118.1 GTGTTT------------GGA------------GATGTAAAGCCTTTCAG

NM_001247584.2 GATG------------------------------ATGTGAAGGCATTTG-

KJ401124.1 GATGTT------------CAAG---T------CGATGTCAAGCCATTTGC

XM_004252354.4 GAAGCT------------TATAGTTT------GGATATCAAACCATTTGC

XM_002266972.5 GAGGAT---------------------------GACATCAAGCCTTCCGC

XM_024596482.1 GAGGAT------------TATGATGTCTTTGCTGATGCCAAGCCATTTGC

XM_006369031.3 GAGAAT------------TATGATGTCTTTGTTGATGCCAAGCCTTTTTC

XM_008341964.3 GAGGAC------------GATGAAAT---GGTTGATTTCAAGCCCTCTGC

NM_001294046.1 GAGGAC------------GATGAAAT---GGTTGATTCCAAGCCCTCTGC

NM_001251371.2 GATCAC------------CAAGTCAAGCC---------------CTTTGC

XM_006604868.4 TAATAT------------CCT-CAGGGTT---------------TTTTGG

NM_001354284.1 GAAGAG------------GAGGAGGAGTTGAAGAAGAAGAAGCCCTTTGG

NM_001349033.1 GAAGAC------------GAGGAGGAGTTGAAGAAGAAG---CCCTTTGG

NM_001155962.1 GTGGACGG------------GGCCGGCGAC---GAGGTCCAGGAGCTTCC

NM_001157201.2 GCCGGCGA------------AGGCGGCGACCCCGAGGTCCAGGAGCTTCC

AK111690.1 GGCGGCGGCGAGGAGGAGGAGGACGACGACGACGTGGTCGTGGTGGTCCC

XM_008660266.4 GAGTCC--------------------------------------------

XM_015788266.2 GAGTCC--------------------------------------------

NM_001156774.2 GAGCTCAG--------------------------------GGAGGGGACT

NM_001155219.2 GAGCTCGG--------------------------------GGAAGGGGCT

EU837255.1 GAGCTCGG--------------------------------GGAGGAGGAC

XM_009403037.2 GACGAAGA--------------------------------GG--------

XM_018823871.1 GACGACGT--------------------------------TG--------

XM_009419716.2 AATGACGA--------------------------------GG--------

XM_009419259.2 GAGGACGA--------------------------------GG--------

XM_009415720.2 GAGGACGA--------------------------------GG--------

XM_009411978.2 GACGGCGA--------------------------------GG--------

XM_009383333.2 GACGAGGA--------------------------------GG--------

XM_009381868.2 TTCGATG-------------------------------------------

XM_009405824.2 TTTGACG-------------------------------------------

XM_009389352.2 GTCGATG-------------------------------------------

XM_009391309.2 GTCGGTG-------------------------------------------

NM_001084343.2 AGCGCCGGAGAAGA----------TTGCGAGATCATCTGGAAAGTCTTCC

NM_001159194.1 CGCACGCGCACGCGCACGGCCAC-GACGAC---GACGACTTCGCCGCC--

XM_015789203.1 CGTCGGCGT-CGCAGGAAGCCGCCGACTTC---GACCACCTCACCGCC--

XM_015774497.2 CGCCGCCGC-CGCAGCACGCCAGCGACGACCCGGCCGAGCAAGCGGCG--

NM_001153709.2 CGCCGCGTCGAGCAG--CGACAGCGATGGCGCGGAGTGCTCCGCGA----

NM_001254770.2 CGCCGGGTCCAGCAG--CGACAGCGACGACGCAGGGTGCTCCGGGACCCG

NM_001150750.2 CGCCGGAGCTGGCGC--AGGAGGCAGTGTCCTGCGGCACAGGGCAGGTGG

CM007650.1_142530856-142531634 ATCTCTGTCCCGTAC--CGTACG---TTTCTGAGACCGCCGTGGGAC---

CM007648.1_220493755-220494609 GCAG--CAAGCTGCAAACGGTCAATGGTGTCAC-----------------

NM_001111800.2 CCTGGTTCTGAAGCAGACGCCGAGGACATTCAT-----------------

XM_023301819.1 CCTGACTCCGAAGCCGACGCCGAGGACTCCGACTCGCACGCCCCTGACCC

XM_015775299.2 CCCG------------ACGCCGACGACTCCGAC-----------------

XM_009384249.2 CCT------------GACAACCTCCTCTCCGCGTCTGACCTCT-------

XM_009385622.2 C------------------ACCTCCTCTCCGCCTCAGACCTCT-------

XM_009388210.2 CCC------------GATCACCTCCTCTCCGCCTCTGACCTCT-------

XM_009389661.2 CCCAGTTCCGACTCCGACAACCTCCTATCCGCTACAGACCTCT-------

XM_009393219.2 CCCC------------TCCCCGTCATGATGAGGAG---------------

XM_009415859.2 TCCA------------ACCCCGTCACGGGGAAGAG---------------

XM_008649541.3 TCCG------------CCTCCATCCTCTCCGGTAGC--------------

XM_015782909.2 GACA------------ACGCCGTCCTCTCCGCTGCC--------------

NM_001349820.1 GA------AGACGACGAGGACGGCCACTTCGTATTCTCGTCCAAAT----

EU837258.1 GACGCGGAGGAAGAAGACGAAGGACACTTCGTGTTCGCGTCCAAAT----

EU847517.1 GT---GGAGGAGGCGGAGGAGGAGGAGGCGACCTCCGAGCACAA------

XM_015775100.2 AT---GGAGGAGGAGGAGGTGGAGGAGGTGGTGGTTGGGAAGAA------

CT833260.1 GTCGACGACGACGACAGCGAGGACGGCGTGGTGGTTCTTCC---------

AK067060.1 GATGATGTGGGTTTCGGGGACGACGACCAAGAATCCGACATGAACG----

XM_015795858.2 GGTAGTGACAAGTGCGTCCACGGCCACGAGGTGGTGGCGTCGACGGTCGG

EU847519.1 GGTAGTGACAAGTGCGTCCACGGCCACGAGGTGGTGGCGTCGACGGTCGG

FR720457.1 GGTGAAAATGATGCAGGCCGGGGCGCCGCCGCCACCATGTCCATGC----

EU847520.1 GATGGAGACGACCACAGAGGTGGCGCCGGCG-----------GCGG----

NM_001143039.1 GCCGAGTTCCA---GCTTCTCGACGACCACCAACCATCTCCCGCAGCTTC

AK073133.1 GCCGTGTCATGTGTGTCGTCCCCCAAGTCGAAGGCAGTACCTTCGTTTTC

XM_006380178.2 -CCCATCAAATCTCTTGTTCCCTCAGAAGCCAAGCTACACCATCCAAGTC

XM_002315454.3 ACCTATCAAATCTCAAGTTCGCTCAGAAGCCACGCCAGCCCAACCAAGT-

XM_008392395.2 GCCGGAG-AATCACAAGGTGGTCCAAAAGCCGAAACCATCTATC--ACC-

XM_029095961.1 GCCTGAG-GATTACAAGGTGGTCCAGAAGCCCAAACCATCAGTC--ACC-

XM_002272390.4 ACCCGTCTGAAAATAGGGTTGCTCCAAAGCCAAAGCAA---GTG--AGC-

NM_001256464.1 --CCAACCACCCCTGCAGAAAATTCCAGACAAAAAAGTGGTGTC------

XM_003546320.5 --CCAACCAACCACCCC-----TTCCAGACAAAAAAGTGGTGTC------

NM_001279196.2 ----ACCTGATTCCGCTCTGTCGCCGGTGACTCACTCCGTCGATAAGCCT

AY035100.1 ---------------------CCTCCAAACTCCATCCCACCAAC------

JF968116.1 --------ACTGCCGGCACCGGCGATGAGAAGAAGAATAAGAAAC-----

JF968119.1 --------ATCACTGCCGGAGCTGAGGAGAAGAAGAATAAGAAGC-----

NM_130320.4 ATCTGAGTCAGAACCGAGTCAACTCGGCTCTGTTAGC-------------

NM_001248198.2 GTCCCCG-CACCCTCACAGCCTCCGAACTCTGGCCCA-------------

AY192368.1 -----------------CACCCAAAATGTTCCTCTCA-------------

NM_001247379.2 -----------------CACCCAAAATGTTCCTCTCA-------------

JX145122.1 -----------------CAAAGAGCATCAGGACATCA-------------

XM_002301454.4 TCCTGTCTGAGCCAACCTAGCAATCAGGAGTCATTTA-------------

XM_002320960.3 ACGTATCCGAGTCCTCTCCGCAATCAGGAGCCATTCA-------------

NM_001320016.1 TGCAATCCCACCCCGGTTAATCGCACCGACTCAACTC-------------

NM_001328756.1 TGCAATCCCACCCCAACTACTCACACCGACTCAATTC-------------

FQ392750.1 TACGATTTGGGCCGCTTTAGCCAGAAGGGGCTTCCTA-------------

NM_001254494.3 TTGGATTACTCCCACATCGCTACCCAACAACCCTCCA-------------

NM_001254517.2 TTCGATTACTCCCACATCGCCGATCAACAACGTTCCA-------------

AK316980.1 TTTCACTTCGACTCCAAAACCCG------CCGTCTCCGCCGCTGCGGAAG

NM_180251.3 CTTCACCGCAACTACTAAGCCCGTAGCTTCCGCTTTCGTCTCCACTGTAG

JF968115.1 TGCTAAAGG---GTTCAACCCTATCAGTTTCAATTCT-------------

JF968117.1 CGTCAAGGG---GTTGAACCCTTTCAATTTCAGCTCT-------------

JF968118.1 CTTCAGGCC---AGTCAATACTTTCAATTCCAGCTCT-------------

NM_001247584.2 --------------------------------------GC----------

KJ401124.1 CTTCTCTGC---TTCCAAACACTCTA---------CTGGT----------

XM_004252354.4 TTTCTCTGC---TTCTGAACTCTCTGGAACCTCTGCTGGA----------

XM_002266972.5 TTTCTCCGC---TGCGAAGCCACATG------------GC----------

XM_024596482.1 TTTCTCTGC---TAGTGTTTCTGAACCTGCTCAAAAACGTGGGCTCCCTC

XM_006369031.3 TTTCTCTGC---TACTGCTTCTGCCCCTGCTAAGAAACGT----------

XM_008341964.3 CTTCTCTGC---CGGAAAGCCCTCTTCTGCCCGTGGT-------------

NM_001294046.1 TTTCTCTGC---CGGAAACCCCTCTTTTGCTCGTGGT-------------

NM_001251371.2 TTTC---------------------GCCGCCTCTTCTCGTCTG-------

XM_006604868.4 GAGA---------------------ATTGGCTAAAAA-ATGGG-------

NM_001354284.1 GTTCTCTCG---CTCCAACAACAAGGCTGCTTCTAAGCCTCTC-------

NM_001349033.1 GTTCTCTCG---CTCCAGCAACAAGGCTGCTTCTAAGCCTCTC-------

NM_001155962.1 TCTGCCGGAGCCGGCGAGGCTCGCCTTCGCCGCCGCGGTTGGGGCGCCGC

NM_001157201.2 TCCGCCGGAGCCGACGAGGTTTGCCTTCGCCACCGCGGCCAAGGCG----

AK111690.1 CCCGCCGGCGGCGGCGAGGTTCGTCGTCCGTGCCGCGGCCAAGGCGGCGC

XM_008660266.4 ------GAGG---ACGAGCCCAAGC-CCTTCGCCGCCCCCAGGAGCGC--

XM_015788266.2 ------GACGCCGACGAGGCCAAGC-CGCTCGCCGCGCCCCGGAGCGG--

NM_001156774.2 GACGACGACGTCGTCGAGATCAAGC-CCTTCACTGC---CAAGAGGAC--

NM_001155219.2 GACGACGACGTCATCGAGATCAAGC-CCTTCGCCGC---CAAGAGTAC--

EU837255.1 GACGATGACGTCGTCGAGATCAAGC-CGGCCGCCTT---CAAGAGGGC--

XM_009403037.2 ----TGGAGCTCGTCGACCACAAGC-ACTGTGCTTT--------------

XM_018823871.1 ----TGGAGATCGTCGATGTCAAGC-TCCCTGCTTT--------------

XM_009419716.2 ----CCGAGCTCGTCGACGTTCAGT-TCTCTGCTTT--------------

XM_009419259.2 ----TGGAGATCGTGGAG---AAGC-CCTTCGCTTT--------------

XM_009415720.2 ----TGGAG------GAT---AAGC-CGCTCGCTTT--------------

XM_009411978.2 ----TTGATCTCTTCCATATCGAGT-CCTTCGCTTT--------------

XM_009383333.2 ----CTGACCTCTTCCACTTCAAGC-CCTTCGCTTT--------------

XM_009381868.2 ----CCGTGCACTTCGGCTTTGGAT-CGGAAGCCCC--------------

XM_009405824.2 ----CCGTGCACTTTGGTTTTGGAT-CCAAAGCCCC--------------

XM_009389352.2 ----CCAATTCCTCTGGTTTCGGAT-CGAATACCTC--------------

XM_009391309.2 ----TAAAATGCTTCGGTTTTGGGT-CGAAACCCTG--------------

NM_001084343.2 TGGAGAAGTAATGGCGTCTTTGACTGCTCAATCTACGATTTCGATGGAAA

NM_001159194.1 --------GCCGCCTCCTTCCATC----------CCGACCAAGAGCC---

XM_015789203.1 --------CCCTGCACCTTCACCC----------CCGACCAAGCGGC---

XM_015774497.2 --------GCGGATGAGGAGGAGC----------AGGAGCAGCAGCC---

NM_001153709.2 --------GCGGCGG-------------------------AGGCGCG---

NM_001254770.2 CGCCAACGGCGGCGGCCTAGGCCT----------AGGCCTAGGCGCG---

NM_001150750.2 CGACGACGGCGTCGGTCGGGGGCG----------GGGCAGCGGCGGC---

CM007650.1_142530856-142531634 --------GTGGCACCCC----------------------AGGAGGA---

CM007648.1_220493755-220494609 -------GCCGTGCTGCCGACTGT--TCC----CATGCAGCGCCGT----

NM_001111800.2 -------GCCTCGCACTCCACTGA--CCCA---CAGAGAGCGCCGCC---

XM_023301819.1 TGAGAGAGCTCCGCTGCCGACTGT--CCCGTCCCGTGCAGTGCCGCG---

XM_015775299.2 --------CCCCACACCC--CCGC--TCCC---GAGAAACCGCCGC----

XM_009384249.2 -------GGCCCAACTCCGACGCA---CCCCTCCATGTCCCTGCTCA---

XM_009385622.2 -------GGCCCGAGTCC-------------------TTTCAGGATA---

XM_009388210.2 -------GGCCCGATTCCTTCCAC---CCACACAGTGTCTCCACCCA---

XM_009389661.2 -------GGCCGGGCTCCTTCGACGACCCCCAACATGTCCCCACCCA---

XM_009393219.2 -------GGCGAGGGCACGAAG----------------ACCGGGGCC---

XM_009415859.2 -------GGCGAGGATACGAAG----------------ACTAGGGCT---

XM_008649541.3 -------GAGGACCATCCGGAGCT--------------GCCGCTGCC---

XM_015782909.2 -------GGTGCCGGTGACGAGTC--------------GTTCGCGGC---

NM_001349820.1 CCGCCTTGTCCCCAGCCCTGCACGACGG-GCGCGCGGCGA----------

EU837258.1 CT--CGTGTCGTC-GCCGGGCACGACGG-GCGCGCGGCGGCGAGGGC---

EU847517.1 -------GCCGTTCGTCTTCCGCGCCAA-GAAGGCGGCGGCGGCGGC---

XM_015775100.2 -------GGCGGCGGTGA--GGCGGCG--GAGGGCGACGCC--CGCC---

CT833260.1 -------GCCGCC-GCCGCCGCCGCCG------CCGGTGAT---------

AK067060.1 GTCTCAAGCTCGCCGGATTCAGCACCA-------CGAAGCTCGGCCT---

XM_015795858.2 CGGTGGCGCAAGCGGCGGACGACGACGA-GCCGACGACGACGACGGCGAG

EU847519.1 CGGTGGCGCAAGCGGCGGACGACGACGA-GCCGACGACGACGACGGCGAG

FR720457.1 CGCTGGACCCCGTGACCGAGGAGGCCGA-GCCGGCGGTGGCTGAGA----

EU847520.1 CGGTGG----------TGGAGAGGCCG----CGGCGGCGGCGAAGG----

NM_001143039.1 GCCTGAGGCCAGCGGCTGC--AAGCGGAAGCCTGCCCTTGCTCCTCCTGC

AK073133.1 TTTTTCGTCGGATGTCTCCTCGAGCTCCAGGCCGCGGCGGCGCGTGGCGG

XM_006380178.2 CAGCAAGTGATAACAGAGAAAGTTGAGAAGCCAAGCCATGCCACAGAGAA

XM_002315454.3 --------GATAACAGAGAGAGTTGAGAAGCCAAGCCAAGCAACAGAGCA

XM_008392395.2 ---------AAAGTGACAAGTGATGAGAAGCCCAAGCAGGCGAGCGGGTC

XM_029095961.1 ---------AAAGCGACAAGTGATGCGAAGCCCAAGAAGGCCACCGGGGC

XM_002272390.4 ---------AAAGGGACGA---ACGAGAATACCCAGAAAGCGATTCGGGT

NM_001256464.1 -----------------ATCATGTGAGAAGAAGAAGAAAAGCGTGGTGGG

XM_003546320.5 -----------------ATCATGTGAGAAGAAGAAGAAGAAAAGTGTGAG

NM_001279196.2 ---------AATAAATCAGATTCCGGCAAAAAAGGTAATAAGACTGTGAA

AY035100.1 ------------------CAAGTTAACGTGAAAGAGGAGGCAGTGAAGAA

JF968116.1 ------------------AGCGCAGCGGTGGAGGAGACGGAGA---CGGA

JF968119.1 ------------------AGCGGGGCGGCGGCGGAGATGGCGGATCCGGG

NM_130320.4 ----AGCAGGAAGAAGCGTAAACCC-----------GTCTCAGTGAGTGA

NM_001248198.2 ----AGCGCAGCGAACCTCAACCTC-----------AACCTCCTCCTCCT

AY192368.1 ----ACCCCAAACGAGCTCGACCCT------------CTACAGG---TGG

NM_001247379.2 ----ACCCCAAACGAGCTCGACCCT------------CTACAGG---TGG

JX145122.1 ----CACACAAGAGGCCTCAATCCC------------CTTCAGG---TGG

XM_002301454.4 ----CTCTCAAACGACCCCAACCCCAACCCCAACCCGCTTCAGGGGATCA

XM_002320960.3 ----CTCTCAAACGACCCCAGCCC------------ACTTCAGGGGATCA

NM_001320016.1 ----CGCGTAAATTATCCCAACCC------------ATTTCAGG---TGA

NM_001328756.1 ----CGCGTAAAAAATCCCAACCC------------ATTTCAGG---TAA

FQ392750.1 ----ATCTGAAAAGGTCTCAACCC------------ATTTTGGA---TGA

NM_001254494.3 ----CTCTCAAAAGGTCCCAACCT-------------CCCAAAGT--TAG

NM_001254517.2 ----CTCTCAAAATGTCCCCACCT-------------CCCAAAGC--TAG

AK316980.1 GTTCAGTTTTTGGTA---AGAAAGTTA-------------CTGGCTTGGA

NM_180251.3 GTTCAGCATATGCCA---AGAAAACTG-------------TAGAGTCCGC

JF968115.1 -----TCTCAAGGAT---CAAAGGCTA-------------CTGAGTTCAA

JF968117.1 -----TCTCAAGGGC---TGAAATCTG-------------CTGAATTCAC

JF968118.1 -----ACTCAAGGAC---CAAAGCCTG-------------TTGAGTTCAC

NM_001247584.2 -----CCCAAATCCG---TGAGATCTG-------------GTGATTCAAA

KJ401124.1 -----TCCAAATCTT---TGAAAACTG-------------TTGATTCAGA

XM_004252354.4 -----TCCGAATCAC---TGATATCTG-------------TTGATGCAAA

XM_002266972.5 -----TATACAGGTA---TAAAATCTG-------------TGGAATTTAA

XM_024596482.1 GTGGTTCTCCTGCTG---TTAAATCTG-------------CTGGATTCAG

XM_006369031.3 -----TCTGCGCCTG---TTAAATCTG-------------CTGAATTCAG

XM_008341964.3 -----TCTACTGCTG---TGAAATCTG-------------TGGAGTTCAA

NM_001294046.1 -----TCTACCGCTG---TGAAATCTG-------------TGGAGTTTGA

NM_001251371.2 -----TCTACGGCAG---CGAAATCTG-------------TGGCATTCCA

XM_006604868.4 -----TCTACCACAG---CGAAATCTG-------------TGGCATTCCA

NM_001354284.1 -----TCTCGTGGAG---CAACAACTGT----GAAATCTGTGGAATCAAA

NM_001349033.1 -----TCTCGTGGAT---CAGCAACTG-------------TGGAATCAAA

NM_001155962.1 GCACTGCAGTTGACGGCGTGATGACTCC----AAAGGATGGTGAAGGAGA

NM_001157201.2 -----GCAGTTGATGGCGTGATGACTCC----------TCCTCCAAAGGA

AK111690.1 CCCCAACTGCAGATGGGATGTTGACTAC----AAAGCTTGTCCAACATGA

XM_008660266.4 -GCTCGCCAGAGGTGGACT---AAACAC----TGGTGCAGCTGGTGTCGA

XM_015788266.2 -CTTCGCTAAAGGTGGATTGAAAAACAC----TACTGTTGCTGGTGCTGA

NM_001156774.2 -TTTCTCCAGCGATGGCTTAAGCACCAT----GACTAGTGCTG-------

NM_001155219.2 -TTTCTCCAGAGATGGCTTAAGCACCAT----GACTACTGCTGGTTATGA

EU837255.1 -CCTCTCCAGAGATAACTTGAGCACCAT----TACCACTGCCGGATTTGA

XM_009403037.2 -CCCTCCCAAAGGTGGGCCAATTAGCTC----AAGACCTATTGAATCTGA

XM_018823871.1 -CGCTCCCAAAGATGGGCAAATTGCTTT----AGGACCTGTAGAATTTGA

XM_009419716.2 -CCCTCCCAAAGATGGAGAAATGACTTT----CAAACCTGTAGGATTTGA

XM_009419259.2 -CGCCTGCAAAGATGGACCAGTTGCTCT----GAAACCTGTGAAATTCAA

XM_009415720.2 -CGCCTCCAAAG---------TCACTCT----GAAATCTGTAGAATTCAA

XM_009411978.2 -AACTTCGGAAGATAAACCAGTCCCCAC----GAGACCTTCAGAGGTTGA

XM_009383333.2 -CGATTCGAGAGATAAACCAGTCCCTCT----GAGGCCTTCAGTGATTGA

XM_009381868.2 -GTTCCCGCGAGATGGCTCACC----------AAAGTCTGTATATCTTGA

XM_009405824.2 -CTTCACCCGAGAGGGCTCAGC----------TAAGTCTGTAAGGTTTGA

XM_009389352.2 -CTTTTCTCGAGAGATCTCAGTCACTCT----AAGATCAAGAGGTTTTGG

XM_009391309.2 -CTTCTCTCGAGAGCGCTCAACCACTCT----AAAATCAGGAGATTTTAG

NM_001084343.2 TTTCGATGAATTAGAGTCCGATGAGCCA--TTTGTCTTCTCCTCTACTCA

NM_001159194.1 -------GCCGG---------CCCGCAAGCG--------GGAGCGCAAGA

XM_015789203.1 -AGAGGAGCCGA---------CCAAGAAGCG--------GGAGCGGAAGA

XM_015774497.2 -GGCGGCGAGGA---------GGCAGCGGCGA-----GGGGAGCGGAGGA

NM_001153709.2 -GGCAA-GCGGA---------GGCGGAGGCG------GAGCAGGGCGAGC

NM_001254770.2 -AGCAA-GCGGA---------GAGGGAGGGG------GAGGAAGGTGATC

NM_001150750.2 -GGCGCTGAGGC---------CCCGGCGGCG------TAGGAACAGGAAC

CM007650.1_142530856-142531634 -GGGACGACGGA---------GCTGCAGGCG------GCGGCGCGGGCAC

CM007648.1_220493755-220494609 ------GCGCGA---------AGGTGAAGCCT-----GGGCGCTGCAAGA

NM_001111800.2 -GCCGCGCGCGA---------AGGCGAAGCCT-----GGGCGCCGCAAGA

XM_023301819.1 -CCCGCGCGGGA---------AGGCGAAGCGT-----GGGCGC---AAGA

XM_015775299.2 ------GCGCGA---------AGAGGGAGC--------GG------AAGA

XM_009384249.2 -GGACAAAGTGC---------CTCGGAAGCGG-----GAGAGG---AAGA

XM_009385622.2 -AAGC--AGCGC---------CTCGGAAGCGG-----GAGCGG---AAGA

XM_009388210.2 -GGACAAAGTGC---------CTCGGAAGCGG-----GGGCGG---AAGA

XM_009389661.2 -GGATAAAGCGC---------CTCGGAAGCGG-----GAGCGG---AAGA

XM_009393219.2 -GCAG--GGGGA---------GGGCAGAGGCG-----GCGG-----AAGA

XM_009415859.2 -GCGG--GGCAA---------GGGGAGAGGCG-----GCGG-----AAGA

XM_008649541.3 -GCTGCCGGCGC---------CGGCGCCGG-G-----GCGC-----AAGA

XM_015782909.2 -GGCCAAGGCGC---------CGGCGCCGG-G-----GAGG-----AAGA

NM_001349820.1 ---------------------GCCAG-AAG-------AAGCAGCGCGGGC

EU837258.1 -----------G---------GCGAGCAAG-------AAGAAGCGGGGGC

EU847517.1 -----------G---------TCGAGCAGG-------CGCAGGAAGCCGG

XM_015775100.2 -----------G---------CCGGGC-GC-------CGCGCGAGGCCGA

CT833260.1 -----------T---------CCACATGAG-------CGCCATGGCGCGA

AK067060.1 -----------C---------GGCGGCAGC-------AGGAAGAGGAAGA

XM_015795858.2 CGGCGGCGGCGG---------CGGCGGAGG-------GAGAAGCGGAGCT

EU847519.1 CGGCGGCGGCGG---------CGGCGGAGG-------GAGAAGCGGAGCT

FR720457.1 ----AGCCTCGC---------CGGCGCCGG-------CCGAGGCGGAGCT

EU847520.1 ----G---------------------------------TGAGGCGGAGCT

NM_001143039.1 TGGTGCCCCCGC---------TTCCACGGATCCGGCGACCCCGTGCTCCA

AK073133.1 CGGCGGCGGCCG---------GTCGTCGGA--AGGCGAGCAAGAAG---A

XM_006380178.2 AGA---AAATGG---------TAACAAGAAGGCTCAAAGAACCAGAAAGA

XM_002315454.3 AGA---AGCCGG---------TAAAAAGAAGGTTCAGAGAACCAGAAAGA

XM_008392395.2 ------TGCTGC---------TGCCGCAAAAGGCAAGAGAGTGAGGAAAA

XM_029095961.1 AGCTGCCGCTGC---------TGCTGCAGAAGGCAAGAGAGTGAGGAAAA

XM_002272390.4 GGAGGAAAAGAA---------GAGTACCACCACTCCGAGGATTCGGAAGA

NM_001256464.1 TGCAGAAAAGAA---------GAAGAGTGATAGTGGGCGAGCTCGTAAAA

XM_003546320.5 TGCAGAAAAGAA---------GAG------TGGTGGGCGAGCTCGGAAGA

NM_001279196.2 GGTTGAGAAGGA---------GAAGAGTAGTGGACCAAGGCCAAGGAAGA

AY035100.1 GGAGCAGGCAAC---------AGAGCCGGGGAAACGGAGGAAGAGGAAGA

JF968116.1 AAA-CAGACGGC---------GCCGGAACCTGCTAAGCCGGCAAGGAAGA

JF968119.1 AAA-CAGATGGC---------GTCGGATCCAGCTAAGCCGCAGAGGAAGA

NM_130320.4 AGAAAGAGATGG---------------------GAAACGAGAGAGGAAGA

NM_001248198.2 CCA----GTTAA---------------------GAGGC---AGAGGAAAA

AY192368.1 TGAGCAGATGAA---------------------GAAGAGGCAAAGGAAGA

NM_001247379.2 TGAGCAGATGAA---------------------GAAGAGGCAAAGGAAGA

JX145122.1 TGAGCGGGTCGA---------AAAGGCT---GTGAAGAGGCAGCGCAAGA

XM_002301454.4 TGAGCAAATGGA---------GAAGCCCAATGCCAAGAGGCAGAGGAAGA

XM_002320960.3 TGAACAAGTGGA---------GAAGCCGAATGCCAAGAGGCAGAGGAAGA

NM_001320016.1 TGTACAAGAAGA---------GAAGCCG---GCTAAGAGGCAGAGGAAGA

NM_001328756.1 TGTGCAAGAAGA---------GAAGCCG---GCTAAGAGGCAGAGGAAGA

FQ392750.1 TGAGCCAGAAGT---------GAAGCCG---GCTAAAAGGGTGAGGAAGA

NM_001254494.3 CGAGCAGGTTGA---------GAATAAGCCGGTGAAGAGGCAGAGGAAGA

NM_001254517.2 CGAGCAAGTGGA---------GAATAAGCCGGTGAAGAGGCAGAGGAAGA

AK316980.1 TGGGGACGCTG---------AGAAATCTGCA---AATAGGAAGAGGAAGA

NM_180251.3 TGAGCAAGCTG---------AGAAATCTTCT---AAGAGGAAGAGGAAGA

JF968115.1 CAGCCAGGCTG---------AGAAGTCTGCC---AAGAGAAAGAGGAAGA

JF968117.1 CAGCCAGGCTG---------AGAAATCTGCC---AAGAGAAAGAGGAAGA

JF968118.1 TGGCCAGACTG---------AGAAATCTGCC---AAGAGAAAGAGGAAAA

NM_001247584.2 CTGCGAAGCTG---------ACAGATCCTCC---AAGAGAAAGAGGAAGA

KJ401124.1 CAAGGATGCTGCTGC---TGATAAATCCTCT---AAGAGAAAGAGGAAGA

XM_004252354.4 CAAGGAAGTTG---------AGAAATCTGCC---AAGAGACAGAGGAAGA

XM_002266972.5 TGGGCAAGCTG---------AAAAATCTGCA---AAGAGAAAGAGGAAGA

XM_024596482.1 TGGACCTGCTG---------AAAAATCAGCC---AAGAGGAAGAGAAAGA

XM_006369031.3 TGGGCAAGCTG---------AAAAATCAGCA---AAGAGGAAGAGAAAGA

XM_008341964.3 TGGGCAAGCTG---------AGAAATCTGCA---AAGAGAAAAAGGAAGA

NM_001294046.1 TGGGCAAGCTG---------AGAAATCTGCA---AAGAGAAAAAGGAAGA

NM_001251371.2 AGGTCGGGCTG---------AGATATCTGCA---AATAGAAAGAGGAAGA

XM_006604868.4 AGTTCGAGCTG---------AGAAATTTGCA---AATAGGAAGAGGAAAA

NM_001354284.1 GGGGCAAGCTG---------AGAAGTGTGCC---AAGAGAAAGAGGAAGA

NM_001349033.1 GGGGCAAGCTG---------AGAAGTGTGCC---AAGAGAAAGAGGAAGA

NM_001155962.1 CATGCCTACTACCGCTACCAACTCAGCAGCAAATAAGCGCAGGCGGAAGA

NM_001157201.2 TGT-CCAAGGA--------GATAGAGCAGTAAAAAAGCGCGGCCGGAAGA

AK111690.1 TGGACCTACTGC---------TAGATCAGCA---AAGCACAAGAGGAAGA

XM_008660266.4 TGGCCCTGCTGC---------AAATTCAGTT---AAAAGGAAGAGGAAGA

XM_015788266.2 TGGGCCTGCAGC---------AAGGTCTGCT---AAAAGGAAGAGAAAGA

NM_001156774.2 --------TAGC---------AAGGTCAGCC---AAGAGGAAGAGAAAGA

NM_001155219.2 TGCCCCTGCAGC---------AAGGTTGGCC---AAAAGGAAGAGGAAGA

EU837255.1 TGGTCCTGCTGC---------AAAGTCTGCC---AAAAGAAAGAGAAAGA

XM_009403037.2 TGGACCTGCAGC---------TAGGTCTCCT---AAAAGAAAGAGAAAGA

XM_018823871.1 TGGACCTGCTGC---------CAGGTCTGCT---AAAAGGAAGAGAAAGA

XM_009419716.2 TGGACCTGCAAT---------GAGGTGTGCT---AAGAGGAAGAGAAAGA

XM_009419259.2 TGGATCTGCAGC---------TAGGTCTGCT---AAAAAAGAGAGAAAAA

XM_009415720.2 TGGACCTGCAGC---------TAGGTCTACT---AAAAGGAAGAGAAAGA

XM_009411978.2 TGTACCTGCATC---------CAGGTCTGCT---AAAAGGTACAGAAAGA

XM_009383333.2 TAAACCTTCATC---------TAAGTCTGCT---ACAAAGAACAGAAAGA

XM_009381868.2 TGGACCTGCCAT---------AAAATCAAGC---AAAAGAAAAAGAAAGA

XM_009405824.2 TGGATCTGCTGC---------TAAATCAGCC---AAAAGAAAGAGAAAGA

XM_009389352.2 TGGACCTGCATG---------CAAGTCAGGC---AAAAGGAAGAGAAAGA

XM_009391309.2 TGGATCTGAAGT---------TCAGTCAGCC---AAAAGGAAGAGAAAGA

NM_001084343.2 CAAACATCATGCTTCAGGCTCAGCATCAGATGGGAAGAAGAAACAGAGCA

NM_001159194.1 CCATGTACCGCGGCATCCGGCGCCGCCCCTGGGGCAAGTGGGCGGCGGAG

XM_015789203.1 CGCTGTACCGTGGCATCAGGCGGCGGCCGTGGGGGAAGTGGGCGGCGGAG

XM_015774497.2 CGCTGTACCGGGGCATCCGGCGCAGGCCGTGGGGGAAATGGGCGGCGGAG

NM_001153709.2 A--GTTACATGGGCGTGCGGCGGCGGCCGTGGGGCAAGTGGGCGGCGGAG

NM_001254770.2 C--AGTACAGGGGCGTGCGGCGGCGGCCGTGGGGCAAGTGGGCGGCGGAG

NM_001150750.2 A--TGTACCGCGGCGTGCGGCAGCGGCCGTGGGGCAAGTGGGCGGCGGAG

CM007650.1_142530856-142531634 ATCAGTACAGGGGCGTGAGGCGGCGGCCGTGGGGCAGGTGGGCGGCGGAG

CM007648.1_220493755-220494609 TCCAGTACCGCGGCATCCGGCAGCGGCCGTGGGGCAAGTGGGCGGCGAAG

NM_001111800.2 ACCAGTACCGCGGGATCCGGCAGCGGCCGTGGGGCAAGTGGGCGGCGGAG

XM_023301819.1 ACCAGTACCGCGGCATCCGGCAGCGGCCGTGGGGCAAGTGGGCGGCAGAG

XM_015775299.2 ACCAGTACCGCGGGATCAGGCAGCGGCCGTGGGGGAAGTGGGCGGCGGAG

XM_009384249.2 ACCTGTTCAGGGGGATACGGCAGCGACCCTGGGGGAAGTGGGCAGCGGAG

XM_009385622.2 ACCTGTACAGGGGGATACGGCGGCGGCCGTGGGGGAAGTGGGCGGCGGAG

XM_009388210.2 ATTTGTACAGGGGGATACGACAGCGGCCGTGGGGGAAGTGGGCAGCGGAA

XM_009389661.2 ACCTGTACAGGGGGATACGGCAGCGGCCGTGGGGGAAGTGGGCGGCTGAG

XM_009393219.2 CGTTGTACAGGGGGATACGGCGGCGGCCCTGGGGGAAGTGGGCCGCGGAG

XM_009415859.2 CGTTGTACAGGGGGATACGCAGGCGGCCGTGGGGGAAGTGGGCGGCGGAG

XM_008649541.3 CGGCGTACCGCGGGATCCGGCGCCGCCCGTGGGGCCGGTGGGCGGCGGAG

XM_015782909.2 CGGCGTACCGCGGCATCCGGCGCCGGCCGTGGGGACGCTGGGCGGCGGAG

NM_001349820.1 GCCAGTTCCGCGGCATCCGGCAGCGGCCCTGGGGCAAGTGGGCGGCGGAG

EU837258.1 GGCACTTCCGAGGCATCCGGCAGCGGCCATGGGGGAAGTGGGCGGCGGAG

EU847517.1 CGCAGTACAGGGGCGTGCGGCGCCGGCCGTGGGGGAAGTGGGCGGCGGAG

XM_015775100.2 GCAAGTACTGGGGCGTGCGGCGCCGGCCGTGGGGGAAGTGGGCGGCGGAG

CT833260.1 GGCGGTTCCGCGGCGTGAGGAAGCGGCCGTGGGGGAAGTGGGCGGCGGAG

AK067060.1 CGCGATACCGAGGGATCCGGCAGCGGCCATGGGGGAAATGGGCGGCGGAG

XM_015795858.2 ACCCGTACCGCGGCATCCGGCAGCGGCCGTGGGGGAGGTGGGCGTCGGAG

EU847519.1 ACCCGTACCGCGGCATCCGGCAGCGGCCGTGGGGGAGGTGGGCGTCGGAG

FR720457.1 ACGAGTACCACGGCATCCGGCAGCGGCCGTGGGGGCGGTGGTCGTCGGAG

EU847520.1 ACCCGTACCGCGGCGTCCGGCAGCGGCCGTGGGGGCGGTGGGCGTCGGAG

NM_001143039.1 GGAAGTACAGGGGCGTCCGGTACCGCCGGTCAGGCAGGTGGGCCGCGGAG

AK073133.1 GCAAGTACAGGGGCGTCCGGCGCCGGCCGTCGGGGAGGTTCGCGGCGGAG

XM_006380178.2 GTGTGTATAGAGGAATAAGGCAAAGGACATGGGGCAAATGGGCAGCTGAA

XM_002315454.3 ATGTGTATAGAGGAATAAGGCAAAGGCCATGGGGTAAATGGGCAGCTGAA

XM_008392395.2 ACGTGTACAGAGGAATAAGGCAGAGGCCGTGGGGCAAATGGGCGGCTGAG

XM_029095961.1 ATGTGTACAGAGGAATAAGGCAGAGGCCGTGGGGCAAATGGGCGGCTGAG

XM_002272390.4 ACGTGTACAGAGGAATCCGGCAGCGGCCTTGGGGAAAATGGGCGGCGGAG

NM_001256464.1 ACGTGTACAGAGGAATCAGGCAAAGGCCATGGGGCAAGTGGGCCGCGGAG

XM_003546320.5 ACGTGTACAGAGGAATCAGGCAAAGGCCGTGGGGCAAGTGGGCCGCGGAA

NM_001279196.2 ACAAGTACAGAGGAATAAGACAGAGGCCATGGGGAAAATGGGCTGCTGAG

AY035100.1 ATGTTTATAGAGGGATACGTAAGCGTCCATGGGGAAAATGGGCGGCTGAG

JF968116.1 ACGTTTACAGAGGAATAAGGCGTCGGCCATGGGGGAAATGGGCGGCGGAG

JF968119.1 ATGTATACAGAGGGATAAGGAAGCGGCCATGGGGGAAATGGGCCGCAGAG

NM_130320.4 ATCTGTACAGAGGGATAAGGCAGAGGCCATGGGGCAAATGGGCAGCGGAG

NM_001248198.2 ACCTCTACAGAGGGATCAGGCAGCGGCCCTGGGGCAAATGGGCCGCGGAG

AY192368.1 ATCTTTACAGAGGGATAAGACAACGTCCATGGGGTAAATGGGCTGCTGAA

NM_001247379.2 ATCTTTACAGAGGGATAAGACAACGTCCATGGGGTAAATGGGCTGCTGAA

JX145122.1 ACCTGTACAGGGGAATCAGGCAGCGTCCGTGGGGCAAATGGGCGGCGGAG

XM_002301454.4 ATCTATACAGAGGTATAAGGCAACGACCTTGGGGTAAATGGGCAGCTGAG

XM_002320960.3 ATCTATACAGAGGTATAAGGCAACGACCTTGGGGTAAATGGGCAGCTGAG

NM_001320016.1 ACCTCTACCGAGGCATCAGGCAGCGTCCGTGGGGCAAATGGGCCGCCGAG

NM_001328756.1 ACGTGTACCGAGGCACCAGGCAGCGTCCGTGGGGCAAATGGGCCGCCGAG

FQ392750.1 ATCTGTACAGGGGGATCCGGCAGCGTCCTTGGGGAAAATGGGCGGCTGAG

NM_001254494.3 ATCTCTACAGAGGGATTCGGCAGCGTCCGTGGGGCAAATGGGCCGCGGAG

NM_001254517.2 ATCTCTACAGAGGGATTCGGCAACGTCCGTGGGGAAAATGGGCCGCGGAG

AK316980.1 ATCAGTACCGAGGGATTAGGCAACGTCCTTGGGGAAAATGGGCTGCTGAG

NM_180251.3 ATCAATACCGAGGGATTAGGCAGCGTCCTTGGGGAAAATGGGCTGCGGAG

JF968115.1 ATCAGTACCGTGGTATCCGTCAACGCCCATGGGGCAAGTGGGCTGCTGAG

JF968117.1 ATCAGTACCGTGGTATTCGCCAACGCCCATGGGGCAAGTGGGCCGCTGAG

JF968118.1 ATCAGTACCGAGGGATCCGCCAGCGTCCATGGGGTAAATGGGCCGCCGAG

NM_001247584.2 ATCAGTACCGGGGGATCAGACAGCGTCCTTGGGGTAAGTGGGCAGCTGAA

KJ401124.1 ATCAATATAGAGGGATCAGACAGAGACCTTGGGGTAAGTGGGCAGCTGAA

XM_004252354.4 ATCAGTATAGGGGGATCAGAAAGCGTCCTTGGGGTAAGTGGGCAGCTGAA

XM_002266972.5 ATCAGTATAGAGGAATCCGACAACGCCCTTGGGGAAAATGGGCTGCAGAG

XM_024596482.1 ACCAGTTTAGAGGAATTAGGCAGCGTCCATGGGGAAAATGGGCTGCTGAG

XM_006369031.3 ACCAGTATAGAGGAATCCGGCAGCGCCCATGGGGAAAATGGGCTGCCGAG

XM_008341964.3 ACCAGTACAGGGGAATTCGCCAGCGCCCATGGGGAAAGTGGGCTGCAGAG

NM_001294046.1 ACCAGTACAGGGGAATTCGCCAGCGCCCATGGGGTAAGTGGGCTGCAGAG

NM_001251371.2 ATCAGTATAGGGGAATCCGTCAACGCCCTTGGGGAAAATGGGCAGCTGAG

XM_006604868.4 ATCAGTATAGGGGAATCCGTCAACGCCCTTGGGGAAAATGGGCAGCTGAG

NM_001354284.1 ACCAGTATCGCGGAATCCGCCAGCGTCCATGGGGAAAGTGGGCTGCTGAG

NM_001349033.1 ACCAGTATCGTGGAATCCGTCAGCGTCCATGGGGAAAGTGGGCTGCTGAG

NM_001155962.1 ACCAGTACAGGGGAATCCGGCGGCGGCCCTGGGGCAAGTGGGCGGCCGAG

NM_001157201.2 ACCAGTACAGGGGAATCAGGCAGCGGCCTTGGGGCAAATGGGCAGCTGAG

AK111690.1 ATCAGTACAGGGGGATCCGCCAGCGTCCCTGGGGCAAATGGGCAGCTGAA

XM_008660266.4 ACCAGTTCAGGGGTATCCGCCGGCGCCCGTGGGGCAAATGGGCTGCTGAG

XM_015788266.2 ACCAATTCAGGGGTATCCGCCAGCGGCCATGGGGCAAATGGGCTGCGGAA

NM_001156774.2 ATCTATACAGGGGTATCCGCCAGCGGCCTTGGGGCAAGTGGGCTGCTGAG

NM_001155219.2 ATCAATACAGGGGTATCCGCCAGCGCCCTTGGGGTAAGTGGGCTGCTGAG

EU837255.1 ACCAATTCAGGGGCATCCGCCAGCGCCCTTGGGGTAAGTGGGCTGCTGAA

XM_009403037.2 ACCAGTATAGGGGAATTCGTCAGCGTCCTTGGGGAAAATGGGCAGCTGAA

XM_018823871.1 ACCAGTTTAGGGGAATCCGTCAGCGTCCTTGGGGAAAATGGGCAGCTGAA

XM_009419716.2 ACCAGTATAGAGGAATCCGTCAGCGTCCTTGGGGGAAATGGGCAGCTGAA

XM_009419259.2 ATCAATACAGGGGAATCCGTCAGCGTCCTTGGGGGAAATGGGCAGCTGAA

XM_009415720.2 ATCAATACAGGGGAATCCGGCAGCGTCCTTGGGGAAAATGGGCAGCTGAA

XM_009411978.2 ATCAATATAGGGGTATCCGTTGGCGTCCTTGGGGGAAGTGGGCAGCTGAA

XM_009383333.2 ATCGATATAGGGGAATCCGCCGGCGTCCCTGGGGGAAATGGGCAGCTGAA

XM_009381868.2 ATCGATACATAGGAATCCGCCAACGACCGTGGGGCAAGTGGGCAGCTGAA

XM_009405824.2 ATCAGTACAGAGGAATCCGCCAACGACCATGGGGCAAGTGGGCAGCTGAA

XM_009389352.2 GTCAATTCCGTGGAATCCGCCAGCGCCCGTGGGGCAGATGGGCAGCTGAA

XM_009391309.2 ATCAATTCCGTGGAATCCGCCAACGTCCATGGGGCAAATGGGCAGCTGAA

NM_001084343.2 GTCGGTACAAAGGAATCAGAAGAAGGCCTTGGGGAAGATGGGCGGCTGAG

* ** * * * ** * * * *

NM_001159194.1 ATCCGGGACCCGGCCAAGGGCGCGCGCGTCTGGCTCGGCACCTTCGCCAC

XM_015789203.1 ATCCGCGACCCGGCGAAGGGCGCGCGCGTCTGGCTCGGCACCTTCGCCAC

XM_015774497.2 ATCCGCGACCCGGCCAAGGGCGCCCGCGTCTGGCTCGGCACCTTCGCCAC

NM_001153709.2 ATCCGCGACCCGCGCCGCGCCGCGCGCAAGTGGCTCGGCACGTTCGACAC

NM_001254770.2 ATCCGCGACCCGCGCCGCGCCGCGCGCAAGTGGCTCGGCACGTTCGACAC

NM_001150750.2 ATCCGCGACCCGCGGCGCGCGGCGCGCGTGTGGCTGGGCACGTTCGACAC

CM007650.1_142530856-142531634 ATACGGGACCCGGAGAAGGCGGCGCGCGTGTGGCTCGGCACGTTCGCCAC

CM007648.1_220493755-220494609 ATCTGCGACCCCGTGAAGGGTGTCCGCGTCTGGCTCGGCACCTACCCCAC

NM_001111800.2 ATCCGCGACCCCGTGAAGGGCGTCCGCGTCTGGCTCGGCACCTACCCCAC

XM_023301819.1 ATCCGCGACCCCGTGAAGGGCGTGCGCGTCTGGCTCGGCACCTACCCCAC

XM_015775299.2 ATCCGCGACCCGGTGAAGGGGGTGCGCGTCTGGCTCGGCACCTACCCGAC

XM_009384249.2 ATCCGCGACCCCGTGAAGGGCGTCCGCGTCTGGCTCGGCACCTTCGCCAC

XM_009385622.2 ATCCGTGACCCGGCGAAGGGCGTCCGTGTCTGGCTCGGCACCTTCACCAC

XM_009388210.2 ATCCGTGACCCCATGAAAGGCGTTCGCGTCTGGCTCGGCACCTTCGCTAC

XM_009389661.2 ATCCGCGACCCGGTCAAGGGCGTCCGCGTCTGGCTCGGCACCTTCGCCAC

XM_009393219.2 ATAAGAGACCCGAGGAAGGGGTCGAGGGTGTGGCTGGGTACGTACGGGAC

XM_009415859.2 ATAAGGGACCCGAGGAAGGGGGCGAGGGTGTGGCTGGGGACGTACGGGAC

XM_008649541.3 ATCCGCGACCCGCGGAAGGGCGCCCGCGTGTGGCTCGGCACCTACGCCAC

XM_015782909.2 ATCCGCGACCCGAGGAAGGGCGCCCGCGTCTGGCTCGGCACCTACGCCAC

NM_001349820.1 ATCCGCGACCCGCACAAGGGCACCCGCGTCTGGCTCGGCACCTTCAGCAC

EU837258.1 ATCCGCGACCCGCACAAGGGCACGCGCGTCTGGCTCGGCACGTTCAACAC

EU847517.1 ATCCGCGACCCCGTCAAGGGCATCCGCGTCTGGCTCGGCACCTTCACCAA

XM_015775100.2 ATCCGCGACCCCGTCGAGGGCGTCCGCGTCTGGCTCGGCACGTTCGCCAC

CT833260.1 ATCCGCGACCCCGTGCGCGGCGTGCGCGTCTGGCTCGGTACCTTCCCCAC

AK067060.1 ATCAGGGACCCCCGCAAGGGCGTCCGCGTCTGGCTCGGCACGTTCGGCAC

XM_015795858.2 ATCCGCGACCCCGTCAAGGGCATCCGCGTCTGGCTCGGCACCTTCGACAC

EU847519.1 ATCCGCGACCCCGTCAAGGGCATCCGCGTCTGGCTCGGCACCTTCGACAC

FR720457.1 ATCCGCGACCCCGTCAAGGGCGTCCGCCTCTGGCTCGGCACCTTCGACAC

EU847520.1 ATCCGCGACCCCGTCAAGGGCGCCCGCGTCTGGCTCGGCACCTTCGACAC

NM_001143039.1 ATCAGGGACCCGCGGCAGGGGCGCCGCGCCTGGCTCGGCACGTACCGCAC

AK073133.1 ATCAGGGACCCCAAGAAGGGGCGGCGCGTGTGGCTCGGCACGTACGGCAG

XM_006380178.2 ATAAGAGACCCACACAAAGGTGCTAGAGTTTGGCTTGGCACATACAACAC

XM_002315454.3 ATAAGAGACCCACACAAAGGTGTTAGAGTTTGGCTGGGCACTTACAACAC

XM_008392395.2 ATTCGCGACCCCTACAAAGGCGTCCGGGTCTGGCTCGGCACCTATGACAC

XM_029095961.1 ATTCGCGACCCCCGCAAAGGCGTCCGAGTCTGGCTCGGCACCTACAACAC

XM_002272390.4 ATTCGGGACCCGCACAAGGGTGTGAGAGTGTGGCTCGGCACCTACAACAC

NM_001256464.1 ATAAGGGACCCACACAAGGGTGTTCGTGTCTGGCTCGGCACCTTCCCCAC

XM_003546320.5 ATAAGGGACCCACATAAGGGCGTCCGCGTCTGGCTCGGCACCTTCCCCAC

NM_001279196.2 ATTCGCGATCCACAGAAGGGTGTACGCGTTTGGCTTGGTACATTCAACAC

AY035100.1 ATTCGAGATCCACGAAAAGGTGTTAGAGTTTGGCTTGGTACGTTCAACAC

JF968116.1 ATCCGAGACCCTAAGAAAGGGGTTCGCGTGTGGCTCGGGACCTTCAGCAC

JF968119.1 ATCCGAGACCCTAAGAAGGGAGTTCGCGTCTGGCTCGGAACTTTCAACAC

NM_130320.4 ATTCGTGACCCGAGCAAAGGTGTACGTGTCTGGCTTGGCACATTCAAAAC

NM_001248198.2 ATTCGCGATCCACGTAAAGGAGTTCGTGTCTGGCTTGGCACCTTCAACAC

AY192368.1 ATTCGTGACCCGAGAAAAGGGGTTAGGGTTTGGTTAGGTACTTTCAACAC

NM_001247379.2 ATTCGTGACCCGAGAAAAGGGGTTAGGGTTTGGTTAGGTACTTTCAACAC

JX145122.1 ATCCGAGATCCCAGAAAGGGGGTGAGGGTTTGGCTCGGTACCTTCAACAC

XM_002301454.4 ATTCGTGACCCAAGAAAAGGAGTCCGTGTTTGGCTCGGTACCTTCAATAC

XM_002320960.3 ATTCGTGACCCAAGAAAAGGAGTCCGCGTTTGGCTCGGTACATTTAACAC

NM_001320016.1 ATTCGTGATCCCAGAAAAGGGGTTCGAGTTTGGCTCGGTACCTTCAACAC

NM_001328756.1 ATTCGTGATCCCAGAAAAGGGGTTCGAGTTTGGCTTGGTACCTTCAACAC

FQ392750.1 ATTCGTGATCCCAGTAAAGGGGTTCGGGTTTGGCTCGGGACCTTCAACAC

NM_001254494.3 ATTCGCGATCCTAGAAAAGGGGTTCGTGTCTGGTTGGGCACCTTTAACAC

NM_001254517.2 ATTCGCGATCCAAGAAAAGGGGTTCGTGTCTGGTTGGGCACCTTCAACAC

AK316980.1 ATACGTGATCCAAGGGAAGGTGCTAGAATCTGGCTTGGAACGTTCAAGAC

NM_180251.3 ATCCGTGATCCGAGAAAAGGCTCCCGAGAATGGCTTGGAACATTCGACAC

JF968115.1 ATCCGTGACCCGAGAAAGGGTGTCCGGGTTTGGCTTGGCACGTTCAACAC

JF968117.1 ATCCGTGACCCTAGAAAGGGCGTCCGGGTTTGGCTTGGAACATTCAACAC

JF968118.1 ATTCGTGACCCAAAGAAAGGTGTTCGAGTCTGGCTTGGAACTTTTGATAC

NM_001247584.2 ATACGTGATCCAAGGAAAGGTATTCGAGTCTGGCTTGGTACTTTCAATTC

KJ401124.1 ATACGTGACCCAAGGAAAGGGGTTCGGGTCTGGCTGGGAACCTTCAATAC

XM_004252354.4 ATACGTGATCCACAGAAGGGGGTCCGAGTTTGGATTGGAACTTTTAATAC

XM_002266972.5 ATCCGTGATCCAAGAAAGGGGGTTCGAGTTTGGCTTGGGACTTTCAACAC

XM_024596482.1 ATTCGTGATCCCAGGAAAGGGGTACGTGTCTGGTTGGGAACATTCAATAC

XM_006369031.3 ATTCGTGACCCCAGGAAAGGGGTACGTGTCTGGCTGGGAACGTTCAATAC

XM_008341964.3 ATCCGCGACCCAAGGAAAGGGGTTCGGGTTTGGCTTGGAACTTTCAACAC

NM_001294046.1 ATCCGAGACCCAAGGAAAGGGGTTCGGGTTTGGCTTGGAACATTCAACAC

NM_001251371.2 ATTCGGGATCCAAGAAAGGGGGTTCGTGTCTGGCTTGGAACCTTCAACAC

XM_006604868.4 ATTCGGGATCCTAGAAAGGGGGTTCGTGTCTGGCTTGGAACCTTCAACAC

NM_001354284.1 ATTCGCGACCCAAGAAAGGGGGTTCGTGTTTGGCTTGGAACTTTCAGCAC

NM_001349033.1 ATCCGTGACCCAAGAAAGGGGGTTCGTGTTTGGCTTGGAACTTTCAGCAC

NM_001155962.1 ATCAGAGACCCCAGCAAGGGCGTGCGCGTCTGGCTCGGCACATACAGCAC

NM_001157201.2 ATCAGGGACCCTAACAAGGGCGTCCGCGTCTGGCTCGGAACCTACAACAC

AK111690.1 ATCCGAGACCCCAGCAAGGGTGTCCGTGTTTGGCTTGGAACATATAACAC

XM_008660266.4 ATCAGAGATCCTCGCAAGGGCGTGCGCGTCTGGCTCGGTACTTTCAACTC

XM_015788266.2 ATCAGAGATCCTCGCAAAGGTGTCCGCGTCTGGCTTGGCACCTTCAACTC

NM_001156774.2 ATCAGAGATCCTCAGAAGGGTGTTCGTGTTTGGCTTGGTACTTTCAATAG

NM_001155219.2 ATCAGAGATCCCCAGAAGGGCGTTCGTGTTTGGCTTGGTACTTTCAATAG

EU837255.1 ATCAGAGATCCTCGCAAGGGTGTTCGTGTCTGGCTTGGCACTTTCAACAG

XM_009403037.2 ATTAGAGATCCTCGCAAGGGTGTACGCGTCTGGCTTGGGACGTTCAACAC

XM_018823871.1 ATTAGGGATCCTCGTAAGGGTGTACGTGTTTGGCTTGGTACATTCAACAC

XM_009419716.2 ATTAGAGATCCTCGCAAGGGTGTACGCGTCTGGCTCGGGACATTCAACAC

XM_009419259.2 ATTAGAGATCCTCGCAAGGGGGTTCGTGTCTGGCTTGGAACCTTCAACAC

XM_009415720.2 ATTAGAGATCCTCGTAAGGGGGTTCGCGTCTGGCTTGGAACTTTCAACAC

XM_009411978.2 ATTAGAGATCCTCGTAAGGGAGTTCGTGTCTGGCTTGGAACCTTCAACAC

XM_009383333.2 ATTAGAGATCCGTGTAAGGGAGTCCGTGTCTGGCTTGGAACCTTTAACAC

XM_009381868.2 ATCAGAGATCCTAACAAAGGAATTCGTGTTTGGCTTGGAACCTTCAACAC

XM_009405824.2 ATCAGGGATCCTCGCAAAGGAATTCGCGTCTGGCTTGGAACCTTCAATAC

XM_009389352.2 ATCAGAGATCCCTGTAAAGGGGTTCGTGTTTGGCTCGGGACATTCGACTC

XM_009391309.2 ATTAGAGATCCCCACAAAGGAGTTCGTGTTTGGCTTGGGACATTCAACTC

NM_001084343.2 ATACGTGATCCAATCAAAGGAGTTCGAGTTTGGCTCGGGACTTTCAACAC

** * ** ** * * *** * ** ** *

NM_001159194.1 CGCCGAGGCCGCCGCGCGCGCCTACGACCGCGCCGCGCGCCACATCCGCG

XM_015789203.1 CGCCGAGGCGGCGGCCCGCGCCTACGACCGCGCCGCCCGCCGCATCCGCG

XM_015774497.2 CGCCGAGGCTGCCGCGCGGGCCTACGACCGCGCCGCCCGCCGCATCCGCG

NM_001153709.2 CGCCGAGGACGCCGCCCGCGCCTACGACGCCGCCGCGGTCGAGCTCCGGG

NM_001254770.2 CGCCGAGGACGCCGCGCGCGCCTACGACGTCGCCGCGGTCGAGCTCCGGG

NM_001150750.2 CGCGGAGGAGGCTGCCAGGGCCTACGACTGCGCCGCGGTCGAGTTCCGCG

CM007650.1_142530856-142531634 GCCCGAGGAGGCAGCCCGCGCCTACGACGACGCCGCTCGCAGGTTCAAGG

CM007648.1_220493755-220494609 CGTCGAGGCCGCCGCGCGCGCATACGACCGCGCCGCGAGGCGCATAAGGG

NM_001111800.2 CGCTGAGGCCGCCGCGCGCGCGTACGACCGCGCCGCGAGGCGCATCAGGG

XM_023301819.1 CGCCGAGGCCGCCGCGCGCGCGTACGACCGCGCTGCCAGGCGCATCAGGG

XM_015775299.2 CGCCGAGGCCGCCGCGCGGGCCTACGACCGCGCCGCGCGCCGCATCAGGG

XM_009384249.2 AGCCGAGGAGGCCGCCCGCGCCTACGACCGCGAGGCCCGTCGCATCCGCG

XM_009385622.2 CGCCGAGGAAGCCGCCCGCGCCTACGACCGCGCCGCCCGCCGCATCCGTG

XM_009388210.2 CGCGGAGGAGGCTGCCCGCGCCTACGACCGCGAGGCCCGCCGCATCCGCG

XM_009389661.2 CGCCGAGGAGGCCGCCCGCGCCTACGATCGCGAGGCCCGCCGCATCCGCG

XM_009393219.2 GGCGGAGGAGGCGGCGCGGGCGTACGACGTGGCGGCGCGAGAGATCCGGG

XM_009415859.2 GGCGGAGGAGGCGGCGCGGGCGTACGATGCGGCGGCGCTGGAGATTCGGG

XM_008649541.3 CCCGGAGGACGCGGCGCGCGCCTACGACGTGGCGGCGCGCGAGATCCGCG

XM_015782909.2 CGCCGAGGAGGCCGCCCGCGCCTACGACGTCGCGGCGCGCGACATCCGCG

NM_001349820.1 CGCCGAGGACGCCGCCCGGGCCTACGACGTGGAGGCGCGCCGCCTCCGCG

EU837258.1 CCCGGAGGAGGCCGCACGCGCCTACGACGTCGAGGCGCGCCGCCTCCGCG

EU847517.1 CGCCGAGGCCGCCGCGCTCGCCTACGACGACGCCGCGCGCGCCATCCGCG

XM_015775100.2 CGCCGAGGCCGCCGCCCACGCCTACGACGCCGCCGCCCGCGACCTCCGCG

CT833260.1 CGCCGAGTCCGCCGCGCGCGCCTACGACGCCGCCGCCCGCCGCCTCCGCG

AK067060.1 CGCCGAGGAGGCCGCCATGGCGTACGACGTCGAGGCACGCCGCATCCGCG

XM_015795858.2 CGCCGAGGGCGCCGCGCGCGCCTACGACGACGAGGTTCGCCGCATCTACG

EU847519.1 CGCCGAGGGCGCCGCGCGCGCCTACGACGACGAGGTTCGCCGCATCTACG

FR720457.1 CGCCGTCGAAGCCGCGCTCGCCTACGACGCCGAGGCCCGCCGCATCCACG

EU847520.1 CGCCGTCGAGGCCGCGCGCGCCTACGACGCCGAGGCGCGCCGCATCCACG

NM_001143039.1 CGCCGAGGAGGCCGCCCTGGCGTACGACCGCGAGGCCCGCCGGATCCGCG

AK073133.1 CGCCGAGGAGGCCGCCATGGCCTACGACCGCGAGGCCCGCCGCATCCGCG

XM_006380178.2 CGCTGAGGAAGCCGCCAAAGCATATGATGAAGCCGCTAAACGCATCCGTG

XM_002315454.3 AGCTGATGAAGCAGCTAAAGCTTATGATGAAGCCGCCAAGCGCATCCGTG

XM_008392395.2 CGCTGAGGAAGCCGCCCGCGCTTACGATGAAGCCGCCGTGCGCATCCGCG

XM_029095961.1 CGCCGAGGAAGCCGCCCGCGCCTACGACGAAGCTGCCGTGCGCATCCGCG

XM_002272390.4 CGCCGAGGAAGCCGCCAGAGCCTACGACGAAGCCGCCAAGAGAATCAGAG

NM_001256464.1 CGCCGAAGAAGCCGCCCAAGCCTACGACGACGCCGCCATACGCATCCGCG

XM_003546320.5 CGCCGAGGAAGCCGCCCGAGCCTACGACGACGCCGCCAAGCGCATCCGCG

NM_001279196.2 AGCAGAAGATGCTGCTAGAGCCTATGATGAGGCTGCTAAGCGCATTCGTG

AY035100.1 GGCGGAGGAAGCTGCCATGGCTTATGATGTTGCGGCCAAGCAGATCCGTG

JF968116.1 CGCAGAGGAAGCCGCCAGGGCGTACGATGAAGCCGCCAGGAAGATCCGCG

JF968119.1 CGCCGAGGAAGCCGCCAGAGCATACGATCAAGCGGCCAGGAAGATCCGCG

NM_130320.4 CGCCGACGAAGCTGCTCGAGCCTACGACGTTGCTGCCATCAAAATCCGTG

NM_001248198.2 CGCCGAAGAAGCCGCCAGAGCCTACGACAAAGAAGCCCGAAAAATCCGCG

AY192368.1 TGCTGAAGAAGCTGCAAGAGCTTATGATAGAGAAGCTCGTAAAATCAGGG

NM_001247379.2 TGCTGAAGAAGCTGCAAGAGCTTATGATAGAGAAGCTCGTAAAATCAGGG

JX145122.1 CGCCGAAGAGGCGGCCAGAGCCTACGACAGGGAAGCTCGTAAGATCAGGG

XM_002301454.4 TGCTGAGGAAGCAGCTCGAGCCTACGACAGAGAGGCTCGCATAATCCGCG

XM_002320960.3 GGCTGAGGAAGCAGCTCGAGCCTACGACAGAGAGGCTCGCAAGATTCGCG

NM_001320016.1 CGCCGAGGAGGCGGCCCGAGCTTACGACAGGGAGGCTCGCAAAATCCGCG

NM_001328756.1 CGCCGAAGAGGCCGCCCGAGCTTACGACAGGGAGGCTCGCAAAATCCGCG

FQ392750.1 TGCGGAAAAAGCTGCGAGAGCTTACGACAGGGAGGCTCGGAAAATTCGGG

NM_001254494.3 CGCAGAAGAAGCCGCGAGAGCCTACGATCGTGAAGCTCGAAAAATCCGAG

NM_001254517.2 TGCAGAAGAAGCCGCAAGAGCCTATGATCGTGAAGCTCGAAAAATCCGCG

AK316980.1 AGCTGAGGAAGCTGCTAGAGCTTACGATGCTGCAGCGCGGAGAATCCGTG

NM_180251.3 TGCTGAGGAAGCAGCAAGAGCTTATGATGCTGCAGCACGCAGAATCCGTG

JF968115.1 TGCTGAAGAAGCTGCTCGGGCTTATGATGCCGAGGCCCGTAGAATCCGTG

JF968117.1 TGCTGAAGAAGCTGCTCGGGCTTATGATGCTGAGGCCCGTAGGATCCGTG

JF968118.1 TGCTGAAGAAGCCGCTAGGGCTTATGATTCTGAGGCTCGTAGGATCCGTG

NM_001247584.2 AGCCGAAGAGGCAGCCAGAGCTTATGATGCTGAGGCGCGAAGGATCAGAG

KJ401124.1 TGCAGAAGAAGCTGCCAAAGCTTATGATATTGAGGCGAGGAGGATCAGAG

XM_004252354.4 TGCAGAAGAAGCTGCCAGAGCTTATGATGCTGAAGCTCGGAGGATCAGAG

XM_002266972.5 TGCTGAAGAAGCTGCAAGAGCTTATGATGCTGAGGCTCGGAGGATCCGAG

XM_024596482.1 TGCAGAAGAAGCTGCTAGAGCATATGATTCTGAGGCACGAAGAATTCGTG

XM_006369031.3 CGCAGAGGAAGCTGCAAGGGCGTATGATGCTGAGGCGCGTAGAATTCGTG

XM_008341964.3 TGCAGAAGAAGCTGCAAGGGCATATGATGCCGAGGCACGCAGAATTCGTG

NM_001294046.1 TGCAGAAGAAGCTGCAAGGGCGTATGATGCCGAGGCACGTAGAATTCGCG

NM_001251371.2 TGCTGAAGAAGCTGCAAGAGCTTATGATGCTGAAGCACGGAGGATTCGTG

XM_006604868.4 TGCTGAAGAAGCTGCAAGAGCTTATGATGCTGAAGCACGGAGGATTCGTG

NM_001354284.1 TGCTGAAGAAGCTGCAAGAGCTTACGATGCTGAAGCAAGGAGGATCCGTG

NM_001349033.1 TGCTGAAGAAGCTGCAAGAGCTTATGATGCTGAAGCAAGGAGGATCCGGG

NM_001155962.1 CGCCGAGGAGGCGGCCAGGGCGTACGACGCCGAGGCTCGCAGGATACGCG

NM_001157201.2 CGCCGAGGAGGCAGCTAGGGCATACGACGCCGAAGCTCGCAAGATCCGCG

AK111690.1 TGCTGAGGAGGCAGCTAGGGCATATGACGCTGAAGCCCGCAAGATCCGTG

XM_008660266.4 CCCCGAAGAAGCTGCCAGAGCTTACGACGCCGAGGCACGCAGGATCCGCG

XM_015788266.2 TCCTGAGGAAGCTGCCAGAGCTTATGATGCTGAAGCACGAAGGATTCGAG

NM_001156774.2 TCCTGAGGAAGCTGCAAGAGCTTATGATGCCGAAGCGCGCAGGATTCGTG

NM_001155219.2 TCCCGAGGAAGCTGCAAGAGCTTATGATGCTGAAGCGCGCAGGATTCGTG

EU837255.1 TGCTGAAGAAGCTGCAAGAGCTTATGATGCTGAAGCACGCAGGATTCGTG

XM_009403037.2 TGCTGAAGAGGCTGCAAGAGCCTATGATGTGGAAGCCCGTAGTATCCGTG

XM_018823871.1 TGCTGAAGAAGCTGCAAGAGCCTATGATGCAGAAGCTCGTAGAATCCGTG

XM_009419716.2 TGCTGAAGAAGCTGCAAGAGCTTATGATGCAGAAGCCCGTAGGATCCGTG

XM_009419259.2 TGCCGAAGAAGCTGCACGAGCATATGATGCAGAGGCCCGTAGGATCCGTG

XM_009415720.2 TGCTGAAGAAGCTGCAAGAGCCTATGATACAGAGGCCCGTCGTATCCGTG

XM_009411978.2 TGCTGAAGAAGCTGCAAGAGCATATGATGCAGAAGCTCGTAAGATTCGTG

XM_009383333.2 TGCTGAAGAAGCTGCCAGAGCATATGATGCTGAAGCTCGTAAGATCCGTG

XM_009381868.2 AGCTGAAGAGGCTGCCAGAGCCTACGACGCTGAAGCCCATCGGCTTCGGG

XM_009405824.2 TGCCGAAGAGGCTGCCAGAGCCTATGATGCTGAAGCCCGCCGGATTCGGG

XM_009389352.2 TGCTGAACAGGCTGCAAGAGCTTATGATGCTGAAGCTCGTAGGATTCGTG

XM_009391309.2 TGCTGAAGAGGCTGCAAGAGCCTATGACGCCGAAGCTAGGAGGATTCGAG

NM_001084343.2 AGCTGAAGAAGCTGCAAGAGCTTATGATCTTGAAGCTAAGAGAATCCGTG

* ** ** ** ** ** * * * *

NM_001159194.1 GGGCCAAGGCCAAGGTCAACTTCCCCAACGAGGAC---------------

XM_015789203.1 GGGCCAAGGCCAAGGTCAACTTCCCCAACGAGGAC---------------

XM_015774497.2 GCACCAAGGCCAAGGTCAACTTCCCCAACGAGGACAACGCCTT-------

NM_001153709.2 GCCGCCGCGCCAAGCTCAACTTCCCGGACGCCGCC---------------

NM_001254770.2 GCCAGCGAGCCAAGCTCAACTTCCCGGCGCGCGCT---------------

NM_001150750.2 GCCCCCGCGCGAAGCTCAACTTCCCGGGCCACGAG---------------

CM007650.1_142530856-142531634 GCGCCAAGGCCAAGCTCAACTTTCCGACGACTACT---------------

CM007648.1_220493755-220494609 GAGCCAAAGCCAAGGTGAACTTCCACAGCGACACCTCCTCC---------

NM_001111800.2 GAGCCAAAGCCAAGGTGAACTTCCCCAGCGACACCTCCTCC---------

XM_023301819.1 GCGCCAAAGCCAAGGTCAACTTCCCCAACGACGCCTCCTCCTC-------

XM_015775299.2 GCGCCAAGGCGAAGGTCAACTTCCCCAACGACTTCGGCGCC---------

XM_009384249.2 GCAAGAAAGCCAAGGTCAACTTCCCCAACGAGGTACAGCCC---------

XM_009385622.2 GCAGGAAGGCCAGGGTCAACTTCCCCAACGAGGTGGAGCCC---------

XM_009388210.2 GCAAGAAGGCCAAGGTCAACTTCCCCAATGAGGTGGAGCCC---------

XM_009389661.2 GCAAGAAGGCCAAGGTCAACTTCCCCAACGAGCTGCAGCCC---------

XM_009393219.2 GGAGCAAGGCCAAGCTCAACTTCCCGAACCCCAGCGACTACGC-------

XM_009415859.2 GGAGCAAGGCGAAGCTCAACTTCCCGGACGCCGCTG---CCGC-------

XM_008649541.3 GGCCCAAGGCCAAGCTCAACTTCCCGCCCGCCGTCGGCGGCGG-------

XM_015782909.2 GCGCCAAGGCCAAGCTCAACTTCCCCCCGACCATCGGCGCCGC-------

NM_001349820.1 GCAGCAAGGCCAAGGTCAACTTCCCCGCAGCCAGCGGTCGCG--------

EU837258.1 GCAGCAAGGCCAAGGTCAACTTCCCCGC----------CACG--------

EU847517.1 GGGACAGGGCCAAGCTCAACTTCCCTTCCGCTACCACCCCTG--------

XM_015775100.2 GCGCGACCGCCAAGCTCAACTTCCCCTCCTCCTCCTCCTCC---------

CT833260.1 GCGCCAAGGCCAAGCCCAACTTCCCCTCCG----CGCCGCCG--------

AK067060.1 GCAAGAAAGCCAAGGTCAACTTCCCCGACG--------------------

XM_015795858.2 GCGGCAACGCCAAGACCAACTTCCCCCCATC-----GCCGCC--------

EU847519.1 GCGGCAACGCCAAGACCAACTTCCCCCCATC-----GCCGCC--------

FR720457.1 GCTGGAAAGCCCGGACAAACTTCCCACCCGC-----CGATCT--------

EU847520.1 GCCACAAGGCAAGGACCAACTTCCCGCCCGA-----CGAGCC--------

NM_001143039.1 GGAAGAGCGCGCGGCTCAACTTCCCGCTGCTCATCCCGCACG--------

AK073133.1 GCAAGGGCGCGAGGCTCAACTTCCCCCGCGACGGCGATGGCT--------

XM_006380178.2 GTGATAAAGCCAAGCTCAACTTC------------CCTTCTCA-------

XM_002315454.3 GAGATAAAGCCAAGCTCAACTTC------------CCCCCTCA-------

XM_008392395.2 GGGACAAGGCCAAGCTCAACTTTGCCCAACCACCATCCTCTTC-------

XM_029095961.1 GCGACAAGGCCAAGCTCAACTTTTCACAACCACCACCCTCTTC-------

XM_002272390.4 GCGACAAGGCCAAGCTCAACTTCGCTGATCC----CTCCTCTC-------

NM_001256464.1 GCGACAAGGCCAAGCTCAACTTC-----------------CCG-------

XM_003546320.5 GCGACAAGGCCAAGCTCAACTTC-----------------CCG-------

NM_001279196.2 GTGATAAGGCTAAACTCAACTTTCCAGCCCCATCACCACCAGC-------

AY035100.1 GTGATAAAGCCAAGCTCAACTTCCCAGATCTG-CACCATCCTC-------

JF968116.1 GCGACAAGGCCAAGCTGAACTTCCCTGAC---TCGGCGCCGGC-------

JF968119.1 GCGACAAGGCCAAGCTCAACTTCCCGGACACTCCGGCGCCGAA-------

NM_130320.4 GCCGGAAAGCCAAACTGAATTTCCCAAACACTCAAGTAG-----------

NM_001248198.2 GCAAGAAAGCCAAAGTCAATTTCCCCAACG----AGGAC-----------

AY192368.1 GTAAGAAAGCTAAAGTTAATTTCCCCAATG---AAGATG-----------

NM_001247379.2 GTAAGAAAGCTAAAGTTAATTTCCCCAATG---AAGATG-----------

JX145122.1 GCAAGAAAGCCAAGGTTAACTTTCCCAACG---AAGACG-----------

XM_002301454.4 GCAAGAAAGCCAAAGTCAATTTTCCTAACG---AAGATG-----------

XM_002320960.3 GCAAGAAAGCCAAAGTCAATTTTCCTAACG---AAGATG-----------

NM_001320016.1 GCAAAAAAGCCAAGGTCAATTTCCCCAACG---AAGACG-----------

NM_001328756.1 GCAATAAAGCCAAGGTCAATTTCCCAAACG---AAGACG-----------

FQ392750.1 GGAAGAAAGCAAAGGTTAACTTCCCCAATG---AAGACG-----------

NM_001254494.3 GCAAAAAGGCTAAGGTGAATTTCCCCAACG---AGGACG-----------

NM_001254517.2 GCAAAAAGGCCAAGGTGAATTTCCCCAACG---AGGACG-----------

AK316980.1 GATCTAAAGCTAAGGTGAATTTCCCTGAAGAAAACA---TGA---AGGCT

NM_180251.3 GCACGAAAGCTAAGGTGAATTTTCCCGAGGAGAAGAACCCTA---GCGTC

JF968115.1 GCAACAAAGCCAAGGTCAATTTCCCTGAGAATTTCCCTGTG---------

JF968117.1 GCAACAAAGCCAAGGTCAATTTTCCTGTGGATATTCCCGCA---------

JF968118.1 GCAAGAAAGCCAAGGTCAATTTCCCTGAGGACTCTGCCCCGG---TTACC

NM_001247584.2 GCAAGAAAGCTAAGGTGAACTTTCCTGATGAAGCTCCAGTGT---CTGTT

KJ401124.1 GCAAGAAGGCTAAGGTAAACTTTCCTGATGAAGCTCCCGCCC---CTGCA

XM_004252354.4 GCAATAAAGCTAAAGTAAACTTTCCAGATGAAGCTTCAGTGC---CTGCC

XM_002266972.5 GCAAGAAAGCAAAAGTTAATTTTCCTGATGAAACTCCTTTGA---CTGCT

XM_024596482.1 GCAAGAAAGCTAAGGTGAACTTCCCTGATGAAGCTCCATGTG---CTTCA

XM_006369031.3 GCAAGAAAGCTAAGGTGAACTTTCCTGATGAAGCTCCACTTG---CTTCA

XM_008341964.3 GTAAGAAAGCCAAGGTTAACTTCCCTGAAGAAACCCCTCGTG---CTTCT

NM_001294046.1 GCAAGAAAGCGAAGGTTAATTTCCCTGAAGAAACTCCTTGTG---CTTCT

NM_001251371.2 GCAAGAAAGCCAAGGTGAATTTTCCTGAGGCACCAGGTACTT---CTTCT

XM_006604868.4 GCAAGAAAGCCAAGGTGAATTTTCCTGAGGAGGCACCAGGTA---CTTCT

NM_001354284.1 GCAAGAAAGCCAAGGTGAATTTCCCTGATGAGCCTTCAGGCG---CTGCT

NM_001349033.1 GCAAGAAAGCCAAGGTGAATTTCCCGGATGAGCCTTCAGGCG---CTGCT

NM_001155962.1 GGAAGAAAGCCAAGGTCAATTTCCGGGACGAAGCGGCGGC---AGCAGCA

NM_001157201.2 GCAAGAAAGCGAAGGTGAATTTTCCCGATGACGCGACAG--------GCA

AK111690.1 GCAAGAAAGCCAAGGTCAACTTTCCTGATGAACCAGCTG-----------

XM_008660266.4 GCAAGAAGGCTAAAGTCAACTTCCCGGATGAGGTTCCTA------CGGCG

XM_015788266.2 GCAAGAAGGCCAAGGTCAATTTCCCAGATGGGGCTCC---------AGTG

NM_001156774.2 GCAAGAAGGCCAAGGTTAACTTTCCTGATG---CACC---------AGCA

NM_001155219.2 GCAAGAAGGCCAAGGTTAACTTTCCTGATG---CACC---------AGCA

EU837255.1 GCAAGAAGGCCAAGGTGAATTTTCCAGAGG---CTCC---------AACA

XM_009403037.2 GAAAGAAAGCTAAGGTGAACTTCCCTGATGAGGCACC---------CCCA

XM_018823871.1 GCAAGAAAGCCAAGGTGAACTTCCCTGAGGAAGCACC---------CACC

XM_009419716.2 GCAAGAAAGCCAAGGTGAACTTCCCTGATGAGGCACC------------A

XM_009419259.2 GTAAGAAAGCCAAGGTGAATTTCCCTGATGAAGCACC---------ACCG

XM_009415720.2 GCAAGAAAGCCAAAGTGAACTTCCCTGATGAGGCACC---------ACCA

XM_009411978.2 GCAAAAAAGCCAAGCTCAACTTCCCTGATGGGTCACT---------TCCT

XM_009383333.2 GCAAAAAAGCAAAGGTCAATTTCCCCGATGCAGCATT---------CCCC

XM_009381868.2 GTAAGAATGCCAAGGTGAACTTCCCGACAGGAGCAGC------ATCATTG

XM_009405824.2 GTAGGAAAGCCAAGGTGAACTTCCCAACAGCAGCAACTGCATCATCATCG

XM_009389352.2 GCCATAAAGCCAAAGTGAACTTCCCTGCAGGATCAAA---------ATCA

XM_009391309.2 GAAAAAAAGCCAAGGTCAACTTCCCCAGAGCAGCAAG---------CTCA

NM_001084343.2 GAGCCAAAGCTAAGCTCAATTTCCCTAACGAATCCTCTGGAAAG-AGGAA

* ** ** **

NM_001159194.1 --------CCGCCGC-CCGAGTACG-ACGACGACGACGACG--GCCACGC

XM_015789203.1 --------CCGCCAC-TCGA---CG-ACCCGGCCGCCGACG--GCCACAG

XM_015774497.2 ------CGCCGCCGCGCCGCCGCCGTACCACCTCGCCGCCT--ACTACGG

NM_001153709.2 -------GCCGCCGCGGCGGCGCGG-GATGTGCAGCCGC------GCCGC

NM_001254770.2 -------GCCGCCGCGGCGCCGGCG-GCAGTGCAGCCTCCGCACCACCGT

NM_001150750.2 -------GC-GCTGTTCCAG-GGCC-ACGGCGACACCGC------GGCGG

CM007650.1_142530856-142531634 -------GCTACTGCTACGACGACG-ACGACATCGCTGTCGTCGCCCCAC

CM007648.1_220493755-220494609 -------TGCGTCACCGTGCCGACGACGGCGTCGAGGTCTACTGCTGCTG

NM_001111800.2 -------AGCGTCACCGTGCCGACGGCGGTGTCGAGGTCTGCTGCTGCTG

XM_023301819.1 ------CAGCGTCACCGCGCCGACGGCGGCATCTGGG------------G

XM_015775299.2 ----------GCCCCCGCGCCGGCCGCGGCGGCGGCGAAGGCCGTCCCTC

XM_009384249.2 -------GGCGAGGTCGACCAGTCCGTCCCCTGTAACCTCGCCCGCACTT

XM_009385622.2 -------AAGCAAGTC------TCCGTC------------------GCTC

XM_009388210.2 -------GAGGAGACCGATAATTCCGTCCCCTGCAACCCCGTCCGCAATT

XM_009389661.2 -------GGCGAGGTCGACCAGCACGCGCCATTCCAGCACGTCCCCGCTA

XM_009393219.2 ------CGCAGCAGCAGCCGCAGCCGCAGCCGCAGCCGCG--GCGGAACC

XM_009415859.2 ------CGCGGGCGGGGACATGGCGGCGGTGCCACCCAAGAAGCGGAGGG

XM_008649541.3 ------CGCGCGCGCGCCGGCGCCGGCGGCGGCCAAGAGGCGCCGCAAGT

XM_015782909.2 ------CGC-CGCGCCACCGCCGCCCAAGAAGCGACGCAAAGCCGCCGCC

NM_001349820.1 -------CTCGCGGTCGCGCGCGCCCACGCCGCGGCGACGACGGCAACCC

EU837258.1 -------CCCGCCGCCGCGCGC--CCACGCCGCGGCAAC-ACGAGAGCCA

EU847517.1 -------ACACCCGCAAG-CGCGGCCGCGCCACCGCCGCCGCCGCCCCGG

XM_015775100.2 -------ACCGCCGCCAC-C----CCACGCCCCCGCAAGTGCCGCCCC--

CT833260.1 -------CCCTCGGCTGC-----TGCTCACCGCCGCAAGAAGCGCCGCGC

AK067060.1 --------CCGCCGCCGC-------CGCCCCGAAGCGGCCACGGCGTTCT

XM_015795858.2 -------CACGCCGCCGC---------CGCCGGAGAAGCCAGCGGCGGAG

EU847519.1 -------CACGCCGCCGC---------CGCCGGAGAAGCCAGCGGCGGAG

FR720457.1 -------TTCTTCGCCGC---------CGCCGCCGTCGCAGCCGCTCTGC

EU847520.1 -------TCCGCTGCCGG---------CGCCATCGCAGGCGCCGTTCTGC

NM_001143039.1 -------AGGGTCCCGGCCGTCACGCGCGCACGCCGGTGG-CCATCGACC

AK073133.1 -------CCCCTCGCCGGAGTAACGACCG-GCCCTGCTGGACCATCGACC

XM_006380178.2 ------GACACCGCCAAC------------------TCCGGAGGC-GCCA

XM_002315454.3 ------GCCACCACCAAC------------------GTCGGAGGC-GGCG

XM_008392395.2 ------ACCGCTTCCATCTC------------TGGCGCCGGATAC-GCCG

XM_029095961.1 ------ACTGCTTCCACCTC------------TGGCTCCGGCTAC-GCCG

XM_002272390.4 ------CCCACTTCCCTC-----------------CGCCCATCCC-ACTG

NM_001256464.1 ------GCCACCACCATTTC------------CG-CCGCCGCCGC-TCCG

XM_003546320.5 ------GCCAC-------------------------------CGC-TCCG

NM_001279196.2 ------TAAGCGACAGTGCA------------CTAGCACTGTCGCTGCTG

AY035100.1 ------CTCCTCCTAATTAT------------ACTCCTCCGCCGTCATCG

JF968116.1 ------GAAG------------------------AACATCGCCGCCTCTG

JF968119.1 ------GAGGCGTTGCTTGGGCGTCGCTGAGGATAACAATACCCACTCTG

NM_130320.4 -----AAGAAGAAGCCG-----------------------------ATAC

NM_001248198.2 -----GACCCTCTGCCGC---------------------------AATAC

AY192368.1 -----ACGACCA--TTAC---------------------------TGCT-

NM_001247379.2 -----ACGACCA--TTAC---------------------------TGCT-

JX145122.1 -----ATCGCTACGTTCC---------------------------TTCTC

XM_002301454.4 -----AACATTACTCCTC--------------------------CTCTCA

XM_002320960.3 -----ACCATTACTACAC--------------------------GTCCCA

NM_001320016.1 -----ACCACCTCCCCGC--------------------------CAAGAC

NM_001328756.1 -----ACGACCTCTCCGC--------------------------CCAGAC

FQ392750.1 -----ATGACTACACTGAGAA------TCACCAAAATCACCGTGCTCTAC

NM_001254494.3 -----ACGAATATTCCATTCAAGCTCGTAATCCAATTCCACCCCTTCCTT

NM_001254517.2 -----ACGAATATTCCATTCAAGCACGTCATCCGATTCCACCCCTTCCTT

AK316980.1 AATTCTCAGAAACGCTCT-------GTGAAGGCTAAT---CTTCAGAAAC

NM_180251.3 GTATCCCAGAAACGTCCTAGT----GCTAAGACTAATAATCTTCAGAAAT

JF968115.1 T---CCAACAGACGCACT-------GTCAAGGTCAAT---CACCAAAAAT

JF968117.1 T---CAAACAGATGCACT-------ATCAAGGTCAAT---CACCAAAAAT

JF968118.1 T---CAAAGCGCACTACT-------ATTAAGGTCAGT---CAAC------

NM_001247584.2 T------CAAGACGTGCT-------ATTAAGCAAAAT---CCCCAAAAGG

KJ401124.1 T------CAAGACACACT-------GTTAAGGTGAAT---CCTCAGAAGG

XM_004252354.4 T------CGAGGCAAGCT-------GGTAAGGTGAAT---CCTCGGAAGG

XM_002266972.5 C------CAAAGCGCACC-------ATCAAGGCAAAT---GCTCAAAAGC

XM_024596482.1 G------CAAGGCATCCA-------ATTAAGGAAAAC---TCACAGAAAC

XM_006369031.3 T------CAAAGCAGTCA-------ATTAAGGAAAAC---TCACGGAAAC

XM_008341964.3 G------CAAAGCGTTCT-------GTGAAGGCAAAT---TCTCAGAAAC

NM_001294046.1 G------CAAAGCGTTCC-------ATCAAGGAAAAT---CCTCAGAAAT

NM_001251371.2 G------TAAAACGTTC----------CAAGGTAAAT---CCACAGG---

XM_006604868.4 G------TAAAACGTTC----------CAAGGTAAAT---CCACAGG---

NM_001354284.1 ---TCCTCAAAACGTCT----------CAAGGCGAAT---CCAGAGGCTC

NM_001349033.1 GCTTCCTCAAAACGCCT----------CAAGGTGAAT---CCAGAGGCTC

NM_001155962.1 GGTGCTCAGAAGGCACCAGCCG-CAACTACTCCAACTGCTTGTGGCAACG

NM_001157201.2 CTCG-TCACAGGCCAACGACCGATGAGATCTTTAACAACCTGAAGAACGA

AK111690.1 -TTGCTCAGAAGCT---------------------CTCCCTGAAGCAAAA

XM_008660266.4 GTTTCTCAGAAGCGCCGTGCTG----CTGGGCCTGCCTCTCTGAAAGCGC

XM_015788266.2 GCTTCTCAGAGGAGTCATGCT-------GAGCCCTCCTCCATGAACATGC

NM_001156774.2 GTTGGTCAGAAGCACCGTTCT-------GGCTCAGCTTCTGCTAAAGCAT

NM_001155219.2 GTTGGTCAGAAGTGCCGTTCT-------AGTTCAGCTTCTGCTAAAGCAC

EU837255.1 ACTGCTCAGAAGCGTCGTGCT-------GGCTCCACCACTGCTAAAGCAC

XM_009403037.2 AGTGCTCAAAAGCACCTTATG-------AAATCAACTGCATGGGAAGCAC

XM_018823871.1 GGTCTTCAAAAGCACCTCACG-------AAATTAACTGCACCAAAAGCAC

XM_009419716.2 ATTCCTCAAAAGTGCCTTGTG-------AGACTAACTGCAGCAGAAGCAC

XM_009419259.2 AGTGTTCAAGAGTGCCCACTC-------AAATCAAATGCACCAAAAGCAC

XM_009415720.2 ATTGTACAAAAGCGCCCCCTC-------AAATCAAATGTAGCCAAAGCAC

XM_009411978.2 TGTGTCCAGAAGTCCCTTCCG-------AAACTAGCTGCATCAAGAACAT

XM_009383333.2 CGTGTCCGGACGCTCCTTACA-------AAACTGACTGCACCAGAAACTC

XM_009381868.2 AGTTCACGCAAACGCTCATCA-------AAGCCTACTGCTTCAGTAATAC

XM_009405824.2 GGTTCAAGAAAATGCTCGACA-------AAGCCTACTGCTTCAGAAATTC

XM_009389352.2 GGTGCTAAAAAAAGCATACTG-------AAACCTACTGCTCCAAGAACTC

XM_009391309.2 GGTTCCAAAAAGATCATCCCA-------AAACCGACTGCTCTAAGAACAC

NM_001084343.2 AGCCAAGGCTAAGACTGTGCAACAGGTAGAGGAGAATCATGAGGCTGATC

NM_001159194.1 CCACGCCCAC----------GCCGCCGCG---GCGCAGGGGATGCTCGCC

XM_015789203.1 CCACGGCGGC----------GCCGCCATC---CCGTGCAGGGAGTTCATG

XM_015774497.2 CGACGCCTCC----------TCCACCTCCTACCTCTACCCGATGGCCATG

NM_001153709.2 CCGTTGCCCG----------GTCAGAGCCTCCGCGAGAACTGCGGGTCCA

NM_001254770.2 CCATTGCCCG----------ATCGGAGCCTCCGCGAGAACTGCGGATCCA

NM_001150750.2 CGAACACCGA----------GACACAGACGCCGTCG-----------CCG

CM007650.1_142530856-142531634 CAGCTGCTGC----------GGT-CAGCCGCGGAGTCGGAGACGGCGGGG

CM007648.1_220493755-220494609 CCGCCGCACA----------GGCCCCGGCCGTT---CTCCCACCGCCTAA

NM_001111800.2 CTGCTGCACA----------GGCCCCGGCCGTT---CTCCCGCTGCCCAA

XM_023301819.1 TGGCGGCACA----------GGCCCCGGCCGTTTTTCTCCCGGCGCCCAA

XM_015775299.2 GCGTCGCGCC----------CACGCCGGCCGTG---CTCCCGCCGCCCAA

XM_009384249.2 CCATGGAACC----------CAAGCTGGAAAAAG--CCCCCGCCTTGGCG

XM_009385622.2 TCGTCGAACC----------CAAGCCGGAA--------------------

XM_009388210.2 TCATTCAACC----------CAAGGTGGAAAAAT--CTCCCGTCTTGGCG

XM_009389661.2 TTGTCGAACC----------CAAGCTGGAAGAGG--CCCCGGC-------

XM_009393219.2 CCC-CACGAA----------GCGGCGAGCCGCTG---CTTGGGCGGAGGA

XM_009415859.2 CC-----GGG----------GCCGCGGAG----G---CAGGGTCGGGAGA

XM_008649541.3 CC-----GCG----------GCGACCGAGGAGAG---CTCGGCCTCGTCT

XM_015782909.2 GCGGCGAACC----------ACCACCACCACCAC---CACCAGCAGGAGA

NM_001349820.1 ACGAACCGCGCCGGAAACGCAGCACCCAGCACAGCCCGCTCTGCTGCCTC

EU837258.1 CCG--CCGTGCC--------ACCGCCGGCGACAGCACCCGCCGCCGCCCC

EU847517.1 CCG--TCAAGGCG-------ACCCCGGTCATCAACCTCGTCGAGGAGGAG

XM_015775100.2 --------------------ACCACCGCCACCG-CCACCCCCAAGGCGAC

CT833260.1 CCACGCCGCCACGCG--CTCGCCGTCGTCTCCGCCCGCCACCAGCGAGGT

AK067060.1 TCGG--CGAAGCA-------TTCGCCGCAGCAGCAGAAGGCCAGGTCGTC

XM_015795858.2 AGGAGCCCC------------TCGACGACGCCGACGACGACCACGGAGGA

EU847519.1 AGGAGCCCC------------TCGACGACGCCGACGACGACCACGGAGGA

FR720457.1 TTC--TTGC------------TCAACGACAACGGCCTCATC-ACAAT---

EU847520.1 TTC--CTGC------------TCGACGACGACGACGACGACGACGGCGTG

NM_001143039.1 TCAACTTGCC------GCCCGTCTCCGACGGTCTCGGCGTCCCGGCTCCG

AK073133.1 TCAACCTCCCCGCGGCGGCCGTCTCCGGTGACGACGACGACGCCATGGCC

XM_006380178.2 CCACCTGCCAAGAAG--CGTTGCATCGTGGCTCCTGAAACAGCT--TATG

XM_002315454.3 CCACC-ACCAC-----------CACCAAAG--TCGGAGGCGGC-------

XM_008392395.2 CCGCCGACAAAGAGG--CGGTGCATTGTTG---CTGAGTCAACTCGGGTG

XM_029095961.1 CCGCCGACAAAGAAG--CGGTGCATTGTTG---CTGAATCAACTCGGGTG

XM_002272390.4 CCGCCATCCAAAAGA--CGATGCGTTGTTC---CAGACCCCAC---AGCA

NM_001256464.1 CCACCTTCCAAAAAG--CAACGCTGCCTCAGCCCTGACATCATC------

XM_003546320.5 CCACCCTCCAAAAAA--CAACGCTGCCTCAGCCCTGACACCACC------

NM_001279196.2 CTGATACACCACCAG--CACTACTCCTTGAGAGTTCTGACAACTCTCCTT

AY035100.1 CCACGATCAACCGAT--CAGCCTCCGGCGAAGAAGGTCTGCGTT------

JF968116.1 CTGAT---AAGGAGT--CGAGCCGG------------CCGAGTTT-----

JF968119.1 TTGCTGTGAACGAGT--TGACTCGGACGAGTCACCCTCCGAGTTTGCCTA

NM_130320.4 TAAACCAGGGGG-------------------GAATCAAAAT--GAGCTGA

NM_001248198.2 GGATCTTGCAAG-------------------AGCCTCAACTTGGAGTTCG

AY192368.1 ----------------------------------------------ACAG

NM_001247379.2 ----------------------------------------------ACAG

JX145122.1 AACCCTGCCAGC-------------------CGTCCTTTTCAATGCACAA

XM_002301454.4 AAATTCAA-ATA------------------CCAAGCCTCCTATGTACCAA

XM_002320960.3 AAACTCGA-ACA------------------CCAAGCCTTTCCTGTACCAA

NM_001320016.1 GTATCTGA-GAAA---------------CCCAAATACTAATCTGTATCAA

NM_001328756.1 CTATCTGA-AAAA---------------ACCAAATCCTCCTCTGTTTCAA

FQ392750.1 CAACTCGCTGGAA---------------CTCCAATCCTTCTATTTGTCAG

NM_001254494.3 TCGCTCCACAAC---------------------ACCCTCCCCTGTACCAG

NM_001254517.2 TCACACCACAAAC---------------ACAAAACCCTCCCCTGTACCAG

AK316980.1 CAGTGGCTAAAC---CTAAC-----CCTAAC---------CCAAGTCCAG

NM_180251.3 CAGTGGCTAAAC---CAAACAAAAGCGTAAC---------TTTGGTTCAG

JF968115.1 CGAGTTCCAACT---GCAAGTCAGAC--------------TCGAACCCTG

JF968117.1 CAAATGCAAATG---CAAAGACAAAC--------------CAAAAGCCAG

JF968118.1 --------------------------------------------------

NM_001247584.2 CACTTCGTGAGG---AAACCCTGAACACAGTTCAGCCCAACATGACTTAT

KJ401124.1 TCCTTCCTGAGG---AGAGCCTGTATTCACTTCAGTCCGACTCAGCAATC

XM_004252354.4 TTCTTTCTGATG---AGAGCTCTAACCCAGTTCCACCCAACACCATGCTT

XM_002266972.5 TAATTCCAAAGG---GTAGCCCTAACTCTTCCCATTCGAATCTGAACCAG

XM_024596482.1 GACTTACGAAGG---CAAA---------------------TTTAAGCCAG

XM_006369031.3 CACTTCCAAAGA---CAAA---------------------TTCAAGCCAG

XM_008341964.3 TGATACCCAAGA---CAAACGTGAATGGCACTGAGTCTAATCTGAACCAG

NM_001294046.1 TGATAGCCAAGA---CAAACTTGAATGGCACTCAGTCTAATCCGAACCAG

NM_001251371.2 ---------------AAAATCTTAAGACTGTTCAGCCCAATCTGGGTCAC

XM_006604868.4 ---------------AAAATC---------------------TGAGTCAC

NM_001354284.1 AGCCAATGAAGA---AAAATCTGAACTCTGTGAAGCCGAAAATAAACCAG

NM_001349033.1 AGCCAATGAAGG---AAAATCTGAACACCGTGAAGCCGAAAATGAACCAG

NM_001155962.1 CAGCGAGGCTAGGTCCTCCACCTCCACCGCCGAAGTTCCGTGCTGACGAG

NM_001157201.2 CGACAACAACA--------ACAACGATGATCTGTTTGCGATGTTTGCGT-

AK111690.1 CGCTGCCA-----------AGCAAGAGAAACTAGCTCCACCTCTGAAGA-

XM_008660266.4 CTAAGATGGAC---------GTTGAGGAGGAGAAGCCGATCATCAAGCTC

XM_015788266.2 CTGCTTTCAGC---------ATCGAAG---AGAAGCCGGCCGTCATGTCA

NM_001156774.2 CCAAGTCAAGT---------TTTGGAC---AGAAGCCTATCGTCAAAGTA

NM_001155219.2 TCAAGTCATGT---------GTTGAAC---AGAAGCCAATTGTCAAAACA

EU837255.1 CCAAGTCAAGT---------GTGGAAC---AGAAGCCTACTGTCAAACCA

XM_009403037.2 CGACATGGAAC---------C---CAGAAGATAGTCTCAATTTTAATCAC

XM_018823871.1 CAACATCGGAC---------CGCCCACAAGAAAGTCTCAACTTCAACCAG

XM_009419716.2 CAAACTCAAAC---------C---CAAGAGATACCTTCAACTCTAACCAT

XM_009419259.2 CCAAGTCAAAC---------C---CACAAGAGATGCTCAATGTCAACCCA

XM_009415720.2 CTAAACTGAAC---------C---CATCAGAGAAGCTTAACTTCAGCCAA

XM_009411978.2 CAA----AAAC---------A--------GAGATGCCCAACTATAATAAG

XM_009383333.2 CAA----GAAC---------A--------GAGATGCCCAGAACTGATAAG

XM_009381868.2 CAAAACCAAAG---------G---TGTTGGAAAAACTTGACTGCAACCAG

XM_009405824.2 CAGAAATAACT---------G---CATTGGAAAAACTTGACTTCGACCAG

XM_009389352.2 ACACATCAAAC---------A---TGTCAGAAAAGCTTGAGTGCAAGCAT

XM_009391309.2 ATGTGCCAAAT---------C---CGTCAGAAAAGTGTGAATTTAAGCAG

NM_001084343.2 TTGATGTGGCGG---------TGGTAAGCTCAGCGCCTAGTAGTAGCTGT

NM_001159194.1 ATGTC-CGCCGGC-----G--GAGGCAGGGA-CGACCACCTAGTCGACTA

XM_015789203.1 GACTA-CGACGCC-----GTCATGGCGGGCTTCTTCCACCAGCCCTACGT

XM_015774497.2 ACGCC-CGCCGCC-----GCCGGACTGAGGGAGCAGCAGCTGAT-GACGA

NM_001153709.2 ACGCCGCGTCGCC-----GCTGCACGTGGCGGTGGCGCGGGCGCCGACCA

NM_001254770.2 ACGCCGCGTCGCC-----GGTGCACGTGGCGCTGGC------GCCGACCA

NM_001150750.2 TCGCC--GTCGCC-----GGGAGA-GTGGCAGCTG-------GGCGGCGG

CM007650.1_142530856-142531634 GAGGAGTTCCCCG-----ACCTCGGGCAGTACATGCACATACTCCAGAGC

CM007648.1_220493755-220494609 GGTGGAGGCAC------------AGACTGAGCTG--------TCCGACGA

NM_001111800.2 GGTGGAGGCAC------------AGACTCAGGTG--------TCCGACGA

XM_023301819.1 GGTAGAGGCGC------------GCGCACAGGTG--------TCCGACGA

XM_015775299.2 GATGGAGGCGGTG-----TCCGAGGGCGCCGGCGCCTGCTCCTCCGACGA

XM_009384249.2 G------------------------ACGGCGGCG---------ACGCCGA

XM_009385622.2 G------------------------GCGATGGCG---------ACGGCGA

XM_009388210.2 GCTTCCAATGA------------CTGCGAGGGAG---------ACGGTGA

XM_009389661.2 --------------------------CAACGGGG---------ATGCCGA

XM_009393219.2 GGAGAGCT---------------CGGCGTCG------TGCTC--------

XM_009415859.2 GGTGAGCT---------------CGGCGTCG------TACTGTGCGGCGC

XM_008649541.3 CCTCCTCCTC-------------CGGCGGCGGCGG--CGGCCGTCGTCGT

XM_015782909.2 GCTCAGGCTCCT--------CGTCGGCGTCGTCGC--TGCCTCCCACC-C

NM_001349820.1 GAGGAGAGAGAGAG----ACGCAGAGGAAGGAAGGGATCGCCGCCGTGAA

EU837258.1 GCCG---------------CGCGGACTGAAGCGAGAATTCTCGCCGCCTG

EU847517.1 GACGAGGAGG-AGG----TCGCCG-CCGCCATGGCGTCCATCAAGTACGA

XM_015775100.2 GACACCGA---ACG----TCGTCG-TCGTCGTCAACCTCGTCGACAAAGA

CT833260.1 CACGGCGG-----------CGTCCGCGTCCGCGTCCAGCGATGTCCCCGC

AK067060.1 GTCGTCGT-----------CGCCGGCGAGCCTGAACGCCAGCGACGCCGT

XM_015795858.2 CTCCGGCGAC--------TCGCGCATACTCATCGAGTGCTGCTCCGACGA

EU847519.1 CTCCGGCGAC--------TCGCGCATACTCATCGAGTGCTGCTCCGACGA

FR720457.1 ---CGGAGAA--------GCG-------CCGAC-----CGACGACGCCGC

EU847520.1 GCCCGTGGAA--------ACAG-----CCCGGCGTCGTCGTCGGCGCCGG

NM_001143039.1 GCTGGCGTCG---------GCGACGACGGCACGGCGAATGCCGACGGAGA

AK073133.1 GTCGACGCCG---------CAGACGCAGACGCAGGCAGTGCTGGCCGTGC

XM_006380178.2 TGGCTAGT-CCTTT----CACACCACCGCTGCAAGAGCCATATTCTGGGT

XM_002315454.3 --GCCACC-ACCTG----CCAAGAAACGTTGCA---------TCTTGGGT

XM_008392395.2 GAGCCGAC-TCAAC----CGAGTTTCCAGACCG-GTTCTTACTATTATGA

XM_029095961.1 GAGTCGAC-TCAGC----CGAGTTTCCACACCG-GTTCTTACTATTATGA

XM_002272390.4 CAGTCAAG-TTA-T----CAGCCGACCGTACCA-CCACCACCATTAATGA

NM_001256464.1 ------AC-TGAAG----AAAGCAGCAGCAGCA--GCTCACATTCCACCA

XM_003546320.5 ------AC-CGAAC----AAAGCAGCAGC--------TCACAATCCACCA

NM_001279196.2 TGATGAAC-TTTGG----ATATGATGTCCAGTA---TCAGAGCCAAACTC

AY035100.1 --GTCTCT-CAGAG----TGAGAGCGAGTTAAGTCAGCCGAGTTTCCCGG

JF968116.1 -CGCTGATGTCGGG----CTGGAGTACGGGTACGGGCTCGGGGAAACGGG

JF968119.1 ACGTGGACGCTGGG----CTCGGGTACGGGCACGGGCACGGAGAAACGGG

NM_130320.4 TTTCGGAA-----------------------AACCAAGTAGAGAGCTTA-

NM_001248198.2 GTTACGATCT--------------------CAACCAAACAGGGCTTTTCC

AY192368.1 TC-----------------------------ATCCAGAGCCCCCTCCCT-

NM_001247379.2 TC-----------------------------ATCCAGAGCCCCCTCCCT-

JX145122.1 TCCTAATCCT--------------------AATCCCAACCCACCTTCTTA

XM_002301454.4 AC-CCCCACT-----------------TGTCATTTCTCAAAAGGTTATGA

XM_002320960.3 GC-CCCCACT-----------------TGTCATTTCTCAAAAGGTTATGA

NM_001320016.1 CC-CAAAAGC-----------------TTTGATTTGGGATTTGGGTATGA

NM_001328756.1 CC-CAAAAGC-----------------TTTGAATTGGGATTTGGGTATGA

FQ392750.1 CC-CTATACC-----------------CCAAATTTCTCAAAAAGTTTGGG

NM_001254494.3 CAACAGTACCG---------------TTGCGATCTCAACAATGCCCCTAA

NM_001254517.2 CAACAGTACCG---------------TTGCGATCTCAACATTGCCCCTAA

AK316980.1 C----------TTTGGTTCAGAA---------------CTCGAA---CAT

NM_180251.3 CAGCCAACACATCTGAGTCAGCAGTACTGCAACAACTCCTTTGA---CAA

JF968115.1 CACCTCAGAATTGGAACCAG----------AGCTACAACAACAACGTTGA

JF968117.1 CACCTCAGAATTGGAACCAG----------AGTTACAATAACA---TTGG

JF968118.1 --------------------------------------------------

NM_001247584.2 A---TTAGTAACTTGGATGG---TGGATCTGATGATTCGTTCA------G

KJ401124.1 A---TGAACAGCGTGGAGGA---TGACCATTATGATTCTTTTG------G

XM_004252354.4 A---TGAACAACTTGAATAG---TGGATATTGTGACAATGTGG------G

XM_002266972.5 AGTTTTAATTTCATGAACAACTCTGATCAGGAATACTATAGTA------G

XM_024596482.1 GATTTTAGTTACTTGAGCAACCCAGAAACGGATTATAATAATA---TGGG

XM_006369031.3 TCTTTCAGTTACTTGAGCAACCCAGAACTGAATTATAATAATA---TGGG

XM_008341964.3 AATTTCAATTTTGTGAACGACTCAAGTCAGGACTATTACAGTGCTCTGGG

NM_001294046.1 AATTTCAATTTTGTGAACGACTCAAGTGAGGACTACTACAGTGCTCTGGG

NM_001251371.2 AAGTTCAGTGCTGGCAACAATC---------AC---------A---TGGA

XM_006604868.4 AAGTTCGGTGCTGGCAACAATC---------AC---------A---TGGA

NM_001354284.1 ATGTTCAATTTTGGTGACAATCTTGAGGGCTACTACAGCCCTA---TAGA

NM_001349033.1 ATGTTCAATTTTGGTCACAATCTGGAGGGCTACTACAGCCCAA---TAGA

NM_001155962.1 GTTTTCGGGAACATGAACGGTGGCACTGGCAGCAACGATCTGTTCGCGAT

NM_001157201.2 -TCGGTGACAATAAGAA-GAAGGTTCCTGCAGCGAAG--CCAGCCGCCGC

AK111690.1 -CCTGTGGCGATGATGC-----TTTCTTTCAGCTAAA---CAGTTCAGAC

XM_008660266.4 GCAGTGAACAA------------TATGACCAACTCAAACGCATATCACTA

XM_015788266.2 GCAGGCAACAAAAC---------CATGTACAACACAAATGCTTATGCCTA

NM_001156774.2 GCTATGAACAA------------CCTTGCCAACACAAATGCATCCTTCTT

NM_001155219.2 GATATGAACAT------------CCTTGCCAACACAAATGCACCCTTCTA

EU837255.1 GCATTCAACAA------------TCTTGCCAATGCAAATGCGTTTGTCTA

XM_009403037.2 AATTTCAATTA------------TGTGAATGATGATAATCATGATTTTTT

XM_018823871.1 TCTTTCGGTTG------------CCTGAATGATATTGATCATGATATCTA

XM_009419716.2 TCTTTCACTTA------------CCTGAATGACACCAATCAAGATTTCCA

XM_009419259.2 TATGGGGGTA---------------TGGATGACCCAGATTTAGATTTCTG

XM_009415720.2 TTTACAAATTA------------CCTGAATGAACCAGATCAAGATTTCTA

XM_009411978.2 TGCTTCAATCA------------CCTCTATGACCCATTTCAAGATTTCTG

XM_009383333.2 TGCTTCAATTA------------CTTGAATGACCCATTTCAAGATTTCTG

XM_009381868.2 AATTCCAACTA------------TCTTTGTGACCAAGATTCTGAACTCTA

XM_009405824.2 ACATCCAAGTA------------CTGGAGTGACCATGAGTCTGAA---TG

XM_009389352.2 ---TCCGACCA------------TCCGAATGACCAAGATTGTGTTTTCTA

XM_009391309.2 ---TCCAACCA------------TCAGAATGATCAGTGTTGTGATCTCTA

NM_001084343.2 CTTGATTTCTTGTGGGAGGAGAATAATCCGGACACGCTTCTGATTGATAC

NM_001159194.1 CGACGTCGTCGACGTCA--TGGGCATG--GGCGGCTTCTTCCAGCACCAC

XM_015789203.1 CGTCGCCGACGGCGTGC--CGGCCGTGCCGGCGGAGGAGGCGCCCACGGT

XM_015774497.2 CGACGGCGGTGGAGTACAGCGTTAATGACGCCGTCGACGTGGCCAGCGTT

NM_001153709.2 CGCTGCAGGGAACAGGGCCGGCGCCGCCCAAGGACCAGGACATCTGGGAC

NM_001254770.2 CGC------------------------------AACAGAATATCTGGGAG

NM_001150750.2 AGC----------------------------AGGAGACGAGCTGTGGGAA

CM007650.1_142530856-142531634 AGCAGC--------------------------GACGCCGACGTTCGGGCC

CM007648.1_220493755-220494609 GGT-CAAGGAGTTGTCCGAGGAGCTGATGGCCTATGAGAACTACACAAAC

NM_001111800.2 GGT-CAAGGAGTTGTCCGAGGAGCTGATGGCCTACGAGAACTACATGAAC

XM_023301819.1 GGT-CAAGGAGCTGTCCGAGGAGCTAATGGCCTACGAGAACTACATGAAC

XM_015775299.2 GGT-CAAGGAGCTGTCCGAGGAGCTGCTCGCGTACGAGAACTACATGAGC

XM_009384249.2 GGT-GAGGAGGCTGTCGGAGGAGCTCATGGCTTACGAGTGCTACATGAAC

XM_009385622.2 GGT-GAGGAGGCTCTCGGAGGAGCTGATGGCTTACGAGTCGTCCATGAAC

XM_009388210.2 GGT-GAGGAGGCTGTCGGAGGAGCTGATGGCCTACGAGTCCTACATGAAC

XM_009389661.2 GGT-GCGGAGGCTGTCGGAGGAGCTAATGGCTTACGAGTCGTACATGAAC

XM_009393219.2 ----GGCGTCGCTGGAGGAGGGGCTGCGGGAGCGGATCTCGAGCTTGGAG

XM_009415859.2 CGGAGGCGTCGTTGGAGGAGGGGCTGCGGGAGCGGATCTCGAGCTTGGAG

XM_008649541.3 GGCGGGCGGCGG-GGAGGAGGCGCTGCGCGACTGCATGTCTGGGCTCGAG

XM_015782909.2 CGCCGCCGGCCGCCGAGCACCAGCTCCGCGAGTGCATGTCCGGGCTGGAG

NM_001349820.1 GCCAGAAGCTACGGAGTCGTTCGACGTGGGCGGCGGTCTCTTCTTCGACA

EU837258.1 --CTGAGACCGCGCTACCTTTCTTCACCAACGGC------TTCGTCGACC

EU847517.1 GCCCGAGACCAGCGAGAGCTCCGA-GTCG-----AACGCCCTCCCGGACT

XM_015775100.2 GGCCGAGGTCAGCGAGAGCTCCGGTGCCAGCAGCAGCGCGCTGCCGGACT

CT833260.1 GCCGGCGTTCGCTTCCTTCGTCGGCGAGCCCGGGCACGGCGGCGCCAAGT

AK067060.1 GTCCAAGTCCAACAACAACCGCGTCAGCTCGGCTGGGAGCAGCACCGACG

XM_015795858.2 CCTGATGGACAGCCTCCTCGCCGCCTTCGACA-TGACC--ACCGGCGACA

EU847519.1 CCTGATGGACAGCCTCCTCGCCGCCTTCGACA-TGACC--ACCGGCGACA

FR720457.1 GTCGA-----CGT-----CGACGTCGACGACGGAGGCG--TCCGGCGA--

EU847520.1 ACAGAGCCTCCGCTTGCACGACGTCGTCGACGGTGGCG--TCCGGCGAGC

NM_001143039.1 TGCAGA-TATGGCCAGTACTAAGACTCAGAGCACGCTA---GCGCGGGTC

AK073133.1 AGCAGC-CTATGCAGATCAAGAAGCACTGAGCGCGGCAA-AGTGCAAGAT

XM_006380178.2 ATCAAAATGAAGATTATGAGCTGGAA-----GAGCAAATCTTGAAGTTGG

XM_002315454.3 CCTGAAACGGCTGCTATG-GCTAGTTTT---GAGCAAATCTTGAACTTGG

XM_008392395.2 TCCATTATATCACGGCGGTGGTGGTGGGGAAATGTATGCTAAGAAAGAGG

XM_029095961.1 TACATTTTATCAGGGTGACGGTGAT------ATGTATGCTAAGAATGAGG

XM_002272390.4 CCTATCCTGCTCCGTACGAGAACGAAGCAGCGAGCGAGTTGGAGTTTAAG

NM_001256464.1 CTGGATCCACCGG-------------------CGAAAGCGGCGGAGGAAA

XM_003546320.5 CTGGATCCACCGGATCGCCGCCTTCCGC----CGCCTTCCACGGCGGAGG

NM_001279196.2 CCTACTACCCCATGGAAATGCCCATAGTTAGTGAAGATTATGAACTGAAG

AY035100.1 TGGAGTGTATAGGATTTGGAAATGGGG----ACGAGTTTCAGAACCTGAG

JF968116.1 TCGGATGGACCCGATTTGGAACCTGGAGAGGTGGCTGGGACTGGAGGAAG

JF968119.1 TCGGGTGGATCCGATTTGGAACCTGGAGAGCTGGTTGGGACTGGACGAAG

NM_130320.4 --TC-----------GGAGGAC-CTGATGGCATTGGAGGATTACATGAGA

NM_001248198.2 CTTC-----------TTCTAACGCTGATGAGAATTGCGGGTCCCACGTGG

AY192368.1 ------------------TGAACATTGCTTGTGATACTACT-------GT

NM_001247379.2 ------------------TGAACATTGCTTGTGATACTACT-------GT

JX145122.1 TCCGAC---------GTTTAACCATTTCGGGGCATACAACTCAAATGGGT

XM_002301454.4 TTT------------TGGGTACGGTTCT----GGTATGAATCAGGTTGAA

XM_002320960.3 TTC------------TGGGAACGTTTAT----GATGCATATCAGATCGAA

NM_001320016.1 TCTGAACCAG----ATTGCGACATTTCCCTCCAATTCCATGTCGATTGAG

NM_001328756.1 TCTGAACCAG----TTTGCGGCATTTCCCTCCAATTCCAATTCAGATGGG

FQ392750.1 TTTTGCTGATC---ACTTGAACCAGATTGGAGCATTCCCCTCCAA-TGGG

NM_001254494.3 AAATCTCAACT---TTGAGTTCGGTTACGACCTGAACCACGCGGAGGCGT

NM_001254517.2 AAATCTCAACT---TTGAGTTCGGGTACGACCTGAACCACGCGGGGGCAT

AK316980.1 CTCCTTTGA---AAATATGTGTTTCATGGAGGAGAAACACCAAGTGAGCA

NM_180251.3 CTCTTTTGG---TGATATGAGTTTCATGGAAGAGAAGCCTCAGATGT---

JF968115.1 ATTTGTTGC---AGAGGAGGCTCTTACT---TACACCAACTCCTTAGCTG

JF968117.1 ATTTGTTGC---AGAGGAGCCTCTAAAT---TATACCAACTCCTTAACTG

JF968118.1 --------------------------------------------------

NM_001247584.2 TTTTTTCGA---AGAGAAACCAGCAACCAAGCAGTACGGCTTCGAGAATG

KJ401124.1 ATTTTTTGA---AGAGAAACCCATGACAAAACAGTATGGATATGAGAATG

XM_004252354.4 CCTGCTCGA---AGAGAAAACAAAGACT---CTGAATGGCTACGAAGCTT

XM_002266972.5 TTTAATGGA---TGAGAAACCATCAACCAATCAATATGGGTATCCAGATT

XM_024596482.1 CTTTGTGGA---AGAGAAACCACAAGTGAGCCAGTTTGGATTAATGAATT

XM_006369031.3 CTTTGTGGA---AGAGAAACCACTTGTTAATCAGTTTGGATCAATGAATT

XM_008341964.3 TTTTCTGGA---TGAAAAGCCAACATTGAATAACTTTGGGTATATGTCTA

NM_001294046.1 TTTTCTGGA---TGAAAAGCCAACAATGAATAACTTTCGATACATGTCCA

NM_001251371.2 TCTGGTGGA---ACAGAAACCCCTAGTTAGCCAGTATGCTAACATGGCTT

XM_006604868.4 TCTGGTGGA---ACAGAAACCCCTAGTTAATCAGTATGCTAACATGGCTT

NM_001354284.1 TCAGGTGGA---ACAGAAACCACTGGTTAACCAGTATGTTAACCGTGCCC

NM_001349033.1 TCAGGTGGA---ACAGAAACCACTGGTTAACCAGTATGTTAACCCTGCCC

NM_001155962.1 TATGTTCGCGTTCAGTGACAGTAGTAGTAAGGTCGTTCGTGTGGAGCCAG

NM_001157201.2 CGAGGGTGG---CAGTGGCAGTGGCAGCTTCCTCGTCCCTGCGCCTGC-G

AK111690.1 AATGATT-----TGTTTGCAATGCTTGCAAAG--GTGCCTGCAAAGCCGG

XM_008660266.4 CCCTGCCGT---CGTCGGCCACAACATCATACCCGAGCCATTCATGCAGA

XM_015788266.2 CCCTGCTGT---TG------AGTACACCTTACAGGAGCCATTTGTGCAGA

NM_001156774.2 CCAATCTGC---TA------GCTACCCCTC---CAATTTATTTGTTCAGC

NM_001155219.2 CCAATCTGT---TA------ACTACGCATC---CAA--------------

EU837255.1 CCCATCTGC---TA------ACTTCACTTCAAACAAGCCGTTTGTTCAGC

XM_009403037.2 CATGACTTT---GG---ATTTGTTTGAGGAGAAGGAACCCATCAAACACT

XM_018823871.1 CTCGACTTT---TG---ATCTGTTTGAGGAAAAGGTGCCCATCAAACAAC

XM_009419716.2 CCCAGATAT---TG---ATTTGTTCGACGAAAAGGTGGCCATCAAGGAAT

XM_009419259.2 TTCTGTTTT---CG---ACTATATTGATGACAAGGAACCCGTCAAGCAAT

XM_009415720.2 CTCTGCTTT---TG---ATTTCATCGATGATAAGGGACCCATCAAGCAAT

XM_009411978.2 CTCATCTTT---TG---ACTTCATCGAAG---TGGATCCCATCATACAAT

XM_009383333.2 CTCTAGTTT---TG---ACTTCACTGAAG---TGGAGCCCATTATACAAT

XM_009381868.2 TTCAACGTT---------CATGGAAGATC---AGGAACTTACGAAGCCTG

XM_009405824.2 TTCAAGGTT---------CATGATAGACA---GGGAACTAACCAAGCCTG

XM_009389352.2 TTCTGCTAT---GG---GCCTCTTGGAGG---AGGAACTGACCAAGCCTG

XM_009391309.2 TTCTACTAT---GA---GCCACTTGGAAG---AGGAGTTGGCTAAGCCTG

NM_001084343.2 ACAATGGCTCGAAGATATCATCATGGGCGATGCGAATAAGAAACATGAAC

NM_001159194.1 G-CGTACGTGCCCGACGCCGTGGCGCAGCAGGAG-----CAGG--TGCCC

XM_015789203.1 GGCGTACGTGCACCACCACCTGCCGCCGCAGCCG-----CAG-------C

XM_015774497.2 TACTTCCAGCCGCCGCCGCCGGCGGTTGCTTACGAGT-TCAGCGCCGTCG

NM_001153709.2 GGCTTGAACGAGATCATGACGATGGATGACG-------GCAG------CT

NM_001254770.2 GGCTTGAACGAGATCATGATGATGGAGGAGGAAC----GCAG------CT

NM_001150750.2 GGTCTACAGGACCTGATGAAGCAGGACGAGG-------TGGA------CC

CM007650.1_142530856-142531634 GTCGCGGCCGGGTTGCCGCTGATGAACCGCC--T----GCCG------CC

CM007648.1_220493755-220494609 ---TTCCTCGGCATCCCTTACATGGAGGGAGGGA----CCACA---GCTG

NM_001111800.2 ---TTCCTCGGCATCCCTTACATGGAGGGAGGGA----CTACA---GCTG

XM_023301819.1 ---TTCCTCGGCGTCCCGTACATGGAGGGCGGGA----ATGCA---GCTG

XM_015775299.2 ---TTCCTCGGCATCCCCTACATGGAGGGCGGCG----CCGCCTCCGCCG

XM_009384249.2 ---TTCTTCGGGATCCCGTACATGGAGGGCGGAT----CTGCCGCCGAAG

XM_009385622.2 ---TTCTTCGGTATACCATACATGGAGGGCGGAG----CCGCAGCTACGG

XM_009388210.2 ---TTCTTCGGTATCCCGTACATGGAGGGCGGCA----CTCCAGCGGCGG

XM_009389661.2 ---TTCTTTGGAATCCCGTACATGGAAGGCGGAG----GAGAGGCTGCGG

XM_009393219.2 GCGCTCCTCGGGTTGGAGCACGAGGAGT-CATCG----GTGGCTGCGGCG

XM_009415859.2 ACGTTCCTGGGGCTGGAGCACCAGGAGT-CGGCG----GCGGGCGAGGTG

XM_008649541.3 GCGTTCCTGGGGCTGCAAAACGCCGA---CGAGG----GTGGCGGCGTCG

XM_015782909.2 GCGTTCTTGGGCCTCGAGGAGGAGGAGGACGACG----GCGGCGCCGGTG

NM_001349820.1 TGGCCTTCCCCACCTTCCCAGCCTCGCCGCCGCCG------CAGGCCGTG

EU837258.1 TGACG------ACCGCCGCGGCGCCGCCACCGGCC------ATGATGATG

EU847517.1 TCTCC--------TGGCAGGGCATGTCGGCCTCCG------ACGAGTTCG

XM_015775100.2 TCTCG--------TGGCAGGGCATGTCGGCGTCGT------CCGA---CG

CT833260.1 CGATGCCGACGACGAGCCACACCTCGCAGCCAGCC------CCGCCGGCG

AK067060.1 CCACCGCCGCCGCCATCGC--CATCGACGACGGCG------TCAAGCTCG

XM_015795858.2 TGCGCTTCTGGAGCTAACGAACAACAGAGCAGATTC--AGGACAACATAA

EU847519.1 TGCGCTTCTGGAGCTAACGAACAACAGAGCAGATTC--AGGACAACATAA

FR720457.1 -------CGCGCGCATAC-AACTGGAGTGCTGCTC----GGACGACGTGA

EU847520.1 GAGGCGATGAGCTCATAC-TGCTGGAGTGCTGCTC----CGACGACGTGA

NM_001143039.1 AAGGAACTGATCGCGCAGGGACCTCACGACGAGCG-----------GCTG

AK073133.1 CAAGCAGTGTCCTCGCGACGAACAGATGGCGAGCGCC-ACACCTGAGCTC

XM_006380178.2 AGTCGTTTCTT---------------------------------GGGCTG

XM_002315454.3 AGTCGTTTCAT---------------------------------GGCCTG

XM_008392395.2 TGGCGGGTGG----------------------------------------

XM_029095961.1 TGGCGGATGGTGACGGAGGGTATGAGCTGAAGGAGCAAATATCGAGCTTG

XM_002272390.4 GAGCAAATCT--------------------------------CGAGCTTG

NM_001256464.1 CGACGAACTC----------------------------------GACCTG

XM_003546320.5 AGATGAACTC----------------------------------GACCTG

NM_001279196.2 GAACAGATTTCC--------------------------------AATTTG

AY035100.1 TTACGGATTTGAGCCG-GATTATGATCTGAAACAGCAGATATCGAGCTTG

JF968116.1 AAGTGGGGCCGAGTGAGTCAAAGGGGAGTAGCACCGAGTTTGACGGGTCG

JF968119.1 AAGTGGGGACAAGCGAGTCAAAGGGGAGTAGTACCGAGTTTGACGAGTCG

NM_130320.4 TTCTATCAGA--TTCCGGTTGCCGACGACCAATCGGCGACCGATATTG-G

NM_001248198.2 CT-TACTCCG--TTACGGAAGCCAACAACAAAGTAGAGAAGCTTTCCGAG

AY192368.1 TACTTACAAT--CAAG-------AATCAAATAACTGTTACCCCTTTTACT

NM_001247379.2 TACTTACAAT--CAAG-------AATCAAATAACTGTTACCCCTTTTACT

JX145122.1 TGTTTAGTGCGCCAAGCCCTCTGAATACAGTCCCTGTT-CCGTTTCTTTA

XM_002301454.4 GCCTACTCTT--CAAATGGTTTTAATGGCCAGCCAATTGTCGCTTCCGGG

XM_002320960.3 ACCTTCCATT--CAAATGGTTTAATTAACGAACCAATTGTTAGTTCTGGG

NM_001320016.1 TTTAACTCCA--AGGCTAATGTGAACACCGACCCAATTGTTATTTCCGGG

NM_001328756.1 TTTAACTCCA--TGGCCATTGCGAACACCGACCCAATTGTTATTTCCGGG

FQ392750.1 TTCAACACCG--TTGGTGCTATGAACGCTAACCCGGTTGCTGTCACAACC

NM_001254494.3 TCCCATCCCGCGTGGACGCCGTCAACGCTGACTCGGTGGTTGTCTCCGTT

NM_001254517.2 TTCCGTCCCACGTGGACGCCGTCAACGCTGACTCGGTGGTTGTCTCCGGC

AK316980.1 ACAACAACAACAACCAGTTTGGGATGACAAACTCCGTTGATGCTGGATGT

NM_180251.3 ------ACAACAATCAGTTTGGGTTAACAAACTCGTTCGATGCTGGAGGT

JF968115.1 ATGTCGAAAAGGTGGAACCCATAAACTTTACTTCCCCTAATCCTTGCAAC

JF968117.1 ATGTTGGAAAGGTGGAACCCATACTCTTTACTGCCCCCAATCCTTGTAAT

JF968118.1 --------------------------------------------------

NM_001247584.2 TGTCTTTTACTGCTGTAGATATGGGACTGGGCTCAGTTTCCCCTTCAGCT

KJ401124.1 GGAGCAGTGCTTCTGCAGATACGGGATTTGGTTCGTTCGTCCCTTCAGCT

XM_004252354.4 TGTGTGTGACTCCTGTAGATACAGGACCTAACCCATATCCCCATCCAGCT

XM_002266972.5 CCTTCCCTGCCAATGGCGAAGTTGGACTAAAATCACTTGCCCCATCTGAG

XM_024596482.1 CTTTCCCGGCCATTGGAGATTCTGGGGTGACACCCTTGACTCCTTCTGAC

XM_006369031.3 CTTTTCCAGTCAATGGAGATTCTGGGATGAAGACCTTAGCTCCATCTGAC

XM_008341964.3 CCTTCCTTGCCAATGGAGATGTTGCACTGAAATCCTCTACTCCATCCGAT

NM_001294046.1 CCTTCCCTGCCAATGAAGATGTTGCACTGAAATCCTCTGTTCCATCAGAA

NM_001251371.2 CCTTCCCTGGCAGTGGAAAT---GGGCTAAGATCCCTTCCTTCGTCTGAT

XM_006604868.4 CCTTCCCTGGAAGTGGAAAT---GGGCTCACATCCCTTCCGTCATCTGAT

NM_001354284.1 CGTTTGCTGGAAATGGAGTT---CAAGTCTCACCTGTTACTCCATCTGCT

NM_001349033.1 CGTTCCCTGGAAACGGAGTT---CAAGTCTCACCTGTTACTCCATCTGCT

NM_001155962.1 GTGAGGGCGCCG-CCGGTTTCCTCCCCGCAGATCTGTTGC--CCGGCAGT

NM_001157201.2 GTGGCGGTGGTG-CCCGGTAA-TAAGAGGAGGTCGTCCGCGACCAACACT

AK111690.1 CAGAGCCTGTTGATCTCATGCCTCCAGTCAAACCTCTTGC---TTCCACT

XM_008660266.4 CTCAGAACATGCCATTCGCTCCTCTGGTGAA---------------TTAT

XM_015788266.2 TTCAGAATGTCTCATTTGTTCCTGCAATGAACGCGA---------TTGAG

NM_001156774.2 ATGGCAATATGCCATTTGTTCCAGCAATGAACTCTACTGCTTCTGTTGAT

NM_001155219.2 ----CAAT---CCATTTGTTCCAGCAATGAACTCTACTGTTTCTTTTGAG

EU837255.1 CTGATAACATGCCATTTGTTCCTGCAATGAACTCTGCTGCTCCTATTGAG

XM_009403037.2 CCAAGAATATGAACTCCTTCACTGAGATAGAACTGGCTCCACCTTCTGAG

XM_018823871.1 CTATGAGTCAGAACTCC---ACCGAGATAGAACCAGCTCCACCTACCGAC

XM_009419716.2 GTATGAATCCAAATTCCTTCACCGAGATAAAACCAGCTGTGCCTACTGAA

XM_009419259.2 CCATGAACATGGACTCCT---CTGGGATGAATCTATCTCCACCCATCGAG

XM_009415720.2 CCATAAACACATGTTCCTTCTCTGGGATGAAACCATCTCCACCTACTGAT

XM_009411978.2 CGGAGAAACTGAGTTCGTTCCCAGAGATAAAACCAGCTCCACCCACTGTG

XM_009383333.2 CTGAGGAACTGAGTCCATTCCCAGTTATAAAACCCGCTCCACCTACTGTG

XM_009381868.2 ATCACCTGA---ATCCTGTTACCACAATAAAGTCTTCAGCACCTTTAGAA

XM_009405824.2 TTCACATGATGAATCCCCTTATCACAGTAAATTCTTCAGCACCTCCAGAA

XM_009389352.2 ACTACTTTT---ACCCCTTTGCAAGTGAAGGACCTTCTGCACCTTGTGAA

XM_009391309.2 TCTATTTGA---ATACCTTGCCAACAAAAGAACCATCTGCACCTGGTGAA

NM_001084343.2 CTAATGATAGTGAAGAAGCCAACAACGTTGATGCTTCTCTGCTTTCTGAA

NM_001159194.1 ACGGTCGCGTACGTACACCACCAGCCGCCGCCGCAGCAACCGCAGGGCGC

XM_015789203.1 A-GGACGCGGGGCTGGAGCTCTGGA-GCTTTGATAACATCCACACGGCCG

XM_015774497.2 GCGGTGGCGCCGTCGTCGTGCCGGTGTCGGCGGTGGCGCCGGCGATGACG

NM_001153709.2 TCTGGTCCAT-GCCGTGATCCTGCT----ACTTCGGCC-----GTCCATC

NM_001254770.2 TCTGGTCTAT-GCGATGATCCTGCC-TGCACCTTCGCCCATGCATCCATC

NM_001150750.2 GCTGGCTCGC-GCCGGTTTTCGGC-----GCCGCGTCTA----GTTTTTG

CM007650.1_142530856-142531634 AGTGGACGGTCGCCAAGACCATGGC-A--GCAGCAGCGCGAGCAGCGGGC

CM007648.1_220493755-220494609 CAAGTGCCACCACAGCTGCCGCC------TCCGC---------CGCCGCC

NM_001111800.2 CAAGTGCCACCACAGCTGCCGCC------TCCGCGGCCGCCGGCGTCGCC

XM_023301819.1 TAAGTGCCACCACCGCTGCCGCT------GCCGCTGCCGC---CGTCCCC

XM_015775299.2 CCGGCGCCGAGGAAGCCGCGGCG------CCCGCCGGGCTCTGGACCTTC

XM_009384249.2 CATCGTTAGCGCCGGACAAGGAGCCGGA-GGCGGCGCTGGTGGGGATCAC

XM_009385622.2 CGCCGGTGGCAACAGATGAGG-------------TTGTGACAGGGAATAT

XM_009388210.2 CGGCGG-AGGAGGGGACAGCA--------GCAGATGATGA-GGTGGTCAC

XM_009389661.2 --------------------T---------------------------AG

XM_009393219.2 TCGGCGGGGGAGGGAAGTGAGACGAGCAGCGCCGATCTGTGCGGTGA-CC

XM_009415859.2 GCTGCGTGGG---------------------------------GTGA-TG

XM_008649541.3 A-GGCGTGGGACGCCGTGGA---------CCTCATGTTGGCGTAGGC-CG

XM_015782909.2 A-GCCATGGGACGCCGTCGA---------CATGATGCTCGAGTAGGCTCG

NM_001349820.1 GATACGTCCTTCGCCGGCAGCACCGCCACGTCGGAGACCGG---GAGCCC

EU837258.1 ACGAGCTCCTTCACCGACAGCGTCGCCACGTCGGAGTCCGGCGGGAGCCC

EU847517.1 CCGTCGCCGCGGCGGCGCTGTCGCTCGACAGCGACGACGAC----CTCGC

XM_015775100.2 ACGACGCCGCGGCGCAGCAGGCACTCCTCGACGCCGCCGGC----GGCGC

CT833260.1 ACGGTGGCGTCCGAGAACGTCGACGACCCGGAGGTGTTCGA-------CC

AK067060.1 A-GCTGCTCTCGGAGACGGATCCTTCTCCGCCCATGGCCGCCGCCGCCGC

XM_015795858.2 ACGGCA-CGTCTTTGCAACCTTGACATGCGACTCGCCTAATAATCTTGTC

EU847519.1 ACGGCA-CGTCTTTGCAACCTTGACATGCGACTCGCCTAATAATCTTGTC

FR720457.1 TGGACAGCCTCCTCGCCGGCTA------CGACGTGGCCAGCGGC---GAC

EU847520.1 TGGACAGCCTCCTCGCCGGCTT------CGACGTGTCCAGCGA-----AC

NM_001143039.1 GCGGCGAGGATCGTGCCTG--AGCTGA----TGATGCACGGGAGCAGGGA

AK073133.1 ATGGAGGAGGACGCGAGCAGCAGCAGAAACATGGTGCCCCTGTCCATGGC

XM_006380178.2 GAACCTGAC-------CAGATGGCTGCTCGTCTGAGG-------------

XM_002315454.3 GAAACGAAG-------CAGACGGCGGCCCAGCTGAGTTG-TGACGGAGGT

XM_008392395.2 -----------------GGACGAGGTGGTGGTAGAGCAGCTGAGTCAGGT

XM_029095961.1 GAGTCGTTCTTGGGGCTGGATGAGGTGGTGGCGGAGCAGCCGAGTCAGGT

XM_002272390.4 GAATCGCTC------CTGGGTTTGGAGCCGGAACAGGCCGCCACTCAGTT

NM_001256464.1 AAACAAATT---GAATGGTTTCTAGGTTTGGAGAATGAGCTGCCTGTTAG

XM_003546320.5 AAACAACTT---GAACGGTTTCTAGGGTTGGA------------------

NM_001279196.2 GAATCGTTC------CTGGAATTGGAGCCATCTGATCAATT---------

AY035100.1 GAATCGTTCCTTGAGCTGGACGGTAACACGGCGGAGCAACC---------

JF968116.1 GACTCGGTGGATTTGTGGTGTTTGGACCAGTTTCAGTTCTCGAGCATGAT

JF968119.1 GACTCAGTGGAATTGTGGGGTTTGGACCAGTTTCAGTTTTCAAGCATGAT

NM_130320.4 AAATTTATGGAGCTAT----C---------AAGACTCCA-----------

NM_001248198.2 GAGCTTATGGCGTA------C---------GAGAATTTA-----------

AY192368.1 CAATCGAGAACGTT----------------------GAA-----------

NM_001247379.2 CAATCGAGAACGTT----------------------GAA-----------

JX145122.1 CAGTGAAGAAATTTCT----G---------GTTCGCGAA-----------

XM_002301454.4 GAGGATGATTCTGGGT---------------CCGCTTCA-----------

XM_002320960.3 GAGGATGATTCTAGGT---------------CAGGTTCA-----------

NM_001320016.1 GAAGAAAACTCTGGGT---------------GTGGTTCA-----------

NM_001328756.1 GAAGAAAACTCTTGGT---------------CTGGTTCA-----------

FQ392750.1 GAAGTGAAATACGGCT---------------CTGATTCG-----------

NM_001254494.3 GATGAAAATTCGGGGTCAGCG---------TCGGGTTCA-----------

NM_001254517.2 GATGAAAATTCAGGGTTAGCG---------TCGGGTTCA-----------

AK316980.1 AA---TGGGTATCAGTATTTCAG------CTCTGACCAG-----------

NM_180251.3 AACAATGGATACCAGTATTTCAG------TTCCGATCAG-----------

JF968115.1 GC---CTCTCCTCTATACCCGAG------TTCAGGCCAG-----------

JF968117.1 GC---CTCTCCTATATACCTGAG------TTCAGGCCAG-----------

JF968118.1 --------------------------------------------------

NM_001247584.2 GG---TACAAATGTTTACTTCAG------CTCTGATGAA-----------

KJ401124.1 GG---CGGTGATATCTACTTCAA------CTCTGATGTA-----------

XM_004252354.4 GC---TGCTGGTGTTTACTTCAA------TTCTGACCAA-----------

XM_002266972.5 AA---TGGTCGCATGTATTTTAA------TTCAGACCAA-----------

XM_024596482.1 AA---TGCTTCTATGTATTTCAA------TTCTGACAAG-----------

XM_006369031.3 AG---TGCTCCTATGTGTTTCAA------TTCTGACCAG-----------

XM_008341964.3 CC---TGCCCCCTTTTATTTGAG------TTCCGATCAG-----------

NM_001294046.1 AA---TGCCCCTTTTTATTTCAG------TTCCGATCAG-----------

NM_001251371.2 GA---TGCAACCCTTTACTTCAG------TTCAGATCAA-----------

XM_006604868.4 GA---TGTCACCCTTTACTTCAG------TTCAGATCAG-----------

NM_001354284.1 GA---TGTTACTGCTTACTTCAG------CTCTGAGCAT-----------

NM_001349033.1 GA---TGTTACTGCTTACTTCAG------CTCTGAGCAT-----------

NM_001155962.1 AAGAGGTCTGCGGCTAACATGCTGCTGCTCTCCGACCAG-----------

NM_001157201.2 ATGCTCTCCGTCTCCGACGACCAGCGCAGCAACTCCTAC-----------

AK111690.1 GAGACATTCGAGATGAACATGCT------CTCTGATACG-----------

XM_008660266.4 GCTGCCCTAGTGA-ACCTGTC--------TTCAGACCAA-----------

XM_015788266.2 GATACTTTCGTGA-ACCTGTC--------CTCTGATCAA-----------

NM_001156774.2 GATCTTATCATGA-ATCTGCA--------CTCTGACCAG-----------

NM_001155219.2 GATCCTATCATGA-ATCTGCA--------CTCTGACCAG-----------

EU837255.1 GACCCTATCATCA-A--------------CTCTGACCAG-----------

XM_009403037.2 GAACCCAGGACAAGAAATGTGAATACATTCTCTGATCAT-----------

XM_018823871.1 GGACCTGGGATGATGTAT------------TCTGATCAG-----------

XM_009419716.2 GAACCTGGGATCA-ATATGT--------TCTTTGATCAG-----------

XM_009419259.2 GGACCTGCAATTA-ATTTATA--------CTCTGACCAG-----------

XM_009415720.2 GGACCTGCAATTA-ACCTGTA--------CTCTGACCAG-----------

XM_009411978.2 GTGGCTGGGATGA-ATTTGCA--------ATCTGACC-------------

XM_009383333.2 ATAGCAGGGATGC-ATTTATA--------TTCTGACCAG-----------

XM_009381868.2 GAAGCTGCTTTGA-ATTTTCA--------CTCTGATGAG-----------

XM_009405824.2 GGAGCTTCTCTGA-ATTTTTA--------TTCTGACGAG-----------

XM_009389352.2 GG------CCTGG-ATTTCCA--------TTCTGCTGAGGAAGGTGCTGA

XM_009391309.2 GG------TATGG-AGTTTCA--------TTCCGATGAG-----------

NM_001084343.2 GAGCTTCTTGCTTTTGAGAACCAGACCGAATATTTCTCGCAGATGCCTTT

NM_001159194.1 TGCCGGGATGG-ACATG--TGGACG-----------TTCGACGCCATCAA

XM_015789203.1 TGCCGATGTGA-GATCGATCTGATG-----------ATCATTCAGATCGA

XM_015774497.2 TACGGACAGAG-CCAAGAGGTGGCGGC---------TCCGCTCATGTGGA

NM_001153709.2 TCCGGATCTTCCTCTTGTTGTTGTGCG---------TTTATT--------

NM_001254770.2 TCCGTATCTTC-TCTTGTT-TAATGCT---------TTTATTCTATCCTA

NM_001150750.2 AGCTATTGTACTGTGCACGCAAGCGTG---------GTTACTGACAGGAT

CM007650.1_142530856-142531634 GCTAA---------------------------------------------

CM007648.1_220493755-220494609 GAAGAATC----GCAGGTCCCGGCTCC---------GGC--------CGG

NM_001111800.2 GAAGAATC----GCAGGTCCCGGCTCC---------GGC--------CGG

XM_023301819.1 GAAGAAGC----GCAGGTGCCGGCGCC---------AGCG-CCAGC-CGG

XM_015775299.2 GAAGACTA----CGAGCTGCCGTCGCT---------AGCG-CTCTA-GTA

XM_009384249.2 AGGGAGTGCAACGGAAGTGTGTAACCC---------GCCG-GTCTCCTCG

XM_009385622.2 TGGGATTG------AGGCGTGCGATCC---------ACCG-GTCTCTTCA

XM_009388210.2 AGGGAATGGAATGGAGGCGTGTAATCC---------GCCG-GTCTCTTCA

XM_009389661.2 ATGGAATG-----GGGGCATGTAATCC---------GCCG-ACCTCAGCA

XM_009393219.2 TCTGTTTCGTTTGAACGACCCACCGCG---------ATGG-ACGGCCGAG

XM_009415859.2 TCCGTTTCGTTTGAACGATC----GTG---------ATGG-ACGGCTGAG

XM_008649541.3 TACGCCTTAGCAGGGGGGCCCAGCGCC---------AGCG-CCAGCCCCA

XM_015782909.2 CCGGGAATGGCAGCGTTGCCATGCTCA---------TGCATCCAAGCTTA

NM_001349820.1 CGCGAAGAGGCCGAGATGCGACGAAG------------ACTCGTCCGAGG

EU837258.1 CGCCAAGAAGGCGAGGTCCGACGACGT---------CGACTCGTCCGAGG

EU847517.1 CAAGAAGCGTCCGAGGACCGAGCCGGA---------GGACACCACCGAC-

XM_015775100.2 CAAGAAGCGTCCCCGGAGCGAGCCCCA---------CGTCACCTCCGACG

CT833260.1 CGTACGACGTCCACGG--CGGCCTCGC---------CTCCTACTTCGCCG

AK067060.1 CGCGTGGCTCGACGCGTTCGAGCTGA------------ACGATCTTGACG

XM_015795858.2 AAACAAGGGCCGCCAGCTTAATTAGC-------------TTTTTCCAAAG

EU847519.1 AAACAAGGGCCGCCAGCTTAATTAGC-------------TTTTTCCAAAG

FR720457.1 GACATATGGACATGGACATCTGGAGC-------------CTCCTCCACCT

EU847520.1 CACGCAGTGTTTTGGGAATGGTTAAT-------------TAGCAGCGCGC

NM_001143039.1 --CGAGGCTGC-------GGCGTTGAT---------CGCCGAGTTCAGCC

AK073133.1 GCTGCAGCTGCAGTATGCGGCGATGAT---------CGCCGAATGCGACC

XM_006380178.2 GA-AAACGGCGGTGATAATCGTGACTC---------TGGGGA---CCTTT

XM_002315454.3 GG-AAGTGGTGATTATAATTGTGACTC---------TGTGGA---CCCTT

XM_008392395.2 GGTGAGTGGAAGCGGAGAGTCGGACTC---------GTTGTA---CCTGT

XM_029095961.1 GGTGAGTGGAAGCGGTGACTCGGACTC---------GTTGGA---CCCGT

XM_002272390.4 G---AACGAAATCACCGAGTCAGACTC---------GGTCGA---CCTCT

NM_001256464.1 CAACAACATTGGTGCTGAGTGGGACAA---------CATGGATGACCTGT

XM_003546320.5 ---CAACATGGGTGCTGAGTGGGACAA---------CATGGATGACCTGT

NM_001279196.2 ---TTCAGGGATCGTCGATTCTGATCC---------TCTTAA---TGTTT

AY035100.1 ---GAGTCAGCTTGATGAGTCCGTTTC---------CGAGGTGGATATGT

JF968116.1 TGGCGGTGGTGGTGG---------------------TGGTGGC--AGCGG

JF968119.1 GGTTGGTGGTGGCGGGGACTGTAGCGG---------TGGTGGT--CCTGG

NM_130320.4 --ATTAAATCTC-TTATTTCC-----------------CGGCCGGTTTGC

NM_001248198.2 --ATGAGGTTTTATCAGATTC-----------------CGGAAAACGTCG

AY192368.1 --CCTGTTAT-----------------------------GGAATTTGCAA

NM_001247379.2 --CCTGTTAT-----------------------------GGAATTTGCAA

JX145122.1 --CCGATTGCTCTTCGACCGA-----------------GGGAAATCATCA

XM_002301454.4 --GAGGAGGG---------AA-----------------CAAGACTGGTGG

XM_002320960.3 --GAGGAGGT---------GA-----------------CAGGACTGCTGG

NM_001320016.1 --GATGGCGCTTTCTCCTCAA-----------------CGGGCTTTCTGG

NM_001328756.1 --GATGGCGCTTTCTCCTCGA-----------------AGGGGTTGCTGG

FQ392750.1 --GAGTCTGTGTACCCTTCCT-----------------CTGGATTGTTGA

NM_001254494.3 --GAGGGTGCTTATTCGACAA-----------------CGGAGTTCATGG

NM_001254517.2 --GAGGGTGCTTATTCGACAA-----------------CGGAGTTTATGG

AK316980.1 -GGTAGTAATTCTTTCGATTG---------------TTCGGAGTTTGGTT

NM_180251.3 -GGCAGTAACTCCTTCGACTG---------------TTCTGAGTTCGGGT

JF968115.1 -TCCAGTAACTCGTTCGATTA---------------TGCTGACTTTGTGT

JF968117.1 -TCTAGTAACTCCTTTGACTA---------------TGCCGACCTTGGAT

JF968118.1 --------------------------------------------------

NM_001247584.2 -GCAAGTAACACTTTTGACTG---------------CTCTGATTTCGGTT

KJ401124.1 -GGAAGCAACTCTTTTGAATG---------------CTCTGATTTTGGTT

XM_004252354.4 -GGAAGTAACTCTTTTGGCCC---------------CTCAGACTT---TT

XM_002266972.5 -GGAAGTAACTCTTTTGATTG---------------TTCTGACCTTGGAT

XM_024596482.1 -GGGAGCAACTCATTTGATTG---------------T---GACTTTGGGT

XM_006369031.3 -GGGAGCAACTCATTTGATTG---------------T---GACTTTGGGT

XM_008341964.3 -GGAAGCAACTCATTTGATTG---------------TTCTGACTTCGGCT

NM_001294046.1 -GGAAGCAACTCCTTTGATTG---------------TTCTGACTTTGGCT

NM_001251371.2 -GGGAGTAATTCATTTGATT------------------------------

XM_006604868.4 -GGGAGTAATTCATTTGGGTG-----------------------------

NM_001354284.1 -TCGAGCAGCTCGTTTGATTA---------------TTCTGACCTCGGAT

NM_001349033.1 -TCGAGCAACTCGTTTGATTA---------------TTCTGACCTTGGAT

NM_001155962.1 -AGCAGCGACTCGTATGGCTC--TTGTGACTTGGG-GTGGGAGTGGGACT

NM_001157201.2 -GGCTCCGGCTCCTCCGACTTGGTGGGGTCGTGGTCGTGGGACGACGACG

AK111690.1 -AGCAGCAACTCATTTGGCTC---------------TTCAGACTTTGGTT

XM_008660266.4 -GGCAGCAACTCGTTCGGTTG---------------CTCGGACTTCAGCC

XM_015788266.2 -GGGAGCAACTCCTTTGGTTG---------------CTCGGACTTTAGCC

NM_001156774.2 -GGAAGTAACTCCTTTGGCTG---------------CTCAGACTTGGGCT

NM_001155219.2 -GGAAGTAACTCCCTTGGCTG---------------CTCAGACTTGGGCT

EU837255.1 -GGAAGCAACTCATTTGGCTG---------------CTCTGACTTTGGCT

XM_009403037.2 -GGAAGCCCCACTTTTGGTTA---------------TTCTGAATATGCTT

XM_018823871.1 -GGAAGCAATTCCTTTGGCTA---------------TTCTGAATTTGGTT

XM_009419716.2 -GGAAGTAACATTTTAGGTTA---------------TTCTGAACATGGTT

XM_009419259.2 -GGAAGCAACACTTTTGATTG---------------TTCTGGATATGGCT

XM_009415720.2 -GGAAGCAACTCTTTTGATTG---------------TTCTGAATATGGCA

XM_009411978.2 --------------------------------------------------

XM_009383333.2 -GGAAGTAACTTATTTGGTTT---------------CCCAGAAAATGAAT

XM_009381868.2 -GGAAGTAATTCTCTTGGTTA---------------TGCTGACTTTGTAT

XM_009405824.2 -GGAAGTAATTCTTTTGGTTA---------------TACCGACTTTGTAT

XM_009389352.2 GGAAAGTGACTCCTTTGGTCA---------------TCCAGAATTCATAT

XM_009391309.2 -GGAAGTAACTCTTGTGGTGA---------------TCCAGACTTTGGAT

NM_001084343.2 TACGGAGGGAAACTGTGATTCCTCAACGTC------TCTGAGTAGTCTCT

NM_001159194.1 ----CAC--GC-CCGTGCCCATGT-GATGTGA-TCGAC--AGGGCAACGG

XM_015789203.1 ----TGCTAGC-TCATGTGTTTTT-AATTATA-TCTTC--ACAGCAGAAA

XM_015774497.2 ATTTCGATGACATCACGGCCATGCCAATGTGA-TTGTCTTACCGTGGAGA

NM_001153709.2 ------TTTA--TGATGACCAGATTCTAC-GG-TTGTTTTTTAGGGGA--

NM_001254770.2 CTCTATTTCA--TGATTATTAGAGTCCATTGA-TTGCCTTTTAGGGAA--

NM_001150750.2 CACAGCTAGAC-TGACCAGCATATCTAGTTGG-TTCTTTTGTAGCGTATT

CM007650.1_142530856-142531634 --------------------------------------------------

CM007648.1_220493755-220494609 GCTATGGA---GCTTCGAAGATT--ACTACTA-CCCATCG---TCTCAGT

NM_001111800.2 GCTGTGGA---GCTTCGAAGATT--ACTACTA-CCCGCCG---TCTCTGT

XM_023301819.1 GCTGTGGA---GCTTCGAGGACT--ACTACTA-CCCGCCG---TCACTGT

XM_015775299.2 AAAATGTC---ACAATAAAAATG--ACATGTA-TGTGAAAAAATTTTTCC

XM_009384249.2 GGCATGGAGATGCTTTGGAGCTTTGAGGACAT-TCTGCCAACAT--CATA

XM_009385622.2 GGAATGGAAATGCTTTGGAGCTTCGACGACAT-CCTTCCAA-AC--TAAT

XM_009388210.2 GGCATGGAGATGCTTTGGATCTTCGACGACAT-TCTGCCGGCGTGACGGC

XM_009389661.2 GACATGGAGATGCTGTGGAGCTTTGACGACAT-CCTTCCCACCT--CGGG

XM_009393219.2 ATGATG--GGTGGCCATTTTGTCGTTCTCCTT-TCT-CTCTCATCAAAAC

XM_009415859.2 ATGTCGATGGTGGCAATTCTGTAGTTTCTTTT-TTT-TTCTCCTT----C

XM_008649541.3 GCCCCAG-CGCGCGCGTGCCGCGTGCCGTCGC-TGT-GAACGGCCGC--C

XM_015782909.2 TTCATGCATGCAGCAGCAAGATATACAACTTT-AGTACTACTACTATGTT

NM_001349820.1 GC---------GGCAGCGGCTCCGCGCTGGAG-CT-CGCTGACGAGCTGG

EU837258.1 GCAGCGTCGGCGGCGGCAGCGACACGCTGGGT-TT-CACCGACGAGCTGG

EU847517.1 --------------TCCGGC----TCCGGCGA-CGACACCGACGCGCTGT

XM_015775100.2 ACGAAGT----GCTCCCGGCGTCATTCGACAG-TGACAACAACACCGCCG

CT833260.1 GCGGC------GCGTACGAGTCCCTGGAGAGC-CTGTTCGCGCACGGTGG

AK067060.1 GATCAAGATGCAAGGACAACG-CATTCGATCA-CCAGATTCACAAGGTAG

XM_015795858.2 GTGGGGAACAATTTTGGGGGA-TGCAATTAGG-ACGCACAGAGATGACCC

EU847519.1 GTGGGGAACAATTTTGGGGGA-TGCAATTAGG-ACGCACAGAGATGACCC

FR720457.1 CTGTTAACCAAGAGATCAAGA-CCCCATCGAT-CCAC-CAAAACATATCA

EU847520.1 ACGCCTG--ATTAGATCGGTA-CATGTGAAGT-ACAA-GAGAAGTAGTTA

NM_001143039.1 GTCAGATGGAGGAGATCGCTGCGTTGCGGAGG-GACCTTGAGACACGCGA

AK073133.1 GCGAGATGGAGGAGATCGCCGCCGTGGAGAGG-GACCTCGAGAGGCGCAG

XM_006380178.2 GGATGCTTGATGATCTTGTG---ACACATC-------ATCAGTATCGACG

XM_002315454.3 GGATGCTTGATGATCT--------------------------TATCAACA

XM_008392395.2 GGATGCTGGATGACCTGGTG---GCATATC-------------AGCAACA

XM_029095961.1 GGATGCTGGATAACCTGGTG---ACGCACCTG-CAACAGCAACAGCAACG

XM_002272390.4 GGATGCTGGAGGACCTTGCTTCCACTCACCCT-CACCACCATCGTCAGCA

NM_001256464.1 GGATGCTGGACGACGTCGTTGTGCCCAACCGT-CACTTAATTTACTAG--

XM_003546320.5 GGATGCTGGACGACGTCGTTGTGCCCAACCGT-CACTTAATTTACTAGAA

NM_001279196.2 TTCTGATGGAGGACTTTGCTTCAACTCATCAT-CAGTTCTACTG--AACA

AY035100.1 GGATGCTTGATGATGTCATT-----GCGTCG-----------TATGAGTA

JF968116.1 CAGCGGCGGGGGGT------TCATGTAAGCCCGGGGGACACGTGGCGGAA

JF968119.1 CGGCGGCGGAGGATACGGTTTCATGTAACCC--GGCGCCACCTGGCAGAA

NM_130320.4 TCACTCATTAATATGCTGCTA--------------AT-TTACTTGTTTTT

NM_001248198.2 TTGGGGATTTGTGGGCCTTTCCTGAC---------AC-CTCTCCACCTCT

AY192368.1 GTTATAATGGAATTGAAGAT---------------------GGAGGAGAG

NM_001247379.2 GTTATAATGGAATTGAAGAT---------------------GGAGGAGAG

JX145122.1 GATG-AATCAATTCCAGGGC---------------------GATGTGAAT

XM_002301454.4 GCTGTAATCAGAACGTGAAGAGTAATATTTATATGGG-TCAAGTGAAGCT

XM_002320960.3 GCTGTAATCAGAATGTGGAGAGCAATAATTATATGGG-TCAAGTAAAGGT

NM_001320016.1 GTTGCAATCAGAACGGGAGCGGCGGTTGTCA---CGG-CGGAGCTGAGCT

NM_001328756.1 GTTGCAATCAGAACGGGATGGGCGGTTGTCA---AGG-CGGATCTGAGCT

FQ392750.1 ATTGCAATCAGAA---------------------------AGCCTGTGTT

NM_001254494.3 GGTCCGTTCAGAACGGGAACGGTTAT------------TTGGGTGGCACG

NM_001254517.2 GATCCAGTCAAAACGGGAACGGGTAT------------CTGGGTGTTACC

AK316980.1 GGAGCGATCAAGCTCCGATAACTCCCGACATCTCTTC---TGCGGTTATC

NM_180251.3 GGAGTGATCACGGCCCTAAAACACCCGAGATCTCTTC---AATGCTTGTC

JF968115.1 TGGGAGCAAATGGCCCCCAAACCCCTGAGATATCATC---TTATTTCTCA

JF968117.1 TTGGAGAGAATGGACCCCAAACCCCTGAGATATCTTC---TTATTTCTCA

JF968118.1 --------------------------------------------------

NM_001247584.2 GGGCTGAACCGTGTGCAAGGACTCCAGAGATCTCATC---TGTTCTGTCG

KJ401124.1 GGGGAGAGCCATGCTCCAGGACTCCAGAGATATCATC---TGTTCTGTCA

XM_004252354.4 GGGGAGAAACATGTTCAAGGACTCCAGACATATCATC---TGTTCTGTCA

XM_002266972.5 GGGGAGAGCAGGGAGCAAAGACCCCAGAAATCTCATC---GGTTCTTTCA

XM_024596482.1 GGGGAGAACAAGGCCC---------TGAAATCTTGTC---TGTTCTTGCA

XM_006369031.3 GGGGAGAACAAACCCCAATGACTCCTGAAAGCTTGAC---TGTTCTTGCA

XM_008341964.3 GGGGAGAACAAGGCTCAAAGACTCCAGAAATCTCATC---AGTTCTTTCT

NM_001294046.1 GGGGAGAACAAGGCTCGAAGACTCCAGAAATCTCATC---CGTTATTTCA

NM_001251371.2 ------------------ATGCTCCTGAGATCTCATC---CATGCTTTCT

XM_006604868.4 -GAGTGAACAGGGCCCCAAAACTCCTGAGATCTCATC---CATGCTTTCT

NM_001354284.1 GGGGTGAACAAGTCCCCAAGACACCCGAGATCTCATC---CATGCTTTCT

NM_001349033.1 GGGGTGAACAAGTCCCCAAGACCCCCGAGATCTCATC---CTTGCTTTCT

NM_001155962.1 GG---GACGATGACACCATGACCTCAGACTACGCCTC---AGTCTTTGCT

NM_001157201.2 CG---GCGGCCGCCGCCATGACCTCGGACTACACCTCCTCGGTCTTCGCT

AK111690.1 GG---GAGGATGACACCCTGACCCCAGACTACACTTC---AGTCTTCGTT

XM_008660266.4 TC---GAGAACGACTCCAGGACCCCTGACATAACTTC---GGTGCCTGCG

XM_015788266.2 AG---GAGAATGATATCAAGACCCCTGACATAACTTC---CATGCTTGCA

NM_001156774.2 GG---GAGAATGATACCAAGACACCAGACATCACTTC---CAT---TGCT

NM_001155219.2 GG---GAGAATGATACTAAGACACCAGACATCACATC---CAT---TGCT

EU837255.1 GG---GAGAATGATACCAAGACACCAGATATTACATC---AAT---TGCT

XM_009403037.2 GG---GAACAGGAGGTTAAATATCCATATATCACAAC---AATTGTTGAT

XM_018823871.1 GG---GAACATGAGGTTAAAACTCCAGAGATCACAAC---AAGTCTTGAT

XM_009419716.2 GG---GAACATGAGTTTAAAACTGCAGCCATCACAAC---AATTCTTGAG

XM_009419259.2 GG---GAAAGTGAGGTTAAAACCCCCAATATCACTTC---ATTTCTTGCT

XM_009415720.2 GG---GAAGGCGAGGCTAAAACTCCTGAGATCACATC---GATTCTTACT

XM_009411978.2 ------TACATGAGATTAAGACTCCAGAAATCACATC---AGTTATTTCT

XM_009383333.2 CT---CTAAATGAGGCGAAAACTCCAGATATCTCATC---AGTTCTTGCT

XM_009381868.2 GG---GAATTTGATGCCAAGACCCCAGAGATCATGTC---AATCCTTGCT

XM_009405824.2 GG---GAGCTCGAGGCCAGGACCCCGGAGATCACATT---GATTCTTGCT

XM_009389352.2 GG---GAACATGAGGACAAAACCCCTGAGATCAAGTT---AGTTGATGTT

XM_009391309.2 TG---TTA---------------------------TC---AGTTCAAGTT

NM_001084343.2 TTGATGGAGGCAATGACATGGGTCTATGGTCCTGAAC-GGTTCTATTGTA

NM_001159194.1 --GCGGATATGCTAGCTGCTGTGCGGCCTGCT-TTGTGCTTCTTA---TG

XM_015789203.1 C-AAAAAAAAGTTCAATTCAGTTTATTTTGTTGTTATACGTTTTAAAATG

XM_015774497.2 T-TAAGGCATTTAAAGGCTTATAGATAACATAAATTTGCCATGTATGATC

NM_001153709.2 -----AGAGAATCATA--AATTGAATTTTGGAGCCATGTGGCCACCAGCT

NM_001254770.2 -----GGGATTCCATACGATCTCCATCTGAAAAGAGAACACTTATTAGCT

NM_001150750.2 TTTTTAGGGAAACATGATTCTTTTTTTTTGTTGTCGTGTATTGATTGGGA

CM007650.1_142530856-142531634 --------------------------------------------------

CM007648.1_220493755-220494609 CGCTCTTTACTGAATGA---------------------------------

NM_001111800.2 CGCTCTTTACTGAATGATGAAC--GTGCTTTGA--CCGTGCCTGTTTTGG

XM_023301819.1 CGCTCTTCACTGAATAATGAAC--GCGGTTTG---CCGTGCCTGTGTTGT

XM_015775299.2 CTACCCGTAGTAAGTAACCAGT--GTCTTTTTTTACCGTACTCTATGCGT

XM_009384249.2 TGGTCCGTAATATATGATTGCT--GTCATCTCTTCAAGCGCTGTGTTGTA

XM_009385622.2 TGCTTTCCTCTCTTCAATGGTT--GTGTTGGACATCGACGCTTGTGCATA

XM_009388210.2 CCCATTCGTCTCCTTGACCGCATGGTGTTTGATACTTATGGCTTCCCG-A

XM_009389661.2 TGATCTT---TAAATGATCTCTATCATCTCTACTTCGACGGCATTCTTCC

XM_009393219.2 GAACCCATCAAGAGACTAGTGGTTATGTTATAAGGTGCTGCGGTAAATAT

XM_009415859.2 TTGTCCTACAAAAG-CAAAAAAGGAAAAAAAAAATCCGTATTTTATGGTT

XM_008649541.3 GCAGCTGTTTTGGATCGAGTAATAATATTATTAG-TAGTATAATGTTTCT

XM_015782909.2 AATTACTACTTACTGCGTAGGTAAATGAAATAAAATGGGGTTGGTTAATT

NM_001349820.1 CGTTCGACCCGTTTGTGCTGCT-GCAGATGCCCTACTCGGGTGGGTACGA

EU837258.1 AGTTCGACCCGTTCATGCTGTT-CCAGCTCCCCTACTCCG------ACGG

EU847517.1 TCGACGCGCTGCTGTTCGCCGA-CCAGTACAACCACTTCAACGGCGGCGC

XM_015775100.2 CCGCCGGCCTGCTCCCGCTCGA-CGATCCTTTCTTGTTCGGCGACCAGTT

CT833260.1 CGACAGCGCCGCCGTCGACCAA-GCGGCGAGCGACCACTGGCCGGCGGCG

AK067060.1 AAGCGG--CTGTCGCTGATG-----AATTCGCGTTCTACGACGATCCGAG

XM_015795858.2 CAACTTGTCAATGGGAATTGAA-CTCTGATGACCTTTGTAATGATAATCA

EU847519.1 CAACTTGTCAATGGGAATTGAA-CTCTGATGACCTTTGTAATGATAATCA

FR720457.1 TATGCAGGTGCCCGCCCCATGA-CTT-----GTCACTTTAAGAATCATAA

EU847520.1 CTACTAGCTAGGAGCAAATTAA-CTT-----GG-AGTGCAAGAATAAAAT

NM_001143039.1 GAGGCAGCTTGTTCAGCTGGTTTCTCTAGTACTTCGTTGACCACTCACTC

AK073133.1 GAGGCAGGT-GTTCGAGCG-------CAGAGGCCACCTGGTCAGGCAGGC

XM_006380178.2 TCAGATTAATTATTAGTAAAATA--ATTA--ACTACTAAT---GCTACTG

XM_002315454.3 TCAGCTTAATTATTAGTAAAATATAATTA--ACTCCTAATCTGGCTAGTG

XM_008392395.2 AGG--GCAGC-TTCTGTATTAAGGCAGC-------GGAAT--TGTTCTAG

XM_029095961.1 GGG--GCAGT-TTCTGTATTAAGGCAGC-------GGAAT--TGTTCTAG

XM_002272390.4 TGTATGCAGCGCTGCGTTTTGATGCACCA--CTCTGTAAT--GGTTCCAG

NM_001256464.1 --------------------------------------------------

XM_003546320.5 GGGAGATAAT--TAATTAATTAATAAATG--GCGTTTTCTTAAGTTATAG

NM_001279196.2 TGATCAAAGTGTTATTTCGGGAGTATTTT--ATGGCTGAGTAGTTTATTA

AY035100.1 AAAGAAAAAAAATAAGTTTAAAAAAAGT-------TAAATAAAGTCTGTA

JF968116.1 TAAGAGGACTGCTGCTCAGAAGCTATATATTTTTGTTGGTGAAACTATAA

JF968119.1 TAAGAGGACCGCT---CAAAATCTAC-----TTTGTTGGTGAAATTATGA

NM_130320.4 TACTTAAC----------------------------AATCAAGTCTAATT

NM_001248198.2 CTGATTTC----------------------------TGTCACATCGTTTC

AY192368.1 GAGATGGT---------------------------GAAAAATTTGAATAA

NM_001247379.2 GAGATGGT---------------------------GAAAAATTTGAATAA

JX145122.1 GTGAAGGT---------------------------GAAGGAGGAGGAAGA

XM_002301454.4 GGAAGTGGAGGA-----------------------GAAGCTAGAGAAGGT

XM_002320960.3 GGAAGAGGAGAAAC--------------------TGGAGGAGAAGAAAGT

NM_001320016.1 GACAGAAGTGGAG----------------------GAAACTAAAGAAGGG

NM_001328756.1 GACAGAAGTGGAG----------------------GAAACACAAGAAGGG

FQ392750.1 GGGAAAGT---------------------------GAAAAATCAGAAAGA

NM_001254494.3 GTAATGGA---------------------------GAAGAAGGAGAAAGA

NM_001254517.2 GCAATGGA---------------------------GAAGAATGAGAAAGA

AK316980.1 ---------AACA------ACAACAACTCAGCTCTGTTCTTTGAGGAAGC

NM_180251.3 ---------AATA------ACAACGA---AGCATCATTTGTTGAAGAAAC

JF968115.1 ---GCTATTCTGG------AGGGCGAAGACG------------AGCAGTG

JF968117.1 ---GCTATTCTGG------AGGGCGAAGAAGCTCAAATTACAGAGCAGAG

JF968118.1 --------------------------------------------------

NM_001247584.2 ---GAAGTTCTGG------AAACCAATGAGACTCATTTTGATGATGATTC

KJ401124.1 ---GCTGCTATTG------AATGTAATGAAGCTCAATTTGTTGAAGATGC

XM_004252354.4 ---GCTGCTATAG------AATGCGATGAAGCTCAATTTATTGAAGGTGT

XM_002266972.5 ---GCTACCCTTG------AAGGTGATGAATCCCAATTTATTGAAGATGC

XM_024596482.1 ---GCAACTCCAG------AAGTTGATGA---ATCCGTCTTTGTGGATGC

XM_006369031.3 ---GCTACACCAG------AAGCTGATGA---ATATCTCCTTG------C

XM_008341964.3 TCTGTGATGGAAG------AAACTGATGACTCACTGTTTCTCGAGGATGC

NM_001294046.1 TCTGTTATGGAAG------AAAGTGATAACTCACCGTTTCTGGAGGATTC

NM_001251371.2 ---GCT---CCTT------TGGATTGTGAATCTCATTTTGTGCAAAATGC

XM_006604868.4 ---GCT---CCTT------TGGATTGTGAATCTCATTTTGTGCAAAATGC

NM_001354284.1 ---GCTGCTCCTT------TGGACGGTGAATCTCAGTTTGTGCAGGGTGC

NM_001349033.1 ---GCTGCTCCTT------TGGA---------------------GGGTGC

NM_001155962.1 CCAGCTGCTCCCA--GTAACGTTGTGCCAGCATG-GTACACGCAAGGTGG

NM_001157201.2 CCCGATAACGCC---GTGCTGCCGG--CGGCGTC-TTACACGCAAGGCGG

AK111690.1 CCTAATGCTGCCATGCCAGCATATGGTGAACCTGCTTACCTGACAGGTGG

XM_008660266.4 CCCGTTGCCACC----------------TTGGCCGCCGTTGGCGAGTCTG

XM_015788266.2 CCG------ACC----------------ATGACAGGTGTTGATGACTCCG

NM_001156774.2 CCCATTTCCACT----------------ATTGCGGAAGGCGACGAGTCTG

NM_001155219.2 CCCATTCCCACT----------------ATTGCTGAAGGCGATGAGTCTG

EU837255.1 CCCATTTCAACC----------------ATAGCTGAAGTCGATGAATCTG

XM_009403037.2 CCAGCGAACCCTG------AGGCTGAAAATTTTGTATTGCTCGAAGATGG

XM_018823871.1 CCAACCATAACTG------AAGTTCAAAATTTTACATGTCTGGAAAATGG

XM_009419716.2 CCAACCATAACTG------AATCTGAAAATTTCGCATGCCTAGAAGATGG

XM_009419259.2 CCAACCATGAATG------AAGCTGAAGGAACACCATACCTGGAACAGGG

XM_009415720.2 CCAACCATGACTG------AATCTGAAGAATCAGCATACTTTGAA---GG

XM_009411978.2 CCAACAATTTCTG------AAGTTGAAGAGACAGCCTGCCTAGAAAAAGG

XM_009383333.2 GCAACAATTTTTG------AAGTTGAAAATGCAGCATACTTGGAAAATGG

XM_009381868.2 CCTATCG---------------------AACCTGAGTTATTTGAAGATGG

XM_009405824.2 CCCACTG---------------------AACCTGATATATACGAAGATGG

XM_009389352.2 CCCACTGCTATTG------AAGGAAATGGTACTGGGTTGCTGGGAGATGA

XM_009391309.2 CCCACTGCT------------------GACAATGGATTGTTG--------

NM_001084343.2 CAAATGACATGAGTGTTTTAAATGAGTTCTTCTTCGACTTGTCTGTTTGC

NM_001159194.1 AATGAACATGTTGCTACTTTCCAATATTTATTTAT---ATTCG--TTGCA

XM_015789203.1 TTTCATTAGGTTTTCATGTTATAGGAGTGATTTATCGGATTGAACTCTCA

XM_015774497.2 AATGGTTAATTCCTATTGCTCAAGGGCTTATATTAAGGGTTACCTACACG

NM_001153709.2 C------ACTGGCTGTGCTGTAAATCT--GTAACTCG---TCAGTCAGCC

NM_001254770.2 TCACAAGATTGCTTGGCATGCAGACGTACGTAAAGTGAGTTTAGGAAGCA

NM_001150750.2 TATAGAGAGTTTTTTTCCTCTCTTGAT--GGAAGTAG---TTGGAAAGCT

CM007650.1_142530856-142531634 --------------------------------------------------

CM007648.1_220493755-220494609 --------------------------------------------------

NM_001111800.2 TAACACCTTTGTGAACA-GATTTCCTGCTCGTCGTAGTCGGTGTGATGTG

XM_023301819.1 TATCTCTTTTGTATCTGTGGCTCGCGAGTTGAGGCATGTAAACAAGCTTT

XM_015775299.2 TATTTTTGTTGAAATTAATCCATTGTTGAAGTTGTAACTGTGCATGGTCG

XM_009384249.2 CATTTCA-TCATCTCTGAGGTTTGT--CGTGGTTC-TTCCG-AGTCGGTA

XM_009385622.2 CGCAACT-TC-TTCGTATGTTGTTT--AGTAGCTT-CATTA-AGAGTGTA

XM_009388210.2 TGCTTGT-ACAATGGCAAATTTTGTTTCGTATTTTGTTTTC-AAGGGTTG

XM_009389661.2 CACCTCGGTCAATGGTTTGTATTGGATAGCGATGGTTTCAGTAACCGTCG

XM_009393219.2 CAGTATCCCACATGTATCATCTAATCACTGCTTTGATTATCGTGAGATCA

XM_009415859.2 AAGATTAACGAACGGTAAATATGATTAGTGGTTGGATGGTC---------

XM_008649541.3 CCAAGCAGCTAA-GATAATTGCAAGCCTTGCT-GGGTGGTATTACTAGCA

XM_015782909.2 AGGGTGTTCTTCTGGGAATCCTTGGCTTTGCT-AATTGCTAGTACTACTA

NM_001349820.1 CGACGA--CTCACTGGACGGCCTTTT-CGCCGCAGATGAGGCCGTGCAG-

EU837258.1 CTACGA--GTCCATCGACAGCCTCTT-CGCCGCCGGCGACGCC----AA-

EU847517.1 CTACGA--GTCCCTGGACAGCCTGTT-CAGCGC---CGACGCCGTGCAG-

XM_015775100.2 CGGCGA--CCTCAACGGCGGCGCGTT-CGCCTCGCTCATGGACGGGCTG-

CT833260.1 CTATGGAGCTTCGCAGACGACGGCTCGTTCTGCTTCTGATGCTGTCAAAT

AK067060.1 CTACAT--GCAGCTGGGTTACCAGCT-CGATCAGGGCAAC-TCGTACGAG

XM_015795858.2 AGGCACCACATTGTGGGGTTAA-GCA-AAGTTTTGGCAAATTAGCTTT--

EU847519.1 AGGCACCACATTGTGGGGTTAA-GCA-AAGTTTTGGCAAATTAGCTTT--

FR720457.1 AAACACTTTTGTACAAATGGAGTGCC-CAACCATGCTAAACTTACTCAA-

EU847520.1 ATGCATGTCTCTGCACTACTACTGT---AGTGTTAGCAAGTTTGCTCA--

NM_001143039.1 CGATGATGCATGATCTGATGACTGTC-AAGTGTGAACAATGTGTTTGTGA

AK073133.1 C--TCTCTTCTGCTCGACTGACGATC-TTGC-TGAAC--TGAACTCTGAA

XM_006380178.2 AT--GCATGCATCTCTTCTGCT-TAATGTACTGCTTTTGGCTTTTACAAA

XM_002315454.3 AT--GCATGCATGTTCACTGCT-TAATGTATTAAGACCCTGTTTTATTAA

XM_008392395.2 CT--AAATATTTGTATGGCTAG-GAATTAAAAACATATTAATTAAAGGGT

XM_029095961.1 CT--GTATGTTGGTATAGCTAG-AAA--AAATAAATATTAATTAAATTAT

XM_002272390.4 TG--CATCGCAGATACAGTAAT-AAAT-GCATGTG-GTTGCATGCATCGC

NM_001256464.1 --------------------------------------------------

XM_003546320.5 TT--TTATAAAACTATGTTGGT-GTATGTGTTCTTAGTTTTCTGTTTTGT

NM_001279196.2 ATTAGCTTTTGGGTGTAGTAGT-AGTAATAGTAGTAATAGAGATTAGAGA

AY035100.1 AT--ATATATGTAACCGCCGTT-ACTTTTAAAAGGTTTTTACCGTCGCAT

JF968116.1 TATTTTGGGGGCGTCTACTACTCTACTCTACTGCACTAATTGATGGTTAG

JF968119.1 TATTTTGAGTCAGTCAA--ACT-TAATTAATTCCAGT------TTGTAAG

NM_130320.4 TGTTTCCATCAATATTTCAGATAAGAG-------TAAAGC----------

NM_001248198.2 TTCTATCATTAATTCTCCAATTCTGTTCT-----TAAAATATGATAT---

AY192368.1 --------CAGGGTTGTAGAGGAAG-----------AGGAGAAAA-----

NM_001247379.2 --------CAGGGTTGTAGAGGAAG-----------AGGAGAAAA-----

JX145122.1 GAA----GCAAGAGCTGAGAAAAAGCAGC-----AAAGGAGGAGAG----

XM_002301454.4 GAAGAATAAGGAGGTGGTGGTGGTGGATT-----TGGGAACTGGAGA---

XM_002320960.3 GATGAACAAGGAGGTGATGGTGGTGGATT-----TGGAAACTGGGGA---

NM_001320016.1 AA-----ACGGAGCT--TGGAGGCGATTG-----CAAGAAT---------

NM_001328756.1 GA-----ATTGAGCT--TGGAGGCGATTG-----CAAGAAT---------

FQ392750.1 GAACCAAACAGAGACTGTGACGGAAGCT-------GAGAA----------

NM_001254494.3 GA------CAGAGGTTATTGAAGCTG---------AAGAA----------

NM_001254517.2 GA------CAGAGGTTATTGAAGCTC---------AAGAA----------

AK316980.1 CAATCCAGCTAAGAAGCTCAA------------------GTCTATG---G

NM_180251.3 CAATGCAGCCAAGAAGCTCAAACCAAACT---CTGATGAGTCAGAC---G

JF968115.1 TAATGCC---AAGAAGATGA---------------AGGTGTCAACC---T

JF968117.1 TAATGCC---AAGAAGATGA---------------AGATGTCAACC---T

JF968118.1 --------------------------------------------------

NM_001247584.2 CAGACCAGAGAAAAAACTGAAGTCCTGTT---CCAGCACTTCATTG---A

KJ401124.1 CAATTCTCAGAAAAAGTTGAAATCATGCA---CCAACAACCCCGTA---G

XM_004252354.4 CGACCTGGAGGAGAAACCAAAATCTTGTA---CCAACAATTTGGTG---C

XM_002266972.5 TAACCCAAAGAAGAAACTGAAGTCTAACT---CTCAGAATGCAGTG---C

XM_024596482.1 TAATCCTA---AGAAGTTGAAATCTTACT---CTGAGAATGCAGTG---C

XM_006369031.3 CAATCCTG---AGGAGTTGGAATCATACT---CCGAGAATGCAGTG---C

XM_008341964.3 TAACCCAACGAAGAAGCTGAAGCCCAACT---CACAGGATCTGGTG---C

NM_001294046.1 TAACCCAACAAAGAAGATGAAGCCTAACT---CACAGGATCTGGAG---C

NM_001251371.2 C---------AACCAGCAGCAGCCTAACT---CTCAGAATGTGGTA---T

XM_006604868.4 C---------AACCAGCAG---CCTAACT---CTCAGAATGTGGTA---T

NM_001354284.1 TGCTGATCAGAATCAGAAGAAGAACAACC---TGCTGGATATGGCA---T

NM_001349033.1 TGCTGATCAGGTTCAGAAGACCAACAACT---CGCAGGATGTGGTG---G

NM_001155962.1 ACCGGTGTCGAAGAGAACGAGGAGCAGCTACGGC----------------

NM_001157201.2 A---GCGCCGAAGAGAATGAGGAGCAGCTACGGCGGCGCGCCGCCCAGCC

AK111690.1 A---GCGCCAAAGAGAATGAGGAACAACTATGGTATCGCCGTGCCC----

XM_008660266.4 TGTTCGTCCAGAACACCGCCGGCCATGC--------TGTGGCGTCT---C

XM_015788266.2 CATTCCTCCAGAACAATGCCAGTGATGC--------AATGGTACCT---C

NM_001156774.2 CATTTGTCGACAGCAATTCAAACAGCTC--------ATTTGTGCTT---C

NM_001155219.2 TATTTGTCAACTCCAATTCAAACAGCTC--------GATGGTGCCT---C

EU837255.1 CATTCATTAAGAGCAGT---ACCAACCC--------AATGGTCCCT---C

XM_009403037.2 CTGTCTGACGAAGAAACTGAAGAATAAT---GCTGGAGAAGCAGTG---C

XM_018823871.1 TGGTCCTCTGAAGAAATTGAAGAACAAT---GCCGGAGAGACGGTG---C

XM_009419716.2 TGGTCCACCGAAGAAACTCAAGAACAAT---GCAGGAGAGGCAGTG---C

XM_009419259.2 TGGTCCACTGAAGAAACTGAAAAATAAT---TCTGGAGAAGTTGTG---G

XM_009415720.2 TCCCCAGCCGAAGAAATTGCGAAACAAT---GCTGGAGAAATGGTG---C

XM_009411978.2 TGTTGCTCCAAAGAAACTGAAGAACAAT---GTAGGACGAGCTGTG---C

XM_009383333.2 TGGTACTAAAAAACAACTGAATAACATT---GTAGGACAATCTGTG---C

XM_009381868.2 TGGCCCTCACAAGAAACTGAGAAGTGAT----------------------

XM_009405824.2 TGGTGCTCAGAAGAAACTGAAACATAATTGCTCTGTAGAGACCATG---C

XM_009389352.2 TGGTCATCAGGAGAATTTGAAAAATGGTTGTGGAGCGGAATCC------T

XM_009391309.2 ----TCTCTGAAGAACTTGAAAGACAATTGTGGAGTGCAAGTC------T

NM_001084343.2 TATTTATTCTTCAAACCTTAGCCAGTGTCAAACAAAAACGGCCACTACGA

NM_001159194.1 A-GCAAAAAAAAA---AAAAAAA---------------------------

XM_015789203.1 A-GTGACCGACTT---CTGAGTTCTTCGTGTAAAGATGAATGCTCCAAGG

XM_015774497.2 GCATGGCTAGCTTAGCCTACAATTTATGTTTCTTGCATTTCTTTCTCTTT

NM_001153709.2 ATGGCTTT------CGTACGAGTTTTACACCACCGTC------ATCCCTC

NM_001254770.2 ATGGTGCCACCAGCCGACTGTACTGTAAATCACTGTTTCTGTAACTCGTT

NM_001150750.2 GTGTTGATAGA---------------------------------------

CM007650.1_142530856-142531634 --------------------------------------------------

CM007648.1_220493755-220494609 --------------------------------------------------

NM_001111800.2 ATGGCATCAATCGAGCTCCTGTC-CTGTAATTTTGTTGAACCCATCCATT

XM_023301819.1 GCAGTTTTTTTTTTAACTCCGGT-CTGTGCTTCTCAGCAGCTCG-CTGCG

XM_015775299.2 GCGCCAGTCTCAGTGAAGCTTTTGCTGAATCCAAGTTTTTCTCTAATGTA

XM_009384249.2 AGAGTAGGATTTCAGGATACTACTGTTCCTGCTCTGTTGGTTGATCTGTT

XM_009385622.2 -GTGTAGGAGTTTAGC---CTCCAGTTCATGTTACTTTGCCCTAATTGTT

XM_009388210.2 GGTGTAGGATTTAAGCTTCCTGTTGTTATTTTTTTTCCGTGTAACACTAA

XM_009389661.2 AAAATAAGGAACTATA----TTGTGTTTTTTTTTTCTTATAT-ATATTAC

XM_009393219.2 TAGT----------------------------------------------

XM_009415859.2 --------------------------------------------------

XM_008649541.3 CTGCTATCTAGGAGTATGTACTAGCAGGATGGGTTTATCTAGCTTGTTGC

XM_015782909.2 TG--TACTTTGGCGTTAAGCATTGCATTGCCAGGTGATGCATGTCATGTA

NM_001349820.1 CAGGACGTGGGCAA------CGGCATGGACG---GCGTCC---GCCTGTG

EU837258.1 CAG--CGCGAACAC------CGACATGAACGCCGGCGTCA---ACCTGTG

EU847517.1 ACCACCGCCGCCGCCGC---CGCCGCCGACCAGGGCATGG---GGCTCTG

XM_015775100.2 TTCGCCGCCGGTGAAGCGAACGTCGCCGGCGAGAGCGTGG---GGCTCTG

CT833260.1 CACATCGCCATTGGCGG--CCATTGCAAGCCATGGCATGGC--ACCTGAT

AK067060.1 AACATCGACGCGCTCTTCGGCGGCGAGGCCGTCAACATTGGTGGACTCTG

XM_015795858.2 ---GCCTTGCAGGAATAAATCAGCATTTCTTTTTTTTTCC---TTTCACT

EU847519.1 ---GCCTTGCAGGAATAAATCAGCATTTCTTTTTTTTTCC---TTTCACT

FR720457.1 -AGGCCACAAACAATAATATAATTCTTTTTCTATATATCA---TCATTAT

EU847520.1 --------------TTGTTCTGTTGTATCCTTTCATGTC-----CATTTC

NM_001143039.1 CACTAGAACAGTCATCATCTACTGCTATAGTTATAGGTCCTGTTTGGTAC

AK073133.1 TGTTTGAGC-----TTGTCTGAAGTCTGAACTCT--GCACTATGCCATAT

XM_006380178.2 AA--GACTCTGTT-----TTATTAATG-TTTAATTAGGGT-TTGGTGCCT

XM_002315454.3 TGTTGGTGCTTGA-----TGTTTAATGCTTTAATTAAGCTATTGGTGTTT

XM_008392395.2 --ATGTATGTT-------TAATTTACGTGTGT----GAAATGCGAGTGTT

XM_029095961.1 GAATGTATGTT-------TAATTCGCGTTTGT----GGAT----------

XM_002272390.4 CGTCGGTTGTCG------TCATCTGCGTTTGCTTTAAGACCCTTGGATTT

NM_001256464.1 --------------------------------------------------

XM_003546320.5 CTTGTCCTCTCGCTTTGGTAATTTCTGTTTTGTACGGTCGAATTATTTCA

NM_001279196.2 GTACATATGATGA------TAATAATAATAAGTTGCGTGCCTTAGCATGC

AY035100.1 TG--GACTGCTGATGATGTCTGTTGTGTAATGTGTAGAATGTGACCAAAT

JF968116.1 TGTTTTGTAAGTATTTAGACAA-GCGATGATGTTGCAACTTCAATCACCA

JF968119.1 TATAAT-TATCAACAAAAACAATATCTTGATCTTGCAACTTCAATCACCA

NM_130320.4 --------------------------------------------------

NM_001248198.2 ----GTACAATCAGCAATTTCAATTTGCATGTATTTAACTTTCAGACTC-

AY192368.1 ------CAGAGGATGAAGTGCAGATACTTTCTGATGAGCTGATGGCTTAT

NM_001247379.2 ------CAGAGGATGAAGTGCAGATACTTTCTGATGAGCTGATGGCTTAT

JX145122.1 -----GCAGAGAACGAAGTGCAGAAGCTTTCGGAGGAGCTGTTGGCCTAC

XM_002301454.4 ----GGAAGAAAACGAGGTACAGAAACTGACCGAGGAGTTAATTGCGTAT

XM_002320960.3 ----GGAAGAAAGCGAGGTGCAGAAGCTGACTGAGGAGTTAATGGCGTAT

NM_001320016.1 ----GGAAGAGGACGAAGTGCAGAAGCTTTCCGAGGAGCTAATGGCGTAT

NM_001328756.1 ----GGAAGATGACGAAGTGCAGAAGCTTTCTGAGGAGCTAATGGCGTAC

FQ392750.1 ----GGAGGAAATGGAAGTGCAGAAGCTGTCGGAGGAGCTAATGGCGTAT

NM_001254494.3 -----GAAAAGAACAAAGTGCTGGAGCTTTCTGAGGAGCTGATGGCGTAC

NM_001254517.2 -----GAAAAGAACAAAGTGCAGGAGCTTTCTGAGGAGCTGATGGCGTAC

AK316980.1 ATTTCGAGACACCTTACAACAACACTGAATGGGACGCTTCACTGGA----

NM_180251.3 ATCTGATGGCATACCTTGACAACGCCTTGTGGGACACCCCACTAGAAGTG

JF968115.1 CTTCAGAAGAGGATAATGGAAAGATCTCCCGCAGTGACCTTTCTGATTTT

JF968117.1 CCTCAGAAGAAGATAATGGAAAGATCTCATGTAGTGATCTTTCTGATTTC

JF968118.1 --------------------------------------------------

NM_001247584.2 CAGTTGACGGTAACACTGTGAACACGCTATCTGAAGAGCTATCGGCTTTT

KJ401124.1 CTGATGATGGAAACCCCGTTACTATGGTACCTGAAGAGCTTCCAGCTTTT

XM_004252354.4 CTAATAATGTGAACACTGAACACAAGCCACCTGAAGTATTTTCAACTTTT

XM_002266972.5 CTGTTGAAGAAAATACTCCAAATGGGCTTTCTGAGGATCTCTCAGCTTTT

XM_024596482.1 CTGTTGAAGAGAAGAATGGGAAATCTCTGTCCGAAGAGTTGCTGGCTTTT

XM_006369031.3 CTGTTGAAGAGAAGAATGGAAAATCCCTGTCTGAAGAGTTGCTGGCGTTT

XM_008341964.3 TTCCTCAGGATAATGCAGGAAAGACACTGTCTGATGAGCTCTCAGCTTTT

NM_001294046.1 CTCCTGAGGATAATTCGGGAAAGACACTGTCTGATGAGCTCTCAGCTTTT

NM_001251371.2 CTATTGAAGATGATTCTGCAAAGACACTCTCTGAGGAGCTTGTGGATATT

XM_006604868.4 CTATGGAAGATGATTCTGCAAAGAGACTCTCTGAGGAGCGTGTGGATATC

NM_001354284.1 CTGTGCAAGATGATTCTGCAAAAACTCTTTCTGAGGAGCTTGCAGACATT

NM_001349033.1 CTGCACAAGATGATTCTGCAAAAACCCTTTCCGAAGAGCTTGCAGACATT

NM_001155962.1 -----TACGGTGCGGCCATGCCGGGC---GGCTTTGACCCTGAGACGAAC

NM_001157201.2 TCGCACATGACGCGGCCATGCCTGGCTTTGGCCTTGATAAAGTGAGCTAC

AK111690.1 -----CAGGGAAATGGCATGCCTAATCTCGCACAAAACATGCCCACCTTC

XM_008660266.4 CTGCGACGGGGAACACTGGTGTTGATCTCGCCGAG------------TTG

XM_015788266.2 CTGTGATGGGGAATGCTAGCATTGATCTTGCTGAC------------CTG

NM_001156774.2 CTGCCCTGGAGAACAGTGCTGTTGATCTCACTGATGGGCTGACAGATTTA

NM_001155219.2 CTGTCCTGGAGAACAATGCTGTTGATCTCACTGATGGGCTGACAGATTTG

EU837255.1 CTGTTATGGAGAACAGTGCTGTTGATCTGCCTGAT------------TTA

XM_009403037.2 CTGCCAAGGAGATTGATGATGTCAAACTCTCTAAAGAATTATCTGCTTTT

XM_018823871.1 CGGCTGAGGAAAATGATGGTGTCAAATTCTCTGAAGATTTGTCTACTTTT

XM_009419716.2 CTACCGAGGAGAACGATGCTGTCGAACTATCTGAAGAATTTTCTACTTTC

XM_009419259.2 CTGCCAAG---AACTCTGTGGCCAAACTGTCGGAGGAGCTATCTGCTTTT

XM_009415720.2 CTGCTGAG---AACTCTGCTGCTAATATTTCCCAGGAACTAGCTGATTTT

XM_009411978.2 CTACTAAGGAAAGTGCTGCTGGGAAACCCTCCAAAGAGTTGTCTGCTTAT

XM_009383333.2 TTAATAATGAAAATGCTTCTGGAAAAACTTTCAAAGAATTGTCTGATTTT

XM_009381868.2 -----------ATTTCTGGAGTTGAACTCTCCGAGGAGTTATATGCCAAT

XM_009405824.2 CAGCTGAGAAAAATTCTGCAATTGAACTCTCTGAGGAGTTATATCCTTAT

XM_009389352.2 CTGCGGAAGAGACAACGGCAATCAAATTGTCTAAGAACTTATATGCTTTC

XM_009391309.2 CAGAAGAAGAGACCACTCCGGTCGATCTATCTGAAGACTTATCTGCTTTC

NM_001084343.2 CTATTGTTTTGAAGATTGCGATATTTTAAGTTGTAAGAATGTAGAGAATA

NM_001159194.1 --------------------------------------------------

XM_015789203.1 AATAAAATGCAATTTGTATTTCACA-------------------------

XM_015774497.2 TTTTGAGATGAGCTAGTGTGTTATACTATTTGTTCATCTTTTCTGGTAGT

NM_001153709.2 AATCCCC------TTTAAAAAAAAAAAAAAAA------------------

NM_001254770.2 AGTCACCATCGCTTTCAAAAAAAAAAAAAAAAAA----------------

NM_001150750.2 --------------------------------------------------

CM007650.1_142530856-142531634 --------------------------------------------------

CM007648.1_220493755-220494609 --------------------------------------------------

NM_001111800.2 GTTTCGTGACTACTGTGTGTCCCTGTAACTCGCAAGTTGAGGCATCAAAG

XM_023301819.1 GTGCAGAGGAATCTGTTTCATTCTGTTCGTGATTGAAACTAGTTTTACGG

XM_015775299.2 AATATGAAATTTCCAGTTAACTCAGCTTGCAATTCTTACCA---------

XM_009384249.2 GTTGCTTTTGTGTTAGAGACTCTTCCATGTGATCTTTCATTATTCCCAAG

XM_009385622.2 ATA-----TGTATCTAATACTCTGCTGTTTGATCTTCCACCTTTGCCAAG

XM_009388210.2 ATCA----TGAAACAATTGTACTTATTCACAGTATTTGTTGAAGGGTGGC

XM_009389661.2 ATAA----CGTACGAGGTATGCGTACAAAATATTTTACAGGCAATAAAAC

XM_009393219.2 --------------------------------------------------

XM_009415859.2 --------------------------------------------------

XM_008649541.3 TAAACAGAAAATTAATCTAAGAAAACAGAGATATGGTCTGTTAATTGCCG

XM_015782909.2 ATCGTGGTAATTATATGGCCGTCCTCCTAGAGCTAGCTAGCAGATTAGAT

NM_001349820.1 GAGCTTCGACGAGTTCCCCGCCGTCGACGGTTCTGTTTTCTGACGTTTGC

EU837258.1 GAGCTTCGACGACTTCCCAA---TCGACGGCGCCCTTTTCTGATGTACTC

EU847517.1 GAGCTTCGACGACGGCTGCT---GC--CTCGTCGACGTCGAGGCCAGCTT

XM_015775100.2 GAGCTTCGGCGACGACTTTC---TCAACGCGTCGTACTATTAGCTAGGGC

CT833260.1 GATGCTGATCCAGTGAACTG---GTCACTCGTTCTTGATGCGTTTTCAGT

AK067060.1 GAGCTTCGACGACATGCCAA------TGGAGTTCAGAGCTTATTGAGCAT

XM_015795858.2 GGACCCCAATAAGGTAATTTGCATACATATGTCC-CATGGATCGAACAAT

EU847519.1 GGACCCCAATAAGGTAATTTGCATACATATGTCC-CATGGATCGAACAAT

FR720457.1 AGTTTTCCCTTAGATA------ATAAAAGTGTTT-TACACTTCTCGGCAT

EU847520.1 AGACTTCCCAATGCTA-----CATAAGGATGTACATATGTATGATGTTGT

NM_001143039.1 AACTTATTTTCAGCTTCTTCACAAATTTAAGCAGAAGTTCTGCCAAACAG

AK073133.1 GGTGT---TTCACCTTCACTGTGAGGCTGACATGTGG----GCCAGACGT

XM_006380178.2 CATTGTCGTACATACCTTGTGCAATGGAGTATGAGAATAATACTTTTGTA

XM_002315454.3 GGTTGCCGAACATAG-TTGTGTAATAGACTATGACAATAAGGATTGTGCG

XM_008392395.2 GAGTA-TGTCTATGACTGGGTTTGTGTAACGTGTGTTGTTTGCTACGGTG

XM_029095961.1 --------------------------------------------------

XM_002272390.4 TACGG-CGTTTGTCGGTGCATGTATCTACTGCTTTTTTTCTGTTAATTTC

NM_001256464.1 --------------------------------------------------

XM_003546320.5 AAATTAGGTGCAACGTATCATGAGAGGGATGATTATATGTTATCATTATG

NM_001279196.2 AATTGTAATA---GTATTAGTGTTTGTTGTCTTGTGCTGTTGTTTATGCT

AY035100.1 GGACGTTATATTACGGTTTGTGGTAAAGAAAAAAAAAAAAAAA-------

JF968116.1 CCATGAACTT-GACCATTTCTTGGTCCTGCTTCTT-GTGTTGTATG---C

JF968119.1 CCATGAACTCAGATCATTTGTGGTTCTTTTTTTTTTGTGTTTTTTGG-TC

NM_130320.4 --------------------------------------------------

NM_001248198.2 --------------------------------------------------

AY192368.1 GAG--TCATTGATGAAGTTCTATGAAATACCGTAT---GTTGACGG----

NM_001247379.2 GAG--TCATTGATGAAGTTCTATGAAATACCGTAT---GTTGACGG----

JX145122.1 GAG--TCGGTCATGAAGTTCTACCAAATCCCGTAC---CTGGACGG----

XM_002301454.4 GAG--AATTACATGAAGTTTTATCAGATTCCCTAT---CTGGACGG----

XM_002320960.3 GAA--AATTTCATGAAGTTTTATCAGATTCCGTAT---CTGGATGG----

NM_001320016.1 GAG--AGCATGATGAAGTTCTATCAGATTCCGTAT---CTGGATGG----

NM_001328756.1 GAG--AACATGATGAAATTCTATCAGATTCCTTAT---CTGGACGG----

FQ392750.1 GAG--TCGGTCATGAAGTTCTATCAGATCCCCTAT---CTCGACGGCAAC

NM_001254494.3 GAA--AATTACATGAAGTTTTATCAGATTCCGTAC---TATGATGG----

NM_001254517.2 GAG--AATTACATGAAGTTTTATCAGATTCCGTAC---TATGATGG----

AK316980.1 ------TTTCCTCAACGAAGATGCTGTAAC---GA---CTCAGGACAAT-

NM_180251.3 GAAG-CCATGCTTGGCGCAGATGCTGGTGCTGTGA---CTCAGGAAGAG-

JF968115.1 GAG--GCTGAGATGCAGTTGGTTCAGACTCCCTTT---CTTGATGAGAA-

JF968117.1 GAG--GCTGAGATGCTGTCGATTGAGACTCCCTTT---CTTGATGAGAA-

JF968118.1 --------------------------------------------------

NM_001247584.2 GAA--TCCCAGATGAAGTTCTTGCAGATCCCATAT---CTCGAGGGAAA-

KJ401124.1 GAA--CCTCAGATGAATTTCTTTCATCTCCCATAT---ATGGAGGGAAA-

XM_004252354.4 GAA--TCCCAGTTGAAGTTCTATCAGACACCATAC---TCAGAAGGAAA-

XM_002266972.5 GAG--TCCCAGATGAAGTACTTTCAGATCCCTTAT---CTTGACGGAAA-

XM_024596482.1 GAC--AATCAGTTGATGAACCTTCAGATGCCAGAT---CTTGTGGGTAA-

XM_006369031.3 GAC--AGTCAGTTGAAATACCTTCAGATGCCAGAT---GCTGAGGGTAGT

XM_008341964.3 GAG--A------TGAAGTACTTTCAGACCCCATAT---CTTGATGGGAG-

NM_001294046.1 GAG--A------TGAAGTACTTTCAGACCCCATAT---CTCGATGAGAG-

NM_001251371.2 GAA--TCTGAGTTGAAGTTCTTTCAGATGCCTTAT---CTTGAAGGGAGC

XM_006604868.4 GAA--TCTGAGTTGAAGTTCTTTCAGATGGCTTAT---CTTGAAGGGAGC

NM_001354284.1 GAA--TCCCAGCTGAAGTTCTTTGAGACCCCTTCTTTTCTTGATGAAGCC

NM_001349033.1 GAA--TCCCAGCTCAAGTTCTTTGAGACCCCTTCTTTTCTTGATGAAGCC

NM_001155962.1 TA----CCAGTACCAGCCGTT-GCCT---TATGTC---GTCGAGAGCAGC

NM_001157201.2 CA----CCACTACCAGGCGTT-GCCTCCTTATTAC---GTCGGGAGCAGC

AK111690.1 GAT--CCCGAGATGAAGTATTTGCCATTACCTTAT---GTTGAGAGCAGC

XM_008660266.4 GAG--CCGTATATGAATTTCCT------------G---ATGGACGGTGGT

XM_015788266.2 GAG--CCGTACATGAAATTTCT------------G---ATCGATGGTGGT

NM_001156774.2 GAA--TCCTATATGAGGTTTCT------------T---CTGGATGGTGGT

NM_001155219.2 GAA--TCCTATATGAGGTTTCT------------T---ATGGATGGCGGT

EU837255.1 GAA--CCCTACATGAGGTTCCT------------T---CTGGATGATGGT

XM_009403037.2 GAG--TCATTCATGAAGCTTCTGCAGACACCATAT---CTGGAGGGCAGC

XM_018823871.1 GAG--TCACTCATGAAGTTTCTGCAGACACCTTAT---CTGGATGGTGGT

XM_009419716.2 GAT--TCATTCATGAAGTTTCTGCACATACCTTCT---CTGGAGGCCAGC

XM_009419259.2 GAA--TCATACATGAAATTTCTGCAAGTACCATAT---CTTGAAGGGGGC

XM_009415720.2 GAA--TCATACTTGAAATTTCTGCAAGTACCTTAT---CAGGAGGGGGGC

XM_009411978.2 GAA--CCATACATGAAATTTCTGCAGATAACTTAT---TTGCAGCCGAGC

XM_009383333.2 GAA--CTGTACATGAAGTTTTTCCAGCAACCTTAT---TCAGATGGGAGC

XM_009381868.2 GAA--CCATACATGAAGTTCCTCCCTACCCCGTAC---TTTGAGGGGAGC

XM_009405824.2 GAA--CCATGCATGAAGTTTCTCCCTGCCCCTTAC---TTTGGGAGGAGC

XM_009389352.2 GAC--TCGTACATGAATTTTCTGCAGTTTCCTTAC---ACTGAGGGGAGT

XM_009391309.2 GAC--CCATACGCGAACTTCATGCAGTTTCCGTAC---CTTGAGGGAAGC

NM_001084343.2 ATGTTTGGGAAGAACAAAGCTCGATTC-----------------------

NM_001159194.1 --------------------------------------------------

XM_015789203.1 --------------------------------------------------

XM_015774497.2 GGTGGATACATTTTCGCCGGCTAGGGTTGGAATTTGTAAATAGAAGATGG

NM_001153709.2 --------------------------------------------------

NM_001254770.2 --------------------------------------------------

NM_001150750.2 --------------------------------------------------

CM007650.1_142530856-142531634 --------------------------------------------------

CM007648.1_220493755-220494609 --------------------------------------------------

NM_001111800.2 TTTCCAGTTTAACTCTGA--------------------------------

XM_023301819.1 TTACAACGCGCAGATGGAGGATCGTCGGACACTCGGACCATTGTAACAAG

XM_015775299.2 --------------------------------------------------

XM_009384249.2 TCAAGGAATTGCTGCATTCTGTTTCGCTCTA-------------------

XM_009385622.2 TTAAGGAACTGTT---TTATGTGTTGAAGGAA------------------

XM_009388210.2 TTAGTAGTTTGTT---TTCA------------------------------

XM_009389661.2 ATATTAATTTTTCA------------------------------------

XM_009393219.2 --------------------------------------------------

XM_009415859.2 --------------------------------------------------

XM_008649541.3 GCTCTAACTAATGGCCGGCGATAATCTGTTAGGCTACCTCAGATTGACCA

XM_015782909.2 TTACTATATAA-AGTGGTAGTAGAAATATATGTCCTCCTAGAGCTAACTA

NM_001349820.1 TTACTACGTACTACTACTATTTTCTGCCTGGTGGAATCATATGTGATAGT

EU837258.1 CTCC-----ATTGATGCAGTTT--------GTGCACTCAATTGTAAGCAT

EU847517.1 GTCC--------TTTTAGGCTCTAGCCGTGGTGTCCGGCGACCCTGTGAC

XM_015775100.2 TTCCA---TAGTTCTTGTACTTTACTTGTACTGTACTGTATTGTACTGAT

CT833260.1 TTCTTGTACAGTGTTTTTCCCC-AGCAACAGTGAAGTAGTATTCAGTTAT

AK067060.1 TT----------GATTCTATTTAGGAGGGAGTGAATTATTTGGGAGGAAG

XM_015795858.2 ATGAAA---AGTAATTGTATAGTGTTGGTTGCAGAGGT-TATCAACACTT

EU847519.1 ATGAAA---AGTAATTGTATAGTGTTGGTTGCAGAGGT-TATCAACACTT

FR720457.1 CTACCG---ATAGACCGACAGCCGACAAAATCATAGTTATATATATACTC

EU847520.1 GTGCC---------TTGTTAATCAATGAA--TGTAAATATCTTTATATTG

NM_001143039.1 TTAGCTTCTGCAGCAGCTTATCAGTT-CAGCTGCTTCTCAGAATACA---

AK073133.1 CCGTTTGCCTTGCTTGCTTTTGAGCTGCAACTGGGCGTTGTGCTGCAAGC

XM_006380178.2 GCTGCACTCATTTGCATCACCTTTTTTTTTTTCTCTAGGTTTGTTTGAAA

XM_002315454.3 TGTGC-CCTGTTGTCGGTCCATGGGTTTGTATTGAAGAACAAATTACATA

XM_008392395.2 TTTATGTTTAATAGTAACTGCTTTTGTCTTTGT-------GCATGTTATA

XM_029095961.1 --------------------------------------------------

XM_002272390.4 CTTCCTAGTCCTTCCTTCTTCTTCTGCTATAATTA-GACTGCTCATTATA

NM_001256464.1 --------------------------------------------------

XM_003546320.5 ATTATAAATAAAGGCCAATTAGGGTGTGTTA-------------------

NM_001279196.2 TTTCTAAATCTTGGATTTACCTTATAAAAAAAAAAAAAAAAA--------

AY035100.1 --------------------------------------------------

JF968116.1 TTTCAATATACTCCTACTTCATCCGTTCTATTATATAATGTTTAGTTATA

JF968119.1 TTTCAATGAATTATGATCTCTTTAATTAATTACCATGTCGGTTCATTGAA

NM_130320.4 --------------------------------------------------

NM_001248198.2 --------------------------------------------------

AY192368.1 ---------------GCAATCAGTGGCGGC------GAC-----GG----

NM_001247379.2 ---------------GCAATCAGTGGCGGC------GAC-----GG----

JX145122.1 ---------------CCAGTCGCCGCCTGC------TCC-----GC----

XM_002301454.4 ---------------GCAATCCATGGCAC--------C------AA----

XM_002320960.3 ---------------GCAATCAACGGCAC--------C------AA----

NM_001320016.1 ---------------GCAGTCCACGGCGG--------CTCCTCAGA----

NM_001328756.1 ---------------GCAGTTCACGGCGA--------CTCCCCAGA----

FQ392750.1 TCAG---------CCGCGGTTCCCAACGCT------GCTCAGGAAA----

NM_001254494.3 ---------------ACAATCCACGAC-----------------GA----

NM_001254517.2 ---------------ACAATCCACGGCTCC------ACC-----GA----

AK316980.1 -GGT---GCAAACCCTATGGACCTATGGA-------GTATTGATG-----

NM_180251.3 -GA------AAACCCAGTGGAGCTATGGA-------GCTTAGATG-----

JF968115.1 -----------CTGGGCTGTTGATGCTTT-------CCTTGGCGG-----

JF968117.1 -----------CTGGGCTGTTGATGCTTT-------CCTTGGTGG-----

JF968118.1 --------------------------------------------------

NM_001247584.2 --TT---GGGATGCATCGGTTGATGCCTT-------CCTCAATAC-----

KJ401124.1 --TT---GGGATGCATCAGGTGGTAACTT-------CCTCAACAC-----

XM_004252354.4 --TA---TGGATGTACCAGTCGATGCCTT-------CCTCGATGC-----

XM_002266972.5 --CT---GGGAAGCTTCAATGGATGCCTT-------ACTCAGCGG-----

XM_024596482.1 --CT---GGGAGGCTTCTCTTGATAGCTT-------CCTTAATGG-----

XM_006369031.3 AGCT---GGGAGGCTTCTCTTGATAGCTT-------CCTTAATGT-----

XM_008341964.3 --CT---GGGATGCTTCAGTGGACGCCTT-------CCTTAACGG-----

NM_001294046.1 --CT---GGGATGCTTCAGTGGACGCGTT-------CCTCAACGG-----

NM_001251371.2 TGGG---GCGATACTTCATTGGAATCCTT-------GCTATCTGG-----

XM_006604868.4 TGGG---GCGATACTTCGTTGGAATCCTT-------GCTATCTGG-----

NM_001354284.1 TGGG---CTGATGCTGCATTGGCGTCTTT-------GCTCAGTGA-----

NM_001349033.1 TGGG---CTGATGCTACATTGGCGTCTTT-------GCTCGGCGG-----

NM_001155962.1 CCGTCAGACGGCGCGTCGACGGACGACATGGACTGCCTTCAAATGATGCG

NM_001157201.2 ---------AACGCGTCGGTGGGCAACCTCGGTCTTCTGCAGCAGGCTGA

AK111690.1 TCA------GATGAATCAATGGACAACCT------TCTGCAAAA------

XM_008660266.4 TCA------GACGACTCGATCAGCACTCT-------CTTGAGCTG-----

XM_015788266.2 TCG------GATGAGTCGATTGACACCCT-------TCTGAGCTC-----

NM_001156774.2 CCA------AGTGATTCAGTTGATAGCCT-------TCTGAACCT-----

NM_001155219.2 GCA------AGTGATTCAATTGATAGCCT-------TCTGAACCT-----

EU837255.1 GCT------GGTGACTCAATTGATAGCCT-------TCTCAACCT-----

XM_009403037.2 TCC------GACGAGGCAATCGACAGCTT-------CCTCGATTG-----

XM_018823871.1 TCA------GATGATTCAATCGATAGCTT-------CCTCAATTA-----

XM_009419716.2 ACA------GATGATTCGATCGATAGCTT-------CCTCAATTA-----

XM_009419259.2 TCA------GATGGATCTATTGAGAGCCT-------TCTCGTTAG-----

XM_009415720.2 TCC------GATGAATCTATTGAGAGCCT-------TCTCGGTAA-----

XM_009411978.2 ACA------GATGACTCAATCGACAACCT-------TCTCGGTGG-----

XM_009383333.2 ACA------GATGACTTGATTAACAACCT-------TCTCAGTGG-----

XM_009381868.2 TCA------GATGCATCAATGGACAGCCT-------ATTTGGTGG-----

XM_009405824.2 TCA------GATGCCTTAATCGACATCCT-------ATTTGGTGG-----

XM_009389352.2 TCC------AATGTGTCGCTCGATTGCCT-------GTTTGGTGG-----

XM_009391309.2 TCT------GATTTGTCAATCGACTCCCT-------ATTTGGTGG-----

NM_001084343.2 --------------------------------------------------

NM_001159194.1 --------------------------------------------------

XM_015789203.1 --------------------------------------------------

XM_015774497.2 TCAATAATAAGATTGTTTCTGGCTA-------------------------

NM_001153709.2 --------------------------------------------------

NM_001254770.2 --------------------------------------------------

NM_001150750.2 --------------------------------------------------

CM007650.1_142530856-142531634 --------------------------------------------------

CM007648.1_220493755-220494609 --------------------------------------------------

NM_001111800.2 --------------------------------------------------

XM_023301819.1 GCTTGCATACCATTTGGAAGATTTTACCATACTGAATTGGCGA-------

XM_015775299.2 --------------------------------------------------

XM_009384249.2 --------------------------------------------------

XM_009385622.2 --------------------------------------------------

XM_009388210.2 --------------------------------------------------

XM_009389661.2 --------------------------------------------------

XM_009393219.2 --------------------------------------------------

XM_009415859.2 --------------------------------------------------

XM_008649541.3 TCTCTATTTCTTATTATAAACTCATTTTCCTCGTCTATTATTTTGAACTC

XM_015782909.2 -----GTGTCTTGTAATGATTTCAGACTTCTTGGGCAACATGAGCAATGC

NM_001349820.1 GCTGCATGTGTCTGCCTGATCGTCAGATTGTACTCCACTCTGGATATTTG

EU837258.1 -CTGAATTCACTTCCATTTTAAT-GGATTCAGTTAAAAACTTAACAAAAA

EU847517.1 T--CGATCTTGTACCATGTCATGTACATAGAAGAGTGGTTTTGCCAAACA

XM_015775100.2 TGTTAACGTTGCCATAAAGCAATCTTGCTAGTTTGTAATTCTGCGTGTGT

CT833260.1 TCACTGTTCAGTCATGAGTTCATCGCAAAATATGATGTAAGTATGTGTCT

AK067060.1 AATCGATGTTGTAACTTGTAAA--ATCTCTGATGATGATCTC-TGCATCA

XM_015795858.2 ATTCGGGTTA----------------------------------------

EU847519.1 ATTCGGGTTAAAAAAAAAAAAAAAAA------------------------

FR720457.1 CCTCCGTCCCTAAATATTTGACGCCGTTGACTTTTTTTAAACATGGCTCA

EU847520.1 CTTTTGTAAAAAAAAAAAAAAAA---------------------------

NM_001143039.1 CTAAAAGCAGCCAATAAGCAGAA-GCTGCTTTTCACCAGCTTCTCAGATA

AK073133.1 CTGCAAAGTGCCTGTGTAAAGTTTGTGGGATTGTGTGTGTGGATCTTGTA

XM_006380178.2 TAAAAACCTTGGATTTGTGCCATCTACTTA--------------------

XM_002315454.3 GTGTATTTTTGACTTTGTGGAAACTTTTCTTTTACAGTTTTAATAATTAA

XM_008392395.2 TGATCAAAGTGTTTCGGTTTGTGTT-------------------------

XM_029095961.1 --------------------------------------------------

XM_002272390.4 TTATATGGGTAATAATGTAAAAACGTCTTTGCTAAA--------------

NM_001256464.1 --------------------------------------------------

XM_003546320.5 --------------------------------------------------

NM_001279196.2 --------------------------------------------------

AY035100.1 --------------------------------------------------

JF968116.1 CGTTTTTTACGTT-------------------------------------

JF968119.1 TTTGGCATGTAATTAAGAGCAGTGTTTTCTTGGATTTGTATATATTGAAT

NM_130320.4 --------------------------------------------------

NM_001248198.2 --------------------------------------------------

AY192368.1 -----TGAATCCAGCGGCGGAGACCGCCGTGGG-----------------

NM_001247379.2 -----TGAATCCAGCGGCGGAGACCGCCGTGGG-----------------

JX145122.1 -----CCAATCTCGCCCAGGAGAGCGTCCTCGC-----------------

XM_002301454.4 -----ATGGTTCCACACAGGAAAAC---CTTGT-----------------

XM_002320960.3 -----ATGGGACAACCCAGGAAAGC---CTTGT-----------------

NM_001320016.1 -----ATCCTCCGCCTCAGGAAAGCACCCTTGG-----------------

NM_001328756.1 -----ATCCTCCGCCTCAAGAAAGCACCGTCAG-----------------

FQ392750.1 -----ATGCTGGTGCTTGCGGTGGTGCTGCCAT-----------------

NM_001254494.3 -----ATAAT---GTTCAGGAAAGC---TTGGT-----------------

NM_001254517.2 -----ATAAT---GTTCAGGAAAGC---TTGGT-----------------

AK316980.1 ------AAATTCATTCCATGATTGGAG---GAG-----------------

NM_180251.3 ------AGATCAATTTCATGCTGGAAG---GAG-----------------

JF968115.1 ------GAATGGAACCCAGGATGATCT---CAG-----------------

JF968117.1 ------GAATGGAACCCAGGATTGTAT---TAG-----------------

JF968118.1 --------------------------------------------------

NM_001247584.2 ------AAGTGCAATTCAGGATGGTGG---AAA-----------------

KJ401124.1 ------AAGTGCAACTCAAAATGGTGGTGAAAA-----------------

XM_004252354.4 ------TGATGCTACTCAGGGTGTTGA---AAA-----------------

XM_002266972.5 ------GGATGCTGCTCAGGATGGTGG---AAA-----------------

XM_024596482.1 ------AGACACAACTCAGGATGGCAC---AAA-----------------

XM_006369031.3 ------AGAGACAACTCAGGATGGCAC---AAA-----------------

XM_008341964.3 ------AGACGCAACTCAGGATGGTGG---TAA-----------------

NM_001294046.1 ------TGACGCTACTCAGGATGGTGG---TAA-----------------

NM_001251371.2 ------TGACACAACTCAGGATGGTG---GAAA-----------------

XM_006604868.4 ------TGACACAACTCAGGATGGTG---GAAA-----------------

NM_001354284.1 ------AGATGCATCTCAGGATGCTGCTGGAAA-----------------

NM_001349033.1 ------AGACGCAACTCATGACGCCGCCGGAAA-----------------

NM_001155962.1 GGCTGGTGATGTTCCACAGGATGGGGCGAGCAGTGGTGGTGGTGGCGGCG

NM_001157201.2 TGATGCTCCAGCTCCACAGGATGGGGCGAGC-------------------

AK111690.1 ------TGATGCTACACAAGACGGGGCAAGC-------------------

XM_008660266.4 ------TGATGGATCCCAGGACGTGGTCAGCAA-----------------

XM_015788266.2 ------TGATGGATCTCAGGATGTGGCCAGTAG-----------------

NM_001156774.2 ------TGATGGATCGCAGGATGTTGGTAGCGA-----------------

NM_001155219.2 ------TGATGGATCACAGGATCTTGGTAGCAA-----------------

EU837255.1 ------GGATGGATCACAGGATGTTGTCAGCAA-----------------

XM_009403037.2 ------TGATGCGACTCAGGGTGAAGATGGTG------------------

XM_018823871.1 ------TGACATGACTCAGGATGAAAGTGGAG------------------

XM_009419716.2 ------TGATGTGATTCAGGAGGAAAGTGGTG------------------

XM_009419259.2 ------TGATGTGCCTCAGGATGTGAGTGAAG------------------

XM_009415720.2 ------TGATGTGACTCAGGATATGAACGGTG------------------

XM_009411978.2 ------TGATTTGACTCAAGACTTTAGCAGTG------------------

XM_009383333.2 ------TGATTTCACTCAAGATATGAGCAACG------------------

XM_009381868.2 ------TGCGGATCTCTGGAGTTTCGATGACTT-----------------

XM_009405824.2 ------TGAACTGGTGCAGGGTGGTGTGGACGT-----------------

XM_009389352.2 ------TGAACTGGCTCAGGATGATTTCAGTAC-----------------

XM_009391309.2 ------TGAATTAGCTCAGGGTGATCTGGCTGA-----------------

NM_001084343.2 --------------------------------------------------

NM_001159194.1 --------------------------------------------------

XM_015789203.1 --------------------------------------------------

XM_015774497.2 --------------------------------------------------

NM_001153709.2 --------------------------------------------------

NM_001254770.2 --------------------------------------------------

NM_001150750.2 --------------------------------------------------

CM007650.1_142530856-142531634 --------------------------------------------------

CM007648.1_220493755-220494609 --------------------------------------------------

NM_001111800.2 --------------------------------------------------

XM_023301819.1 --------------------------------------------------

XM_015775299.2 --------------------------------------------------

XM_009384249.2 --------------------------------------------------

XM_009385622.2 --------------------------------------------------

XM_009388210.2 --------------------------------------------------

XM_009389661.2 --------------------------------------------------

XM_009393219.2 --------------------------------------------------

XM_009415859.2 --------------------------------------------------

XM_008649541.3 CATTATATAAATGCAGTCTACATATTGTACAA------------------

XM_015782909.2 ATTTCGACTTCCACTTTACA------------------------------

NM_001349820.1 TGCT-ACGTACTCGTCTGGTATT-TCCTACTGTGGGTCAATGGTTCCTAT

EU837258.1 AAAA-AAAAAAAAAAAA---------------------------------

EU847517.1 AGC--ATGTGTTCGTCCTGGTTC-CCTTCGGTTATGAAAAAAAAAAAAAA

XM_015775100.2 GGTCGAAATTTTCATGTTTGTGT-TGTTTGAATCTGTAGTTGTCGTATCG

CT833260.1 TGAGATTGTTTTAGCAGTGGCTT-TTGTTTGTATAATGTATTTCTCTGTA

AK067060.1 TATGATCAATTTGAGTGCAGTTT-TGTTTTTGTAAATACGAATTCTTTAT

XM_015795858.2 --------------------------------------------------

EU847519.1 --------------------------------------------------

FR720457.1 CCGTTCGTCTTATTCAAAAACTT-CTGTGAAATATGTAAAATCATATGTA

EU847520.1 --------------------------------------------------

NM_001143039.1 AGCTGCTTTTTCAACAAGCATCAGCTATGCCA-----AACAGGGCCATAG

AK073133.1 TCCTTGTGACTTTGCAAGCTTTAATTTTGTGAGGAACAATAGTATTTTAG

XM_006380178.2 --------------------------------------------------

XM_002315454.3 AAAAAACTGTTTTGTA----------------------------------

XM_008392395.2 --------------------------------------------------

XM_029095961.1 --------------------------------------------------

XM_002272390.4 --------------------------------------------------

NM_001256464.1 --------------------------------------------------

XM_003546320.5 --------------------------------------------------

NM_001279196.2 --------------------------------------------------

AY035100.1 --------------------------------------------------

JF968116.1 --------------------------------------------------

JF968119.1 TGTG----------------------------------------------

NM_130320.4 --------------------------------------------------

NM_001248198.2 --------------------------------------------------

AY192368.1 ---CGGTGGCTCGATGGAGCTTTGGA-----GTTTTGATGAT-------G

NM_001247379.2 ---CGGTGGCTCGATGGAGCTTTGGA-----GTTTTGATGAT-------G

JX145122.1 ---TGGCGGCGCTTTGGATCTCTGGA-----GCTTCGACGAT-------G

XM_002301454.4 ---GGCTAAT-CTTTGGAACTTTGAT----GATGTTTGTGTA-------G

XM_002320960.3 ---GGGTAAT-CTTTGGAGCTTTTTT----GATGATGGTGTT-------G

NM_001320016.1 ---TGATGAT-CTCTGGAGCTTCGAT----GACGACCATAGC-------G

NM_001328756.1 ---TGATGAC-CTTTGGAGCTTCGTT----GACGACGAGAGC-------G

FQ392750.1 ---GGAAGCT-CTTTGGAGCTTTGAT----GAATTTGCAGTT-------G

NM_001254494.3 ---TGGGGAT-CTCTGGAGTTTCGATTAGCGAGGAATATATCAA-GGATG

NM_001254517.2 ---CGGGGAT-CTCTGGAGTTTTGATTAACGAGGAATATATCAAAGGATG

AK316980.1 -TCTTCTGAAGAGATCCAGTTTCATG--AGTTCCT-GTTTGCA---TTGC

NM_180251.3 -ACTTTTGAAGTGATCGA------TG--GTTCCTTAGTTTGTA---AATA

JF968115.1 -CATGATGAATCTGTGGAACTTTGAT--GATGTTCAGGTTGGA---TCCT

JF968117.1 -CCCAATGAATCTGTGGAACTTTGAT--GATGTTCAGGTTGGA---TCCT

JF968118.1 --------------------------------------------------

NM_001247584.2 -CGCCATGGACCTTTGGTCCTTCGAT--GATGTACCTTCTTTA---ATGG

KJ401124.1 -TGCTATGGACCTGTGGTCCTTTGAT--GATGTTCCTTCTTTA---ATGG

XM_004252354.4 -TGCTATGGACCTTTGGTCCTTTGAT--GAGCTTTCTTCTTTA---ATGG

XM_002266972.5 -CCCAATGGACCTTTGGAGCTTTGAT--GATCTGCCTACTGTG---GTGG

XM_024596482.1 -CGCAGTGGACTTGTGGAGCTTCGAA--GACTTCCCCTCCATG---GTTG

XM_006369031.3 -TGCAATGGACTTGTGGAGCTTCGAT--GACTTCCCCTCTATG---GTTG

XM_008341964.3 -TCCGGGGGACCTATGGACCTTTGAC--GATCTGCCCGCAATT---GTTG

NM_001294046.1 -TCCAATGGACCTTTGGAGCTTTGAT--GATCTGCCCGCAATT---GTTG

NM_001251371.2 -CCTCATGAACCTTTGGTGCTTTGAT--GACATTCCTTCCATG---GCTG

XM_006604868.4 -CCTCATGAACCTTTGGAGCTTTGAT--GACATTCCTTCCATG---TCTA

NM_001354284.1 -CCCTATGAACCTTTGGAGCTTCGAC--GACCTGCCTTCCATG---GCA-

NM_001349033.1 -CCCTATGAACCTTTGGAGCTTCGAC--GACCTGCCTTCCATG---GCA-

NM_001155962.1 ACGGCGGCGATATCTGGAGCCTTGAC--GAGCTGCTCATG------GCCG

NM_001157201.2 --GCCGGGGATATCTGGAGCCTCGAC--GAGCTGCTCATGCTGGCAGCAG

AK111690.1 --AACGAGGGCATCTGGAGCCTTGAT--GAGCTGCTCATG------GCAG

XM_008660266.4 -CA---TGGACCTTTGGAGCTTCGAG--GACATGCC---CATG---TCTG

XM_015788266.2 -CA---TGGACCTTTGGAGCTTCGAT--GACATGCC---CGTG---TCGG

NM_001156774.2 -CA---TGGACCTCTGGAGCTTCGAC--GACATGCC---CATC---GTCG

NM_001155219.2 -TA---TGGACCTCTGGACCTTCGAT--GACATGCC---CATC---GCTG

EU837255.1 -CA---TGGACCTCTGGAGCTTTGAT--GACATGCC---C------GTTA

XM_009403037.2 ------TGGATCTCTGGAGCTTTGAC--AACCTGCCACCTATA---GCGG

XM_018823871.1 ------TGGACCTGTGGAGCTTTGAT--GACCTGCCACCGGTA---GCTA

XM_009419716.2 ------TGGACCTGTGGAGCTTCAAT--GACCTGCCGCTAATT---GCAG

XM_009419259.2 ------TGGATCTCTGGAGCTTTGAT--GACCTGCCACCAGTG---GCGG

XM_009415720.2 ------TGGATCTCTGGAGCTTTGAT--GACCTGTTTCCAATC---ACAG

XM_009411978.2 ------TGGACCTCTGGAGCTTCGAC--AACCTGCCACAGTTG---GGGA

XM_009383333.2 ------TGGATCTCTGGAGTTTTGAT--GGCATGGCACACATG---GGAG

XM_009381868.2 -GCCTATGGAGGCAGTGTTTACTGAGGGGATATGAT---CCTC---GCCA

XM_009405824.2 -TC---TGGACCTATGGAATTTTGAT--GACTTGCC---CATG---GAGG

XM_009389352.2 -TG---TCAACCTATGGAGCTTCGAT--GACCTGCC---CATG---GAGG

XM_009391309.2 -TG---TGAACTTATGGAGCTTTGAT--GAGCTGCC---CATG---GAGG

NM_001084343.2 --------------------------------------------------

NM_001159194.1 --------------------------------------------------

XM_015789203.1 --------------------------------------------------

XM_015774497.2 --------------------------------------------------

NM_001153709.2 --------------------------------------------------

NM_001254770.2 --------------------------------------------------

NM_001150750.2 --------------------------------------------------

CM007650.1_142530856-142531634 --------------------------------------------------

CM007648.1_220493755-220494609 --------------------------------------------------

NM_001111800.2 --------------------------------------------------

XM_023301819.1 --------------------------------------------------

XM_015775299.2 --------------------------------------------------

XM_009384249.2 --------------------------------------------------

XM_009385622.2 --------------------------------------------------

XM_009388210.2 --------------------------------------------------

XM_009389661.2 --------------------------------------------------

XM_009393219.2 --------------------------------------------------

XM_009415859.2 --------------------------------------------------

XM_008649541.3 --------------------------------------------------

XM_015782909.2 --------------------------------------------------

NM_001349820.1 GAATTTGTTCACGTCGGCGAAACTCTGTATTGTACGGTCGTGATTGATGT

EU837258.1 --------------------------------------------------

EU847517.1 AAAAAAAA------------------------------------------

XM_015775100.2 TTTGGGGTGTGTTTGATTTGAA----------------------------

CT833260.1 ATTAATTAATAGCTGTTTTCAGTGATAAACTAAATTTTCCG---------

AK067060.1 TATGATGTTAGGTTCTT---------------------------------

XM_015795858.2 --------------------------------------------------

EU847519.1 --------------------------------------------------

FR720457.1 TACATAAAAATATATTTAACAATGAATCAAATGATAGAAAAATAATTAAT

EU847520.1 --------------------------------------------------

NM_001143039.1 CCTTGTTTGCAGCTTATTTCTATAGAATTTTGCTGGGAGGCTGGCTGTCT

AK073133.1 ATTGGTGTGTAAAATATAGAAATA-AATAATAGTAGCAACTAAATTTTGG

XM_006380178.2 --------------------------------------------------

XM_002315454.3 --------------------------------------------------

XM_008392395.2 --------------------------------------------------

XM_029095961.1 --------------------------------------------------

XM_002272390.4 --------------------------------------------------

NM_001256464.1 --------------------------------------------------

XM_003546320.5 --------------------------------------------------

NM_001279196.2 --------------------------------------------------

AY035100.1 --------------------------------------------------

JF968116.1 --------------------------------------------------

JF968119.1 --------------------------------------------------

NM_130320.4 --------------------------------------------------

NM_001248198.2 --------------------------------------------------

AY192368.1 TTAGTCGTG-----------TACAACC------AAGTTATAATGTAGTTT

NM_001247379.2 TTAGTCGTC-----------TACAACC------AAGTTATAATGTAGTTT

JX145122.1 TTCTCCGTCC----------TGCCATCTCCTAGAATCAGCATTCCCGTCC

XM_002301454.4 CTCCTCCT-------------GTCACCTCGGCACCTCTGTAACGGCGACG

XM_002320960.3 ATGCTCCT-------------GTCACTTCTGCACCTCTGTAATGGCGACG

NM_001320016.1 TTCCT-------------------GCTTCCGTGTGAT--TAATGTTATCT

NM_001328756.1 TTCCT-------------------GCTTCCGTGTGAT--TAATGTTGTCT

FQ392750.1 CTCCTCAA-------------CCCACCTCAGCTGCTCTATAA--GCATTT

NM_001254494.3 TTCTTTTCATGCATGTGTTTTGTTTCTTCGTCAATGCTGTGAAAGCTTTT

NM_001254517.2 TTCTTTTCATGCATGTGTTT-GTTTCTTCGTCAATGCTGTGAAAGCTTCT

AK316980.1 GAGAAGCCATGAGCCTCTA------TCTTGAGGGTAGTTGTGATGAAGTT

NM_180251.3 AAGCTGTGTTGGATTTTGC------TGTTGGGGGATGGTACAAGTCA--C

JF968115.1 TCTAAGGTGTTCTCATCTA------GGACCGACCGCAGCTGCTTTCGTGT

JF968117.1 TCTAAAGGACC---------------AACC--CCGCCGCTGCTTTCGTGT

JF968118.1 --------------------------------------------------

NM_001247584.2 GAGGTGCCTACTAAGCTGC---ATACACATCTTCCCTTGCTAAGTTTTGT

KJ401124.1 GAGGTATCTTTTAAGTCA------ACATGCCT--------TGAGTTTTGT

XM_004252354.4 GAGGCATCTGAGAAATGTG---TTACCTGCAG---------ATTCTGTGT

XM_002266972.5 GGGGAGTTTTCTGAGCAA----ACTTTTCCCTTGCTAGCTGGTCTTCTGT

XM_024596482.1 GGGGAGTTTATTGAGCCAA------CTTTTCTGTGCTTGCTAGGTTTTGT

XM_006369031.3 GGGGAGTTTATTGAGCCAA------CTTT-CTGTGCTTGCTAGGCTCTGT

XM_008341964.3 GAGGAACTTTCTGAGAGATG--AACCTTACCCCAGCTTTCCAGTTTATGT

NM_001294046.1 GGGGAGTTTTTTAAGCGATG--AACCTTTCCCCACCTTTCTAGTTTATGT

NM_001251371.2 GTGGAGTTTTCTGAACCG-------CTTTTTCTTTGGTCTCCCTGTGTGT

XM_006604868.4 GCGGAGTTTTCTGAACAG-------CTTTTTCTTTGGTCTTCCTGTGTAT

NM_001354284.1 --GGAGTCTTCTGAACAC-------C-TTCATAT----CCCTTTTTATGT

NM_001349033.1 --GGAGTCTTCTGAACAC-------CCTTTATCT----CCCCTTTTATGT

NM_001155962.1 CTGGCGCTTACTGAGA-----A----AAGCTATGTGCATCGCATTCGCAT

NM_001157201.2 CTGGTGCTTATTGAGA-----ACCGCAAGAAAAAAAAAATGTGTTGGCGC

AK111690.1 CTGGTGCCTACTGAGG-----AGACGAAATGTTCTGGTCAGTGTGGTCTG

XM_008660266.4 CTGGTTTCTACTGAGG---CTGAGGCCCAGCGACTGGTGCTTGTGTACAT

XM_015788266.2 CCGAGTTCTACTGAGGGGTTTGGGGTGTAGCAACTGGTGCCTGTATATAT

NM_001156774.2 GCGATTTCTTTTGAGGATTTTGAAGCTTGGTCATAGGAGTATGTACATAG

NM_001155219.2 GCGATTTCTTCTGAGGAATTTGAAGCTTGGCCATAGGACTATGTACATAG

EU837255.1 GCGATTTCTATTGAGGAATTCGAAGTCTTCTAGTCGGAGCATGTACACAG

XM_009403037.2 AAACCATTTACTGA-------GGACCAAAGATTATGGCAAATGGCCTTCT

XM_018823871.1 GCAGCGTTTACTGAT------GGACCTTAAATTAAGGCAAATGGTCTTCT

XM_009419716.2 GCAGCATATACCGAG------GGACCTTGAATTATGGCTAATTGTCTTCT

XM_009419259.2 TCGGCAGTTACTGAG------AACATTGATATTCCGCCAAATAATGCTCT

XM_009415720.2 GCAGTGATTACTGAG------GAACCACATCTTCTCACAACTG--GTTCT

XM_009411978.2 GCAGCATCTTCTGAG------GATCCTCGAGTTATGGCATCCATTATCAT

XM_009383333.2 GCAGTGTCTTCTGAG------GGTACTCAAAATATGACATTCAGTGTTGT

XM_009381868.2 TTGGTGTTCCAGAAAGAGCAAATAAATAATACAATGTTGATGAAGGATTT

XM_009405824.2 CCAGTGTTTACTAA------AATAAATGA-ACCATGTTGATGAAGGATAT

XM_009389352.2 GCAGTGTCTACTGA-------GAGACTTCGGCTGTCGCTACCGGTGTTCT

XM_009391309.2 GCAGTCTTTACTGA-------GGGACTTGGACCTTAGCCACAGGTGCTCT

NM_001084343.2 --------------------------------------------------

NM_001159194.1 --------------------------------------------------

XM_015789203.1 --------------------------------------------------

XM_015774497.2 --------------------------------------------------

NM_001153709.2 --------------------------------------------------

NM_001254770.2 --------------------------------------------------

NM_001150750.2 --------------------------------------------------

CM007650.1_142530856-142531634 --------------------------------------------------

CM007648.1_220493755-220494609 --------------------------------------------------

NM_001111800.2 --------------------------------------------------

XM_023301819.1 --------------------------------------------------

XM_015775299.2 --------------------------------------------------

XM_009384249.2 --------------------------------------------------

XM_009385622.2 --------------------------------------------------

XM_009388210.2 --------------------------------------------------

XM_009389661.2 --------------------------------------------------

XM_009393219.2 --------------------------------------------------

XM_009415859.2 --------------------------------------------------

XM_008649541.3 --------------------------------------------------

XM_015782909.2 --------------------------------------------------

NM_001349820.1 GCCAAATTAAATAAGCCTGTGAACCTGGTTCTATGTA-------------

EU837258.1 --------------------------------------------------

EU847517.1 --------------------------------------------------

XM_015775100.2 --------------------------------------------------

CT833260.1 --------------------------------------------------

AK067060.1 --------------------------------------------------

XM_015795858.2 --------------------------------------------------

EU847519.1 --------------------------------------------------

FR720457.1 AATTACTTAAATTTATTAAATAAGAAGAGCGGTCAAATATATTTAAAAAA

EU847520.1 --------------------------------------------------

NM_001143039.1 C-------------------------------------------------

AK073133.1 TTT-----------------------------------------------

XM_006380178.2 --------------------------------------------------

XM_002315454.3 --------------------------------------------------

XM_008392395.2 --------------------------------------------------

XM_029095961.1 --------------------------------------------------

XM_002272390.4 --------------------------------------------------

NM_001256464.1 --------------------------------------------------

XM_003546320.5 --------------------------------------------------

NM_001279196.2 --------------------------------------------------

AY035100.1 --------------------------------------------------

JF968116.1 --------------------------------------------------

JF968119.1 --------------------------------------------------

NM_130320.4 --------------------------------------------------

NM_001248198.2 --------------------------------------------------

AY192368.1 AAT----------------------------TATTGTTTTGTTTAAACTT

NM_001247379.2 AAT----------------------------TATTGTTTTGTTTAAACTT

JX145122.1 CCT----------------------------TTTTGGTTCTTCTGTTCTG

XM_002301454.4 GCAG--TGTCT---------------TGTCTTTTCATTTTTT---TGTTA

XM_002320960.3 GCG-------------------------------CAGTATCT---TGCTG

NM_001320016.1 GCT--------------------------TTTATTACTTTTA---TTTTA

NM_001328756.1 GCT--------------------------TTTATTAGTTT-G---TTTTA

FQ392750.1 GGT---------------------------TAATTATTTTTG---CATCT

NM_001254494.3 GCTT--TCTCCCTCCATTCTGCTGACTAAACTAACATCTCTGGCGTTTTG

NM_001254517.2 GCT-------------------------GACTAATATCTTTG-CGTTTTA

AK316980.1 A------AGTAG---AGGCTTATTTTTAGGGGTTGTGGTAGTTTTTGTTT

NM_180251.3 A------CCTCA---AGCTCTATGCATTGGTATCTCATGAGCCTCTCTTC

JF968115.1 A------AATAA---CCAGACGTGTTAGTGATTTCCGTAAC---------

JF968117.1 A------AATAA---GAAGACATGTTAGTGATTTTCATGATTAAGAAGAA

JF968118.1 --------------------------------------------------

NM_001247584.2 A------AATAA--CG-CTTCATTTGAGTGAAGTTTGCGCCTGCGTTTA-

KJ401124.1 A------AATAA--GG-CTTCAT---------------------------

XM_004252354.4 A------AATAA--GGGCTTCATGTGAAGATAAGGTGTCTGTGTTAAGG-

XM_002266972.5 A------AATAA--AG-CTACAT--GAATGGTTGTTGCCAGCGTTGCAG-

XM_024596482.1 A------AATAATAAGGCTACATGTTAGTGAGTTTCATCTCTACA-----

XM_006369031.3 A------AATAACAAGGCTACATGCTCGTGAGTTTCATCTCTATGTTTGG

XM_008341964.3 A------AATA---AAGCTACATGAT------------------------

NM_001294046.1 A------AATA---AAGCTACATGTTAGTGAGTTTTTCAGTCCTCCGTG-

NM_001251371.2 A------AATAA---AGCTACAAGTTGTT---TTTC--------------

XM_006604868.4 A------AATAA---AGCTACAAATTATT---TTTCATTTTCCTTCTGGG

NM_001354284.1 A------AATAA---AGCTACAAG--AAT---TGTGATCGTGATGTTGG-

NM_001349033.1 A------AATAA---AGCTACAAG--AAT---TGTGATCGTGATGTTGG-

NM_001155962.1 G------TGCTAGACGGCTACTT--GCTGTG--------CCGTGAATGGA

NM_001157201.2 -------TGCTAGCTGGAGACGA--GACGAG--------AGGGCGAGAGG

AK111690.1 T------CACTAGCAAACCATGTTGAATGTGTATGGCCAAGATGAAGAGC

XM_008660266.4 AGGGGGGGACAAAG--GGAATAA-TG-TTCTGGAGATG-AAGAAACG-CT

XM_015788266.2 A----AGGACAAAT--GGAATAAACA-TTCTGGACATCCAAGAAGCGGCA

NM_001156774.2 G------GACTAGG--GGAATAAAGA-CCGGGGGAGATTGGGAAGCA-CC

NM_001155219.2 G------GACTAGG--G-AATAAAGA-CTGGG--AGATTGGGAAGCA-CC

EU837255.1 G------GAAAAAAAAGGAATAAATA-CTATTGGAGATTGGGAGGCA-CC

XM_009403037.2 --------TCAAATAAATGTG---------------------------CC

XM_018823871.1 --------GTAAATACGTGTGTTGTTGTTTTCCTCTTTGTTCCAGGAACC

XM_009419716.2 --------GTAAACAAGGA----------------------------ACC

XM_009419259.2 --------GTAAATAAGATGCGACC--------------------GAATC

XM_009415720.2 --------GTAAACGGGATGCAAGC--------------------GAATC

XM_009411978.2 --------GTAGATAACATATAAGT---GGAATGAAAGTGTTGGAGGATA

XM_009383333.2 --------GTCGATAGGATATAA------AAATGCTCTCCTTTAATAAAA

XM_009381868.2 T------GGAGGACTGGAAACATTGG---CAAGCTGTCATGAAGTTCTTG

XM_009405824.2 -------GGAGGTTTGGAAACATTGG---CTGGCTATCATGAATTCATTG

XM_009389352.2 --------GTAAATAGGGAGAAAAAA---TAAGC-ATTCCAAAGGTGACA

XM_009391309.2 --------GTAAATAGGGAGAAAAA----CAAGC-ATCCCAAAGATGGCA

NM_001084343.2 --------------------------------------------------

NM_001159194.1 --------------------------------------------------

XM_015789203.1 --------------------------------------------------

XM_015774497.2 --------------------------------------------------

NM_001153709.2 --------------------------------------------------

NM_001254770.2 --------------------------------------------------

NM_001150750.2 --------------------------------------------------

CM007650.1_142530856-142531634 --------------------------------------------------

CM007648.1_220493755-220494609 --------------------------------------------------

NM_001111800.2 --------------------------------------------------

XM_023301819.1 --------------------------------------------------

XM_015775299.2 --------------------------------------------------

XM_009384249.2 --------------------------------------------------

XM_009385622.2 --------------------------------------------------

XM_009388210.2 --------------------------------------------------

XM_009389661.2 --------------------------------------------------

XM_009393219.2 --------------------------------------------------

XM_009415859.2 --------------------------------------------------

XM_008649541.3 --------------------------------------------------

XM_015782909.2 --------------------------------------------------

NM_001349820.1 --------------------------------------------------

EU837258.1 --------------------------------------------------

EU847517.1 --------------------------------------------------

XM_015775100.2 --------------------------------------------------

CT833260.1 --------------------------------------------------

AK067060.1 --------------------------------------------------

XM_015795858.2 --------------------------------------------------

EU847519.1 --------------------------------------------------

FR720457.1 ATCAACGGCGTCAAATATTTAGGGACGGAGGGAGTATATGATATTTGCCT

EU847520.1 --------------------------------------------------

NM_001143039.1 --------------------------------------------------

AK073133.1 --------------------------------------------------

XM_006380178.2 --------------------------------------------------

XM_002315454.3 --------------------------------------------------

XM_008392395.2 --------------------------------------------------

XM_029095961.1 --------------------------------------------------

XM_002272390.4 --------------------------------------------------

NM_001256464.1 --------------------------------------------------

XM_003546320.5 --------------------------------------------------

NM_001279196.2 --------------------------------------------------

AY035100.1 --------------------------------------------------

JF968116.1 --------------------------------------------------

JF968119.1 --------------------------------------------------

NM_130320.4 --------------------------------------------------

NM_001248198.2 --------------------------------------------------

AY192368.1 TTCATA---------ATTTTATTTTATCGAATTAGGAAGAATTG--AGTT

NM_001247379.2 TTCATA---------ATTTTATTTTATCGAATTAGGAAGAATTG--AGTT

JX145122.1 ATTTCA---------ATTTTCCTTTTCTGTCGTTTCAATTCCTG--TGTT

XM_002301454.4 TTTCAGATGTTT---TTTGTAAACTGGCAA-TTGATGATTAATT-ATGTT

XM_002320960.3 GT-------------TTTGTAGATTGGGAAATTGATGATTAATT-ATGGT

NM_001320016.1 ATTTTGGTG------GTTGTAAATTGAGTTGGTCAGGGGAAATTGATGAT

NM_001328756.1 ATTTAAGCG------ATCGTAAATTGAGTCAGTCGGGCAAAATTGATGAT

FQ392750.1 TTTAGGTCG------TTTGTAAATTGGGTCATGGGAGGAAATTGTTAGAT

NM_001254494.3 GCAGGGATGCACAGGAATTTAAATTGAGTGCTTCGGTAGAAAT----GGT

NM_001254517.2 GCAGGGATGGACAAAAATGTAAATTGGGTGCTTCGGTAGAAAT----GGT

AK316980.1 TAGTGAA------TCTTTTGAATTCGTTTGTGTTTTGTTTTTGTTACTTT

NM_180251.3 CATAGAGAGTTTCTCTTTTAATTTTGTCGAAATAAAAAAGGTGTGATGAA

JF968115.1 ------------------CATATTTTCTCGGCCTGTTTTGCTTATTCTTC

JF968117.1 GACTATCTTTTTATTAAACATTTTTTCTTGGCCTGGTTTATCTTCCTTTT

JF968118.1 --------------------------------------------------

NM_001247584.2 ---CGTTTATCACCAAACTAAAAGACTATATATGTGTTGTATTAATTTAT

KJ401124.1 --------------------------------------------------

XM_004252354.4 ---AGGAGGATGACAAGTTGACAACCATTCAAGCATGTTTAGGATTCCAT

XM_002266972.5 ---TTTGAGTTCACGGTTGGACATGCATAAGGCTTGGCTGAAGAGTGTTT

XM_024596482.1 -------AGTTATTGTTTTACTCTATTATGAGTTTCATCGATAATTGCCT

XM_006369031.3 --CTCTTAGTTACAGATGAAGTTTGTT-CGAAGCATATCAATAATTGCCT

XM_008341964.3 ----------------------------------------GTGTT-----

NM_001294046.1 -------AGCTTCTACATTGTTTCATTATTGGTCTC----GTGTTCGCTC

NM_001251371.2 -----------TCTGTTTTTGATTTTTGTTGCATTTGTTGTTCTCTTGTT

XM_006604868.4 --TTCATTAATTTTGTTTTTGGTTTTTGTTGCATTTATTGCTCTCTTGTT

NM_001354284.1 ---TGATGGAATCCACAGCAAGAAAAC-CAACCTGCTTAAAGCATATGTG

NM_001349033.1 ---TGATGGAGTCCACAGCAAGAAA------CCTGCTTAAAGCTTATGTG

NM_001155962.1 GTGTTTC-TCAGCTTCGTTTTGCTTTTAATTTTTTGTTATGCCAGG-TCT

NM_001157201.2 CAGTTGCATCCGCCATCCGCTATCGTTTTGTCTCTCCTATATCTAG-TA-

AK111690.1 TGGTGATGTCTGCTATGTTTTGTAGAGGGATGCTACCAAAGTAATGCTAG

XM_008660266.4 TGCGTGGGC---GTGCCTGCAGGCACGC-GTGTAGTAGCTGCGGTATTAG

XM_015788266.2 TGTGTCTGTCGGGCGCTTCTAGTTGCGCTATATAGCTATGTTAGTATGTT

NM_001156774.2 TGCTTG------GCACCTTGGGGGTAGC-ATTTGCTCGTGTCTAGCTCAG

NM_001155219.2 TGCTTG------GCACCTTGGGG-TAGC-ATATGCTCGTGTCTAGCTCAG

EU837255.1 TGCATG------GCACCTTGGGGGTAGC-ATATCGTTATGTTTAGCTTAG

XM_009403037.2 AGTATTGATGGAAGAC---TTGAAC-CAATGATGTCCACAATGATTATAT

XM_018823871.1 AGTGTCGATGGATGTTATGCTGAAC-CAGGGATTTCCTTTATCATGATTT

XM_009419716.2 TGTGTCGACGAAGGATATGTTGAAC-CAGGGTTGTCCGCTAGGATGATCT

XM_009419259.2 CATGCTGATGGA-GATATGGAGAAC-AAGACGTATGAGCTATGATCATAG

XM_009415720.2 AACGCTGACGAC-GATACAGAGAAC-CAGTTATGCTATATATGATCACTG

XM_009411978.2 TAAAATTGTTGCATGGACTATGATC-TATGTCACTGTACTAT-ATCTCTG

XM_009383333.2 TGTATGCTTTTTATG------GTTC-TGTTTATCTTTTTTCT-CTTGCTA

XM_009381868.2 TAATTTGCCGT--GCAAAAGTGCCTTCAAGAACACTTCTTGTTAT---CT

XM_009405824.2 TGATTTGTCTT--GCAAAGGTGTCTTCAAGGATGCTTCTTATAATTGCCT

XM_009389352.2 CACGTTTTCTTTGGCCATGTTGGCT-TGCAAGTATCTCCT-TGTTCGGAG

XM_009391309.2 CTCTTTCTCCT--GCTGTTCTGCTTATATGTATATCTCCT-TGTTTGGAG

NM_001084343.2 --------------------------------------------------

NM_001159194.1 --------------------------------------------------

XM_015789203.1 --------------------------------------------------

XM_015774497.2 --------------------------------------------------

NM_001153709.2 --------------------------------------------------

NM_001254770.2 --------------------------------------------------

NM_001150750.2 --------------------------------------------------

CM007650.1_142530856-142531634 --------------------------------------------------

CM007648.1_220493755-220494609 --------------------------------------------------

NM_001111800.2 --------------------------------------------------

XM_023301819.1 --------------------------------------------------

XM_015775299.2 --------------------------------------------------

XM_009384249.2 --------------------------------------------------

XM_009385622.2 --------------------------------------------------

XM_009388210.2 --------------------------------------------------

XM_009389661.2 --------------------------------------------------

XM_009393219.2 --------------------------------------------------

XM_009415859.2 --------------------------------------------------

XM_008649541.3 --------------------------------------------------

XM_015782909.2 --------------------------------------------------

NM_001349820.1 --------------------------------------------------

EU837258.1 --------------------------------------------------

EU847517.1 --------------------------------------------------

XM_015775100.2 --------------------------------------------------

CT833260.1 --------------------------------------------------

AK067060.1 --------------------------------------------------

XM_015795858.2 --------------------------------------------------

EU847519.1 --------------------------------------------------

FR720457.1 TTTATTTTCTAATATTCACTATACAAAACGCCTAATAGAGAATAGCTCAT

EU847520.1 --------------------------------------------------

NM_001143039.1 --------------------------------------------------

AK073133.1 --------------------------------------------------

XM_006380178.2 --------------------------------------------------

XM_002315454.3 --------------------------------------------------

XM_008392395.2 --------------------------------------------------

XM_029095961.1 --------------------------------------------------

XM_002272390.4 --------------------------------------------------

NM_001256464.1 --------------------------------------------------

XM_003546320.5 --------------------------------------------------

NM_001279196.2 --------------------------------------------------

AY035100.1 --------------------------------------------------

JF968116.1 --------------------------------------------------

JF968119.1 --------------------------------------------------

NM_130320.4 --------------------------------------------------

NM_001248198.2 --------------------------------------------------

AY192368.1 TTTATAATTTAATCAATTGTGTAAAACTATGTTTCTAATTCATTAATATT

NM_001247379.2 TTTATAATTTAATCAATTGTGTAAAACTATGTTTCTAATTCATTAATATT

JX145122.1 TG-GCAGTCTGTAAATTTGGAAAGATTAATGATTCTGGGAGATTGGCATC

XM_002301454.4 ATTTTCTTTTTTGTTGTGTGAAATATGTCTTGTTTCAATGAACAGGGATT

XM_002320960.3 TTTT---------------AAGGATTGTGTTGTTTTAATTAAGAGGAATT

NM_001320016.1 TTTG---------------ATCATTGGTTTCTTCCTGATTAATTTGAATT

NM_001328756.1 TTTG---------------ATCATTAGTTTCTTCCTGATTAATCTGTATT

FQ392750.1 TTCCTATTCCCCCTTAATTAGGATGATTTGCTGCTAAATTTTGTCGGATA

NM_001254494.3 CTCATTAGTTAG---TGCTATGCTATCATCTGGTTTTGTTGAATTGTAAT

NM_001254517.2 CTCATGATTT-----TCCGACTTTTTTTTTTTTTTTTACCAATTTATTTA

AK316980.1 ATGCCCCAAAACTC-----------CTTTAACATTTGTCATAATG-TGTT

NM_180251.3 GTAAAT-AGAGGTA-----------TAATAATATCTATC-TATTA-AGTC

JF968115.1 ATAAGCCAGTGACCGTTT------CTCGTCGTTTGGTGCAGGGAT-GTCT

JF968117.1 A---ATGAGTGGTTATTT------CTCATCGTTTGATGCAGGAAT-GTCT

JF968118.1 --------------------------------------------------

NM_001247584.2 T--CAAAATTTACT--------CGTTTGATATAT-GTAAGTATGT-ATCC

KJ401124.1 --------------------------------------------------

XM_004252354.4 G--GATGGTGTACT--------GAGTAGCTGTTTTGGTTGTATAG-ACCA

XM_002266972.5 T--CTTAGGTAGCT--------A--TTGTTATATGGGGAGTAAAT-A--G

XM_024596482.1 CCTTTCATTTTGCTGCTCGTAGGAATGGTTGTTTATCTGAGATGG-GAAT

XM_006369031.3 T-TCACGTTTTGTTGCTTGTAGGGATGGTTGTTTATCTGAGATGG-GGCT

XM_008341964.3 -------------------------------TCGGGTTGGAGTAC-ATCT

NM_001294046.1 C----CATTTTATC-----CAGAAAGAGTTGTCGGGTTGGAGTGC-AACT

NM_001251371.2 GGACACATCCCCCC-----------ATGTGTCTGAAAAAAAAAAT-GTAA

XM_006604868.4 GGACACATCTCCAT-----------GTGTCTGAAAAAAAAAGAAT-GTTA

NM_001354284.1 GAGTTTATTT-ATC-----------TTGTAGCTAATGCAGTA----TAGG

NM_001349033.1 GAGTTTATTTTATC-----------TTGTAGCTAATGCAGTAGTA-TAGG

NM_001155962.1 C-CTGCT-TGTGCTTGTGGTGATGATGCAGG--AGAGCCATG--------

NM_001157201.2 ---TATA-TATGCTTCTGGCAGTCGAACTCT--ATGGCAATGCG-----T

AK111690.1 A-ATATTACAAGCTGCTATCAGCTGTACTCTCTATGACAATGTT-----T

XM_008660266.4 T---ATATATGCTTAGAT--GTTCAGTCACTTC--CTTTAAGTA--CAAT

XM_015788266.2 A---GTATGTGCTGTGTCTAGCTTAGATGCTGAAGTCTCAAGTA--CTAT

NM_001156774.2 AGATGCAGAAG-TTGCATT--CTGAAACTACTTGGTTATCG-AC--CTGT

NM_001155219.2 A--TGCAGAAG-TCGCATT--CTGAAACTCTTTGGTTATCG-AC--CTGT

EU837255.1 A--TGCAAAAGGCTGCATC--CTGAAACTCTTTGGTGATTGGAC--CTGT

XM_009403037.2 T-ATGAACCT-CTGATTTATGCTACT-TGCT---ATACAT-CTA-----T

XM_018823871.1 A-GTAAACCTTCTGAGTTATGCTACTATGTTTAGGTATTA-TCA-----T

XM_009419716.2 A-ATGAACCT-TTGATTTATGCTACTATCTATCGCCACTAGTTA-----T

XM_009419259.2 G-TTGGAACG-TTGGTTTATGCTATTATGTT-AGATCTTATGTA-----T

XM_009415720.2 G-TCGGAACT-TTGGTTAATTCTATTATCCC-CGGCGCTAAATA-----T

XM_009411978.2 T-TTGAGAGTGTTCAAGTGTTTTGGTCCTT-TTGGATTCCTCTA-----A

XM_009383333.2 G-TTGAGCTGTTTCTCCTGTTTTTTTTCATATTGGAGGGAGCTT-----C

XM_009381868.2 T-GTGATGTATCTCAATTATTAGTCTTTGGACCCGTTTCTCGTT--GTGT

XM_009405824.2 A-GTTAGGTGTTTCAATTTTTAGTTTTTGGACAAGCTTTTCATT--CGAT

XM_009389352.2 A-ACAAATTGGTTCTTTCCCTTAGATTTAGTTTCCTATCTCATG--AACC

XM_009391309.2 A-ACAA-TTGGTTATTTTCCTTACATGTCATTTACTTTCTCTTGGAAACC

NM_001084343.2 --------------------------------------------------

NM_001159194.1 --------------------------------------------------

XM_015789203.1 --------------------------------------------------

XM_015774497.2 --------------------------------------------------

NM_001153709.2 --------------------------------------------------

NM_001254770.2 --------------------------------------------------

NM_001150750.2 --------------------------------------------------

CM007650.1_142530856-142531634 --------------------------------------------------

CM007648.1_220493755-220494609 --------------------------------------------------

NM_001111800.2 --------------------------------------------------

XM_023301819.1 --------------------------------------------------

XM_015775299.2 --------------------------------------------------

XM_009384249.2 --------------------------------------------------

XM_009385622.2 --------------------------------------------------

XM_009388210.2 --------------------------------------------------

XM_009389661.2 --------------------------------------------------

XM_009393219.2 --------------------------------------------------

XM_009415859.2 --------------------------------------------------

XM_008649541.3 --------------------------------------------------

XM_015782909.2 --------------------------------------------------

NM_001349820.1 --------------------------------------------------

EU837258.1 --------------------------------------------------

EU847517.1 --------------------------------------------------

XM_015775100.2 --------------------------------------------------

CT833260.1 --------------------------------------------------

AK067060.1 --------------------------------------------------

XM_015795858.2 --------------------------------------------------

EU847519.1 --------------------------------------------------

FR720457.1 ATGTGCCGATTCATTTTAAACCAACACGTATGAACATTTTTCCACCTTCA

EU847520.1 --------------------------------------------------

NM_001143039.1 --------------------------------------------------

AK073133.1 --------------------------------------------------

XM_006380178.2 --------------------------------------------------

XM_002315454.3 --------------------------------------------------

XM_008392395.2 --------------------------------------------------

XM_029095961.1 --------------------------------------------------

XM_002272390.4 --------------------------------------------------

NM_001256464.1 --------------------------------------------------

XM_003546320.5 --------------------------------------------------

NM_001279196.2 --------------------------------------------------

AY035100.1 --------------------------------------------------

JF968116.1 --------------------------------------------------

JF968119.1 --------------------------------------------------

NM_130320.4 --------------------------------------------------

NM_001248198.2 --------------------------------------------------

AY192368.1 ATATTGGATATGTTGTTATCAAAAAAAAAAAAA-----------------

NM_001247379.2 ATATTGGATATGTTGTT---------------------------------

JX145122.1 CTCCATTGTCCCCCAAAAAAAAAAAAAAAAAAAAAAAAAAAA--------

XM_002301454.4 TAACTTCTTGAATTTGCTGTCGAAATGTAAATTTTATGAGTTGATTGTCA

XM_002320960.3 GAACTTCTTGAGTTCGCTGTCAAAATGTAATTTTTACAAGTTAACTGTCA

NM_001320016.1 GTAGGTGTTTAATGTTTAATTAGTTTGT------TCTTAATTACCCAAAG

NM_001328756.1 GGAGGCGTTTAATCTTTGATTAGATTGT------T---AATTACCCACAA

FQ392750.1 GAAGTTGAATAAACTTTTGATCCTGTATTCCAGTGACTTCTTAATATCGA

NM_001254494.3 TTTTTGTTTTGGTGGTATGTTGAACAGTAATTTTACTGGATATATTCAAG

NM_001254517.2 ATATTAATTTATTTGTTTCTAGAAC-------TTAG-GGCTATCATCTGG

AK316980.1 TGAACCTCTCATCTGTTTAATCAAATAAATCTTCTTTGTAAAAAAAAAAA

NM_180251.3 T---TGTTTTGTTCTTTCATTTTTGTATTTCTTTTCTATTTAAAAGACAG

JF968115.1 CTTTTGTTGTTGTTGTATGTCGTTAAGAGTACGCTTGAGGCTCTGGAT-G

JF968117.1 CTTTTGTTGTTGTTGTTCG--------AGTAAACTTGAGGGTTTGGAT-G

JF968118.1 --------------------------------------------------

NM_001247584.2 ---TTGTTTTCATAAGTCAATGT-CTTATTTTCTTACAGTCAAAAAAAAA

KJ401124.1 --------------------------------------------------

XM_004252354.4 ---ATAGGTGGGTAA-TTAGTTT-ATTAACAACTGTTGACAACAAGCTGA

XM_002266972.5 ---GTATTTAGGTAT-CTTTTAC-TTTCAAGACTTTGAATTGGCACTTTG

XM_024596482.1 GGAGTACAA-CACAGCTGAACATGCTTAAAGCCTCGGTGTGGGAGAAGCG

XM_006369031.3 GGAGTACAAACACAGCTGAACATGCATAA-GCCTTAGTATGGGAGAAGCA

XM_008341964.3 ---GTGCAA--GCTGTTGA-CATGGATAAGGCTTGTACG-GGGGAGCATT

NM_001294046.1 ---GTGTAG--GCTGTTGAACATGCATAAGGCCTGTGCG-GGGAGTATTT

NM_001251371.2 TTTCCATTTTTGTTGCTTTCTGGAATGGTTGTCATCCGTGTTGAAGATGG

XM_006604868.4 TTTCCATTTCTGTTGCCTTCTGGAATGGTTGTCATCTGTGTTGAAGATGG

NM_001354284.1 ACTATATATAGGTT--TTTAT---A-GGGTATCCTTTGTGTGAAA-----

NM_001349033.1 ACTATATATAGGTT--TTTATTATA-GGGTATCCTTT-TGTGAA------

NM_001155962.1 GTGGGTACTATGCTTT----TGCTGCC----AGTGGAACTG------TAA

NM_001157201.2 GTGTATAGTGTGTTGC----TGCCGTCTATGATTGGAAGCGAG--CTCGA

AK111690.1 ATACTATGTTTGCTGTATGGTGTGGTCTATGATTTGAAGTATG--TTGGA

XM_008660266.4 TTGGCGCTGGA--CATGTACCTTATTTTACTATGTATCCGTGACAACAGC

XM_015788266.2 TTGGCAGTGAAACTATCTATCTGTAACTGCTATATGAGGCTGGAACAAGT

NM_001156774.2 TCCCTATTTTAGCTATCTCTTTATGAGAGCCATTTATGAGACT---GAAC

NM_001155219.2 TCCCTATTTTAACTATCTCTGTATGAGAGCCATTTATGAGACT---GAAC

EU837255.1 TCCCTATCC--GCTGTCTATGTATGGCAGCCATCTATGAGACTCAAGAAC

XM_009403037.2 TTCTAGTTTCTGTTCTCT--CTATGTGATG-TGTTTATG------CTCAG

XM_018823871.1 CGGTAGTCTCTATTCTGTTGCCATGTGAAG-TGTTCCTG------TCTAG

XM_009419716.2 GTCTATTGCCTGCTCTGTTGCCATGTAAAG-TGTTTCTC------TCTAG

XM_009419259.2 CG-TGT-TTATGCTTGCCTTGTGTGTTTTCTTGTTGAGT------GCCAA

XM_009415720.2 CT-TATGTTATG-TTGCCTCCTAAATGTTCCTGTTAAAC------ACTGA

XM_009411978.2 TGTTAGT-CAAATTATCAGTATAGTAGGAAATGCTGAAT-------ATG-

XM_009383333.2 TGCTGACGCAGGATGTAAG-ATTGTCGCGTAGACTGATT------TATGC

XM_009381868.2 AATAAATA-ATGCTTTCTTGCAAGTTAATTCCATGTTGA------TTTGG

XM_009405824.2 GATGAATA-AATGCTTCTTGCAAGTTAACTC-ATGTTGC------TATGG

XM_009389352.2 AATTAGA--GAAGCTTATGGAGGGTCAGAATTGTTTGAATGCCATCATGA

XM_009391309.2 AATAACGATGAAGGATCTGGAGGACCAGAATTGTTTG--------CGTGA

NM_001084343.2 --------------------------------------------------

NM_001159194.1 --------------------------------------------------

XM_015789203.1 --------------------------------------------------

XM_015774497.2 --------------------------------------------------

NM_001153709.2 --------------------------------------------------

NM_001254770.2 --------------------------------------------------

NM_001150750.2 --------------------------------------------------

CM007650.1_142530856-142531634 --------------------------------------------------

CM007648.1_220493755-220494609 --------------------------------------------------

NM_001111800.2 --------------------------------------------------

XM_023301819.1 --------------------------------------------------

XM_015775299.2 --------------------------------------------------

XM_009384249.2 --------------------------------------------------

XM_009385622.2 --------------------------------------------------

XM_009388210.2 --------------------------------------------------

XM_009389661.2 --------------------------------------------------

XM_009393219.2 --------------------------------------------------

XM_009415859.2 --------------------------------------------------

XM_008649541.3 --------------------------------------------------

XM_015782909.2 --------------------------------------------------

NM_001349820.1 --------------------------------------------------

EU837258.1 --------------------------------------------------

EU847517.1 --------------------------------------------------

XM_015775100.2 --------------------------------------------------

CT833260.1 --------------------------------------------------

AK067060.1 --------------------------------------------------

XM_015795858.2 --------------------------------------------------

EU847519.1 --------------------------------------------------

FR720457.1 TAGAATATAAAAAACAAAGAATCGATACGTATAGGTGTCAGCTCTATAAA

EU847520.1 --------------------------------------------------

NM_001143039.1 --------------------------------------------------

AK073133.1 --------------------------------------------------

XM_006380178.2 --------------------------------------------------

XM_002315454.3 --------------------------------------------------

XM_008392395.2 --------------------------------------------------

XM_029095961.1 --------------------------------------------------

XM_002272390.4 --------------------------------------------------

NM_001256464.1 --------------------------------------------------

XM_003546320.5 --------------------------------------------------

NM_001279196.2 --------------------------------------------------

AY035100.1 --------------------------------------------------

JF968116.1 --------------------------------------------------

JF968119.1 --------------------------------------------------

NM_130320.4 --------------------------------------------------

NM_001248198.2 --------------------------------------------------

AY192368.1 --------------------------------------------------

NM_001247379.2 --------------------------------------------------

JX145122.1 --------------------------------------------------

XM_002301454.4 ATCAATAAAATTATTATCC-----TTCCCTTCA-----------------

XM_002320960.3 TCTGATGAAATTTCTATCC-----TTCAATTTCGTGTTTTTGTTATGAAT

NM_001320016.1 ACAATTGCAACTCTGTTTA-----AATTATTCAATTTTTATTCTAATCAA

NM_001328756.1 AAAAAAAAAA----------------------------------------

FQ392750.1 AATTGTCGAATAAAATTCA-----GCGGCCTAATTCCGCGTTTTTGGAAA

NM_001254494.3 TCTATTTGAATTCCAATTCCAATTATTCTTTCGGTGAATTTTATCTATTA

NM_001254517.2 TTTTGTTGAATTGTAATTT------TTCTTTTGGTGG--TATGTCGAACT

AK316980.1 AAAAA---------------------------------------------

NM_180251.3 TTTATTAGTCTTCTGAGCTCTCTTTTTGATCTTTGTTATAGCGTATCATC

JF968115.1 AGTGTTCTTACTTACCTG---------TGCCGTTATGTTG----------

JF968117.1 AGTCCTCTTACTTATCTGCCAAGGTTGTGCCATTATGTTGCAGTTGTGCG

JF968118.1 --------------------------------------------------

NM_001247584.2 A-------------------------------------------------

KJ401124.1 --------------------------------------------------

XM_004252354.4 AGTTTCCAAATTTTGCATCAATGTGGATGATAATTGATGGTTCAAAATGC

XM_002266972.5 AATTTTATGTCTGTATCTTGAAGATTATATGGGTTGGAAGACTGAAACCT

XM_024596482.1 TTCTCAGGAGCTTTTTCCATATAAAAGTAGTACATTCGCTTTC----CCG

XM_006369031.3 TTCTCAGGAGCTTTTGCCATATAAAAGTAGGAGTTGGGTATTTAGTTCCC

XM_008341964.3 TAAGCCGTAGCTAATGGCGTATGGAGATCGTA--TAGGTAATG----GGT

NM_001294046.1 TAAGCCGTAGCTAATGTCATATGGAGAGCGTG--TAGGTATTA----GGT

NM_001251371.2 AGTTAAAACAATTAACTCGCTCAAGCATCTCTTGTATCTAAAGTATGGGG

XM_006604868.4 AGTTAAAACAATTTATTT----AGGTGCT---------------------

NM_001354284.1 -CTCAAGAGACCTCGTTTTCA-GGGGATTTTCTGTTTGATGTCCATAAGG

NM_001349033.1 -CTCAA-AGACCTCGTTTTCA-GGGGATTTTCTGTTTGATGTCCTTAAGG

NM_001155962.1 GACAATGTTTATATAGTATCTATACTATGTTGGTGCGTCGATG---GTTC

NM_001157201.2 GACTGGTTCAATAATTCACCTTTGCC-TACCGTCGTACCGCCG---GCCT

AK111690.1 GACTGATTCAAATAACTGCCTTGGCCATGTGTGTGCTGTAATGT--GCTT

XM_008660266.4 TATGTGTC--TGCTCCTTTTATTTTCTTGTCTTTGCTTCAAAAAAATGGC

XM_015788266.2 TACTTAGC--TTCTACCTTA-----TCTGTACTTGCT-----ATAGTGGC

NM_001156774.2 CATTTTG---TTTTGCTTTCT------TTTCGTTGTTACA-ATATATGGT

NM_001155219.2 CATTTTACCATTTTGTTTTCCCCCTTCTTTCGTTGTTACA-GTATATGGT

EU837255.1 TGCTTTTTTTTTTCCGTTCTAGTTTTCTGTCGTTGCTACCTGTATATGGC

XM_009403037.2 AATTTGGGTCCTTTAAGTTGGTTGTATTAAGTATAGGCTGAAGACTGTTT

XM_018823871.1 AGTCTGGGTTGTCTATGTCGGACATGTTATGGGTATGACGAAATCTATTG

XM_009419716.2 AATTCAGGGTTCTAATG-----AACCTTTGATTTATGTTACTATCTATC-

XM_009419259.2 -ACTTGGACCTTCTTAGT----TAGCTTGTGGTTTTGTCGAAAAC-----

XM_009415720.2 GACTTGGTTATGCTTACT----TGTCTGGTGGCTATGTGGAAAATGGAAC

XM_009411978.2 GATTTGGCTGTATGTCT----GATACTGCTAATGGAGATGCTTGTAGACA

XM_009383333.2 AATTTTACCATCTCTCTTTAGGATTCTGAGTATAGGAGTTCTGTTGGAGT

XM_009381868.2 AACA----------------------------------------------

XM_009405824.2 G-CATGCT--TGGTTTCTTTTGCTAGTCATGGCCAGAAATACTAG--ATC

XM_009389352.2 ATCAAAGTGT-GTTTGGTTTGCAGCATAATCTTCAGGAACAACAGCTTTT

XM_009391309.2 ATCAGTATGTTGTTTGCTTTTGGACTTGAGTTTTATCATTA-----TTCT

NM_001084343.2 --------------------------------------------------

NM_001159194.1 --------------------------------------------------

XM_015789203.1 --------------------------------------------------

XM_015774497.2 --------------------------------------------------

NM_001153709.2 --------------------------------------------------

NM_001254770.2 --------------------------------------------------

NM_001150750.2 --------------------------------------------------

CM007650.1_142530856-142531634 --------------------------------------------------

CM007648.1_220493755-220494609 --------------------------------------------------

NM_001111800.2 --------------------------------------------------

XM_023301819.1 --------------------------------------------------

XM_015775299.2 --------------------------------------------------

XM_009384249.2 --------------------------------------------------

XM_009385622.2 --------------------------------------------------

XM_009388210.2 --------------------------------------------------

XM_009389661.2 --------------------------------------------------

XM_009393219.2 --------------------------------------------------

XM_009415859.2 --------------------------------------------------

XM_008649541.3 --------------------------------------------------

XM_015782909.2 --------------------------------------------------

NM_001349820.1 --------------------------------------------------

EU837258.1 --------------------------------------------------

EU847517.1 --------------------------------------------------

XM_015775100.2 --------------------------------------------------

CT833260.1 --------------------------------------------------

AK067060.1 --------------------------------------------------

XM_015795858.2 --------------------------------------------------

EU847519.1 --------------------------------------------------

FR720457.1 AAAACCGACACATATATTATAGGCATCGGATATATACGCATGGCTAGTGT

EU847520.1 --------------------------------------------------

NM_001143039.1 --------------------------------------------------

AK073133.1 --------------------------------------------------

XM_006380178.2 --------------------------------------------------

XM_002315454.3 --------------------------------------------------

XM_008392395.2 --------------------------------------------------

XM_029095961.1 --------------------------------------------------

XM_002272390.4 --------------------------------------------------

NM_001256464.1 --------------------------------------------------

XM_003546320.5 --------------------------------------------------

NM_001279196.2 --------------------------------------------------

AY035100.1 --------------------------------------------------

JF968116.1 --------------------------------------------------

JF968119.1 --------------------------------------------------

NM_130320.4 --------------------------------------------------

NM_001248198.2 --------------------------------------------------

AY192368.1 --------------------------------------------------

NM_001247379.2 --------------------------------------------------

JX145122.1 --------------------------------------------------

XM_002301454.4 --------------------------------------------------

XM_002320960.3 CTCAATTGTGTTTCAAACC-------------------------------

NM_001320016.1 AAAAAAAAAA----------------------------------------

NM_001328756.1 --------------------------------------------------

FQ392750.1 AAAAAAAAAAAAAAAAA---------------------------------

NM_001254494.3 ATTCATACCACTTGTTGTTTATGTTAATGTCGTCTCCATCCCTAATTCGA

NM_001254517.2 GTA-ATTTTACTAGATATAT---TCAACCGTGTTTGAATTCCAAAAAAAA

AK316980.1 --------------------------------------------------

NM_180251.3 ACCCTCGAAAGTGTAATGTTTTGTACCCCCAAACTTGTTTAGCATTATAA

JF968115.1 --TAGGTAACATTTTAGGCCCTCTTTTATTACA-ATCCAAAGACCTGAGA

JF968117.1 TTTAGGTAACATATTAGGCCTTCTTTTATTACATATCCAAAAACCTGAGA

JF968118.1 --------------------------------------------------

NM_001247584.2 --------------------------------------------------

KJ401124.1 --------------------------------------------------

XM_004252354.4 TTTAATATTTCACAACTTGGGTGCTTGACATTGTTTATCCTATATTGCAT

XM_002266972.5 GATAAAATACCACCTTTGCTGTTTGTTTCAAA------------------

XM_024596482.1 TTTTCATTACATTTTAAGTCATTGGACTTTGGAA-GTTGGA-TTCTATGA

XM_006369031.3 GTTCTGTTGCATTTGAAGTCATTGAACTTGGGAATGTTGGA-TATGATGA

XM_008341964.3 CCACTTTCTCGTCCAAAGA------ACTTGTGGTTTCTGAACTTTCATGT

NM_001294046.1 TTACTTTCACCTCCAAAGAGCTTGAACTTGTGGTTTCTCAACTTTGATGT

NM_001251371.2 CTATATAGTTATTTAGGTGTCCTTTGCCTTGTCAAAGGCCTTAGTTTTGG

XM_006604868.4 --------------------------------------------------

NM_001354284.1 ATTATCAATTAT---GTTATATATGGTCTGGGATACAGTCTTGACT----

NM_001349033.1 ATTATCAATTAT---GTTATATATGGTCTTGGATACAGTCTTGACTGCTG

NM_001155962.1 AAAAAAAAAAA-----AAAA------------------------------

NM_001157201.2 GCTGGACTGTA-----GTGTGCGTACTGCATATATATGCCATAGTTATGT

AK111690.1 ATTAAGTTATATAGTTGTGCTCATATTGCGTATCTACG------------

XM_008660266.4 TCTGAACATTGCGAGTTTGTACT---TTGTAGACAATATATA-TATATA-

XM_015788266.2 TGTGAACCTTGTGGATCTGAACT---CTGAAGCCAATGTTTACTATATAA

NM_001156774.2 TT--AACATTATGA-TTTATGGA---TATGTG-CCAAATATGGTGCTCAA

NM_001155219.2 TT--AACATTATGA-TTTATGGA---TATGTGGCCAAACATGCCTTTTAT

EU837255.1 TATGAACATCGTGAATCCATGGCCATTATGTTTTAATCTATGTTGTTGAC

XM_009403037.2 AAAGACTACTTTGCCTCTATGTTCCTCCTGTTGTTACTATGCAAGATTC-

XM_018823871.1 AAATACTGGTTTGGCTCTATGTTCA-CCTGTTGTTGCCATAGAAGATGC-

XM_009419716.2 -GCCACTATTTATGTCTATTGTCTGCTCTGTTGTCATGTAAAGTGTTTCC

XM_009419259.2 -----TTGCTTTCGCTCTATGTTTG--GCATTGTTATTATCAGTGATGC-

XM_009415720.2 AAATACTGCTTTATATCTATGTTTG--TCATTGTTGCTCTTGGAGATGC-

XM_009411978.2 TCATGGCATGCTTATTTTATG---GTTCAATGGCTACCTTCCATATTTA-

XM_009383333.2 ACTGGATTTGCAATCCTTATGTTGGTTAAACAATCAGTGTATTAGCAAC-

XM_009381868.2 --------------------------------------------------

XM_009405824.2 AGTTATGAGGTCATGGAGATGGTGGACACAAGGTTGCTGCAATTAAAGTG

XM_009389352.2 TGTTGCTGCTTTGTGGACTCTCGAGTTTACCAGTGTTGACTGTTTCTGTA

XM_009391309.2 GACTA---------------------------------------------

NM_001084343.2 --------------------------------------------------

NM_001159194.1 --------------------------------------------------

XM_015789203.1 --------------------------------------------------

XM_015774497.2 --------------------------------------------------

NM_001153709.2 --------------------------------------------------

NM_001254770.2 --------------------------------------------------

NM_001150750.2 --------------------------------------------------

CM007650.1_142530856-142531634 --------------------------------------------------

CM007648.1_220493755-220494609 --------------------------------------------------

NM_001111800.2 --------------------------------------------------

XM_023301819.1 --------------------------------------------------

XM_015775299.2 --------------------------------------------------

XM_009384249.2 --------------------------------------------------

XM_009385622.2 --------------------------------------------------

XM_009388210.2 --------------------------------------------------

XM_009389661.2 --------------------------------------------------

XM_009393219.2 --------------------------------------------------

XM_009415859.2 --------------------------------------------------

XM_008649541.3 --------------------------------------------------

XM_015782909.2 --------------------------------------------------

NM_001349820.1 --------------------------------------------------

EU837258.1 --------------------------------------------------

EU847517.1 --------------------------------------------------

XM_015775100.2 --------------------------------------------------

CT833260.1 --------------------------------------------------

AK067060.1 --------------------------------------------------

XM_015795858.2 --------------------------------------------------

EU847519.1 --------------------------------------------------

FR720457.1 CGGCACCTATGGCTAGTTTCTAACATATTGGTGCCGGCTAGGAAACTGGC

EU847520.1 --------------------------------------------------

NM_001143039.1 --------------------------------------------------

AK073133.1 --------------------------------------------------

XM_006380178.2 --------------------------------------------------

XM_002315454.3 --------------------------------------------------

XM_008392395.2 --------------------------------------------------

XM_029095961.1 --------------------------------------------------

XM_002272390.4 --------------------------------------------------

NM_001256464.1 --------------------------------------------------

XM_003546320.5 --------------------------------------------------

NM_001279196.2 --------------------------------------------------

AY035100.1 --------------------------------------------------

JF968116.1 --------------------------------------------------

JF968119.1 --------------------------------------------------

NM_130320.4 --------------------------------------------------

NM_001248198.2 --------------------------------------------------

AY192368.1 --------------------------------------------------

NM_001247379.2 --------------------------------------------------

JX145122.1 --------------------------------------------------

XM_002301454.4 --------------------------------------------------

XM_002320960.3 --------------------------------------------------

NM_001320016.1 --------------------------------------------------

NM_001328756.1 --------------------------------------------------

FQ392750.1 --------------------------------------------------

NM_001254494.3 GGATGGAGAACAGTCAAGAGCATGACTCGGGCAAGCATTACCGCGTGAGG

NM_001254517.2 AA------------------------------------------------

AK316980.1 --------------------------------------------------

NM_180251.3 TAAAGTCTCTTTGGAACTTCTCTATCTGTTCAATGAATCTTCTCTGCC--

JF968115.1 CTGTATATTTATGTATGATCAAAAGCTGTGCTGTTAGCTTGATCATCCTT

JF968117.1 CTGTA-ATTT---TATGATCACAAGCTATGCTGT-AGCTGGATCAATCTT

JF968118.1 --------------------------------------------------

NM_001247584.2 --------------------------------------------------

KJ401124.1 --------------------------------------------------

XM_004252354.4 ATGCTGACTGAGAAGGCTTTTTGCTCA-----------------------

XM_002266972.5 --------------------------------------------------

XM_024596482.1 CTGTAATTTGACAATGTCGTGTGATTTATTTTG-AGAACTGAAGCCTTAA

XM_006369031.3 CTGTAAAT-GACAATATTGTGTGATTTGTGTTG-AAGACTAAATCCATAA

XM_008341964.3 TTGTAAC--GATGAAATTGTGTGAGTTATGATG--ATACTGAAACCTCAT

NM_001294046.1 TTGTATT--GATGAAATTGTGTGAGCTATGATGGAATGCTGAAACCTCAT

NM_001251371.2 GGAAATCTCGATTGTTGTTCGAGGGTGATTATATATGATCTGTGAAACAA

XM_006604868.4 --------------------------------------------------

NM_001354284.1 -TACAGTTTTT----AACTTGGTTGTTTTCATT------------GTTAT

NM_001349033.1 ATACAGTTTTTTTTTAACTTGGTTGTTTTCATTTTCATTATTATTATTAT

NM_001155962.1 --------------------------------------------------

NM_001157201.2 ATGCACTCCTCCTATATATACTATATATTATTA-----------------

AK111690.1 --------------------------------------------------

XM_008660266.4 --TATATGTGTGTATGTGCTTGGTTTGCTTCA------------------

XM_015788266.2 TGTGGTTGGTTTTATAAACTCTAGTTGATTTGGACCCCTGTCAA------

NM_001156774.2 C-AGTGGTCAAACATGCCTTTTAGATGCTCTTGTTTCTCGTATTGTTGTG

NM_001155219.2 A-AAAAAAAAAAAAAA----------------------------------

EU837255.1 T-GCTCAAAAAAAAAAAAAAAAA---------------------------

XM_009403037.2 -TCTAGACATGATGGTTCCTCTATTTGATCT-------------------

XM_018823871.1 -CCTGGACATAATGATTAATGTGCTTAAGCTTATGCTGCGATCCCTCTTG

XM_009419716.2 CTCTAGAATTCAGGGTTC-TATGTTC------------------------

XM_009419259.2 --ATGGACATGATTGTGCTTATGCTTATTT-TGCAGTTCTTATCC-----

XM_009415720.2 --TTGGACATCCTTCTTTTGAAGTTAAACAGTACATCCTTCAA-------

XM_009411978.2 ---TGTTA------------------------------------------

XM_009383333.2 ---TGTTGATTATTGTTTTGGCTTTATGCCTGATATTTCTAATGAAGATA

XM_009381868.2 --------------------------------------------------

XM_009405824.2 TATGGTCTATGATGAGAATTTGAAATAAATCTGGATATTAATGGCCATTG

XM_009389352.2 TATGATGTAAGCTAATGGTTTAAGACATGCTGCTGAACCTGGTTCCCTCG

XM_009391309.2 --------------------------------------------------

NM_001084343.2 --------------------------------------------------

NM_001159194.1 --------------------------------------------------

XM_015789203.1 --------------------------------------------------

XM_015774497.2 --------------------------------------------------

NM_001153709.2 --------------------------------------------------

NM_001254770.2 --------------------------------------------------

NM_001150750.2 --------------------------------------------------

CM007650.1_142530856-142531634 --------------------------------------------------

CM007648.1_220493755-220494609 --------------------------------------------------

NM_001111800.2 --------------------------------------------------

XM_023301819.1 --------------------------------------------------

XM_015775299.2 --------------------------------------------------

XM_009384249.2 --------------------------------------------------

XM_009385622.2 --------------------------------------------------

XM_009388210.2 --------------------------------------------------

XM_009389661.2 --------------------------------------------------

XM_009393219.2 --------------------------------------------------

XM_009415859.2 --------------------------------------------------

XM_008649541.3 --------------------------------------------------

XM_015782909.2 --------------------------------------------------

NM_001349820.1 --------------------------------------------------

EU837258.1 --------------------------------------------------

EU847517.1 --------------------------------------------------

XM_015775100.2 --------------------------------------------------

CT833260.1 --------------------------------------------------

AK067060.1 --------------------------------------------------

XM_015795858.2 --------------------------------------------------

EU847519.1 --------------------------------------------------

FR720457.1 ACCTATGGTCAATTTCCAACCGGCACTACTTATATTATGGCGATTTTTGT

EU847520.1 --------------------------------------------------

NM_001143039.1 --------------------------------------------------

AK073133.1 --------------------------------------------------

XM_006380178.2 --------------------------------------------------

XM_002315454.3 --------------------------------------------------

XM_008392395.2 --------------------------------------------------

XM_029095961.1 --------------------------------------------------

XM_002272390.4 --------------------------------------------------

NM_001256464.1 --------------------------------------------------

XM_003546320.5 --------------------------------------------------

NM_001279196.2 --------------------------------------------------

AY035100.1 --------------------------------------------------

JF968116.1 --------------------------------------------------

JF968119.1 --------------------------------------------------

NM_130320.4 --------------------------------------------------

NM_001248198.2 --------------------------------------------------

AY192368.1 --------------------------------------------------

NM_001247379.2 --------------------------------------------------

JX145122.1 --------------------------------------------------

XM_002301454.4 --------------------------------------------------

XM_002320960.3 --------------------------------------------------

NM_001320016.1 --------------------------------------------------

NM_001328756.1 --------------------------------------------------

FQ392750.1 --------------------------------------------------

NM_001254494.3 TCCTACAATGGGAATAATATTCCAAGCATTTTTGGTTACGCGCCGGAAAA

NM_001254517.2 --------------------------------------------------

AK316980.1 --------------------------------------------------

NM_180251.3 --------------------------------------------------

JF968115.1 CAATGCTTTATGACTTCTGAAAGATTCTTTCTGCTGTCTGTAATATTCAG

JF968117.1 TAATGCTTTATGACTTCTGAAAGATTCTGTCTAGTGTCTGTAATATTCAG

JF968118.1 --------------------------------------------------

NM_001247584.2 --------------------------------------------------

KJ401124.1 --------------------------------------------------

XM_004252354.4 --------------------------------------------------

XM_002266972.5 --------------------------------------------------

XM_024596482.1 ATTTCAACTTTGTGGTTGGTTTTCCCCCGTGGCCATCATTTTTGAATCCT

XM_006369031.3 ATTTCAACTCTTCTGTTGGTTTCCCCCA----------------------

XM_008341964.3 AATTCTGTACTTGTTTAAATTTATTTCGGTCTTCCTTTTTACAAGAGGCC

NM_001294046.1 AAATCTGTACTTGTTTAAATTAAAAAAAAAAAA-----------------

NM_001251371.2 TCTTGGCTGCTGATAATTTTTAACTGGGTTGTTTCTCTATGGTTAAAAAA

XM_006604868.4 --------------------------------------------------

NM_001354284.1 GGCAATTTGATTATGA-TTTGAGACCAGCCATTTTTCAAAAAAAAAAAAA

NM_001349033.1 GGCAATTTGATTATGACTTTGAGACCAGCCATTTCTCAA-----------

NM_001155962.1 --------------------------------------------------

NM_001157201.2 --------------------------------------------------

AK111690.1 --------------------------------------------------

XM_008660266.4 --------------------------------------------------

XM_015788266.2 --------------------------------------------------

NM_001156774.2 TACCTTGTGATATTTCATTCGTTGAAATTGAGTTAAACTAGTTATCTGCT

NM_001155219.2 --------------------------------------------------

EU837255.1 --------------------------------------------------

XM_009403037.2 --------------------------------------------------

XM_018823871.1 TGGGTTATTCACTGGGGTTGAGATTAATTTTTCAGATTCAAGTGTCATGG

XM_009419716.2 --------------------------------------------------

XM_009419259.2 --------------------------------------------------

XM_009415720.2 --------------------------------------------------

XM_009411978.2 --------------------------------------------------

XM_009383333.2 CCTGTGGACA----------------------------------------

XM_009381868.2 --------------------------------------------------

XM_009405824.2 TTTCAAAACTTTTTTGTGGTTAGTGAGAATTCAAACTCACTTATT-----

XM_009389352.2 TGTGCTTTATTTTCGTTGCTTTCTCAATATTGGAACTGATGTGAAGCCAA

XM_009391309.2 --------------------------------------------------

NM_001084343.2 --------------------------------------------------

NM_001159194.1 --------------------------------------------------

XM_015789203.1 --------------------------------------------------

XM_015774497.2 --------------------------------------------------

NM_001153709.2 --------------------------------------------------

NM_001254770.2 --------------------------------------------------

NM_001150750.2 --------------------------------------------------

CM007650.1_142530856-142531634 --------------------------------------------------

CM007648.1_220493755-220494609 --------------------------------------------------

NM_001111800.2 --------------------------------------------------

XM_023301819.1 --------------------------------------------------

XM_015775299.2 --------------------------------------------------

XM_009384249.2 --------------------------------------------------

XM_009385622.2 --------------------------------------------------

XM_009388210.2 --------------------------------------------------

XM_009389661.2 --------------------------------------------------

XM_009393219.2 --------------------------------------------------

XM_009415859.2 --------------------------------------------------

XM_008649541.3 --------------------------------------------------

XM_015782909.2 --------------------------------------------------

NM_001349820.1 --------------------------------------------------

EU837258.1 --------------------------------------------------

EU847517.1 --------------------------------------------------

XM_015775100.2 --------------------------------------------------

CT833260.1 --------------------------------------------------

AK067060.1 --------------------------------------------------

XM_015795858.2 --------------------------------------------------

EU847519.1 --------------------------------------------------

FR720457.1 ACTTAGTGATTATCTCGTTTAATATTTTCTTCTCTTGGTCTTAACAGGGG

EU847520.1 --------------------------------------------------

NM_001143039.1 --------------------------------------------------

AK073133.1 --------------------------------------------------

XM_006380178.2 --------------------------------------------------

XM_002315454.3 --------------------------------------------------

XM_008392395.2 --------------------------------------------------

XM_029095961.1 --------------------------------------------------

XM_002272390.4 --------------------------------------------------

NM_001256464.1 --------------------------------------------------

XM_003546320.5 --------------------------------------------------

NM_001279196.2 --------------------------------------------------

AY035100.1 --------------------------------------------------

JF968116.1 --------------------------------------------------

JF968119.1 --------------------------------------------------

NM_130320.4 --------------------------------------------------

NM_001248198.2 --------------------------------------------------

AY192368.1 --------------------------------------------------

NM_001247379.2 --------------------------------------------------

JX145122.1 --------------------------------------------------

XM_002301454.4 --------------------------------------------------

XM_002320960.3 --------------------------------------------------

NM_001320016.1 --------------------------------------------------

NM_001328756.1 --------------------------------------------------

FQ392750.1 --------------------------------------------------

NM_001254494.3 AACATTTAATATACTGTCTCGACAAAAATTGCAAATGTTTTTACTATGTT

NM_001254517.2 --------------------------------------------------

AK316980.1 --------------------------------------------------

NM_180251.3 --------------------------------------------------

JF968115.1 GATTCTATTCTCTGCTGCTTGAAATGCAATGATGTATACTTTTGGTTAGC

JF968117.1 GATTCTATTCTCTGCTTCCTCAAATGCAATGATGTATGCTTTAC------

JF968118.1 --------------------------------------------------

NM_001247584.2 --------------------------------------------------

KJ401124.1 --------------------------------------------------

XM_004252354.4 --------------------------------------------------

XM_002266972.5 --------------------------------------------------

XM_024596482.1 GATTAACCA-GTACTTTGTATTGATAAATTCCTGAAAGAAAAAGTTGCTT

XM_006369031.3 --------------------------------------------------

XM_008341964.3 ATCAGATTTTGCATTGTTTGGTTTTCAGTTCTATGTACTTTTGACTTGGT

NM_001294046.1 --------------------------------------------------

NM_001251371.2 AAAAAAAAA-----------------------------------------

XM_006604868.4 --------------------------------------------------

NM_001354284.1 AA------------------------------------------------

NM_001349033.1 --------------------------------------------------

NM_001155962.1 --------------------------------------------------

NM_001157201.2 --------------------------------------------------

AK111690.1 --------------------------------------------------

XM_008660266.4 --------------------------------------------------

XM_015788266.2 --------------------------------------------------

NM_001156774.2 GAAACTTTTCTTGTACAAGCAAAAGTATTCAAGATTTAATCTTATTGGCA

NM_001155219.2 --------------------------------------------------

EU837255.1 --------------------------------------------------

XM_009403037.2 --------------------------------------------------

XM_018823871.1 AGGAGGCTGCTGCAAAGCTGAATGTTGACTAGGAATCTGAGCTTTTAAAG

XM_009419716.2 --------------------------------------------------

XM_009419259.2 --------------------------------------------------

XM_009415720.2 --------------------------------------------------

XM_009411978.2 --------------------------------------------------

XM_009383333.2 --------------------------------------------------

XM_009381868.2 --------------------------------------------------

XM_009405824.2 --------------------------------------------------

XM_009389352.2 AAGGATGACCTTTAGTCGTTGTTGCACAGATGCAAATATATCATGTATCA

XM_009391309.2 --------------------------------------------------

NM_001084343.2 --------------------------------------------------

NM_001159194.1 --------------------------------------------------

XM_015789203.1 --------------------------------------------------

XM_015774497.2 --------------------------------------------------

NM_001153709.2 --------------------------------------------------

NM_001254770.2 --------------------------------------------------

NM_001150750.2 --------------------------------------------------

CM007650.1_142530856-142531634 --------------------------------------------------

CM007648.1_220493755-220494609 --------------------------------------------------

NM_001111800.2 --------------------------------------------------

XM_023301819.1 --------------------------------------------------

XM_015775299.2 --------------------------------------------------

XM_009384249.2 --------------------------------------------------

XM_009385622.2 --------------------------------------------------

XM_009388210.2 --------------------------------------------------

XM_009389661.2 --------------------------------------------------

XM_009393219.2 --------------------------------------------------

XM_009415859.2 --------------------------------------------------

XM_008649541.3 --------------------------------------------------
[truncated: 107,281 more chars]
